# Supplementary material for: Special Issue: Gender and Health
Source: Glob Health Action. 2015 Jan 23;8:10.3402/gha.v8.27362. doi: 10.3402/gha.v8.27362 (PMC4306759; doi:10.3402/gha.v8.27362)
Supplement: Related texts

# Sub article: Impact of HIV on and the constructions of masculinities among HIV-positive men in South Africa: implications for secondary prevention programs

**DOI:** 10.3402/gha.v7.24631

**Published:** 2014-10-01

**Authors:** Yandisa M. Sikweyiya, Rachel Jewkes, Kristin Dunkle

**Affiliations:** 1 Gender and Health Research Unit, Medical Research Council, Pretoria, South Africa;; 2 Department of Behavioral Sciences and Health Education, Rollins School of Public Health, Emory University, Atlanta, Georgia, USA

**Keywords:** Men, HIV, masculinities, coping and adjustment, South Africa

## Abstract

**Background:**

To date, whilst there have been many published studies exploring the links between masculinity and HIV, not much work has been done to explore how an HIV-positive diagnosis impacts men's sense of masculinity and contextualizing the masculinities as fluid and changing.

**Objective:**

To explore how human immunodeficiency virus (HIV) impacts the lives of men and their constructions of masculinity through interviews with 18 men living with HIV.

**Design:**

Qualitative study involving conveniently and purposively selected black South African adult men who lived with HIV. In-depth interviews were conducted with 18 men who resided in Johannesburg and Mthatha, South Africa.

**Results:**

Our analysis suggests that the performance of risky masculinity may influence the acquisition of HIV. Yet, it also reveals that HIV can have a significant effect on men and their masculinities. Men's constructions of harmful notions of hegemonic masculinity pre-HIV diagnosis negatively affected their help-seeking behavior and coping and adjustment to living with HIV, post-diagnosis. The dominant discourse that men are strong and healthy visibly presented challenges for men when faced with an HIV-positive status. They interpreted HIV diagnosis as a loss, a sign of failure as a man, and evidence of an inability to retain control. Being sick undermined their ability to perform roles expected of them, and this led to feelings of powerlessness, worthlessness, and distress.

**Conclusions:**

Interventions with men living with HIV need to provide safe spaces for men to critically explore gender and constructions of social identities and the pressures these place on men and implications for their health. With this approach, harmful constructions of masculinities may be challenged and mitigated, and this process may render men amenable to change.

Successes with highly active antiretroviral therapy (HAART) have led to decreases in mortality and morbidity from human immunodeficiency virus (HIV), and people living with HIV are living longer and more productive lives (1–3) . However, in some quarters there are concerns that as people who live with HIV feel better, their sexual behavior may become more risky, or not change (4, 5), with the chance that they acquire resistant strains of the virus or, if viral suppression is incomplete, spread HIV (6).

In South Africa, to be eligible for HAART, adults and adolescents must have a CD4 count of ≤350 cells/mm^3^ irrespective of the WHO clinical stage (7). With the promotion of mass early HIV testing, there are increasing numbers of people who know they have HIV, but are not eligible for HAART, and it is essential that we promote safe sexual practices with this group of people as a key priority for HIV prevention in the population, as well as enabling them to stay in the health system until they are ready to start ARVs (8).

For a number of years, there has been awareness that the focus of interventions with people living with HIV should include their current psychological state including adjustment problems, depression, and a tendency to use avoidance as a coping mechanism (8). These problems elevate the risk of a person living with HIV transmitting the virus (8). However, in the general population women are much more willing to test for HIV than men and enter care with a higher CD4 count (9–11) .

To date, whilst there have been a number of published studies exploring the links between masculinity and HIV (12, 13), very little work has been done exploring how an HIV-positive diagnosis affects men's sense of masculinity and contextualizing the masculinities as fluid and changing (14).

Gender theorists, following the lead of Connell (15), have argued that ideals of masculinity are fluid and dynamic, and within a given social setting there exist multiple masculinities (see also 16, 17). Connell and Messerschmidt (18) further argue that there is constant construction and reconstruction of masculinities as the situations or circumstances in which they were initially formed change over time.

However, among these masculinities there is a shared cultural model of masculinity that is viewed as an ideal (19, 20). This has been termed hegemonic masculinity, drawing from the Gramscian notion of hegemony which implies a dominance which is attained through a social agreement, rather than through violent subjugation of others (15). The social agreement, or acquiescence, extends to those who are disadvantaged by the hegemonic masculinity (15). Within a patriarchal social context, this includes women and girls, and some alternative masculinities (notwithstanding important minorities who resist or construct themselves in opposition to hegemonic masculinity) (20, 21).

Because hegemonic masculinity operates as a cultural model it pertains within a culture (17), and there may be multiple hegemonic masculinities (especially in multi-ethic settings) (17, 18). However, within a single culture there tends to be one hegemonic masculinity and other masculinities (e.g. determined by age or sexual orientation) are subordinated or protest masculinities (22). Within a single setting, these may share important features. As a cultural model and ideal (23), hegemonic masculinity has traits that are recognized as features of ideal manhood, and these inform the practices of men (21), but they do not serve to create ‘cookie cutter’ men. In fact most men do not behave according to the ideals of hegemonic masculinity in all their practices, or all the time. For some men, they aspire to be seen as occupying a hegemonically masculine position, but may not do so either because they cannot or in some respects they choose not to do so.

In South Africa, there is a body of scholarship on hegemonic masculinity which is in agreement about certain male characteristics that are valorized. Hegemonic masculinity, rooted in patriarchy, is fundamentally based on the idea of male superiority over women (15, 21), and South African men are expected to demonstrate this and demonstrate control over women (24). Although their power over women does not stem from the use of violence, violence is accepted as a strategy for control when ‘necessary’. South African men are expected to be heterosexual and to engage in conspicuous performance of heterosexuality and male desirability, in competition with other men (25).

A South African patriarch is also expected to be head of a household and a provider, although in practice this is emphasized in the masculinity of older men (26). Men are expected to be strong and tough, and by implication healthy (12, 27). Linked to this is the idea that men are supposed to be in control of their environment. Particularly at a younger age, this may be tested and demonstrated through being able to engage in risky demonstrations of heterosexuality (e.g. multiple partners) (14), without incurring penalties, such as getting HIV which may be seen as a reflection of weakness (21, 28).

From this, it can be anticipated that having HIV may be interpreted as reflecting an inadequate (or loss of) masculinity both because it shows lack of strength and toughness, but also because it reflects a failure of being in control. Thus, men living with HIV may experience an attachment of a negative or deficit masculine identity that is meant to show that they are no longer viewed as ‘real’ man. This was paralleled by Hunter (28), writing about Zulu men, who described *isoka* as a celebrated social identity that is given to men with multiple concurrent girlfriends (29) but if they impregnated a girlfriend they were seen as disgraced men (30). In other words, they were stigmatized. Goffman's seminal work on stigma in the 1960s highlights how stigmatized social positions were resisted by those who would occupy them, either through denial of the condition that was stigmatizing, or through ‘passing’ (an attempt to conceal or get away with not disclosing invisible stigma) as not having it (31). In the context of HIV, denial may take the form of not testing in the face of illness and efforts to pass may involve rejecting opportunities for antiretroviral therapy, support groups and safer sexual practices (especially the use of condoms) (21).

Much of the work on understanding masculinities has focused on particular groups (often identified by race, socio-economic status, location in space, and time or religion) (see 32–36) , and has often resulted in the description of one masculine position in the group studied (e.g. (37).

A weakness of this analysis is that it implies that the masculinity is fixed, either through suggesting that in the population studied there is just one masculinity, or else that among the individuals studied there is only one masculinity occupied. A strength of Raewyn Connell's analysis has been the emphasis on the dynamic and multiple nature of masculinities, which gives the possibility of not just multiple masculinities pertaining in an individual setting, but also an individual man occupying multiple masculine positions at different times and in different contexts.

The purpose of the present paper is to explore how HIV impacts the lives of men and their constructions of masculinity through interviews with 18 men living with HIV in South Africa.

## Material and methods

This study was conducted in Johannesburg in the Gauteng Province and Mthatha in the Eastern Cape Province. These two towns represent two contrasting settings in South Africa, i.e. urban and rural settings, respectively.

Johannesburg is the largest city in South Africa in terms of population, and also the provincial capital of the Gauteng Province, which is the wealthiest province in South Africa. In contrast, Mthatha is a small rural town located in the Eastern Cape Province-one of the poorest provinces in South Africa.

This paper draws on 18 in-depth interviews conducted with adult men living with HIV. All participants were black African men between the ages 18–49, and all but one identified themselves as heterosexual. Eleven men resided in Johannesburg central or in Soweto, a nearby township. The other seven men resided in Mthatha and surrounding communities.

Interviews were conducted between 2008 and 2010 by two male interviewers. Convenience and purposive sampling techniques were employed to recruit the men into the study (38, 39). In Johannesburg, we employed convenience sampling to recruit men who were attending two local clinics as out-patients, one in town and the other in Soweto. These clinics had a dedicated service for men living with HIV. The majority of the interviews in Johannesburg were conducted by a male research assistant who was in his late 20s and experienced in conducting qualitative interviews. Prior to conducting interviews, he was trained on research ethics and techniques of conducting in-depth interviews on sensitive topics. Whilst he worked and lived in Johannesburg, he did not know the men he interviewed prior to interviews. YS, who is an author is this paper, also conducted three interviews in Johannesburg, and all seven interviews in Mthatha. To recruit participants in Mthatha, we employed a purposive sampling technique. YS contacted a male colleague who worked in the HIV and health sector in Mthatha and requested him to assist in recruiting men who lived with HIV that he knew or had worked with in his health promotion programs.

There was no predetermined number of interviews to be conducted, rather we interviewed until saturation, i.e. when no new information was emerging in each question of interest to the study (39). Each participant was interviewed once. Researchers may have preconceptions about the study topic, and this may influence the data collected and its analysis. To reduce this potential bias, we used the technique of bracketing where we attempted to put aside what we thought we knew about the topic and remained curious and open to the information we were getting in the interviews and findings emerging from the data (38). Interviews in Johannesburg were a mixture of IsiZulu, SeSotho, and English, and interviews in Mthatha were conducted in IsiXhosa, the dominant language in the Eastern Cape Province.

The scope of enquiry was developed to guide the interviews. It included asking men to share their views and experiences on how being diagnosed with HIV had affected their lives, and how the diagnosis had made them to view themselves as men. We asked men to share life stories on how they have experienced living with HIV and how living with HIV had evolved over time, if it has. Men's sexual self-expression including sexual risk taking and protection was also explored for both the pre- and post-HIV diagnosis periods. We also explored whether men acted or behaved differently as men who lived with HIV when in public spaces as compared to when in private spaces, and whether was there a difference between pre- and post-diagnosis periods. However, new issues that were not initially in the scope of inquiry but had emerged during the course of the interview and were relevant to the study topic were explored in the interview, and then incorporated into the scope of enquiry for further exploration in subsequent interviews with other men. All interviews were audio-recorded.

### Data analysis

Audio-recordings of the interviews were transcribed verbatim and translated into English in preparation for data analysis. Data were analyzed inductively employing thematic content analysis. However, there were deductive elements to the analysis as YS who led the data analysis reviewed the literature to explore themes that had been presented in similar studies and thereafter checked for those in our data (38–40) .

YS first read the transcripts repeatedly to familiarize himself with the content of the transcripts (40). Thereafter, he established few broad codes which somewhat corresponded with the questions in the scope of enquiry (39). Thereafter, text which seemed to fit together was grouped under a specific code. Subsequent to this, he explored the data and identified numerous open codes. Further to this, similar and or related open codes were grouped together under clearly defined themes. All authors then explored the relationships between the themes and interpreted what they saw emerging, but also compared the findings with those of other studies and drew the conclusions presented in this paper.

### Participants and socio-economic circumstances

Participants gave varying reasons why they tested for HIV. Twelve men said they tested because they had been sick and a HIV test was recommended either by the treating doctor or nurse or family member. Four men said they tested following their intimate partners’ death which often was suspected to be AIDS related. Three men reported that they were encouraged by their partners to test, with some of the partners having first disclosed to the men to be living with HIV. One man said he tested for HIV as a requirement to participate in contact sport. At the time of the interviews, the period men had lived with HIV ranged from being recently diagnosed (approximately 2 weeks) to 8 years.

In terms of men's employment status, at the time of the interviews, three said they were working in the HIV field as lay counselors or doing other HIV voluntary work. Two men were self-employed, four said they were formally employed doing office-based work, while eight men said they were not working. We do not have information about one man's employment status. Furthermore, we did not collect information on the education level of the participants.

We asked men about intimate partners and 14 men said they were currently dating or had a sexual partner, and the number of sexual partners ranged from having one to four concurrent sexual partners, few men reported to be currently in a relationship with a woman who was living with HIV. Sixteen men reported that they had disclosed their HIV status to at least one person. However, two men said no one knew about their status.

### Ethical considerations

Ethics approval was given by the ethics committees of the University of the Witwatersrand, South Africa and Emory University, United States of America. The purpose of the study, procedures involved, risks and benefits, and informants’ rights were explained to the participants before they signed informed consent. A total of R50 (US $5.7) was given to the informants for taking part in this research. Data presented in this article were anonymously processed with names of all the informants replaced by pseudonyms and potential identifies removed. We envisaged that some men may have strong emotional reactions to some of the issues that will be discussed in the interviews. As such, we prepared study information pamphlets with a list of local services that men could contact for psychological assistance should they feel the need. However, none of the men requested or took up the offer for referral.

## Results

In the following section, we present a synopsis of the main results. We show that HIV testing can set in chain a set of experiences that result in radical change to a person's self-assessment of masculinity as well as public assessment, depending on experience of care seeking, ill health, disclosure (voluntary or involuntary) and reactions of others to this. Thus, presenting a situation in which masculinity appears at its most fluid, with multiple masculine positions (emphasized masculinity, traditional masculinity, and diminished masculinity) presented as being occupied or aspired to over time, and existing at one time within the imagination and practices of individual men. Additionally, we show that the context of change provides the possibility of reshaping of masculinity to construct masculinities (resourceful–responsible men) based on ideas of caring and protecting which are less sexually risky and enable treatment adherence. Furthermore, we illustrate how shifts in identity can have an enduring and critical impact on men's ability to access and adhere to care and safer sex practices, and highlight the possibilities this creates for intervention to promote easier adjustment to a HIV-positive status among men.

### Emphasized masculinity

Men's practices and masculine identities before they acquired HIV were only visible through their discourse of their practices and priorities around these in the interviews we conducted with them when they had already acquired HIV. In our analysis, we noted fluidity, and sometimes blending, of masculine positions within individual man. This fluidity seemed to depend on various factors including time, space occupied by men, and, through historical accounts, age and showed that these masculine positions are not fixed.

The accounts highlight many of the features which were mentioned above as being indicative of a dominant masculinity, especially among younger people where heterosexual performance is greatly emphasized. This is illustrated in the narrative below:

Well I have started using condoms now, I wasn't practicing safe sex and I had many girlfriends and me and my friends were competing about having many sexual partners though I was from a poor family but that didn't affect my performance in having many partners. (Menzi)

Menzi was one of the 11 men in the study who indicated that he had previously sought to occupy a position that would enable him to be recognized as a ‘real’ man. He describes competing with other men on his sexual performance, measured in terms of having more girlfriends. The competition with other men over girlfriends echoes that described by Wood and Jewkes (37) among young men in the rural Eastern Cape.

Men indicated that they were socialized to understand that to be a man they should take risks, be brave and demonstrate strength, have the ability to propose love to women, initiate and sustain relationships with multiple women; be dominant and in control in such relationships.

In this context, the performance of the emphasized masculinity seems to be deeply grounded on the socialization of these men. Luxolo further illustrates this when he explained:

And we were raised in a manner that we are men, you find that we behave in a manner that we like and we socialize [party] … do you understand? And as men we have to smoke and have the drink [alcohol] do you understand? To sleep [have sex] wherever and whenever and with whomever you meet, do you understand? You are a man. (Luxolo)

Whilst it may seem that Luxolo was generalizing, and perhaps exaggerating, he was also reflecting social norms of male behavior. It is not clear whether he was talking about himself here, but he suggests that he was, and other men in this study reported similar pressure and behavior in the interviews.

In the analysis we also noted that some of the men were clearly still identifying with the youthful masculinity and at the time of the interview had several sexual partners.

### Traditional masculinity

In this setting, the traditional masculinity position is considered a mature masculinity (30). This masculinity emphasizes the expectation that men should provide a secure home for the family, fulfill a role as providers, and ensure the survival of the family line through childbearing and raising of children (41).

In our analysis, there appeared to be a blurring between the emphasized and traditional masculine positions in this setting. However, we also noted a difference between these masculine positions in terms of views on what is successful manhood and practices associated with these. The main area of difference was that the emphasized masculine position is more a youth masculinity; in contrast the traditional masculinity position is a more mature position that is tightly correlated with particular expectations and responsibilities fitting of a man who has transited from being a youth to attaining the status of adulthood.

In our study some of the men were in a different life stage. Itumeleng and Jeff were cohabiting or married, and Thabo was divorced. Itumeleng stayed with his partner and a child in a flat in Johannesburg and was the main provider in the family. Jeff was married, and had three children.

Other men, faced with the loss of their youthful masculinity after HIV diagnosis, grieved. The interviews suggested that in so doing, they mostly did not hanker after the youthful position that they had occupied and lost, but rather lamented an inability to occupy the more traditional masculinity of Jeff and Itumeleng, the one hegemonic in this setting. In other words, they grieved the future they felt they could not attain and not the past that they had lost. Tshepo described his loss in terms of an inability to occupy the carefree hegemonic masculinity, i.e. the traditional position:

You know I like women, so for my situation now I can't have many of them and I should use protection every time and the other thing is that I can't have another child because of my status.

Jeff and Itumeleng were among the men who grieved a loss of traditional masculinity. They appeared to grieve an inability to occupy a masculine position which ostensibly it appeared they were already in. For these men, it appeared they were framing HIV as a loss and judging what is lost in terms of a traditional masculine position. In this respect, they were similar to many of the men who were still in a more youthful masculinity.

In the interviews, there was a great deal of angst among men when discussing issues of childbearing and fatherhood. Men who had no biological children showed strong emotions of sadness and regret when discussing this topic. For them, having HIV meant that they would not leave anyone behind to perpetuate their family name, proving them to be failures in the eyes of their families. The following narratives illustrate this anxiety among some men.

In my life I planned to have children but after finding out about my status I just knew that I won't have children because of my status. (Joel)

Eh indeed it does not make me to feel good for sex since I'm a man, I need to revive the family name so I cannot do that because I always do it with a condom … there is no choice that I can make because I will make a poor soul dirty (transmit HIV), it is better that I just …, use a condom. (Katlego)

In contrast, men who already had children worried about the potential inability to provide for them; about dying prematurely and leaving their children without a father to provide for them; and others recognized with sadness that they will not be able to have other children. For example, Jeff stated:

That one [having children], I dream about, … I tell myself as long as I've got two kids there's no more kids, there's nothing I can do I'll never have another kid.

Luxolo talked about his wish to live longer so that he could raise his children:

I wish to find myself staying in a house or next to my place or next to the kraal you see then spending my pension money … being that man who has extensive experience in life where they [children] keep coming in and out saying I have a certain problem daddy and share tirelessly with my children … that is the thing that scares me, that seems difficult for us as men in this HIV/AIDS issue. (Luxolo)

As we note in the extract below, being sick undermined men's ability to perform their roles and led to feelings of powerlessness, worthlessness, and distress:

So then I am sitting here on the bed just as the month is coming to an end I don't know how I will pay the rent, what are the kids going to eat, I wish to be well. (Luxolo)

Providing support to this notion of manhood, Katlego highlighted the importance for him, as a man, to find a job, work and provide for his family. He explained:

So it's like I am saying that I am washing cars in town at the rank. I am able to support my family with that money. (Katlego)

In this sample of men, the traditional masculine identity is largely an aspired one and appears to most be present as a preoccupation post-HIV diagnosis.

### Diminished masculinity

HIV compromised men's ability to sustain the traditional masculine position – one that is hegemonic in this setting, and in response, many of them found themselves forced into a deficit identity. That is one that was at odds with the traditional notions of masculinity they so aspired for (42). To date, there has been little emphasis in scholarship on the emotional vulnerability of South African men, with some notable exceptions (e.g. (23). In our analysis, the diminished masculine position was particularly characterized by feelings of helplessness, hopelessness, and men's interpretation of their lives as compromised or constrained by having acquired HIV. Nine men occupied this position after they were diagnosed with HIV.

There was some distinction among men who reported this. Men who had been recently diagnosed (approximately within 1 year), at the time of the interviews, were more likely to report the emotions mentioned above. Yet, men who had lived with HIV for more than a year were more likely to mention this position in their past. The latter group had over the years, departed from this position as they adjusted to their diagnosis (see also 10, 43). For example, Itumeleng who had been living with HIV for a prolonged period of time reflected:

After I tested positive I felt that something very important had been taken from me by somebody you see and that thing I will never get it again, so I felt very weak and I felt that I'm powerless, I'm powerless you know what I mean and ja you feel like that once you have acquired HIV, you become stuck and helpless you know and hopeless.

Feelings of dejection after diagnosis were reported by other men as well, best illustrated in Thabo's narrative below:

At first when I learned that I had acquired HIV, it wasn't easy for me and I used to lock myself in the bedroom especially when it was time for me to take vitamins, but my family would tell me that it is not the end of the world.

Several other men reported feeling diminished by having acquired HIV. In their narratives, there was a great sense of loss:

Things that affect men is losing dignity, self-esteem because as a man you are a leader but if you are living with HIV it is difficult to exercise that power … it can also affect your relationship with your partner … and as a father you have to be supportive to your family and how are you going to do that when you are the one who needs support. (Menzi)

A man looks and looks and realizes that this thing that he has involved himself in [acquiring HIV] makes his life cold. (Luxolo)

The practices that some men identified as vital and marking successful manhood included strength and bravery. As such, for them, having acquired HIV and being sick, being cared for by others in the family or community, or seeking help were signs of weakness which undermined their manhood.

You know when you get sick it's a problem because many people would want to know and if you tell them they will start to feel pity for you and it's something that men don't want … and culture really, men believe that clinics are for women and if a man goes to the clinic it means that he is weak. (Menzi)

Menzi clearly read his HIV status and its impact on his health in terms of his masculinity. It was unmasculine to be ill and pitied, and to attend a clinic to seek care. HIV represented weakness, and that was unmasculine (c.f. (44). This resentment of illness could also reflect an avoidance of the reality of living with HIV and a negative coping strategy.

Some men said that the HIV-positive diagnosis necessitated behavior change in their lives. Yet, for many, making such changes seem not to be out of their own volition, rather the changes ran counter to how they preferred to position themselves as a man. It seems changing their behavior was a burdensome process for them as Luxolo explained:

That indeed in the morning and evening I will take this thing [ARVs] and for the rest of my life I will never do anything else so they are just things that you see that we are totally forced into that you must do a certain thing, do not do a certain thing you see. (Luxolo)

It appears Luxolo felt that he was living a prescribed life, and that he had lost the freedom to live his life the way he desired. His lack of control over his own life again reflected a lack of access to the hegemonic masculinity.

For other men, the need to reduce sexual partners, and to use a condom consistently when having sex were some of the changes men felt were forced upon them because of their status. This is evidenced by Boitumelo's assertion that ‘using a condom is not easy sometimes’.

What is depicted in the above narratives as an occupied position by these men is diminished manhood, a loss position that was fairly consistently described as following the diagnosis.

### Coping mechanisms: avoidance or acceptance

The men interviewed spoke of the impact of HIV in diminishing their manhood as being intolerable. Thus, they sought ways of shaking off their feelings of inadequacy which in an immediate sense stemmed from their perception of diminished masculinity but this itself was a response to being told they had acquired HIV. The data suggest that there could be three ways of doing this. The first was through deceiving partners and using traditional healers and treatments rather than clinics. As shown in Table 1, many men only tested for HIV when they were sick, and some only sought medical help at the behest of their families, or on recommendation by the doctor or nurse consulted. Men in our study, such as Menzi above, described public health centers as spaces they were uncomfortable to access and resisted doing so.

**Table 1:** Sketches of research participants

Name	Relationship	Social position	Year diagnosed	Support group attendance	Province	Why tested	Date of interview	Disclosed
Luxolo	Dating	HIV volunteer work	2000	Yes	EC	Required for participating in sport	2009	Family/publicly/partners
Ntsikelelo	Casual sex partners	Self-employed	2002	Yes	EC	Was sick	2009	Mother/friends
Itumeleng	One partner	HIV counselor	1993	Yes	Gauteng	Seems was sick? Developed glands 2 days after diagnosed	05/2009	Family/church/partner/publicly
Jeff	Wife and three girlfriends	Working	2009	No	Gauteng	Was sick and doctor recommended testing	05/2009	No one
Tobias	One partner	Working	2009		Gauteng	Advised by girlfriend	05/2009	Younger brother
Katlego	One partner	Self-employed	2003	Yes	Gauteng	Was sick	05/2009	Family/friends/clients
Mbongeni	One partner	Working	2005	No	EC	Encouraged by partner (HIV +) to test. Previous partners had died	07/10/08	Partner/family/friends
Menzi	Four sexual partners	Working	2000	No	Gauteng	Was sick	07/10/08	Family/friends/partners (All partners HIV+)
Thabo	Divorcee and one partner	Not working	2008	Yes	EC	Was sick	12/11/2008	Family
Lebohang	No partner	Not working	2008	Yes	Gauteng	Was sick	13/11/08	Family/friends and neighbors
Joel	Two partners	Working in the HIV field	1995	Yes	EC	Was sick and partner died	14/11/2008	Significant others/family/partners
Tshepo	One partner	Not working	March 2008	Yes	Gauteng	Partner had died	15/11/08	Brother
Oupa	One partner	Not working	2005	No	Gauteng	Was sick and had TB	20/11/2008	Family/ partner and neighbors
Boitumelo	Two partners	Not working	2004		Gauteng	Was sick	03/12/2008	Family/friend and one partner
Johnson	Two sexual partners	Not working	March 2006	No	EC	Was sick and in prison	10/12/2008	Mother
Mthuthuzeli	Girlfriend (HIV+)	Not working	Late 2006	No	EC	Was sick	11/12/2008	Family/ friends/ ex-girlfriends and current girlfriend
Bophelo	No partner	_	12/2008	No	Gauteng	Partner suspected of having HIV	12/2008	No one
Benjamin	Abstaining and not dating since partner's passing	Not working	May 2008	No	Gauteng	Partner had died	03/12/2008	Family/friends

This type of denial was seen again in the men who did not disclose their status to their intimate and or sexual partners as they feared a negative reaction. They feared being blamed for placing their partners at risk of HIV, although ironically were continuing to do this by non-disclosure. Additionally, they feared that their acquisition of HIV may be linked to promiscuous behavior on their part. Some said this link between men's promiscuous behavior and HIV acquisition often lead to blame and stigmatization by others in their communities. Ntsikelelo's comment to follow is illustrative:

Ey some men are boisterous others are fearful they [partners] will say they acquired the virus maybe through bad behavior … most men think that they are going to break up with the person that they were dating, they are going to break up with their wives if they ever explain that they have the virus. (Ntsikelelo)

Earlier we argued that, for some men, having many sexual partners was an essential part of being a man, so in this context this appears paradoxical. The paradox can be explained through understanding that it is the failure to be able to have lots of partners and stay in control of the situation that is perceived as unmanly, in this case loss of control is demonstrated through having acquired HIV. Our data show that these men were aware of the importance of disclosing their status to their partners, yet they thought that disclosing their status was a huge risk for themselves, their relationships and families, and they were, at least initially, not prepared to do this.

This risk perception may have had obtained credence from the lived experiences of some of the men who said that after disclosing to their partners, they experienced severe and harsh backlash. They reported being blamed for transmitting the virus to the partners, ridiculed, and called names, and for some, their wives or girlfriends left them. For instance, when Luxolo disclosed his status publicly, he experienced hostile reaction from some of his former girlfriends and the community at large. He said:

I disclosed to the community over the radio … It was very hard I was ‘that’ person, I realized that I had my ex-girlfriends that I dated when I was still young and knowing that even then the virus was not existing then, I was being followed, insulted and I understood others were going to open cases [press criminal charges]. (Luxolo)

Assessing the risk as huge, some men concealed their status from their partners. This decision, however, had negative health consequences for men and their partners. Some men said they found it difficult to take their medication in the presence of their partners and were forced to hide their medication resulting in treatment non-adherence. Other men said they continued to have unprotected sex with their girlfriends or wives even though they knew they were potentially transmitting the virus to them.

Using traditional healers seems to have had been a preferred option by some of the men. When we interviewed Bophelo, he had recently been diagnosed with HIV and mentioned having asked someone to prepare traditional medicine for him to cure the HIV:

Interviewer: Can you tell me who have you told about your status?

Bophelo: No one, I only found out this week but I just asked someone to prepare me muti (traditional medicine) that heals HIV.

Interviewer: does that person have information about HIV?

Bophelo: Yes, he had AIDS, his wife died of AIDS and when he tested he was told he has got AIDS. So when he was at church he had a vision of muti that cures HIV. He drank the muti and when he went back to test he was told he was HIV negative. So he is committed to help others … I will take it (ARVs) but I will not stop taking the muti treatment because I know muti has helped many people.

Highlighting perhaps another reason for preferring *muti* as a treatment for HIV, Itumeleng located its use as an HIV treatment to cultural practices. He explained:

African [black] men wouldn't mind to drink imbiza [traditional medicine], to induce vomiting and do all these traditional rituals and don't bother about using the condom because they want to prove that that culture is more important to them than any other thing.

Notwithstanding public education around there being no HIV cure, some men consulted traditional healers because they still held out a hope for being cured. Others preferred traditional healers as they hoped that the latter would explain away their sickness in terms other than having acquired HIV.

Another avenue of avoidance of diminished masculinity was in alcohol and drug abuse (see also 46). Stanton and colleagues argue that coping through avoidance may predict maladjustment and lead to distress for those using it (45). In our study, while some men may have also been using these substances prior to HIV diagnosis, it seems for others, post-diagnosis substance abuse may have been a coping mechanism. For instance, some men reported that they drank heavily as ‘a way of dealing with the diagnosis’, ‘to relieve the pain accompanying diagnosis’, and ‘to make their lives bearable’. This is best captured in Jeff's interview to follow. At the time of his interview, he had recently been diagnosed and was visibly emotional and teary in the interview. He narrated how he felt and how he used alcohol as a way of dealing with the diagnosis, a coping strategy. He explained:

That's what I was trying to do by the time I was home, I couldn't eat, I couldn't sleep, but … I tried too much booze [alcohol], I'd say ‘hayi [no] this is crap’ … I can't sleep while I'm drunk, it's a waste of money, waste of time, waste of everything, you just go back there, do whatever.

Consistent with Jeff's experience, Luxolo highlighted the common use of excessive alcohol as a way of avoiding to confront the reality of the diagnosis. He posited:

Another person will tell himself that it's something that is waiting for me, I am going, as I think I am waiting for death, and some of them [men living with HIV] say I am leaving work, another person may end up telling himself that he will succumb to alcohol to try and dismiss this problem which he has in his head, if I am always drunk it seems I might feel okay.

The third coping mechanism resulted in a state of empowerment through acceptance of one's HIV-positive status. Almost all men interviewed spoke of the importance of accepting one's HIV status, but this is not to say that all of them had concluded this stage of acceptance. Some were clearly still battling to accept the HIV diagnosis. Notwithstanding this, our data suggest that reaching a stage of acceptance and disclosure of their status (sometimes publicly), facilitated the process of help-seeking and treatment adherence. Whilst there were men who had negative experiences of disclosure (e.g. stigmatization), there were those who said they received support from the people they disclosed to even if those had been hostile initially (c.f. 46).

Being accepted and supported by significant others was valued by and crucial in empowering men to move from the diminished masculine position and reconfigure another masculine identity which allowed them to live positively with HIV. The following narratives are illustrative:

Just living with HIV, one becomes depressed and wants to be alone, it takes time to adjust and accept until you reach a point where you need to choose the life that you want. (Lebohang)

Ja, I even go to church, church are the ones who will tell you that there is life even if you have got AIDS. If you go there those people are so supportive there, praying for you sometimes you can be …, I can even feel that I can live with those people who ever is there. (Jeff)

### Resourceful–responsible men

Post-diagnosis, nine men positively reconfigured their masculinities and repositioned themselves as leaders, HIV activists and advocates, and were viewed as resourceful persons in their communities (see also 47). In our analysis, we noted particular characteristics among these men. They were mainly those who had lived with the HIV for a prolonged period of time; were more likely to have been through extensive HIV counseling and education or had been part of a local support group for an extended period of time; had disclosed their status to their families, intimate partners, and community; had disclosed publicly about living with HIV.

Whilst most of these men had no formal training on HIV counseling and life skills, most had taken up the role of being HIV peer counselors and performed such roles principally drawing from their experience of living with HIV over a lengthy period of time.

In their narratives, there was a relatively strong desire to make the lives of other people living with HIV bearable or to protect others from acquiring HIV. In his interview, Luxolo spoke at length about the role he was playing to support other people living with HIV in his community. It seems he was motivated by the need to prevent emotional and physical pain and suffering that could potentially be experienced by people living with HIV, if there was no support. He elucidated:

Those who have just tested because I know the challenges and that they can …, so that they do not experience them the same way I did since I didn't have any support I didn't know anything. (Luxolo)

Mthuthuzeli and Ntsikelelo also reported that they encouraged other men who lived with HIV to seek help and adhere to their medication. They motivated these men through referring to their life stories of living with HIV, as examples:

I encouraged some guy at the clinic about taking treatment, he didn't believe that our medication will help him and I had to tell him about where I come from. I told him that taking medication helped me a lot. (Mthuthuzeli)

In his interview, Ntsikelelo narrated a story where he actively played a caring role where a man in his community was refusing help and not taking his medication:

I was looking after him, he was a person then who used to not attend the clinic even when he was sick no he was not attending the clinic, he used to be a person who will sit and not even take the tablets … but when he fell sick I made him to meet the social workers then so that the caregivers at the hospice … I said to them then they must look after him. (Ntsikelelo)

Boitumelo spoke about the role he was playing in his community to prevent people from acquiring HIV. He motivated them to use condoms consistently when having sex, and echoed the same sentiment that he did this to prevent other people from experiencing the suffering he did after he was diagnosed with HIV. He posited:

I tell them about condoms and also give them condoms to use because I don't want them to suffer the way I have suffered. (Boitumelo)

In some cases, men were sometimes asked by local families to talk with a family member who was suspected of being sick with an AIDS related disease, but was refusing to seek help or take medication.

Okay, my family and friends and even in the community when someone is ill they would call me but sometimes they would call me for a person who is about to die. (Joel)

These men derived a sense of importance and self-worth from occupying these caring positions in their communities, positions that are certainly counter to hegemonic ideals of masculinity. In the following extracts, we note the feeling of self-worth which, in turn, seems to have helped these men to regain self-respect and aided them in reconfiguring their masculinities into positive ones.

When they keep talking about the HIV/AIDS issue I sometimes disassociate myself from it, I'm always on this side where I am expected to help when they say there is a person with a problem, I ask myself who is helping them, how can I reach that person and be able to assist him? (Luxolo)

There's nothing like this that I am going to tell now that has made me happy in my life, it makes me feel good especially when I'm out with guys standing in front of young girls and boys 13 years old and sharing ideas with them telling me what they know especially about sex and sexuality and me sharing ja my experiences in life like in terms of sex and sexuality and leaving them with that question mark of deciding what to do, because such thing is taboo. (Itumeleng)

By protecting their sexual partners from acquiring HIV from them by consistently wearing condoms when having sex; abstaining from sex; reducing the number of sexual partners; these men viewed themselves as responsible man and protectors. These practices are often used to describe a mature and responsible man in this setting. Thus, on noting the role they were playing in their communities, this may have restored their sense of manhood and perhaps facilitated rationalization about being worthy men in a different way. The following narrative from Katlego supports our interpretation:

Katlego: So in order for me to come to that decision, I thought of that first, then I made a decision that I will always be a person that I will have sex with I will need to have a condom

Interviewer … how does that make you feel on the inside my brother?

Katlego: Eh it makes me very happy instead of killing the community … to protect another person.

## Discussion

In this paper, we have argued that the men interviewed in many ways read their HIV diagnosis and illness in terms of their masculinity and its impact on it. Our analysis revealed that some men described having occupied the emphasized masculine position which we argue is a youthful masculinity, even though some older men may also occupy this position, that is characterized by pleasure seeking and having multiple concurrent sexual partners amongst other things (see also 29, 48). Most men described having occupied this position mainly before they were diagnosed with HIV, with most reporting having slightly departed from this position as they felt they could no longer occupy it after acquiring HIV. Reasons given for the departure from this position are consistent with Hunter's finding on *Isoka lamanyala* (a dirty and unacceptable masculinity) (30) which he described as a negative labeling given to Zulu men who had, according to the dominant culture of the time, ‘gone too far’ in their enactment of masculinity. Similarly, some men in our study felt that as they had HIV, having multiple concurrent sexual partners, one night stands, and not using condom during sex would attract harsh judgment from their peers and society, thus could no longer occupy this masculine position.

However, we found little or no evidence suggesting that these men cried over the loss of this youthful masculine position, rather they grieved the loss of access to a more valued form of masculinity, the traditional masculine position. The latter masculine position has cultural currency in many African settings. The men who occupy it are seen as exemplifying what manhood is. Thus they are celebrated and marveled at as they have demonstrated maturity and a transition from being a boy into being a responsible man (25, 48).

As such, this culturally informed manner of attaining manhood provides a cultural prescription for young boys and men to measure themselves and their masculinities against (49). To occupy this position, men should perform roles such as finding a stable job, marry and start a family, provide for and sustain it (41, 48). Indeed, most men interviewed in this study aspired to occupy this position, yet the majority of them regrettably felt it was no longer accessible to them as they now had acquired HIV. In general, men closely linked HIV acquisition to inadequacy and loss [of manhood]. This was more apparent in Itumeleng's and Jeff's narratives whom we argue had certainly tapped into some of the aspects of traditional masculinity, but they too viewed the traditional masculine position as unattainable, linking their lack of potency to attain it to their having HIV.

We have noted a sizeable number of men who reconfigured their masculinity after being diagnosed with HIV. We argue that this positional shift in masculinities was in reaction to having been diagnosed with HIV. Through performing more caring roles toward others (50), being concerned about their health and starting and adhering to treatment (48), expressing their needs and emotions and seeking help, these men had clearly departed from the harmful notions of hegemonic masculinity, as these behaviors are clearly in opposition to it (51). It appears that the observed shift was preceded by some critical reflection on the men's part. The possibilities of men changing their behaviors and masculinities have been alluded to by Barker and Ricardo (48). They argued that in Africa, there is evidence that masculinities are changing as ‘the dimensions of the AIDS epidemic in Africa and the devastation of families are forcing some men to question gender norms and attitudes that were once unquestionable’ (p. 44).

Similarly, in our study, the vast majority of men who were able to reconfigure their masculinities into one emphasizing responsibility and caring were likely to be those who had, over the years, mulled over their life circumstances and masculinities, disclosed their status, sought help thus had linkages to care, were on ARVs and adhering. This highlights the critical need to reach out to those men who are living with HIV but may not be sick and thus not eligible for ARVs and ensure that they are linked to support and care. Furthermore, we argue that the men who were unable to reconfigure their masculinities and had remained in the diminished position were particularly vulnerable to negative coping strategies (e.g. drinking alcohol at harmful levels, denial, and other self-destructive behavior) which ultimately were likely to be life shortening.

Interventions to support adjustment to living with HIV need to take into account the impact of HIV on masculinity and the impact of masculinity on coping with a life with HIV and its length. Available gender-transformative interventions in the gender-based violence (GBV) and HIV prevention sectors have sought to change the way men see themselves as men as part of the prevention of GBV and sexually risk behavior. The success of these interventions which are ‘grounded on research-based theoretical models of prevention’ (52) has been documented (53), and the Stepping Stones intervention is an example in South Africa (see 54, 55). The Stepping Stones curriculum comprises a participatory approach that includes critical reflection to encourage safer sexual practices through building more gender-equitable relationships (54). This approach draws on Freire's (56) work in which he argues that through discourse and reflection, people can begin to question themselves and their behavior and pursue alternative ways of being. In working with men for gender-transformation, this approach affords men spaces to reappraise their masculinities and adopt more gender-equitable forms of masculinities (47). Thus, we argue that the same principles of critical reflections could be used with good effect in interventions that seek to promote positive adjustment for men living with HIV in the critical phase after diagnosis and before care, as well as those starting care.

### Strengths and limitations

Most men interviewed in this study were recruited from a HIV clinic in Johannesburg and Soweto. Thus, because they had access to HIV services from the clinics, they may have been different to other men living with HIV in the general population. Notwithstanding, however, in our analysis, in terms of experiences of living with HIV and the behaviors reported, we could not discern any difference between these men (recruited through clinics) and those recruited in the general community in Mthatha.

In this study, we interviewed a man who self-identified as a man who has sex with both men and women. Whilst our analysis revealed no discernable differences in terms of his experiences of living with HIV and constructions of masculinities as compared to those who self-identified as heterosexual, future studies would do well not to mix men who have different sexual orientations as this may introduce gender identity complexities that may be difficult to explicate. We acknowledge that social desirability could be a potential bias in this study as some men may have reported behaviors they felt would be acceptable to be told to the interviewers.

## Conclusion

In this paper we have shown that HIV can have a significant effect on men and their masculinities. Furthermore, we have presented evidence that men's constructions and performance of harmful notions of hegemonic masculinity pre-HIV diagnosis negatively affect their help-seeking behavior and adjustment to living with HIV, post-diagnosis. Interventions with men living with HIV need to provide safe spaces for men to reflect on their life circumstances (including health risk behaviors), and identify significant, meaningful and future-oriented goals and aspirations. These could be the necessary motivations and triggers for men to reconfigure their masculinities and help-seeking behavior. For this change to occur and for men to adopt more gender and progressive masculinities that would enable them to accept, cope, and adjust more easily to living with HIV, the infusion of elements of critical reflection (56) in interventions may be beneficial. Such interventions should critically explore the constructions of social identities and the pressures these place on men. Furthermore, the interventions should vigorously challenge harmful constructions of masculinities, this process may render men amenable to change.

## References

[1] Rosen S, Larson B, Brennan A, Long L, Fox M, Mongwenyana C (2010). Economic outcomes of patients receiving antiretroviral therapy for HIV/AIDS in South Africa are sustained through three years on treatment. PLoS One.

[2] Bor J, Tanser F, Newell M-L, Barnighausen T (2012). Nearly full employment recovery among South African HIV patients on antiretroviral therapy: evidence from a large population cohort. Health Aff (Millwood).

[3] Bor J, Herbst AJ, Newell M-L, Barnighausen T (2013). Increases in adult life expectancy in rural South Africa: valuing the scale-up of HIV treatment. Science.

[4] Shafer LA, Nsubunga RN, White R, Mayanja BN, Chapman R, O'brien K (2011). Antiretroviral therapy and sexual behaviour in Uganda: a cohort study. AIDS.

[5] Kembabaziz A, Bunjunirwe F, Hunt PW, Martin JN, Muzoora C, Haberer JE (2013). Disinhibition in risky sexual behaviour in men, but not women, during four years of antiretroviral therapy in rural, Southwestern Uganda. PLoS One.

[6] Eisele TP, Mathews C, Chopra M, Brown L, Silvestre E, Daries V (2008). High levels of risk behaviour among people living with HIV initiating and waiting to start antiretroviral therapy in Cape Town South Africa. AIDS Behav.

[7] Department of Health Republic of South Africa (2013). The South African antiretroviral treatment guidelines.

[8] CDC (2003). Advancing HIV prevention: new strategies for a changing epidemic. Morbidity and mortality weekly report.

[9] Dageid W, Govender K, Gordon SF (2012). Masculinity and HIV disclosure among heterosexual South African men: implications for HIV/AIDS intervention. Cult Health Sex.

[10] Skovdal M, Campbell C, Madanhire C, Mupambiyeri Z, Nyamukapa C, Gregson S (2011). Masculinity as a barrier to men's use of HIV services in Zimbabwe. Global Health.

[11] Snow RC, Madalane M, Poulsen M (2010). Are men testing? Sex differentials in HIV testing in Mpumalanga Province, South Africa. AIDS Care.

[12] Wyrod R (2011). Maculinity and the persistence of AIDS stigma. Cult Health Sex.

[13] Siu GE, Wight D, Seeley J (2012). How a masculine work ethic and economic circumstances affect uptake of HIV treatment: experiences of men from an artisanal gold mining community in rural eastern Uganda. J Int AIDS Soc.

[14] Lynch I, Brouard PW, Visser MJ (2010). Constructions of masculinity among a group of South African men living with HIV/AIDS: reflections on resistance and change. Cult Health Sex.

[15] Connell RW (1987). Gender and power.

[16] Morrell R (1998). Of boys and men: masculinity and gender in Southern African studies. Masculinities S Afr.

[17] Morrell R, Jewkes R, Lindegger G (2012). Hegemonic masculinity/masculinities in South Africa: culture, power, and gender politics. Men Masculinities.

[18] Connell RW, Messerschmidt JW (2005). Hegemonic masculinity: rethinking the concept. Gender Soc.

[19] Connell RW (1993). The big picture: masculinities in recent world history. Theor Soc.

[20] Evans T, Wallace P (2008). A prison within a prison? The masculinity narratives of male prisoners. Men Masculinities.

[21] Courtenay WH (2000). Constructions of masculinity and their influence on men's well-being: a theory of gender and health. Soc Sci Med.

[22] Groes-Green C (2009). Hegemonic and subordinated masculinities: class, violence and sexual performance among young Mozambican Men. Nord J Afr Stud.

[23] Ratele K (2013). Subordinate black South African men without fear. Cahiers d’Études Afr.

[24] Jewkes R, Sikweyiya Y, Morrell R, Dunkle K (2011). Gender inequitable masculinity and sexual entitlement in rape perpetration South Africa: findings of a cross-sectional study. PLoS One.

[25] Jewkes R, Morrell R (2010). Gender and sexuality: emerging perspectives from the heterosexual epidemic in South Africa and implications for HIV risk and prevention. J Int AIDS Soc.

[26] Koenig-Visagie LH, van Eeden J (2013). Gendered representations of fatherhood in contemporary South African church imagery from three Afrikaans corporate churches. Verbum Ecclesia.

[27] Sorrell JBJ, Raffaelli M (2005). An exploratory study of constructions of masculinity, sexuality and HIV/AIDS in Namibia, Southern Africa. Cult Health Sex.

[28] Hunter M (2005). Cultural politics and masculinities: multiple-partners in historical perspective in KwaZulu-Natal. Cult Health Sex.

[29] Hunter M (2004). Masculinities and multiple-sexual-partners in KwaZulu-Natal: the making and unmaking of Isoka. Transformation.

[30] Hunter M (2010). Love in the time of AIDS: inequality, gender, and rights in South Africa.

[31] Goffman E (1963). Stigma: notes on the management of spoiled identity.

[32] Ouzgane L (2006). Islamic masculinities.

[33] Archer L (2001). ‘Muslim brothers, black lads, traditional Asians’: British Muslim young men's constructions of race religion and masculinity. Femin Psychol.

[34] Kimmel M (1996). Manhood in America: a cultural history.

[35] Dunk TW (1991). It's a working man's town: male working-class culture.

[36] Staples R (1982). Black masculinity: the black male's role in American society.

[37] Wood K, Jewkes R, Morrell R (2001). Dangerous’ love: reflections on violence among Xhosa township youth. Changing men in Southern Africa.

[38] Dahlgren L, Emmelin M, Winkvist M (2004). Qualitative methodology for international public health.

[39] Hennink M, Hutter I, Bailey A (2011). Qualitative research methods.

[40] Silverman D (2002). Interpreting qualitative data. Methods for analysing talk, text and interaction.

[41] Walker L (2005). Men behaving differently: South African men since 1994. Cult Health Sex.

[42] Lindegger G, Durrheim K, Stones C (2001). Men, HIV/AIDS and masculinity, in socio-political and psychological perspectives on South Africa.

[43] Gray J (2006). Becoming adherent: experiences of persons living with HIV/AIDS. J Assoc Nurs AIDS Care.

[44] Morrell R (2001). Changing men in Southern Africa.

[45] Stanton AL, Revenson TA, Tennen H (2007). Health psychology: psychological adjustment to chronic disease. Ann Rev Clin Psychol.

[46] Abrahams N, Jewkes R (2010). Barriers to post exposure prophylaxis (PEP) completion after rape: a South African qualitative study. Cult Health Sex.

[47] Reihling H (2013). Positive men: searching for relational dignity through health activism in a South African township. Soc Dyn.

[48] Barker G, Ricardo C (2005). Young men and the construction of masculinity in sub-Saharan Africa: implications for HIV/AIDS, conflict, and violence.

[49] Barker G (2005). Dying to be men: youth and masculinity and social exclusion.

[50] Morrell R, Jewkes R (2009). “I am a male, although I am a little bit soft”: men who care in South Africa. A case study of masculinities and gender equality.

[51] Barker G, Greene M, Nascimento M, Segundo M, Ricardo C, Taylor A (2012). Men who care: a multi-country qualitative study of men in non- traditional caregiving roles.

[52] Gevers A, Jama Shai N, Sikweyiya Y (2013). Gender-based violence and the need for evidence-based primary prevention in South Africa. Afr Saf Promot.

[53] Dworkin SL, Treves-Kagan S, Lippman SA (2013). Gender-transformative interventions to reduce HIV risks and violence with heterosexuallly-active men: a review of the global evidence. AIDS Behav.

[54] Jewkes R, Nduna M, Levin J, Jama N, Dunkle K, Khuzwayo N (2006). A cluster randomised controlled trial to determine the effectiveness of stepping stones in preventing HIV infections and promoting safer sexual behaviour amongst youth in rural Eastern Cape, South Africa: trials design, methods and baseline findings. Trop Med Int Health.

[55] Jewkes R, Wood K, Duvurry N (2010). “I woke up after I joined stepping stones”: meanings of an HIV behavioural intervention in rural South African young people's lives. Health Educ Res.

[56] Freire P (1973). Pedagogy of the oppressed.

# Sub article: Risk factors for unplanned and unwanted teenage pregnancies occurring over two years of follow-up among a cohort of young South African women

**DOI:** 10.3402/gha.v7.23719

**Published:** 2014-08-21

**Authors:** Nicola J. Christofides, Rachel K. Jewkes, Kristin L. Dunkle, Frances McCarty, Nwabisa Jama Shai, Mzikazi Nduna, Claire Sterk

**Affiliations:** 1 School of Public Health, Faculty of Health Sciences, University of the Witwatersrand, Johannesburg, South Africa; 2 Gender and Health Research Unit, Medical Research Council, Pretoria, South Africa; 3 Rollins School of Public Health, Behahioral Sciences and Health Education, Emory University, Atlanta, GA, USA; 4 Department of Psychology, University of the Witwatersrand, Johannesburg, South Africa

**Keywords:** unplanned pregnancy, unwanted pregnancy, adolescent pregnancy, gender-based violence, South Africa

## Abstract

**Background:**

Although teenage pregnancies in South Africa have declined, the short and longer term health and social consequences are a potential public health concern. This longitudinal study aimed to describe the range of risk and protective factors for incident unwanted and unplanned pregnancies occurring over 2 years of follow-up among a cohort of adolescent women in the Eastern Cape, South Africa. It also investigated the relationship between gender inequality and gender-based violence and subsequent unplanned and unwanted pregnancies among the cohort.

**Objective:**

Teenage girls, aged 15–18 years (*n*=19), who were volunteer participants in a cluster randomized controlled trial and who had data from at least one follow-up were included in this analysis. To assess risk and protective factors for incident unwanted or unplanned pregnancies, we constructed multivariate polytomous regression models adjusting for sampling clusters as latent variables. Covariates included age, having a pregnancy prior to baseline, education, time between interviews, study intervention arm, contraceptive use, experience of intimate partner violence, belief that the teenage girl and her boyfriend are mutual main partners, and socioeconomic status.

**Results:**

Overall, 174 pregnancies occurred over the 2-year follow-up period. Beliefs about relationship control were not associated with unwanted and unplanned pregnancies, nor were experiences of forced first sex or coerced sex under the age of 15. Hormonal contraception was protective against unplanned pregnancies (OR 0.40; 95% CI 0.21–0.79); however, using condoms was not protective. Physical abuse (OR 1.69; 95% CI 1.05–2.72) was a risk factor for, and having a pregnancy prior to baseline was protective against an unwanted pregnancy (OR 0.25; 95% CI 0.07–0.80). Higher socioeconomic status was protective for both unplanned and unwanted pregnancies (OR 0.69; 95% CI 0.58–0.83 and OR 0.78; 95% CI 0.64–0.96). Believing that the teenage girl and her boyfriend were mutual main partners doubled the odds of reporting both an unplanned and unwanted pregnancy (OR 2.58 95% CI 1.07–6.25, and OR 2.21 95% CI 1.13–4.29).

**Conclusion:**

Although some of the measures of gender inequity were not associated with unplanned and unwanted pregnancies, there is evidence of the role of both gender power and socioeconomic status. This was evident in teenage girls who experienced physical violence being more likely to have an unwanted pregnancy. Interventions to prevent teenage pregnancies need to be tailored by socioeconomic status because some teenagers may see having a pregnancy as a way to have a more secure future. Interventions that engage with relationship dynamics of teenagers are essential if unwanted and unplanned pregnancies are to be prevented.

Teenage pregnancies have adverse short- and long-term health outcomes for both the young women and their infants. Teenage mothers have been found to be at increased risk for anemia, urinary tract infection, and pregnancy-induced hypertension (1–4) . Infants were more likely to suffer infant and neonatal death, accidents, infections, and sudden infant death syndrome (SIDS) (5). There is also a body of literature that has explored the longer term social and mental health consequences of teenage pregnancy. These consequences include depression and substance use, increased sexual risk behavior, as well as lower educational attainment and socioeconomic status (6–8) . Most of this literature emanates from developed countries in North America, Australia, and Europe.

Nearly one-third of South African women report having had a teenage pregnancy (9, 10). There is evidence that teenage fertility declined by 10% between 1996 and 2001 from 79 births per 1,000 women to 65 births per 1,000 women (11).

Studies from South Africa have described the relationship between teen pregnancies and poorer educational outcomes (12, 13). A recent study of young women in South Africa found that early teenage pregnancies increased risk of acquiring HIV (14).

It is against this backdrop of both short and longer term adverse health outcomes that teenage pregnancies are largely viewed as a public health issue. However, few studies investigating the risk factors for teenage pregnancies differentiate between desired, unwanted, and unplanned pregnancies. In the South African context, some teenage pregnancies are desired, but most are unplanned or unwanted (11, 15). An unplanned pregnancy is described in the literature as a pregnancy that is not desired at that particular time, in other words it is mistimed, whereas an unwanted pregnancy is not wanted at all (16). The adverse consequences for unwanted pregnancies tend to be more severe than those for unplanned pregnancies (17). Literature that differentiates between unplanned and unwanted pregnancies has focused on women of all age groups and not on teenage girls in particular. Differentiating between unplanned and unwanted pregnancies may allow for a more nuanced understanding of the risk factors of teenage pregnancy, and may allow for the development of more closely targeted and more effective prevention strategies. In addition, increasing understanding of the difference between unplanned and unwanted pregnancy may assist health care providers in determining not only the unmet need for contraceptive services but also for abortion services.

Studies from South Africa have identified lower educational achievement and a shock to the household, defined as death of a household member, job loss, marital disruption, or loss of a grant or remittance, as a risk factors for teenagers becoming pregnant (12, 13). Other studies have found an association between gender inequality and gender-based violence, including child sexual abuse and forced first sex, and teenage pregnancies (15, 18–24) . However, this research has rarely differentiated on the bases of whether the teenage pregnancy was unplanned or unwanted.

This longitudinal study aimed to describe the range of risk and protective factors for incident unwanted and unplanned pregnancies occurring among a cohort of 819 teenage women aged 15–18 over approximately 2 years of follow-up. It also aimed to describe the relationship between gender inequality and gender-based violence and subsequent incident pregnancies among a cohort of teenage girls in the Eastern Cape, South Africa.

## Methods

### Study population and setting

In total, 1,416 South African women aged 15–26 were recruited from 70 villages and residential areas near Mthatha in the Eastern Cape, South Africa. The analysis presented here is based on a subset of 922 teenage women aged 15–18 years at baseline.

### Study design

Data for the analysis was from a cluster randomized controlled trial of the Stepping Stones HIV prevention intervention (25, 26). The study was a stratified, two-stage survey with villages sampled from predefined strata based on geographical characteristics and participants clustered within villages. Villages were eligible for inclusion if they were in the study area, located 10 km or more from other study villages, contained a secondary school, and were willing to participate. Once village gatekeepers agreed to inclusion, volunteers were recruited, primarily from secondary schools. Twenty women were recruited per cluster. All participants were resident in the village where they were schooling and mature enough to understand the content of the intervention (25). For the purposes of these analyses, the women were treated as an observational cohort, and exposure to the Stepping Stones intervention was treated as a covariate.

### Questionnaire

Detailed discussion of the development and content of the questionnaire appears elsewhere (25). Data used here include sociodemographic characteristics, sexual behavior, and a range of reproductive outcomes including pregnancy. Women who responded in the affirmative to the question ‘Since the previous interview have you been pregnant?’ or to the question ‘Are you currently pregnant?’ at either follow-up interview were categorized as having an incident pregnancy. An additional question asked when the respondent became pregnant. To eliminate any misclassification of the timing of pregnancies, the reported dates when a woman became pregnant and her baseline interview dates were compared to ensure that the pregnancy started after the baseline interview. Assessment of pregnancy desire was based on the response to the item: ‘At the time you became pregnant did you want to become pregnant then, did you want to wait until later, or did you not want to have any children at all?’ Women who responded that they ‘wanted to wait until later’ were classified as having an unplanned pregnancy whereas those who said that they ‘did not want to have children at all’ were classified as having an unwanted pregnancy. Teenage girls who said that they wanted the pregnancy at that time were dropped from further analysis (*n*=10). Many of the teenage girls had a pregnancy prior to the baseline assessment. For the analysis of the association between child sexual abuse and first pregnancies, all first pregnancies were included in the analysis, including those prior to baseline and those that occurred over the 2 years of follow-up.

The questionnaire included the WHO violence against women instrument which was modified to be culturally appropriate (27). The instrument included five items measuring single and multiple occurrences of physical abuse occurring within the past 12 months and over a woman's lifetime, and four items measuring single and multiple occurrences of sexual abuse within the past 12 months and over a woman's lifetime. Three variables measuring intimate partner violence (IPV) were derived. These included a 3-level categorical variable of type of abuse which included no abuse, physical only, and sexual abuse with or without physical abuse. The frequency of IPV was measured through a categorical variable with no abuse, once only, and more than once. The temporality of IPV was measured using a 4-level variable: no abuse, abuse occurring before the previous 12 months only, abuse occurring in the past 12 months only, and abuse occurring both before and during the past 12 months.

Coerced first sex was derived from an item that asked ‘Which of the following statements most closely describes your experiences the first time you had sexual intercourse? ‘I was willing; I was persuaded; I was tricked; I was forced; I was raped’. This was dichotomized into those who were willing and those who were coerced.

Child sexual abuse was measured through a response to one or more of four items that asked about the period ‘before the age of 18,’ for example ‘Someone touched my thighs, buttocks, breasts, or genitals when I did not want him to or made me touch his private parts when I did not want to’ (28).

For women with current main male partners at baseline, a sexual relationship power scale developed by Pulerwitz and her colleagues (10-items, Cronbach's alpha =0.73), previously shown to be associated with prevalent HIV among South African women, was used to measure gender power equity (29). A typical item was, ‘When (Name of boyfriend) wants me to sleep over, he expects me to agree’. Each item was assessed on a 4-point Likert scale and the measure was scored and categorized into tertiles. For the analyses, the tertile with lowest equity was compared to the middle and higher ones.

Contraceptive use was measured by an item that asked ‘Are you currently doing something or using any method to delay or avoid getting pregnant?’ and follow-up questions which asked about which contraceptive method was being used. Contraceptive use was then categorized into a 3-level variable: no contraceptive use, hormonal contraceptives, or condoms only. Contraceptive knowledge was measured using a 6-item scale, with items such as: ‘A woman who is not using contraception and has sex during her period will probably get pregnant’. The continuous variable was later dichotomized into higher and lower knowledge.

Three questions established past year numbers of main boyfriends, *khwapheni* (hidden partners concurrent with main partners), and men with whom the participant had sex only once. Socioeconomic status was assessed by use of a scale that encompassed household goods ownership, food, and cash scarcity.

### Data collection

Face-to-face interviews by trained, female interviewers using standardized questionnaires were carried out at 12-month intervals over approximately 2 years of follow-up. Detailed data were collected from all participants on reproductive outcomes and sexual behavior at each of three time points: baseline (T_0_), first follow-up (T_1_) which occurred between 1 year and 18 months after baseline, and second follow-up (T_2_) which occurred between 1 year and 18 months after the first follow-up data collection. The amount of time participants were followed up is controlled for in analyses by creating a variable of time since baseline data collection. The date of the baseline interview was subtracted from the final interview.

### Data analysis

Data analysis was carried out in Stata 10 (Stata Corp., College Station, TX, USA). Descriptive statistics were first calculated for all variables. Potential risk and protective factors and incident unwanted or unplanned pregnancies were explored. Two-way associations were determined between measures of gender power relations including relationship control and IPV variables, sexual risk behavior such as number of sexual partners, and demographic variables. For continuous variables the summary took the form of means with 95% confidence intervals (95% CIs), whereas for binary variables the summary took the form of percentages with 95% CIs. Variables were considered statistically significant if *p*<0.05.

To assess risk and protective factors for incident unwanted or unplanned pregnancies, we constructed multivariate polytomous regression models using svy: mlogit, which adjusts for clusters as latent variables within the model. Variables were considered for inclusion in the model if the significance in two-way analyses was less than 0.2 or if they were theoretically important.

Explanatory variables included age at baseline, having a pregnancy prior to baseline, education, time between interviews, study intervention arm, mutual main partners, contraceptive use, experience of IPV, and socioeconomic status. Elimination was used in the modeling to obtain the most parsimonious model. Variables that had a non-significant *p*-value were systematically eliminated with the least significant variable eliminated first. All IPV variables and relationship control were included in the original model and systematically eliminated, with the exception of experience of IPV by type of violence. All theoretically relevant interaction terms were tested and none were found to be statistically significant.

### Ethical considerations

Ethical clearance for the study was granted by the University of Pretoria ethics committee. Written consent was obtained for all participants recruited into the study.

## Results

Eight hundred and nineteen study participants provided data at baseline and at least one follow-up time point. These participants reported a total of 174 pregnancies over approximately 2 years of follow-up. Of these pregnancies, 10 (3.6%) were wanted at that time, and these respondents were dropped from subsequent analyses. Of the remaining 164 pregnancies, 53 (32.3%) were unplanned and 85 (67.7%) were unwanted. Pregnancy intention data were missing for 22 young women, and they were also dropped from analyses, for a total of 756 women reporting 136 pregnancies.

Table 1 shows the sociodemographic and select sexual and reproductive behaviors for three groups: women who reported no incident pregnancy, and those who had at least one unplanned or unwanted pregnancy at T1 or T2. Those women who reported an unwanted or unplanned pregnancy came from households with lower socioeconomic status. Young women who had incident unplanned or unwanted pregnancies were less likely to have had a pregnancy prior to baseline. They were also less likely to report hormonal contraceptive use at baseline and more likely to report using only condoms as contraceptives. They were more likely to believe that they were their boyfriend's main partner, and have a relationship of shorter duration.

**Table 1:** Sociodemographic and behavioral characteristics of teenage girls (15–18) by whether they reported no pregnancy or incident unplanned or unwanted over approximately 2 years of follow-up

Demographic variables	No incident pregnancy (*n*=620)			Incident unplanned pregnancies (*n*=51)			Incident unwanted pregnancies (*n*=85)			
			
	*n*	%	95% CI	*n*	%	95% CI	*n*	%	95% CI	*p*
Age at baseline										0.67
15 years	23	3.65	2.3–5.7	1	1.85	0.3–12.5	4	4.55	1.7–11.7	
16 years	148	23.49	20.4–26.8	12	22.22	12.0–37.4.5	17	19.32	13.1–27.5	
17 years	251	39.84	35.5–44.4	27	50.0	34.7–65.3	42	47.73	35.7–60.0	
18 years	208	33.02	28.9–37.4	14	25.22	15.0–40.9	25	28.41	17.7–42.3	
Boyfriend in past 12 months	609	96.67	95.1–97.8	52	96.3	86.1–99.1	87	98.9	92.2–99.8	0.43
Mutual main partners	458	72.70	68.4–76.6	46	85.19	71.8–92.9	71	80.7	68.1–89.1	0.11
10 or more years of education	276	43.81	36.7–51.2	21	38.89	26.4–53.0	35	40.23	27.4–54.6	0.69
Mean socioeconomic status score	629	0.11	−3.1–3.8	54	–0.47	−3.1–2.3	88	–0.14	−3.1–3.8	<0.01
Mean duration of main relationship	615	23 mo	0–148	53	21 mo	0–68	85	19 mo	0–74	0.04
Mean no of days since last sex	545	107 days	0–1170	51	114 days	1–2100	82	77 days	1–870	0.16
Pregnancy prior to baseline	83	14.72	11.6–18.6	7	13.46	6.3–26.6	3	3.61	1.2–10.5	0.02
Contraception										0.02
None	221	39.89	35.1–44.9	29	55.77	43.2–67.7	34	40.96	30.9–51.0	
Hormonal	245	43.44	38.5–48.6	14	26.92	15.9–41.8	27	32.53	23.2–43.0	
Condoms only	94	16.67	13.3–20.7	9	17.31	9.0–30.6	22	26.51	19.7–34.0	
Contraceptive knowledge										0.64
Low	262	41.72	38.1–45.5	26	48.15	33.2–63.5	39	42.47	34.9–54.0	
High	366	58.28	54.5–62.0	28	51.85	36.5–66.8	49	55.68	45.8–65.0	
Risky behavior										
Number of sexual partners at baseline										0.86
0	29	5.32	3.6–8.0	3	5.88	2.0–16.2	3	3.66	1.2–10.6	
1	340	62.39	58.0–66.6	30	58.82	42.9–73.1	52	63.41	52.8–72.9	
2 or more	176	32.29	28.5–36.4	18	35.29	22.1–51.3	27	32.93	23.2–44.3	

### Loss to follow-up

Table 2 shows that of the (*n*=922) women aged 15–18 years, 11.4% (*n*=103) were lost to follow-up. These women were older and were less likely to have completed 10 years of schooling than those who were successfully followed; those lost to follow-up were also more likely to have been sexually active at baseline. The mean age of the teenage girls retained in the cohort was 17.04 years (range 15–18 years). At baseline, 87.3% of participants had had sexual intercourse. By the end of the follow-up period, 93.6% of the young women had had sexual intercourse.

**Table 2:** Comparison of the study participants followed up and lost to follow-up

	Followed up (%) (*n*=819)	Lost to follow-up (%) (*n*=103)	*p*
Age (Mean years)	17.0	17.3	<0.01
Education:>grade 10	43.5%	30.1%	<0.01
Socioeconomic status (score)	0.05	0.03	0.90
Ever had a boyfriend	96.1	98.1	0.32
Ever had sex	87.3	94.2	0.05
Pregnancy prior to baseline	13.7	12.5	0.73
Mutual main partner	76.6	83.2	0.10
Duration of sexual activity (years)	2.24	2.24	0.99
Alcohol problem	3.5	6.8	0.14
Lifetime Number of sexual partners			0.64
≤1	67.5	71.9	
2–5	29.9	25.0	
>5	2.7	3.1	
Contraceptive use – ever	68.3	69.4	0.88
Age difference with partner (years)	3.11	2.77	0.15
Experience of IPV ever by type			
None	68.07	60.0	0.14
Physical abuse only	20.69	27.0	
Physical and/or sexual abuse	11.24	13.0	
Reproductive health knowledge score	23.0	22.4	0.14
Intervention: Stepping Stones	53.0	56.3	0.57

### Relationship between unwanted and unplanned pregnancies and gender-based violence and power and control in relationships

Table 3 shows the associations between experiences of gender-based violence and power and control in relationships and incident unwanted and unplanned pregnancies. Young women who had an incident unplanned and unwanted pregnancy were more likely to report experiences of physical abuse at baseline than those women who did not have an incident pregnancy. However, they were less likely to report experiences of sexual abuse. There was no association between power and control in relationship at baseline and subsequent incident unplanned or unwanted pregnancies. Having a non-consensual first sexual experience was also not associated with having a teenage pregnancy. Coerced sex under the age of 15 years was not associated with incident pregnancies. Child sexual abuse was associated with first pregnancies (some occurred prior to baseline), but not with subsequent pregnancies (44.1% vs. 31.9%; *p*=0.002) (data not shown in table).

**Table 3:** Associations between gender-based violence and control and incident pregnancies over approximately 2 years of follow-up

	No incident pregnancy (*n*=620)			Incident unplanned pregnancies (*n*=51)			Incident unwanted pregnancy (*n*=85)			
			
	*n*	%	95% CI	*n*	%	95% CI	*n*	%	95% CI	*p*
Lifetime experience of IPV by a boyfriend by type										0.06
None	437	69.81	65.9–73.5	38	70.37	58.2–80.2	57	64.77	54.9–73.5	
Physical abuse only	116	18.53	15.2–22.4	11	20.37	11.5–33.4	26	29.55	21.2–39.5	
Sexual abuse with/without physical	73	11.66	9.1–14.8	5	9.26	4.2–19.3	5	5.68	2.5–12.4	
Temporality of experience of IPV by a boyfriend										0.82
None	372	62.52	58.1–66.7	32	62.75	49.0–74.7	50	58.82	49.0–68.0	
Before the past 12 months only	37	6.22	4.4–8.7	3	5.88	1.9–16.8	4	4.71	1.8–12.0	
Within the past 12 months only	117	19.66	16.4–23.4	9	17.65	9.0–31.6	16	18.82	11.6–29.1	
Both before and within the past 12 months	69	11.60	9.2–14.6	7	13.73	6.7–26.1	15	17.65	11.7–25.8	
Experience of IPV by a boyfriend by frequency										0.16
None	407	64.60	60.5–68.5	35	64.81	52.3–75.6	53	60.23	50.4–69.3	
Once only	108	17.14	14.2–20.6	4	7.41	2.7–18.9	18	20.45	13.3–30.1	
More than once	115	18.25	15.3–21.7	15	27.78	17.7–40.8	17	19.32	13.3–30.1	
Relationship control scale										0.90
Low control (more equal)	189	29.0	25.4–35.0	15	27.78	17.2–41.5	29	32.95	22.8–45.0	
Medium control	315	50.0	45.7–54.4	26	48.15	33.7–62.9	44	50.0	38.7–61.3	
High control (less equality)	126	20.0	16.6–24.0	13	24.07	12.7–40.8	15	17.05	16.7–23.7	
Forced first sex (*p*=0.21)	85	13.49	10.9–16.7	5	9.26	4.3–18.9	8	9.09	4.1–18.9	0.94
Coerced sex before 15 (*p*=0.61)	20	3.67	2.4–5.6	3	5.88	2.0–16.3	4	4.88	2.0–11.2	0.64

### Multivariable analysis results

Table 4 shows the results from the polytomous regression on unwanted and unplanned pregnancies. Hormonal contraception was significantly protective against unplanned pregnancies; however, it showed no impact on unwanted pregnancies. Using condoms as contraceptives was not associated with unplanned pregnancies. Physical abuse was a risk factor for unwanted pregnancies, but not unplanned pregnancies. Lower socioeconomic status was a risk factor for both unplanned and unwanted pregnancies. Believing that the teenage girl and her boyfriend were mutual main partners doubled the odds of reporting both an unplanned and unwanted pregnancy. Having a pregnancy prior to baseline was protective against an unwanted pregnancy; however, this was not the case for unplanned pregnancies.

**Table 4:** Polytomous regression model of risk factors for incident unplanned and unwanted teenage pregnancies, adjusted for time between interviews and treatment arm

	OR	95% Confidence Interval		*p*
Incident unplanned pregnancies				
Pregnancy prior to baseline	1.01	0.39	2.64	0.98
Age	0.90	0.60	1.35	0.61
Relationship duration	1.00	0.98	1.01	0.58
Contraception – none	Ref.			
Hormonal	0.40	0.21	0.79	<0.01
Condoms only	0.86	0.38	1.99	0.73
Mutual main partners	2.58	1.07	6.25	0.03
Socioeconomic status	0.69	0.58	0.83	<0.01
Exposure to intervention	0.96	0.50	1.85	0.89
IPV – none	Ref.			
Physical abuse only	1.06	0.51	2.21	0.88
Sexual abuse with/without physical abuse	0.74	0.30	1.81	0.51
Incident unwanted pregnancies				
Pregnancy prior to baseline	0.25	0.07	0.80	0.02
Age	1.09	0.82	1.46	0.54
Relationship duration	0.98	0.97	1.00	0.06
Contraception – none	Ref.			
Hormonal	0.74	0.43	1.30	0.29
Condoms only	1.81	0.99	3.30	0.05
Mutual main partners	2.21	1.13	4.29	0.02
Socioeconomic status	0.78	0.64	0.96	0.02
Exposure to intervention	1.43	0.85	2.41	0.18
IPV – none	Ref.			
Physical abuse only	1.69	1.05	2.72	0.03
Sexual abuse with or without physical abuse	0.55	0.24	1.27	0.16

## Discussion

The study found that lower socioeconomic status and a teenage girl believing that she was her boyfriend's main partner were risk factors for both incident unwanted and unplanned pregnancies. Having had a previous pregnancy and using hormonal contraceptives were protective against unplanned but not unwanted pregnancies. Women who had experienced physical abuse were more likely to have an unwanted pregnancy.

Unlike other studies, contraceptive knowledge and educational attainment were not associated with incident pregnancies over the 2 years of follow-up (12, 13). Our study suggests that the relationship dynamics and access to health services may play a greater role in teenage pregnancies than knowledge and education, although it may also be the case that because participants were recruited from schools, their educational attainment was too homogenous to observe an effect.

This study explored multiple potential connections between the experience of violence and abuse and teenage pregnancy. Unlike some previous studies, the results of the current study do not support a link between forced first sex and teenage pregnancies (15, 21); however, child sexual abuse was associated with first pregnancies (although not with second or third pregnancies). Our findings thus support a link between experience of early trauma and early pregnancy, but suggest that cumulative trauma is more important than a single sentinel event. Similarly, although we did not observe an association between power and control in intimate relationships and incident pregnancies, we did show that young women who experienced physical abuse were more likely to report a new unwanted pregnancy. A parallel ethnographic study conducted among young women from the study cohort show that women reported feeling little-to-no ability to control their sexual or reproductive lives (30). Although the findings on violence and unwanted teenage pregnancy reported here reflect multiple nuanced links at the level of individual analysis, it is likely that the pervasive context of women's disempowerment, created in part through the common occurrence of violence, also contributes to unwanted pregnancy.

Lower socioeconomic status was a risk factor for both unplanned and unwanted pregnancies. Teenage girls who come from poorer families may perceive pregnancy as a way to ensure greater security for their future, if the paternity of the child is established and the father of the child's family provides economic support (31, 32). By contrast, teenage girls who come from families who are better off may have a greater fear of becoming pregnant and disappointing their families, negatively having an impact on their educational and social status (31). However, not all teenage pregnancies are stigmatized; there are families which embrace teenage pregnancies and view it as a rite of passage to womanhood (33).

Young women's beliefs about the nature of their relationship increased the likelihood of having an incident pregnancy over the follow-up period. In particular, teenage girls who believed that they and their boyfriends were mutual main partners were more likely to report both unwanted and unplanned pregnancies. The perceived stability of the relationship could lead teenage girls to feel less concerned about the possibility of becoming pregnant. This may lead to inconsistent contraceptive use. In addition, the association with physical abuse from a male partner, combined with believing themselves to be in a committed relationship could result in young women acquiescing to pressure to take risks and not use contraceptives (especially condoms) consistently (32).

Use of hormonal contraceptives was protective against unplanned pregnancies, whereas using only condoms was a risk factor for an unwanted pregnancy. This could be because of condom breakage or lack of commitment to consistent and correct use (34). We also found that having a prior pregnancy was protective against a future unwanted pregnancy. This is additional evidence of a fertility trend in South Africa reported by Garenne and his colleagues (35) who found that women who give birth as teenagers wait several years before having another child. Having a pregnancy also enables and encourages young women to interact with health care services. In South Africa, more than 90% of women attend antenatal care services at least once, and most women deliver in hospitals or maternal and obstetric units. This provides an opportunity for women to access contraceptive services after delivery.

There is an unmet contraceptive need among the young women in the Eastern Cape, evident by the high percentage of teenage girls having unplanned and unwanted pregnancies. Although these services are provided free of charge at primary health care clinics in South Africa, there are problems with access, especially for young women. Health care providers are often judgmental about teenagers seeking contraceptive services (31, 36). In addition, we found that the mean time since last sex was 3 months, indicating that many of the young women are having sex infrequently. This may affect the consistency of contraceptive use, especially the commonly used injectable contraceptives (34). The two injectables used in primary health care clinics are depot medroxyprogesterone acetate (DMPA) and norethisterone enantate (NET-EN). Both are progestin-only injectables with DMPA providing 12 weeks’ protection and NET-EN 8 weeks’ protection, requiring repeat visits to clinics (34). Because many of the young women in the study were having sexual intercourse less frequently, they may not have attended the clinic for their follow-up visit and the next dose of the injectable contraceptive. This finding suggests the need for a wider range of contraceptive methods and wider promotion of emergency contraceptives.

Our study has several limitations. Pregnancies were self-reported and it is possible that women did not disclose pregnancies that resulted in a termination. This may have resulted in under-reporting of pregnancies. Subsequent longitudinal studies could explicitly measure induced abortions and miscarriages. Participants were volunteers, and this may limit the generalizability of the findings. Teenage girls who were lost to follow- up were older, with lower educational attainment, and the effect of this differential loss on the outcomes investigated in our study is unknown. However, the key strength of the study is the longitudinal design with incident pregnancies occurring after the risk and protective factors under study. In addition, the study differentiates between the risk and protective factors for unwanted and unplanned pregnancies among adolescent women. This allows for a more nuanced response.

## Conclusion

Although not all of the measures of gender inequity were associated with unplanned and unwanted pregnancies in this study, there is evidence that inequitable gender power relations and low socioeconomic status do increase risk. Interventions to prevent teenage pregnancies need to be tailored by socioeconomic status because some teenagers may see having a pregnancy as a way to have a more secure future. Interventions that engage with teenagers’ relationship dynamics are essential for effective prevention of unwanted and unplanned pregnancies. There is also an unmet contraceptive need among the young women in the Eastern Cape.

## References

[1] Fraser AM, Brockert JE, Ward RH (1995). Association of young maternal age with adverse reproductive outcomes. New Engl J Med.

[2] Lewis LN, Hickey M, Doherty DA, Skinner SR (2009). How do pregnancy outcomes differ in teenage mothers? A Western Australian study. Med J Aust.

[3] Magadi M (2006). Poor pregnancy outcomes among adolescents in South Nyanza region of Kenya. Afr J Reprod Health.

[4] Ventura SJ, Abma JC, Mosher WD, Henshaw SK (2008). Estimated pregnancy rates by outcome for the United States, 1990–2004. Natl Vital Stat Rep.

[5] Chen XK, Wen SW, Fleming N, Demissie K, Rhoads GG, Walker M (2007). Teenage pregnancy and adverse birth outcomes: a large population based retrospective cohort study. Int J Epidemiol.

[6] De Genna NM, Cornelius MD, Donovan JE (2009). Risk factors for young adult substance use among women who were teenage mothers. Addict Behav.

[7] Moore DR, Florsheim P, Butner J (2007). Interpersonal behavior, psychopathology, and relationship outcomes among adolescent mothers and their partners. J Clin Child Adolesc Psychol.

[8] Ramos-Marcuse F, Oberlander SE, Papas MA, McNary SW, Hurley KM, Black MM (2010). Stability of maternal depressive symptoms among urban, low-income, African American adolescent mothers. J Affect Disord.

[9] Department of Health The 1998 South African Demographic and Health Survey.

[10] Department of Health (2008). The 2003 South African Demographic and Health Survey.

[11] Moultrie TA, McGrath N, Moultrie TA, McGrath N (2007). Teenage fertility rates falling in South Africa. S Afr Med J.

[12] Grant MJ, Hallman KK (2008). Pregnancy-related school dropout and prior school performance in KwaZulu-Natal, South Africa. Stud Fam Plann.

[13] Marteleto L, Lam D, Ranchhod V (2008). Sexual behavior, pregnancy, and schooling among young people in urban South Africa. Stud Fam Plann.

[14] Christofides NJ, Jewkes RK, Dunkle KL, Nduna M, Shai NJ, Sterk C (2014). Early adolescent pregnancy increases risk of incident HIV infection in the Eastern Cape, South Africa: a longitudinal study. J Int AIDS Soc.

[15] Jewkes R, Vundule C, Maforah F, Jordaan E (2001). Relationship dynamics and teenage pregnancy in South Africa. Soc Sci Med.

[16] Henshaw SK (1998). Unintended pregnancy in the United States. Fam Plann Perspect.

[17] D'Angelo DV, Gilbert BC, Rochat RW, Santelli JS, Herold JM (2004). Differences between mistimed and unwanted pregnancies among women who have live births. Perspect Sex Reprod Health.

[18] Butler JR, Burton LM (1990). Rethinking teenage childbearing: Is sexual abuse a missing link?. Fam Relat.

[19] Fiscella K, Kitzman HJ, Cole RE, Sidora KJ, Olds D (1998). Does child abuse predict adolescent pregnancy?. Pediatrics.

[20] Kellogg ND, Hoffman TJ (1999). Early sexual experiences among pregnant and parenting adolescents. Adolescence.

[21] Koenig MA, Zablotska I, Lutalo T, Nalugoda F, Wagman J, Grey R (2004). Coerced first intercourse and reproductive health among adolescent women in Rakai, Uganda. Int Fam Plann Perspect.

[22] Roberts R, O'Connor T, Dunn J, Golding J, Team AS (2004). The effects of child sexual abuse in later family life; mental health, parenting and adjustment of offspring. Child Abuse Negl.

[23] Roosa MW, Tein J-Y, Reinholtz C, Angelini PJ (1997). The relationship of childhood sexual abuse to teenage pregnancy. J Marriage Fam.

[24] Vundule C, Maforah F, Jewkes R, Jordaan E (2001). Risk factors for teenage pregnancy among sexually active black adolescents in Cape Town. A case control study. S Afr Med J.

[25] Jewkes R, Nduna M, Levin J, Jama N, Dunkle K, Khuzwayo N (2006). A cluster randomized-controlled trial to determine the effectiveness of Stepping Stones in preventing HIV infections and promoting safer sexual behaviour amongst youth in the rural Eastern Cape, South Africa: trial design, methods and baseline findings. Trop Med Int Health.

[26] Jewkes R, Nduna M, Levin J, Jama N, Dunkle K, Puren A (2008). Impact of Stepping Stones on incidence of HIV and HSV-2 and sexual behaviour in rural South Africa: cluster randomised controlled trial. BMJ.

[27] World Health Organization (2000). WHO multi-country study on women's health and domestic violence: core questionnaire and WHO instrument – version 9.

[28] Bernstein DP, Fink L, Handelsman L, Foote J, Lovejoy M, Wenzel K (1994). Initial reliability and validity of a new retrospective measure of child abuse and neglect. Am J Psychiatr.

[29] Pulerwitz J, Gortmaker SL, DeJong W (2000). Measuring sexual relationship power in HIV/STD research. Sex Roles.

[30] Jewkes R, Wood K, Duvvury N (2010). ‘I woke up after I joined Stepping Stones’: meanings of an HIV behavioural intervention in rural South African young people's lives. Health Educ Res.

[31] Kaufman CE, de Wet T, Stadler J (2001). Adolescent pregnancy and parenthood in South Africa. Stud Fam Plann.

[32] Wood K, Jewkes R (2006). Blood blockages and scolding nurses: barriers to adolescent contraceptive use in South Africa. Reprod Health Matters.

[33] Jewkes R, Morrell R, Christofides N (2009). Empowering teenagers to prevent pregnancy: lessons from South Africa. Cult Health Sex.

[34] Shai NJ, Jewkes R, Nduna M, Dunkle K (2012). Masculinities and condom use patterns among young rural South Africa men: a cross-sectional baseline survey. BMC Publ Health.

[35] Garenne M, Tollman S, Kahn K (2000). Premarital fertility in rural South Africa: a challenge to existing population policy. Stud Fam Plann.

[36] Ehlers VJ (2003). Adolescent mothers’ utilization of contraceptive services in South Africa. Int Nurs Rev.

# Sub article: Whose problem is it anyway? Crimes against women in India

**DOI:** 10.3402/gha.v7.23718

**Published:** 2014-07-21

**Authors:** B. L. Himabindu, Radhika Arora, N. S. Prashanth

**Affiliations:** 1 Institute of Public Health, Bangalore, India; 2 Institute of Tropical Medicine, Antwerp, Belgium

**Keywords:** Delhi gang rape, women in India, child sex ratio, gender inequality

## Abstract

The recent public outcry following a brutal gang rape of a young woman in India's national capital was a watershed moment in the world's largest democracy. It generated widespread public and political support for strengthening legal provisions to punish sex offenders. Although the legal response is a useful deterrent against such heinous crimes, women continue to suffer due to deeply rooted social prejudices that make them vulnerable to violence and discrimination in society. In this commentary, we aim to analyse the current developments with respect to gender violence in India within a background of the social position of women in Indian society. Using secondary data related to sex-selective abortions and crimes against women, and a critical review of the portrayal of women in Indian cinema, we reflect on the role of health workers, researchers and public health professionals in shaping a social response towards improving gender parity in our country.

On 13 September 2013, a New Delhi judge sentenced four men to death for the brutal gang rape of a 23-year-old physiotherapy student. She died due to severe injuries suffered during the attack. The barbaric nature of the crime appalled the country and brought worldwide attention to what print media now calls the *Rape crisis* of India (1, 2). Nationwide protests forced lawmakers to refer this case to a fast-track court, and the judgment was pronounced in less than a year. The perpetrators were sentenced to the gallows, a punishment reserved in Indian law for the *rarest of the rare* instances of inhuman crime. The government sought to appease the widespread street protests in many cities. The law concerning violence against women was amended by the Parliament (3). The maximum punishment for rape resulting in death (or vegetative state) of the victim was modified from life imprisonment to include death penalty. Other laws related to sexual crime were made stricter, in the hope that this would deter people from committing such crimes. The source and subsequent impact of these immediate reactions and quick fixes have been extensively debated in the print media (4, 5).

In this commentary, we aim to discuss the current scenario of violent crimes against women in India within a backdrop of the social position of women in Indian society in general and the media response following this incident in particular.

## Crimes against women in India

The numbers of violent crimes in India especially those against women including rape that are reported in official statistics are increasing with each passing year (6). This violence thrives within a milieu of steady economic growth, and increasing inequality between the rich and poor in Indian society; India's GINI coefficient^1^ that has increased from 0.32 to 0.38 in the last two decades is evidence to that (7). India's new riches and development strides as witnessed by its GDP growth from $450.42 billion in 2000 to $1841.7 billion seem to bear no fruits for its women. In 2012, the crimes against women reported by official statistics increased by 24.7%, compared to those reported in 2008 (8). Ranging from the so-called *eve teasing*^2^ and outright sexual harassment on the street or workplace, to harassment for *dowry*,^3^ molestation in public transport vehicles, and the often-reported rape, these crimes against women reflect the vulnerability and deep-rooted problems related to the position of women in Indian society. Out of 28 states, 10 states reported more than 10,000 cases of crime against women in 2011 putting states with both high and low HDI (Human Development Index) and literacy rates in the list; probably an indication that education and economic growth alone do not influence the occurrence of these crimes and pointing towards socio-political and cultural factors. This can be further observed in the National Crime Records Bureau (NRCB) statistics which show that cruelty by a husband or his relatives (46.8%) and *dowry*-related crimes (7.1%) account for more than half of the crimes against women. With increased incidence and visibility of these gruesome crimes, there is an urgent need to address this problem at multiple levels of Indian society, including professional, familial and social settings.

According to the NCRB, 24,923 cases of rape were reported in 2012 (9), amounting to one rape every 22 minutes. A continuous increase in the reported cases of rape has been observed in the period from 2009 to 2012 with more than 3% increase in the number of cases reported in 2012 over 2011. Nearly, 12.5% (3,125) of the total victims of rape were girls younger than 14 years, 23.9% (5,957) were in the 14–18 age group, 50.2% (12,511) were in the 18–30 age group and 12.8% (3,187 victims) were in the 30–50 age group. These statistics possibly do not capture the actual numbers. While gross under-reporting could be one reason for this (10), the other reason is that crimes such as gang rapes, stalking and acid attacks on women were not included in official statistics of crime against women until the law was amended on 3 February 2013 (3). Even amongst those crimes, the NCRB statistics takes only the principal offence of the formal complaint (First Information Report) into account. So in cases such as the Delhi gang rape, which resulted in the death of the rape victim, the rape would be unaccounted for in the official statistics; the true scale of gender violence thus remaining undercounted.

The problem of underestimation of the gender-based crime is compounded by failure of the justice system of the country in securing convictions. The NCRB statistics (9) show that 54.6% of rape cases reported in 2011 are yet to be investigated, while 30.6% are waiting for trial. Only 16% of the cases have resulted in convictions. The recent protests demanded for stricter laws, possibly under an assumption that greater punishment will reduce the rate of sexual crime. However, with the abysmal conviction rate, stricter laws alone may not achieve the necessary deterrence.

The amendments made to the criminal law (3) are not (yet) comprehensive. Marital rape, for example, is still not considered a criminal offence. Rape by armed personnel (military and police), although under the purview of the law, is excluded if it occurs in several states of India (north-eastern states, Jharkhand, and Jammu and Kashmir) where the draconian Armed Forces Special Powers Act (AFSPA) deprives women from seeking legal recourse in such circumstances. The consequences of having separate law for Armed forces are many. For example, the alleged mass rape of 53 women in 1991 and the alleged rape and subsequent killing of Manorama Devi in the year 2000, both incidents involved armed forces personnel. Both cases are still in court and the verdict is yet to be delivered, decades after the crimes (11, 12).

On one hand is the political apathy in formulating and implementing gender-sensitive policies. On the other is the lack of a clear protocol of action in these issues, care of victims of rape being one such example. Although a detailed directive was sent to all the state governments on establishment of Rape Crisis Centres (RCCs) and specialised Sexual Assault Treatment Units (SATUs) in 2009, no such units have been set up in the states even now, except in New Delhi (13). The law states that a female police officer should record the victim's statement, as well as assist her with medical and legal support. However, female police personnel account for only 6.5% of the police force, which makes it difficult to implement this. Further, the government health services in the country lack the infrastructure and resources needed to implement care for rape victims as specified by the law in most district and sub-district hospitals. It has been reported that traumatised victims often have to go from one hospital to another for forensic examination following rape (14). Victims often sit for hours in soiled clothes in the hospital and feel humiliated all over again in the course of insensitive history-taking by doctors and health workers. Judgemental attitudes and lack of privacy in government healthcare establishment worsens their trauma (14).

## Tip of an iceberg: women in Indian society

In a democracy, it is said that the politicians are only as good as the people. The deep-rooted patriarchy of Indian society lay exposed when several people, including senior politicians, type casted the victims of sexual violence, as possibly having contributed to the perpetration of the crime (15). Some of the typical characterisations of the victims included women who dressed ‘provocatively’, ‘was out late in the night’ or was ‘behaving in a suggestive way that invited trouble’. Others suggested in an apparent gesture of sympathy that the rape victim becomes a *living corpse* indicating the life of shame that the victims of sexual abuse will be subjected to in the country.

The problem of gender-based violence runs very deep in India. The rape crisis is just one facet of the multitude of problems that reflect the gender discrimination scenario. These prejudicial attitudes are seen right from womb to tomb (16). They start with the practice of sex-selective abortion and infanticide, and continue through adolescent and adult life with high levels of female infant mortality, child marriage, teenage pregnancy, lesser wages for women, unsafe workplaces, domestic violence, maternal mortality, sexual assault and neglect of elderly women.

India has made great strides in terms of economic growth in the past decade. The increase in female literacy rates from 54% in 2001 to 65% in 2011 (17) and improved maternal mortality ratio from 327 in 2001 (18) to 178 in 2012 (19) suggest a movement towards greater gender equality in the country. However, the 2011 census showed that the child sex ratio dropped to its lowest value since independence to 914 females for every 1,000 males, showcasing the continuing trend of boy preference. What is surprising is that socio-economic development alone does not modify this trend. Even states that rank high on the HDI report relatively low child sex ratio and high female infant mortality rate (see Table 1) (20–22). In other aspects of gender equality too, such as education or participation of women in the workforce, or representation of women in elected bodies, India falls short of international standards. It ranks 136 out of 186 among the nations of the world in the United Nations Development Programme's gender inequality index. Violence against women, either sexual or physical – wife beating is widely spoken of, but hardly reported – is an expression of power asymmetry between men and women. It seems to co-occur with other indicators of gender disparity. The dismal reporting rate of domestic violence can also be attributed to the attitudes of the victims. According to National Family Health Survey 3 (NFHS-3) data, 41% of women believed their husbands were justified in slapping them and 35% of the women even believed that a brutal beating is also justified if they neglected doing the household chores or looking after their children.

**Table 1:** Comparison of the CSR and FIMR amongst Indian states with High and Low Human Development Index

State	Human Development Index	CSR (the number of females per thousand males in the age group 0–6 in a human population)	FIMR (the number of deaths of female children less than one year of age per 1,000 live births)
India	0.554	914	52
Delhi	0.750 (Rank=3)	866	34
Himachal Pradesh	0.652 (Rank=4)	906	45
Punjab	0.605 (Rank=5)	846	39
Haryana	0.552 (Rank=9)	830	53
Odisha	0.362 (Rank=22)	934	66
Chhattisgarh	0.358 (Rank=23)	964	57

*Sources*: From Refs. 20, 21, and 22.

CSR, child sex ratio; FIMR, female infant mortality rate.

## Women and Indian cinema

The portrayal of societal themes in popular cinema could be considered as a reflection of popular societal attitudes. In Indian cinema, ‘kissing’ was not allowed on-screen on the grounds of modesty until the mid-2000s. However, rape or *izzat lootna* (dishonouring) of women has been a recurrent theme and sub-theme in mainstream Bollywood cinema for decades now, examples are movies like *Insaaf Ka Tarazu* or more recently *Woh Lamhe*. Rape and subsequently avenging rape often forms the central narrative of many films. Rape also appears as a sub-plot to reinforce the heroic role of male actors in films (23). The familiar portrayal of rape and sexual assault of women in cinema, however tacit, is disturbing in its lack of censorship (versus censorship of acts like kissing, for example) and its conflicting pervasiveness in a mainstream form of entertainment. However, this is not to deflect from the limited, but realistic representation of rape and forms of sexual abuse in alternate films such as *Bandit Queen* 4 (Shekhar Kapur, 1994) and *Monsoon Wedding* (Mira Nair, 2001) which used the film media to bring the issues into mainstream discourse. Another genre of mainstream media the *saas–bahu serials* (translation: *mother-in-law and daughter-in-law* soaps) have been acknowledged for their role in featuring other forms of violence and discrimination within Indian households. At a time when the country is introspecting its treatment of women, it would be useful to remind ourselves that sexual violence in the popular media may be a way of highlighting issues of violence against women and may also, in many cases, be an echo of pervasive prejudices in our society (24). Although the presence and acceptance of violence against women in mainstream media in India warrants further research, its existence as a reflection of societal attitudes is indeed indisputable (Box 1).

Box 1 **Women and Indian cinema**

In the 1978 film *Ghar*, a newlywed couple on their way home, are attacked by a group of young men (drinking, driving a car and listening to music). The wife is abducted and raped. The rest of the film/script aside, the construction of the 3 min sequence from 1978 could be considered reflective of society even today. The point to note here is the portrayal of the woman. She is portrayed as an honourable or a virtuous woman owing to the fact that she was (a) married; (b) she was walking with *her husband* when she was attacked. The rapists’ portrayal however was as men not known to the victim, but rather as indulgent young men out to have a good time in a car, under the influence of alcohol. According to 2012 NCRB data, rapists were known to the victim in as many as 98.2% of the cases (15).

While on one hand it is essential to develop comprehensive laws to address this issue, laws are not the only solution. The effectiveness of these laws depends on women's awareness of these laws and their ability and ease to call upon them, if need be. Legal awareness among women is an important step towards improving the reporting rates of rapes. Allotment of adequate funds to build the necessary infrastructure and to ensure the enactment of this law through qualified and trained personnel are the steps to be taken by the government. In terms of judicial measures, although the amendment to criminal law states that all rape cases should be tried in fast-track courts and the trial to be completed within 2 months, without the necessary judicial reforms and infrastructure in place it will be extremely difficult to achieve the desired conviction rate. According to NCRB statistics, 83.6% of cases are still pending in courts across various states in the country. With this poor conviction rate, even the most stringent of laws do not serve their purpose.

The government of India failed to incorporate several important recommendations of the three- member committee that was formed to amend the criminal law (3), the crucial one being the criminalisation of marital sexual abuse. According to the National Family Health Survey report of 2005–06 (25), 9% of all women aged 15–49 experienced sexual violence at some time during their lifetime. Out of these 87.5% reported that the perpetuator was their current husband. With over 104 countries in the world outlawing marital rape, it is only imperative for India to follow in their footsteps without using tradition or the institution of marriage as an excuse.

## Gender-based violence and public health

As health workers (doctors, nurses and public health professionals), we all come across various facets of this issue. Be it an out-patient encounter with a victim of domestic violence presenting with non-specific complaints, or post-rape care of a victim at a primary care facility, health workers’ appreciation of this societal problem is an important part of the solution. Public health implications of rape are numerous. The repercussions of sexual violence are beyond the victim and include the family as well as society at large.

According to the NCRB statistics (6), out of the 24,923 reported rape cases in the country during the year 2012, offenders were known to the victims in as many as in 24,470 (98.2%) cases. Primary healthcare/emergency healthcare professionals, who may be the first point of contact for these victims should be able to recognise the signs of a sexual assault and report them to the appropriate authorities. It is essential to train these personnel not only in medical treatment of these victims, but also in providing psychosocial support to them. To incorporate routine screening of violence into healthcare practise, healthcare professionals’ preparedness to treat such patients needs to be assessed and they should be trained in the maintenance of confidentiality, positive attitudes, and respect for patients’ rights.

Provision of medical as well as legal support to the victims at their first point of contact following the incident can increase the reporting as well as conviction rate in these crimes. Ensuring the provision or providing the victims with emergency contraception pill (ECP) or post-exposure prophylaxis (PEP) should be the responsibility of the first point of contact as these are immediate measures, most effective within the first 72 hours after the incident. Development and training of both police as well as medical personnel on standardised protocols in post-rape care is imperative.

Conviction in any criminal case largely depends on the forensic evidence. Abysmal conviction rate of rape cases, standing at 16% of the total, can be improved by meticulous forensic sample collection and transfer to the concerned authorities. Every medical officer in public health services should be trained to perform physical assessment as well as meticulously maintaining the medical records. They should be trained in sample collections of forensic evidence from the victims and aid the police and other concerned authorities in providing evidence. Advocacy campaigns about the optimum post-rape care should be undertaken by public health professionals to achieve these goals.

Establishment of RCCs and SATUs in all major cities and towns is essential, which can act as a one-stop centre for medical, social, psychological and legal support of rape victims. Government, in collaboration with NGOs and other public health organisations, should set up helplines through which the victims have access to a network of professionals who are trained to support them in seeking care as well as legal recourse. Gender sensitisation of personnel towards this sensitive issue is another step that needs to be taken to make sure that the law enforcers as well as medical staff – largely men – are more sensitive towards rape victims.

## Conclusion

Gender-based violence, especially violent crime like rape, is a multifaceted problem. To address this, it is essential to tackle various other concurrent issues that act as contributing factors and thus play an equally important role. An example for this is the portrayal of women in Indian cinema. This bears evidence to the deep-rooted prejudicial attitudes towards women and other deeper societal issues that are contributory to these crimes. Although the incorporation of stringent laws and stricter punishments are important to deter people from committing such crimes, the solution to this is much more than just promulgation. Though the amendment to criminal law addresses a few of these issues, it still falls short in many aspects. It is important to acknowledge that judicial reform is only one aspect; there is a more humane side to this whole issue. Legal solutions in the form of amendments to improve conviction rates could function as deterrents to such acts. However, in such a scenario health workers could play a key role in applying a gender lens to their work as healthcare providers, researchers and policymakers. In a country with gender discrimination operating at so many levels and in so many ways, bringing about the needed change requires dedicated and combined efforts of multiple agencies. While education and empowerment of women is a larger social process to which public health professionals may not be able to contribute directly, we urge health workers and public health professionals to facilitate improved access, utilisation and coverage of women in the services that we study, plan, implement and evaluate. Doctors, nurses and other healthcare providers, researchers and public health professionals need to respond to this social predicament individually and engage with this problem in their own families, organisations and communities.

## Footnotes

1 GINI coefficient: The GINI coefficient is a measure of statistical dispersion intended to represent the income distribution of a nation's residents.

## Footnotes

2 Eve-teasing: the making of unwanted sexual remarks or advances by a man to a woman in a public place.

## Footnotes

3 Dowry: an amount of property or money brought by a bride to her husband on their marriage.

## Footnotes

4 Bandit Queen is based on a real-life bandit turned politician, Phoolan Devi.

## References

[1] Ghosh P (2012). Delhi: the Center of India's growing rape crisis. http://www.ibtimes.com/delhi-center-indias-growing-rape-crisis-705482.

[2] O'Hare S (2012). India's rape crisis revealed: now 16-year-old runaway is ‘attacked by bus conductor’ as full horror of sex assaults emerges’. Daily Mail.

[3] The Gazette of India Department of Publication, Ministry of Urban Development, Government of India. http://egazette.nic.in/RecentUploads.aspx?Category=1.

[4] Kurup D (2013). Death sentence is a quick-fix option. http://www.thehindu.com/news/cities/bangalore/death-sentence-is-a-quickfix-option/article5125470.ece.

[5] Ghosh P (2013). Delhi gang-rapists sentenced to death: India's conflicting attitudes towards capital punishment. http://www.ibtimes.com/delhi-gang-rapists-sentenced-death-indias-conflicting-attitudes-towards-capital-punishment-1405448.

[6] Government of India Ministry of External Affairs. National Crime Records Bureau: Crime in India – 2012.

[7] The World Bank (2013). Working for a world free of poverty. http://data.worldbank.org/indicator/SI.POV.GINI/countries?order=wbapi_data_value_2011%20wbapi_data_value&sort=asc&display=default.

[8] Government of India Ministry of External Affairs. National Crime Records Bureau: Crime in India – 2012.

[9] National Crime Records Bureau (2011). Cases registered and their disposal under Crime against Women during 2010 – 11. http://ncrb.nic.in/CDCII2011/Additional%20table%20cii%202011/Cases%20registered%20and%20their%20disposal%20under%20Crime%20against%20Women%20during%20-%202011final.xls.

[10] Dreze J, Sen A (2013). An uncertain glory: India and its contradictions.

[11] Global Press Institute (2012). Mass rape survivors still wait for justice in Kashmir. http://www.trust.org/item/20120307023000-i7m26/?source=search.

[12] Sen A, Bagriya A (2013). Manorama Devi rape-murder: no action against armed forces yet. IBN Live.

[13] Dhar A (2013). States ignored Centre's advisory on Rape Crisis Centres. http://www.thehindu.com/news/national/states-ignored-centres-advisory-on-rape-crisis-centres/article4284055.ece.

[14] Human Rights Watch (2005). Dignity on trial: India's need for sound standards for conducting and interpreting Forensic examinations of rape survivors.

[15] India's insensitive political class: who said what after Delhi gang-rape (2013). http://daily.bhaskar.com/article/DEL-india---s-insensitive-political-class-who-said-what-after-delhi-gang-rape-incide-4137388-NOR.html.

[16] Sen A (2003). Missing women—revisited. BMJ.

[17] Status of literacy (2011). http://censusindia.gov.in/2011-prov-results/data_files/mp/07Literacy.pdf.

[18] Government of India (2013). India country report 2013 – statistical appraisal. http://mospi.nic.in/mospi_new/upload/SAARC_Development_Goals_%20India_Country_Report_29aug13.pdf.

[19] Office of Registrar General, India (2013). Special bulletin on maternal mortality in India 2010–12. http://www.censusindia.gov.in/vital_statistics/SRS_Bulletins/MMR_Bulletin-2010-12.pdf.

[20] Government of India Ministry of Finance. Union budget and economic survey: economic survey 2011–12.

[21] John EM (2007). Census 2011: Governing populations and the girl child. Economic and Political Weekly.

[22] Government of India Ministry of Health and Family Welfare, Statistics Division. Family welfare statistics in India – 2011.

[23] Datta S (2000). Globalisation and representations of women in Indian cinema. Soc Sci.

[24] Gopalan L (1997). Avenging women in Indian cinema. Screen.

[25] International Institute of Population sciences (IIPS) and Macro International (2007). National Health and Family Survey (NFHS-3), 2005–06. http://hetv.org/india/nfhs/nfhs3/NFHS-3-Chapter-15-Domestic-Violence.pdf.

# Sub article: A cross-sectional study on attitudes toward gender equality, sexual behavior, positive sexual experiences, and communication about sex among sexually active and non-sexually active adolescents in Bolivia and Ecuador

**DOI:** 10.3402/gha.v7.24089

**Published:** 2014-07-11

**Authors:** Sara De Meyer, Lina Jaruseviciene, Apolinaras Zaborskis, Peter Decat, Bernardo Vega, Kathya Cordova, Marleen Temmerman, Olivier Degomme, Kristien Michielsen

**Affiliations:** 1 International Centre for Reproductive Health (ICRH), Ghent University, Ghent, Belgium; 2 Department of Family Medicine, Lithuanian University of Health Sciences (LUHS), Kaunas, Lithuania; 3 Faculty of Medicine, University of Cuenca, Cuenca, Ecuador; 4 South Group, Cochabamba, Bolivia

**Keywords:** adolescents, gender attitudes, Latin America, sexual behavior, positive sexual experiences

## Abstract

**Background:**

It is widely agreed upon that gender is a key aspect of sexuality however, questions remain on how gender exactly influences adolescents’ sexual health.

**Objective:**

The aim of this research was to study correlations between gender equality attitudes and sexual behavior, sexual experiences and communication about sex among sexually active and non-sexually active adolescents in 2 Latin American countries.

**Design:**

In 2011, a cross-sectional study was carried out among 5,913 adolescents aged 14–18 in 20 secondary schools in Cochabamba (Bolivia) and 6 secondary schools in Cuenca (Ecuador). Models were built using logistic regressions to assess the predictive value of attitudes toward gender equality on adolescents’ sexual behavior, on experiences and on communication.

**Results:**

The analysis shows that sexually active adolescents who consider gender equality as important report higher current use of contraceptives within the couple. They are more likely to describe their last sexual intercourse as a positive experience and consider it easier to talk with their partner about sexuality than sexually experienced adolescents who are less positively inclined toward gender equality. These correlations remained consistent whether the respondent was a boy or a girl. Non-sexually active adolescents, who consider gender equality to be important, are more likely to think that sexual intercourse is a positive experience. They consider it less necessary to have sexual intercourse to maintain a relationship and find it easier to communicate with their girlfriend or boyfriend than sexually non-active adolescents who consider gender equality to be less important. Comparable results were found for boys and girls.

**Conclusions:**

Our results suggest that gender equality attitudes have a positive impact on adolescents’ sexual and reproductive health (SRH) and wellbeing. Further research is necessary to better understand the relationship between gender attitudes and specific SRH outcomes such as unwanted teenage pregnancies and sexual pleasure among adolescents worldwide.

It is widely agreed upon that gender is a key aspect of sexuality (1–4); however, questions remain on how gender exactly influences adolescents’ sexual health (2, 5, 6). The relationship between gender and sexuality is a multifaceted and multi-determined social process (7), strongly affected by societal, interpersonal, and personal factors (1, 8). During adolescence, sexual and reproductive development and health are key issues that go hand in hand with gender equality (2, 9). Studies have shown that less egalitarian gender norms threaten the sexual health and wellbeing of adolescents (2, 10–13). Adolescent boys with less egalitarian gender norms are more likely to engage in sexual risk behavior, such as having multiple sexual partners. Adolescent girls with less egalitarian gender norms are more vulnerable to negative sexual and reproductive health (SRH) outcomes, such as experiencing sexual coercion (6, 8, 14).

## Background

On a societal level, former research clearly indicated that social influences and cultural attitudes have an effect on sexual behavior among adolescents (2, 8, 14). Different gender norms exist for adolescent boys and girls (2). Adolescents internalize these social norms and values before they become sexual active (15) and their sexual attitudes and behavior are shaped by them (6, 14, 16).

On an interpersonal level, research indicated the need to further explore factors that are related to adolescent communication about sexuality (17, 18). It has been proven that gender stereotypes shape the way young people communicate (19) and that communication about sexuality is different for adolescent boys and girls (18, 20–22).

On an individual level, aspects such as norms and attitudes influence adolescents’ sexual behavior and experiences. Girls tend to have more egalitarian gender role attitudes than boys who claim to have more traditional gender role attitudes (6, 23). These differences play a role in diverse sexual behavior for both sexes (2). Little is known on the association between adolescent sexual behavior and sexual pleasure (2, 24). However, research has proven that adolescents consider sexual pleasure to be an important goal in their relationships and that they expect sex to result in sexual pleasure (13). Studies among students in the United States have shown that gender equality and perceived equity in a relationship are both associated with sexual enjoyment (12, 25). Similar data were found among Swedish adolescent heterosexual girls who mentioned sexual pleasure on equal terms as a characteristic of their ideal sexual situation (26).

Limited research in Latin America confirmed these results. At societal level, Latino cultures are characterized by a cultural machismo–marianismo system, which includes a traditional gender ideal of male dominance and female submission (10, 27, 28). Studies in the Caribbean and Ecuador indicated that these diverse social and cultural gender norms lead to different sexual behavior among boys and girls (8, 28). The macho male adolescents are supposed to be heterosexual, have many sexual partners and should engage in higher sexual risk behavior than the female adolescents who are expected to be innocent and self-sacrificing and therefore more vulnerable to negative SRH outcomes (8, 28, 29). These traditional gender norms also constitute barriers for adolescent girls to enjoy sexual experiences (10, 28).

At an interpersonal level, research in the United States has shown that a cultural Latin American background increases the difficulty to communicate on sexuality (30, 31). The Horizon project in Brazil concluded that boys who participated in interventions that promoted gender equitable behavior, communicated with their primary partners about a broader range of key HIV/STI-related topics (29).

At an individual level, the same project indicated that men who had more equitable gender norms showed less sexual risk behavior (32). Research in Ecuador and Brazil found an increasing tendency among adolescents to have more consensual and pleasant sex and depicts a close relationship between gender norms and adolescent sexual pleasure (10, 28, 32).

We can conclude that evidence on the association between gender and adolescent sexuality exists and is growing. The importance of gender for adolescents’ sexuality is also recognized by international organizations such as the United Nations Population Fund and the World Health Organization (WHO) who recognize the need to address gender as an ‘upstream’ antecedent of adolescents’ sexual health behavior (33, 34). However, until now in Latin America, only limited research was conducted on societal, interpersonal and personal levels to understand the link between gender equality and adolescents’ sexuality (8, 10, 27–29, 32). On a societal level, comparing different gender equality indicators could assess this relationship. Nevertheless, our research focusses on the individual and interpersonal level, while taking into account that adolescents remain the main target group in changing behavior programs to improve their sexual health. We defined the interpersonal level as the level which includes factors that are related to the interaction of the adolescent with their partners, peers and parents. The main objective of this article is to describe how gender equality attitudes among adolescents in Latin America are correlated to their sexual behavior, positive sexual experiences and communication about sex. To the best of our knowledge, these correlations have not yet been structurally studied in any large-scale research performed in Latin America.

## Methods

This paper presents partial results of the international interventional research project Community Embedded Reproductive Health Care for Adolescents in Latin America (CERCA), funded by the European Commission (35). CERCA seeks to create a community-based model to improve adolescent health, by organizing activities such as workshops, family visits, sending informative text messages and psychological counseling. The topics treated were related to SRH and wellbeing for adolescents, communities, health care providers and authorities. The intervention ran for a period of approximately 2 years.

In 2011, a cross-sectional study was carried out among 5,913 adolescents aged 14–18 years in 20 secondary schools in Cochabamba (Bolivia) and 6 secondary schools in Cuenca (Ecuador). These schools were purposively selected according to a strategy developed by the CERCA consortium: 1) selection of primary health care centers that took part in the interventions: 2 in Cochabamba and 3 in Cuenca; 2) selection of secondary intervention schools that fell within the area of coverage of these health care centers: 12 in Cochabamba and 3 in Cuenca; and 3) selection of secondary control schools within the area of coverage of other primary health care centers: 8 in Cochabamba and 3 in Cuenca. The selected intervention and control schools had similar characteristics (socio-economic indicators, geographic location and the size of school). The survey was conducted in both intervention and control schools (20 out of a total of 1,100 schools in Cochabamba and 6 out of a total of 127 schools in Cuenca) before the intervention started. The amount of selected schools and participants was based on the calculations for a cross-sectional control study measuring the impact of interventions on contraceptive use among adolescents. We estimated that among 14–18 year-old adolescents, 30% are sexually active and that 30% of the sexually active adolescents use a modern contraceptive. Using the finite population correction factor, we determined that a minimum of 2,057 respondents was needed in each country to detect a significant difference of 10% in contraceptive use between the intervention and control groups. Due to the larger amount of schools in Cochabamba, within the area of coverage of the selected health care centers, more schools were selected in Cochabamba than in Cuenca. In Cochabamba, the selected health care centers and schools were located in 3 different zones, which are all urban with basic health services. One zone is mainly populated by middle-income families (Sarcobamba); the other 2 areas (Central Cochabamba and Quintanilla) have a mix of very poor and very rich inhabitants. In Cuenca, the survey was conducted in urban (parish Cañaribamba) and rural areas (parish El Valle and Chiquintad). The parish of Chiquintad is characterized by migration and poverty; the other 2 parishes have residents of middle-income. Due to the fact that the selected schools in Cuenca were mainly technical schools, girls were underrepresented in our sample. However, our sample had a representative mix of adolescents with an urban versus rural (only Ecuador) and diverse socio-economic background.

In Bolivia, the study was approved on 6 July 2011 and in Ecuador on 13 September 2011 by the relevant ethical committees (Tribunal de Ética Médica Colegio Médico de Cochabamba and Comisión de Bioética Facultad de Ciencias Médicas, Universidad de Cuenca). In both countries, different procedures were applied, depending on the national legislation. In Bolivia, the permission of the Ministry of Education and institutional permission of all selected schools was obtained. Subsequently, trained CERCA staff invited all 14–18 year-old students who were present that day, to complete a self-administered questionnaire after having signed an informed consent form (36). In the selected schools in Ecuador, firstly, institutional permission was obtained and secondly, all parents or guardians of the students were, during a school meeting, informed by project staff about the study. Afterwards, they were asked to provide written refusal or agreement on the participation of their children. The students who had parental permission to participate were asked to sign an informed consent. Of all Ecuadorian students who received approval of their parents to participate (all but 11), 2 refused to take part in the study. In Bolivia all invited adolescents participated. In total, 4,000 adolescents in Bolivia and 2,699 in Ecuador (total 6,699 adolescents), aged between 14 and 18, took part in the research. However, 486 questionnaires in Bolivia and 300 in Ecuador were invalid and excluded from the study. This brought the total final sample down to 5,913 adolescents.

One of the investigated topics was adolescents’ gender attitudes, which was measured using the Attitudes toward Women Scale for Adolescents (AWSA) (37). This scale is widely used to assess gender attitudes among adolescents (38–42). Galambos et al. found high reliability for the internal consistency estimates and the test–retest stability of the scale as well as a large support for the construct validity of the scale (37). For this study, the Spanish version of AWSA was used. The scale was pilot tested before conducting the survey.

A factor analysis of the AWSA scale was performed and is described in detail in the authors’ article on the AWSA scale (36). Three subscales emerged from the factor analysis: power dimension, equality dimension and behavioral dimension. When testing the external validity of the different factors, mainly the factor of equality dimension revealed a consistent correlation with adolescent sexual behavior. This article provides a more in-depth analysis of the equality dimension subscale. Respondents were asked if they agreed with the following 4 statements which refer to the fact that men and women should have the same rights and opportunities (in brackets the number of the item in the AWSA is presented):

(V03) On average, girls are as smart as boys
(V05) It is alright for a girl to want to play rough sports like football
(V09) If both husband and wife have jobs, the husband should do a share of the housework such as washing dishes and doing the laundry
(V12) Girls should have the same freedom as boys

Each item represents an attitude to which the study participants responded on a 4-point Likert-type scale ranging from 1 ‘strongly agree’ to 4 ‘strongly disagree’. In the analyses, the responses were reversely coded to create a gradient with higher scores indicating a more positive attitude toward gender equality. For each item, on average 40 (0.7%) values were missing. They were replaced by the estimated mean from the respondent's answers to the remaining items. For further analysis, the values of the factorial scores on the equality dimension were divided in 3 categories, using their terciles as cut-off points and coded as: 1=low, 2=medium and 3=high positive attitude toward gender equality. Due to differences in responses, this procedure was performed by splitting data into: respondents’ sex, age group (14–16 and 17–18) and country.

The Cronbach α was used as a measure of internal consistency of the subscale. The subscale was subjected to the Spearman–Brown prediction formula to adjust its reliability to the reliability of the full 12-item test (43). A Cronbach *α*≥0.70 (0.70 for girls and 0.73 for boys) was considered acceptable.

The statistical analyses were done using SPSS 21.0. As the sample of our study was not randomly selected, in all analyses, data were adjusted through weighing by sex, country and age, using the average distribution of the respondents as a standard population. By weighing, we aimed at reflecting the distribution in the general study population.

Logistic regressions were performed to estimate the adjusted odds ratios (aORs) and their 95% confidence intervals of adolescents’ sexual behavior, sexual experiences and communication about sex in relation to adolescents’ attitudes toward gender equality. The goodness of fit of the logistic regression model was evaluated calculating the Hosmer and Lemeshow test (*p*>0.05 indicating an acceptable model). Interactions between sex and gender equality attitudes (both categorical variables) were tested.

Sexual behavior was measured, using the following questions and variables: ‘Did you ever have sexual intercourse (coitus)?’ (yes/no), ‘Do or did you feel pressure to have sexual intercourse because a lot of your peers already had sexual intercourse?’ (yes/no), number of sexual partners (2 or more/1), current contraceptive use of couples (yes/no) (current use of contraceptives or the use of a condom during the 3 most recent sexual experiences), the fact whether both had taken the initiative to have sexual intercourse the last time (yes/no) and the agreement on the necessity to have sexual intercourse to maintain a relationship (yes/no).

Positive sexual experiences for sexually active adolescents were measured by the outcomes ‘positive experience’ and ‘not positive experience’ (neutral, negative, don't know) on the question ‘How did you feel the last time you had sexual intercourse?’ For sexually inactive adolescents a bivariate variable was formulated based on the question ‘Do you think that sexual intercourse is a positive experience?’

The bivariate variable (yes/no) ‘easy communication with the partner’ is based on the answers of adolescents who indicated currently having a partner.

We assessed the predictive role of attitudes toward gender equality in different components of adolescents’ sexual behavior, experiences and communication for sexually active and for sexually non-active adolescents separately. The same confounding factors (age, sex, country, living with mother/father, living conditions, and importance of religion) were included in both models as adjusting components. The confounders were identified based on correlation analysis and on literature research.

## Results

Of the 5,913 respondents, 3,330 were boys and 2,583 were girls, 59.4% were Bolivian and 40.6% Ecuadorian. Of all respondents 23.4% ever had sexual intercourse. In the overall sample, 93.9% of the respondents completed the AWSA scale. One hundred and thirty-seven respondents (2.3%) did not respond to any of the items V03, V05, V09 and V12 and were therefore excluded from the analysis. Table 1 describes the crude and the weighted distribution of respondents by social, demographic and sexual outcome variables.

**Table 1:** Crude and weighted distribution of respondents by social, demographic and sexual outcome variables

	Crude number of cases	Weighted number of cases
	
Characteristics (predictors and outcome variables)	*n* (%)	*n* (%)
**All cases**	5,913 (100.0)	5,913 (100.0)
Sex		
Boys	3,330 (56.3)	2,957 (50.0)
Girls	2,583 (43.7)	2,956 (50.0)
Age (years)		
14	1,173 (19,8)	1,183 (20.0)
15	1,451 (24.5)	1,183 (20.0)
16	1,456 (24.6)	1,183 (20.0)
17	1,274 (21.5)	1,182 (20.0)
18	559 (9.5)	1,182 (20.0)
Country		
Bolivia	3,514 (59.4)	2,957 (50.0)
Ecuador	2,399 (40.6)	2,956 (50.0)
Living with mother during the last 3 years		
Less than last 3 years	1,043 (18.2)	1,034 (18.1)
3 years or more	4,696 (81.8)	4,691 (81.9)
Living with father during the last 3 years		
Less than last 3 years	2,273 (39.5)	2,437 (42.4)
3 years or more	3,486 (60.5)	3,315 (57.6)
Quality of living house		
Poor	2,069 (35.0)	2,125 (36.0)
Good	3,834 (65.0)	3,779 (64.0)
Importance of religion		
Not important	1,487 (26.9)	1,344 (24.3)
Important	4,047 (73.1)	4,186 (75.7)
Factor of gender equality		
Low	1,963 (33.2)	1,971 (33.4)
Middle	2,077 (35.1)	2,042 (34.5)
High	1,873 (31.7)	1,900 (32.1)
Had sexual intercourse (penetration)		
No	4,518 (76.6)	4,341 (73.6)
Yes	1,379 (23.4)	1,557 (26.4)
**Those who had sex**	1,379 (100.0)	1,557 (100.0)
Number of sexual partners		
1	596 (51.2)	719 (53.6)
2 or more	569 (48.8)	622 (46.4)
Actual use of contraceptives		
No	954 (69.2)	1,054 (67.7)
Yes	425 (30.8)	502 (32.3)
Experience of last sexual intercourse		
Not positive	518 (39.5)	531 (35.8)
Positive	794 (60.5)	953 (64.2)
Mutual initiative to have sexual intercourse the last time		
No	767 (55.6)	798 (51.3)
Yes	612 (44.4)	758 (48.7)
Pressure to have sexual intercourse		
No	1,010 (85.6)	1,169 (86.9)
Yes	170 (14.4)	175 (13.1)
Easy communication with partner about sex		
No	432 (37.8)	457 (36.3)
Yes	712 (62.2)	802 (63.7)
**Those who did not have sex**	4,518 (100.0)	4,341 (100.0)
Ideas about sexual intercourse		
Not positive	3,336 (76.8)	3,245 (77.5)
Positive	1,005 (23.2)	943 (22.5)
Agreement with necessity to have sexual intercourse to maintain a relationship		
Did not agree	3,271 (74.4)	3,205 (75.7)
Agreed or did not know	1,128 (25.6)	1,031 (24.3)
Feeling the pressure to have sexual intercourse		
No	2,991 (89.3)	2,884 (89.5)
Yes	359 (10.7)	340 (10.5)
Easy communication with partner about sex		
No	985 (69.3)	952 (68.5)
Yes	436 (30.7)	437 (31.5)

Table 2 displays the distribution of the scores per AWSA item by sex and age.

**Table 2:** The distribution of the scores per AWSA item by sex and age

	Distribution of responses to the items of the gender equality subscale^a^							
	
	V03		V05		V09		V12	
				
Group of respondents	Disagree	Agree	Disagree	Agree	Disagree	Agree	Disagree	Agree
Boys	770 (26.3%)	2,157 (73.7%)	1,041 (35.3%)	1,904 (64.7%)	260 (8.8%)	2,679 (91.2%)	667 (22.7%)	2,271 (77.3%)
Girls	508 (17.3%)	2,426 (82.7%)	808 (27.6%)	2,120 (72.4%)	127 (4.3%)	2,805 (95.7%)	455 (15.5%)	2,486 (84.5%)
*p*^b^	<0.001		<0.001		<0.001		<0.001	
14–16-year old adolescents	808 (23.0%)	2,707 (77.0%)	1,158 (32.9%)	2,363 (67.1%)	264 (7.5%)	3,259 (92.5%)	610 (17.3%)	2,911 (82.7%)
17–18-year old adolescents	469 (20.0%)	1,876 (80.0%)	692 (29.4%)	1,661 (70.6%)	123 (5.2%)	2,225 (94.8%)	512 (21.7%)	1,846 (78.3%)
*p*^b^	0.007		0.005		0.001		<0.001	

a Responses ‘Strongly disagree’ and ‘Disagree’ were aggregated to ‘Disagree’ and responses ‘Agree’ and ‘Strongly agree’ were aggregated to ‘Agree’;

b Chi-square test.

The mean of the total score on the gender equality subscale was 12.68 and the median 13.00. Girls expressed more positive attitudes toward gender equality than boys (mean scores were 13.10 and 12.27, *p*<0.001, respectively for girls and boys). The scores did not significantly differ by age or by whether or not the adolescents were sexually active.

Table 3 shows the results of the logistic regression for sexually active adolescents. Adolescents who were sexually active and who considered gender equality as important (high vs. low) declared higher current use of contraceptives within the couple, were more likely to describe their last sexual intercourse as a positive experience and considered it easier to communicate with their partner about sex than sexually experienced adolescents who were less positively inclined toward gender equality. Gender equality attitudes were not a significant predictor of ever having had sexual intercourse, of the number of sexual partners, of mutual initiative to have sexual intercourse, or of pressure for sexual intercourse. When calculating the interaction between sex and gender equality, the outcome remained consistent.

**Table 3:** Demographic and social factors predicting different aspects of sexual behavior among sexually active adolescents: odds ratios (ORs) and 95% confidence intervals (CIs) estimated from the multivariate binary logistic regression

	Ever had sexual intercourse		Number of sexual partners		Current use of contraceptives		Positive experience last sexual intercourse		Both have taken initiative to have sexual intercourse the last time		Pressure for sexual intercourse		Easy communication with partner	
							
Predictors	OR	(95% CI)	OR	(95% CI)	OR	(95% CI)	OR	(95% CI)	OR	(95% CI)	OR	(95% CI)	OR	(95% CI)
Age^a^	**1.85**	(1.75; 1.95)***	**1.17**	(1.06; 1.30)**	**1.17**	(1.06; 1.29)**	**1.20**	(1.09; 1.33)***	**1.31**	(1.20; 1.44)***	0.92	(0.80; 1.06)	**1.18**	(1.06; 1.31)**
Country^b^	**1.21**	(1.05; 1.39)**	0.98	(0.76; 1.26)	**1.33**	(1.05; 1.70)*	**1.81**	(1.42; 2.31)***	**1.51**	(1.23; 1.94)***	**0.68**	(0.47; 0.97)*	1.15	(0.88; 1.51)
Sex^c^	**2.17**	(1.89; 2.50)***	**3.1**	(2.41; 3.98)***	0.88	(0.69 1.18)	0.79	(0.62; 1.02)	**0.65**	(0.52; 0.82)***	0.85	(0.60; 1.22)	0.77	(0.58; 1.01)
Gender equality^d^	0.9	(0.77; 1.07)	1.01	(0.75; 1.36)	**1.52**	(1.14; 2.03)**	1.13	(0.85; 1.50)	1.27	(0.97; 1.66)	0.89	(0.57; 1.38)	1.06	(0.79; 1.44)
Gender equality^e^	0.98	(0.84; 1.16)	1.16	(0.87; 1.55)	**1.67**	(1.26; 2.21)***	**1.71**	(1.28; 2.28)***	1.21	(0.93; 1.58)	1.28	(0.85; 1.92)	**1.83**	(1.34; 2.50)**
Living with mother^f^	**0.61**	(0.51; 0.72)***	**0.72**	(0.53; 0.97)*	0.83	(0.62; 1.10)	0.88	(0.65; 1.18)	1.10	(0.83; 1.45)	**0.42**	(0.28; 0.62)***	0.88	(0.64; 1.23)
Living with father^f^	**0.77**	(0.66; 0.89)***	0.95	(0.73; 1.23)	1.15	(0.89; 1.48)	**1.44**	(1.11; 1.86)**	0.90	(0.70; 1.14)	1.12	(0.76; 1.63)	**0.75**	(0.57; 0.99)*
Living conditions^g^	**1.16**	(1.01; 1.34)*	**1.37**	(1.05; 1.78)*	1.13	(0.88: 1.45)	**1.34**	(1.04; 1.73)*	1.12	(0.88; 1.42)	0.80	(0.55; 1.16)	**2.05**	(1.56; 2.69)***
Importance of religion^h^	**0.61**	(0.53; 0.72)***	**0.6**	(0.45; 0.79)***	0.87	(0.67; 1.13)	**1.64**	(1.27; 2.12)***	**1.32**	(1.03; 1.69)*	**0.69**	(0.48; 1.00)*	**0.68**	(0.51; 0.92)*
*p*^i^	0.853		0.133		0.098		0.090		0.090		0.058		0.155	

a Change by 1 year;

b Ecuador vs. Bolivia (ref.);

c boys vs. girls (ref.);

d medium vs. low gender equality (ref.);

e high vs. low gender equality (ref.);

f during last 3 years, lived together with father/mother all time vs. not all time (ref.);

g good vs. poor (ref.);

h religion was considered as a value vs. not a value (ref);

i Hosmer and Lemashow test to evaluate the goodness of fit of the logistic regression model.

* *p*<0.05;

** *p*<0.01;

*** *p*<0.001 (bolded).

The same analysis was done for non-sexually active adolescents (Table 4). In this group, adolescents who considered gender equality as important, were more likely to think that sexual intercourse is a positive experience, considered it less necessary to have sexual intercourse to maintain a relationship and found it easier to communicate about sex with their girlfriend or boyfriend than sexually non-active adolescents who were less supportive toward gender equality. An association between gender equality attitudes and pressure to have sexual intercourse has not been found. Similar aOR have been obtained when including the interaction between sex and gender equality in the model.

**Table 4:** Demographic and social factors predicting different aspects of sexual behavior among adolescents who haven't had sexual intercourse yet: odds ratios (ORs) and 95% confidence intervals (CIs) estimated from the multivariate binary logistic regression

	Positive ideas about sexual intercourse		Agreement to have sexual intercourse to maintain a relationship		Easy communication with partner about sex		Feeling pressure to have sexual intercourse	
				
Predictors	OR	(95% CI)	OR	(95% CI)	OR	(95% CI)	OR	(95% CI)
Age^a^	**1.17**	(1.10 1.24)***	**1.09**	(1.03; 1.15)**	**1.30**	(1.21; 1.40)***	**1.20**	(1.10; 1.31)***
Country^b^	**1.57**	(1.33; 1.86)***	0.96	(0.82; 1.13)	**0.81**	(0.66; 1.00)*	**0.73**	(0.57; 0.93)*
Sex^c^	**3.75**	(3.16; 4.44)***	**3.67**	(3.11; 4.32)***	**1.64**	(1.34; 2.02)***	**2.22**	(1.74; 2.84)***
Gender equality^d^	**1.30**	(1.06; 1.59)*	**0.79**	(0.65; 0.95)***	**1.41**	(1.10; 1.81)**	0.94	(0.70; 1.25)
Gender equality^e^	**1.36**	(1.11; 1.66)**	**0.60**	(0.49; 0.73)***	**1.86**	(1.44; 2.39)***	0.93	(0.70; 1.24)
Living with mother^f^	**1.32**	(1.03; 1.71)*	1.11	(0.87; 1.41)*	0.89	(0.67; 1.18)	0.87	(0.65; 1.17)
Living with father^f^	0.91	(0.77; 1.09)	1.09	(0.92; 1.30)	1.03	(0.83; 1.28)	0.94	(0.66; 1.32)
Living conditions^g^	**1.59**	(1.34; 1.90)***	0.90	(0.77; 1.06)	1.08	(0.87; 1.33)	0.90	(0.69; 1.16)
Importance of religion^h^	1.14	(0.93; 1.39)	**0.77**	(0.64; 0.92)**	1.13	(0.86; 1.44)	0.83	(0.63; 1.10)
*p*^i^	0.173		0.223		0.716		0.234	

a Change by 1 year;

b Ecuador vs. Bolivia (ref.);

c boys vs. girls (ref.);

d medium vs. low gender equality (ref.);

e high vs. low gender equality (ref.);

f during last 3 years, lived together with father/mother all time vs. not all time (ref.);

g good vs. poor (ref.);

h religion was considered as a value vs. not a value (ref);

i Hosmer and Lemashow test to evaluate the goodness of fit of the logistic regression model.

* *p*<0.05;

** *p*<0.01;

*** *p*<0.001 (bolded).

The differences between the groups with medium and high attitudes toward gender equality are small and not significant.

Considering the confounding factors, we can observe that especially religion was positively correlated with the sexual experiences of adolescents and the mutual initiative to have the most recent sexual experience. Except for the outcome of feeling pressure to have sexual intercourse, age was also positively correlated with adolescents’ sexual behavior, experiences, and communication. Negative correlations were found between sexual active adolescents’ religion and the fact that they ever had sexual intercourse, the communication with their partner about sex, their number of sexual partners, and with feeling pressure for sexual intercourse. For non-sexually active adolescents, religion was merely negatively correlated with the agreement of needing to have sexual intercourse to be able to maintain a relationship. For these adolescents who did not have sexual intercourse yet, age is an important confounding factor for all outcomes.

## Discussion

This study investigated how gender equality attitudes among adolescents in Bolivia and Ecuador are linked with sexual topics at the individual level (mainly sexual behavior and positive sexual experiences) and at the interpersonal level (communication with partner about sex). Our study revealed that more egalitarian gender attitudes are related to higher current use of contraceptives within the couple, with more positive experiences and ideas related to sexual intercourse and easier communication about sex with the partner among sexually active and sexually non-active adolescents.

The finding of higher current contraceptive use within couples corresponds with research results found in Brazil, where intervention research indicated the link between gender equitable norms of young men and a higher reported condom use at last sexual intercourse (32). The fact that individual positive attitudes toward gender equality are related to a higher use of contraceptives is not surprising within a Latin American culture, known for its machismo. Having positive attitudes toward gender equality means one breaks free from the typical male role as virile, promiscuous and dominant and from the female stereotype as being innocent, submissive and self-sacrificing (2, 8, 14, 28). This might – at the interpersonal level – open opportunities to discuss not only topics related to HIV, as was demonstrated in Brazil (29), but also to discuss topics concerning contraceptive use. This assumption is in line with our research, which indicates a positive correlation between gender equality attitudes and communication with the partner about sexuality.

Former research indicated an association between physically measured sexual enjoyment and perceived equity among young adults in the United States (12). The research of Goicolea et al. (10) revealed an emerging interest in women's sexual pleasure among Ecuadorian adolescents. Our research is the first to indicate the relationship between attitudes in favor of gender equality and more positive experiences and ideas about sexual intercourse in Latin America.

The fact that no significant difference was found between the groups with medium and high positive attitudes toward gender equality could be related to the characteristics of the Latin American culture, known for its’ distinct gender roles for men and women. As gender equality is not yet widely accepted, the fact that adolescents’ have positive attitudes toward gender equality or not could be more important and significant then the magnitude of these attitudes. We hypothesize that the intensity of gender equality attitudes is of more importance in cultures where gender equality is well accepted.

Our data did not show a correlation of positive attitudes toward gender equality with the number of sexual partners, or with a mutual initiative to engage in the most recent sexual experience or with feeling pressure to have sexual intercourse. Former research conducted in 37 countries concluded that individuals living in highly egalitarian countries are more likely to have more sexual partners, compared to someone living in a country where women's status is significantly inferior to the status of men (44). It might be that we could not find a correlation between adolescents’ individual gender attitudes and their number of sexual partners due to the impact of gender at societal level. Bolivia and Ecuador are respectively ranked 97 and 83 in the list of the Gender Inequality Index (45). This influence of social gender norms can also explain why we did not find a relation between a mutual initiative to engage in the most recent sexual experience and gender attitudes. Regarding sexual pleasure of adolescents girls in Ecuador, Goicolea mentions that, on the individual level adolescent girls may feel equal to boys, but due to powerful cultural expectations, at the interpersonal level they may consider it inappropriate or impossible to take initiative for having sex and thus for seeking sexual pleasure (10). And finally, the fact that our study neither shows correlations between gender equality attitudes and feeling pressure to have sexual intercourse among both sexually active and non-sexually active adolescents, could be influenced by the fact that our question concerning pressure did not exclusively refer to the partner – presumed mostly of the opposite sex. The answers to our question could also imply pressure felt by peers or siblings – of both sexes. If, for example, girls reported about pressure felt by other girls, their decision to initiate sex could mainly be influenced by peer pressure and not by the gender stereotype that, being a girl, they should fulfill their boyfriend's wishes.

Our research indicated that adolescents who considered religion as important, were less likely to have developed (an extensive) sexual life. However, when they were sexually active, they demonstrated more positive experiences and mutual initiative to have sexual intercourse. This significant relationship of religiosity is of interest as it is, besides gender attitudes, an important cultural factor influencing adolescents’ sexual health and wellbeing.

Sexuality education for adolescents in Latin America is rarely widely embedded in the cultural context of a country (46) and still needs improvement. However, our research is in line with former research, which demonstrates the importance of incorporating a gender transformative approach and of promoting gender-equitable relationships between men and women to produce effective behavior that improves SRH (5). The impact of such educational programs could be measured using the ‘gender equality’ scale, which we obtained through factor analysis on the AWSA scale (36). Additionally, we would like to point out that our results suggest that these gender programs could be important for boys and for girls. Although until now, principally boys are targeted in established gender transformative projects to reduce sexual risk behavior and to prevent violence (29), our study depicts that gender attitudes are related to sexual behavior, experiences and communication of both sexes. Furthermore, our results show a correlation between positive attitudes toward gender equality and communication and ideas about sexual intercourse of adolescents who didn't have sexual intercourse yet. This implies that gender transformative programs could also be important for the sexual health and wellbeing of adolescents who are at earlier stages of their sexual trajectory (3).

Our study has various limitations. The first is related to the sampling methodology. It is important to note that the adolescents who participated in the study were not randomly chosen and they all attended schools in the city of Cochabamba (Bolivia) and Cuenca (Ecuador). Hence, we did not capture answers of adolescents in other cities nor from more vulnerable adolescents who lived in rural and poorer areas or who did not go to school. Taking into account the fact that lower socio-economic status is associated with risky sexual behavior (8) and the knowledge that education influences the gender role attitudes of adolescents (23, 47), we could expect to find different results among adolescents in broader target groups. Secondly, we are confronted with blindness within the sample, as we did not gather data on the sexual diversity of our participants. Nevertheless, in a Latin culture where many people define gender roles based on a binary biological division (man vs. woman), a relationship between sexual identity, gender attitudes and sexual behavior could be expected. Thirdly, at the level of analysis and due to the fact that our research was a sub-study within a broader investigation about SRH of adolescents, we were bound by limited socio-demographic features as confounding factors. And finally, inherent to a cross sectional study, our research did not allow to formulate causal relationships between adolescents’ gender equality attitudes and aspects related to their sexual behavior, experiences and communication with their partner.

These limitations of our study indicate the need for additional research to understand how gender has an impact on the sexual behavior of a more diverse group of adolescents. Considering the necessity to incorporate gender into a socio-ecological model of adolescent sexual health, as indicated by Pilgrim et al. (2012) and Tolman et al. (2003), we consider it important to conduct longitudinal research among a randomly selected adolescent population aiming at understanding how gender barriers function at the different levels of the socio-ecological model and how they can be removed in order to ensure healthy and satisfactory sexual health outcomes for all adolescents. Additionally, we would like to suggest to do research on comprehensive indicators for adolescent sexual pleasure. Our research results on the topic can only be viewed as a first step in the systematic measurement of sexual pleasure in Latin America.

In spite of the mentioned limitations, we were able to conduct one of the first systematically performed descriptive researches on the relationship between gender attitudes and sexual behavior, experiences and communication among a large sample of Latin American adolescents.

## Conclusion

Descriptive research in Bolivia and Ecuador has indicated a positive relationship between attitudes toward gender equality and sexual behavior, sexual experiences and communication of sexually active and non-sexually active adolescent boys and girls. Our results suggest that gender equality attitudes have a positive impact on adolescents’ SRH and wellbeing. Further research is necessary to better understand the relationship between gender attitudes and specific SRH outcomes such as unwanted teenage pregnancies and sexual pleasure among adolescents worldwide.

## References

[1] Tolman DL, Striepe MI, Harmon T (2003). Gender matters: constructing a model of adolescent sexual health. J Sex Res.

[2] Marston C, King E (2006). Factors that shape young people's sexual behaviour: a systematic review. Lancet.

[3] de Graaf H, Vanwesenbeeck I, Meijer S, Woertman L, Meeus W (2009). Sexual trajectories during adolescence: relation to demographic characteristics and sexual risk. Arch Sex Behav.

[4] UNFPA (2008). State of world population 2008. Reaching common ground: culture, gender and human rights.

[5] Barker G, Ricardo C, Nascimento M, Olukoya A, Santos C (2010). Questioning gender norms with men to improve health outcomes: evidence of impact. Glob Public Health.

[6] Zuo X, Lou C, Gao E, Cheng Y, Niu H, Zabin LS (2012). Gender differences in adolescent premarital sexual permissiveness in three Asian cities: effects of gender-role attitudes. J Adolesc Health.

[7] Vanwesenbeeck I (2009). Doing gender in sex and sex research. Arch Sex Behav.

[8] Pilgrim NA, Blum RW (2012). Protective and risk factors associated with adolescent sexual and reproductive health in the English-speaking Caribbean: a literature review. J Adolesc Health.

[9] WHO (2012). Preventing early pregnancy and poor reproductive outcomes among adolescents in developing countries.

[10] Goicolea I, Torres MS, Edin K, Ohman A (2012). When sex is hardly about mutual pleasure: dominant and resistant discourses on sexuality and its consequences for young people's sexual health. Int J Sex Health.

[11] Torres VM, Goicolea I, Edin K, Ohman A (2012). ‘Expanding your mind’: the process of constructing gender-equitable masculinities in young Nicaraguan men participating in reproductive health or gender training programs. Glob Health Action.

[12] Galinsky AM, Sonenstein FL (2013). Relationship commitment, perceived equity, and sexual enjoyment among young adults in the United States. Arch Sex Behav.

[13] Ott MA, Millstein SG, Ofner S, Halpern-Felsher BL (2006). Greater expectations: adolescents’ positive motivations for sex. Perspect Sex Reprod Health.

[14] Smith D, Roofe M, Ehiri J, Campbell-Forrester S, Jolly C, Jolly P (2003). Sociocultural contexts of adolescent sexual behavior in rural Hanover, Jamaica. J Adolesc Health.

[15] Machel JZ (2001). Unsafe sexual behaviour among schoolgirls in Mozambique: a matter of gender and class. Reprod Health Matters.

[16] Moore AM (2006). Gender role beliefs at sexual debut: qualitative evidence from two Brazilian cities. Int Fam Plan Perspect.

[17] Schouten BC, van den Putte B, Pasmans M, Meeuwesen L (2007). Parent–adolescent communication about sexuality: the role of adolescents’ beliefs, subjective norm and perceived behavioral control. Patient Educ Couns.

[18] Wilson EK, Koo HP (2010). Mothers, fathers, sons, and daughters: gender differences in factors associated with parent–child communication about sexual topics. Reprod Health.

[19] Lundgren R, Beckman M, Prasad Chaurasiya S, Subhedi B, Kerner B (2013). Whose turn to do the dishes? Transforming gender attitudes and behaviours among very young adolescents in Nepal. Gend Dev.

[20] Lefkowitz ES, Boone TL, Shearer CL (2004). Communication with best friends about sex-related topics during emerging adulthood. J Youth Adolesc.

[21] Sprecher S, Harris G, Meyers A (2008). Perceptions of sources of sex education and targets of sex communication: sociodemographic and cohort effects. J Sex Res.

[22] Afifi TD, Joseph A, Aldeis D (2008). Why can't we just talk about it? An observational study of parents’ and adolescents’ conversations about sex. J Adolesc Res.

[23] Fan P, Marini MM (2000). Influences on gender-role attitudes during the transition to adulthood. Soc Sci Res.

[24] Koyama A, Corliss HL, Santelli JS (2009). Global lessons on healthy adolescent sexual development. Curr Opin Pediatr.

[25] Armstrong EA, England P, Fogarty ACK (2012). Accounting for women's orgasm and sexual enjoyment in College Hookups and relationships. Am Sociol Rev.

[26] Elmerstig E, Wijma B, Sandell K, Bertero C (2012). Sexual pleasure on equal terms: young women's ideal sexual situations. J Psychosom Obstet Gynecol.

[27] Torres JB, Solberg VSH, Carlstrom AH (2002). The myth of sameness among Latino men and their machismo. Am J Orthopsychiatry.

[28] Goicolea I, Wulff M, Sebastian MS, Ohman A (2010). Adolescent pregnancies and girls’ sexual and reproductive rights in the amazon basin of Ecuador: an analysis of providers’ and policy makers’ discourses. BMC Int Health Hum Rights.

[29] Pulerwitz J, Barker G, Segundo M, Nascimento M (2006). Promoting more gender-equitable norms and behaviors among young men as an HIV/AIDS prevention strategy.

[30] O'Sullivan LF, Meyer-Bahlburg HFL, Watkins BX (2001). Mother–daughter communication about sex among urban African American and Latino families. J Adolesc Res.

[31] Morgan EM, Thorne A, Zurbriggen EL (2010). A longitudinal study of conversations with parents about sex and dating during college. Dev Psychol.

[32] Pulerwitz J, Michaelis A, Verma R, Weiss E (2010). Addressing gender dynamics and engaging men in HIV programs: lessons learned from Horizons research. Public Health Rep.

[33] UNFPA (2013). State of the world population 2013. Motherhood in childhood. facing the challenge of adolescent pregnancy.

[34] WHO (2011). The sexual and reproductive health of younger adolescents. Research issues in developing countries.

[35] Decat P, Nelson E, Meyer S, Jaruseviciene L, Orozco M, Segura Z (2013). Community embedded reproductive health interventions for adolescents in Latin America: development and evaluation of a complex multi-centre intervention. BMC Public Health.

[36] Jaruseviciene L, De Meyer S, Decat P, Zaborskis A, Degomme O, Rojas M (2014). Factorial validation of the Attitudes toward Women Scale for Adolescents (AWSA) in assessing sexual behaviour patterns in Bolivian and Ecuadorian adolescents. Glob Health Action.

[37] Galambos NL, Petersen AC, Richards M, Gitelson IB (1985). The attitudes toward women scale for adolescents (AWSA) – a study of reliability and validity. Sex Roles.

[38] Signorella ML, Frieze IH (2008). Interrelations of gender schemas in children and adolescents: attitudes, preferences, and self perceptions. Soc Behav Pers.

[39] Dahlberg LL, Toal SB, Swahn M, Behrens CB (2005). Measuring violence-related attitudes, behaviors, and influences among youths: a compendium of assessment tools.

[40] Neff K, Terry-Schmitt LN (2002). Youths’ attributions for powerrelated gender differences: nature, nurture, or God?. Cognit Dev.

[41] Leaper C, Brown CS (2008). Perceived experiences with sexism among adolescent girls. Child Dev.

[42] Smiler AP, Gelman SA (2008). Determinants of gender essentialism in college students. Sex Roles.

[43] Whiston SC (2009). Principles and applications of assessment in counselling.

[44] Baumeister RF, Mendoza JP (2011). Cultural variations in the sexual marketplace: gender equality correlates with more sexual activity. J Soc Psychol.

[45] UNDP (2013). The 2013 human development report. The rise of the South: human progress in a diverse world.

[46] Demaria LM, Galarraga O, Campero L, Walker DM (2009). [Sex education and HIV prevention: an evaluation in Latin America and the Caribbean]. Rev Panam Salud Publica.

[47] Cunningham M, Beutel AM, Barber JS, Thornton A (2005). Reciprocal relationships between attitudes about gender and social contexts during young adulthood. Soc Sci Res.

# Sub article: Empowerment evaluation of a Swedish gender equity plan

**DOI:** 10.3402/gha.v7.23710

**Published:** 2014-07-02

**Authors:** Georgios Gavriilidis, Nivetha Natarajan Gavriilidou, Erika Pettersson, Eva Renhammar, Anna Balkfors, Per-Olof Östergren

**Affiliations:** 1 Division of Social Medicine and Global Health, Social Medicine and Global Health, Lund University (LU), Malmö, Sweden; 2 City of Malmö, Commission of a Socially Sustainable Malmö, Malmö, Sweden

**Keywords:** policy, empowerment, evaluation, gender equity, policy empowerment index

## Abstract

**Background:**

Empowerment is essential for gender equity and health. The city of Malmö, Sweden, has formulated a development plan for gender equity integration (GEIDP). A ‘Policy Empowerment Index’ (PEI) was previously developed to assess the empowerment potential of policies.

**Objectives:**

To pilot-evaluate the GEIDP’s potential for empowerment and to test the PEI for future policy evaluations.

**Design:**

The GEIDP was analyzed and scored according to electronically retrieved evidence on constituent opinion, participation, capacity development, evaluation–adaptation, and impact.

**Results:**

The plan’s PEI score was 64% (CI: 48–78) and was classified as ‘enabling’, ranging between ‘enabling’ and ‘supportive’. The plan’s strengths were: 1) constituent knowledge and concern; 2) peripheral implementation; 3) protection of vulnerable groups; and 4) evaluation/adaptation procedures. It scored average on: 1) policy agenda setting; 2) planning; 3) provisions for education; 4) network formation; 5) resource mobilization. The weakest point was regarding promotion of employment and entrepreneurship.

**Conclusions:**

The PEI evaluation highlighted the plan’s potential of constituency empowerment and proposed how it could be augmented.

Gender equity and women’s empowerment are social determinants of health (1). Power imbalances result in unequal health access and outcomes, knowledge, skills and employment, living conditions, and opportunities (2). Public policies have major impacts on empowerment (3–7).

Despite the European Union’s (EU) gender equity recommendations, women’s empowerment and inequities in individual, social, economic, and professional conditions remain a challenge in Europe (8, 9). Sweden has the tenth highest human development index, the highest gender equality and the lowest income inequality worldwide (10–12). Gender equity is considered fundamental in Swedish internal policy development (13), and the country has signed the EU plan for gender equity’s integration (9, 14). However, differences exist between socially and economically vulnerable groups and women and men on living conditions, education and health, power and influence in society as well as on employment and unpaid work distribution (15). In addition, Malmö has the most ethnically diverse population among Sweden’s big cities (16, 17). In 2011, the Malmö city office (henceforth, City) developed a gender equity integration development plan (GEIDP), in accordance with EU and national guidance. According to the plan, latest by the year 2013, all of the city agencies and services should be working toward equal distribution of services, resources, power and influence irrespective of gender, orientation, and background, and, as the city’s biggest employer, the City should provide equal employment opportunities, conditions, and salaries, and move toward a balanced gender distribution at all work categories (14).

An evaluation index named ‘Policy Empowerment Index’ (PEI) has been developed by Gavriilidis and Östergren (18), aiming to increase understanding on how policy planning can affect constituent empowerment. It aspires to do so by assessing the policy elements that affect empowerment through evidence reviews, stakeholder, and constituent feedback.

Evidence-based evaluations are necessary for more gender-sensitive and empowering policy planning. A few comparative evaluations have been attempted using ad hoc criteria and policy document content analyses (19–21); however, generally accepted criteria for such evaluations are lacking.

This study aimed to pilot-evaluate the Malmö GEIDP from the perspective of comprehensive empowerment of its constituents, in order to contribute with evidence-based feedback to the policy discussions and to evaluate and develop the PEI for more comprehensive and systematic future policy evaluations.

## Methods

The PEI was previously developed to assess policy elements that have a potential impact on empowerment. It applies logical and discussed scores (from 0=minimum to 5=maximum) of empowerment potential (18) and confidence intervals (0 to 5). Due to lack of evidence on empowering factors’ impacts, the scores are not evidence-weighted. To make the results more visible, the PEI evaluators attach scores to logical statements. For example, <10% describes ‘a small minority’, 10–50% ‘a significant minority’, 50–75% ‘a majority’, 75–90% ‘a big majority’, and 90–100% ‘a vast majority/almost all’, as previously described (18).

A pilot evaluation of the plan was performed after a few adaptations: Questions (Q) 10 and 11 (on policy evaluation and adaptation) were fused, and Q12 was removed (investigation of related policies) for simplicity (see Appendix 1). The remaining 10 questions interrogated the policy plan on the following issues: 1) participation; 2) capacity building; 3) evaluation/adaptability. The index questions cover these policy empowerment elements as follows:

- Constituent concern with the policy issue and participation in agenda setting (Q1, 2), policy planning (Q3), and implementation (Q4).
- Building constituent capacities and opportunities through education/training (Q5), employment/entrepreneurship (Q6), network formation (Q7), addressing power inequalities (Q8), and resource mobilization (Q9).
- Modes of policy evaluation (Q10).

The PEI questions the evaluation of the electronically retrieved evidence addressing them; the PEI standards and scores are presented in Appendix 1. Each question’s argued scores and CI of the GEIDP are averaged to generate a total score and CI, which are presented as proportions of the maximum 50 for intuitiveness. A summary and discussion of the evaluation and the final result is presented in ‘Results’ and in Table 1.

**Table 1:** Summary of PEI evaluation, City of Malmö, development plan for integration of gender equity

Q1. How many political constituents (residents in any way affected by the plan) are informed and concerned with the addressed problem?	Score: 3.5 (3–5)
Q2. How was the political agenda set? Did the plan in question start from a discourse in the community and local grassroots movement advocacy, or by professional experts and politicians at the City level and above (or both in interaction)?	Score: 3 (2–4)
Q3. How was the policy planned? Did peripheral agencies and interest groups contribute significantly to the planning?	Score: 2.5 (2–3)
Q4. What percentage of the development plan actions was delegated peripherally for implementation? Will the plan be implemented mainly by the central or peripheral City authorities?	Score: 5 (4–5)
Q5. Does the policy plan call for education/training of the constituents?	Score: 2 (1–3)
Q6. Is peripheral employment and entrepreneurship being strengthened? Will the plan create jobs or business opportunities for women or men?	Score: 1 (1–2)
Q7. Does the plan promote constituent participation in horizontal and vertical networks? Does the plan create links between the community members/citizens/residents and between them and the authorities of the city?	Score: 2 (1–3)
Q8. Are hard to reach, vulnerable or disadvantaged populations being considered and affirmatively protected and empowered (including vulnerable gender and age groups, socially/physically/economically disadvantaged individuals, groups and communities)?	Score: 5 (4–5)
Q9. Does the policy provide for or will there most likely be adequate financial, human and other resources?	Score: 3 (2–4)
Q10. How will the policy plan be evaluated and adapted?	Score: 5 (4–5)
**Total: 32 (23–39)**	**Score: 64%**
**Policy classification: enabling (supportive–enabling)**	**CI (46–78)**

According to the PEI, a policy can be classified as ‘Dictative’ (score 0–20%), a top–down policy that disregards constituent empowerment, or ‘Empowering’ (81–100%), an optimally focused, planned, and evaluated policy with empowerment in central focus, ‘Directive’ (21–40%), ‘Supportive’ (41–60%) or ‘Enabling’ (61–80%) being the intermediate policy categories as their names imply [see Appendix 2 for the full definitions of these policy categories, as in (18)]. These policy categories are ideal, theoretical models. Real policies may contain elements from more than one policy type, which average on the category indicated by the PEI score.

We conducted evidence and public debate/opinion searches to address each PEI question on constituent opinion and participation in plan design, implementation and evaluation/adaptation, and capacity development through education, employment, networks, and affirmative support. The following search queries were performed on Google, Google Scholar, Web of Science (WoS), and PubMed in English and Swedish using the following search queries: [‘gender equity’ AND ‘study’], [‘gender equity’ AND ‘study’ AND ‘Malmö’], [‘gender1 equity’ AND ‘study’ AND ‘women’], [‘gender equity’ AND ‘study’ AND ‘immigrant’], [[‘survey’ AND [‘gender’ OR ‘women’] AND [‘equity’ OR ‘equality’] AND [‘Sweden’ OR ‘Malmö’]], [‘survey’ AND ‘gender’ AND ‘immigrant’], [[‘gender’ AND [‘equity’ OR ‘equality’] AND Sweden]]. Only the first 100 search results from Google were examined (total 600). Relevant data such as education and employment statistics and official documents were retrieved from ‘Statistics Sweden’ (SCB), the ‘City of Malmö’ webpage, and through contact with city employees. The retrieved documents and statistics were selected according to relevance to each of the search questions and to Malmö, and broader inferences to Sweden were included in the absence of local ones.

The PEI evaluations can optimally be performed by many evaluators and with stakeholder feedback through surveys, interviews, and discussions (18); however, due to practical constraints it was only based on a literature review performed by two of the co-authors (GG and NN). The evaluation report was submitted to the ‘Commission for a Socially Sustainable Malmö’ and was included as an element in its final proposal (22, 23).

## Results

A total of 778 documents were retrieved and examined for relevance on Google and Google Scholar (600), PubMed (54), and WoS (124). After examination for relevance and repetition, 70 documents from Google, 30 from PubMed, and 61 from WoS (total 161) were used for the evaluation.

The complete evaluation and bibliography are presented in Appendix 1. The questions and evaluation scores are summarized in Table 1. Below we summarize the GEIDP evaluation findings:

### Constituent concern and participation

Q1. The GEIDP addresses the persistence of gender inequity in Malmö, for example, the inequities in employment, living conditions, education and health, power and influence in society, wages, expectations, unpaid work distribution and parental leave, sexual harassment, and gender-based violence. The evaluation suggests that between 50 and 90% of Malmö residents are currently concerned with gender inequality at home and work, and with associated issues such as gender-based violence.

Q2. Gender equity is of widespread concern in Sweden and internationally, and the agenda for this policy appears to have been set beyond the city level, although non-expert local constituents (e.g. party members) have raised the issue in Malmö. The EU and State roles in driving the issue toward the City Council and Board seem to dwarf the local community contributions.

Q3. This is a centrally conceived policy plan with significant but not determining peripheral feedback by local authorities (such as local councils), agencies (such as the educational, technical and cultural agencies) and interest groups (such as the teachers’ union), some of which had developed their own plans for gender equity. Q2 and Q3 could profit from further discussion and input from the policy-planners.

Q4. The plan is to a very large extent delegated to the peripheral authorities, agencies, departments and businesses for implementation. A number of city departments and agencies have already designed their adapted plans. However, the overall control, supervision and support are left to the central authority.

### Building constituent capacities and opportunities

Q5. The plan relies significantly and invests on education on gender equity issues among the City employees at all levels but mainly for coordinators and mention is made for education as a means of more equitable social integration. Gender education is targeted toward students of all levels, from primary school to university and leaders. However, no specific programs for targeted training in areas where women or men are lagging behind are planned although some especially vulnerable groups are supported for job seeking by publicly supported programs.

Q6. There is some direct creation of employment through this plan, mainly in its implementation in education on gender, gender sensitive budgeting and communication and in application of gender disaggregated statistics as well as, indirectly, through developmental benefits, private sector attitude change and fairer parental leave distribution. However, there is no explicit provision for extra jobs; therefore the impact on jobs seems unlikely to exceed 1% of the city’s employable residents (i.e. aged 16–64).

Q7. The plan calls directly for the responsibility of the City to support gender equity contact persons of peripheral administrations and companies through an existing website (malmo.se/jamstalldhet) and the city intranet (KomIn). Indirectly, it aims to place new emphasis on the issue, by spreading knowledge and serving as a model for wider equity in the society. The discussions and seminars are likely to improve the communication between the many groups concerned with gender equity, institutions, agencies and businesses active in Malmö and between those and the City. Given the large number of existing groups, it is unlikely that many new groups and networks will be formed because of the plan. Therefore, the proportion of constituents that the plan will indirectly motivate to participate in these links is unlikely to exceed 10% of the resident population.

Q8. Despite living in one of the world’s most equitable societies, many women in Sweden can still be considered disadvantaged and vulnerable. Seventy two percent of all leaders and 82% of board members of publicly listed companies are men. Only 16% of professors are women and a woman’s average salary is 8% lower than a man’s. The aggregate total income of women is 70% of men’s while they contribute threw times more in unpaid services. One in five women feels that she is being treated unequal at work and one in four is unhappy with gender equity in this country. There are over 25,000 crime reports, mostly by intimate partners, and around 6,000 rapes against women every year. Malmö is 37th among 290 municipalities on the SCB’s gender equity index (Jämindex), last among the major cities. Forty percent of its population has an immigrant background and 30% were actually born abroad. Immigrant women often face even more challenges toward employment and empowerment. Men are also disadvantaged in regards to parental leaves.

The plan calls directly for improvement in the both genders situation among the City’s employees first, and through the handling of public matters to all the gender-disadvantaged residents of Malmö, clearly more than 10% of the population.

Q9. Central (City Council) funding and resources (mainly human) are called for, which are most likely to be sufficient since significant funds have already been budgeted and allocated and the City currently has sufficient financial assets and budget commitments were made. Given the local and national academic environment’s focus on gender knowledge and human resources are also most likely to be available.

### Modes of policy evaluation

Q10. The plan calls for annual, gender disaggregated statistics of measurable targets and consequence reports from the city boards and administrations to be continuously created, and an annual revision update report and analysis will be presented by the city board. The City will be responsible for the overall follow-up of gender equity integration′s progress by the city administrations and also provide support and coordination (24), p. 15). In addition to the statistics the plan explicitly asks for qualitative equity analyses, in order to sample the ‘residents and users experiences through different kinds of surveys’ (24, p. 24). The City Council must approve changes in the plan. However, as adequate authority is delegated peripherally for implementation, several city departments and administrations have already formulated and adapted the plan to their situations so this is also likely to happen after the evaluations.

### Evaluation conclusions

After applying the index scores to these questions (see Table 1 and Appendix 1), a total score of 64% (46–78%) best describes the policy plan as ‘Supportive’ of empowerment and ranging between the ‘Supportive’ and ‘Enabling’ PEI policy categories (see Appendices 1 and 2).

The policy plan’s strong points according to the index are: 1) the wide constituent knowledge and concern with gender inequality (Q1); 2) the peripheral implementation by the city departments, administrations, agencies and companies (Q4); 3) the protection conferred to women and men against gender discrimination by the City as employer, and the push for support by the City agencies for a more equitable society and empowerment through employment, education and fairer distribution of resources and agency (Q8); and iv) the comprehensive evaluation and adaptation procedures in the city (Q10).

The plan scored average on the following points:

1. A centrally set policy agenda (Q2). Although initiated by grassroots movements, the gender equity agenda in Sweden is nowadays pushed forward by international and national agencies, outside the constituent communities. One central assumption of the PEI is that policies are more empowering when their constituents own them from their conception. Obviously the plan is part of a wider policy framework, which has sprung from population movements in the mid-1900s. However, the PEI here estimates its own empowering potential, and not that of the overall gender equity policy.
2. The planning was done mainly by City-office experts, with no game-changing peripheral and lay contributions (Q3). Active, wide and equitable constituent participation in policy planning may be effort- and time-consuming but has a potential to empower by delegating agency and influence, and through better responding to current and consensual community needs.
3. The plan’s provisions for education are focused on gender issues (Q5). Additional capacity development could stem from more explicit support to skill development to overcome proficiency inequalities.
4. Emphasis is placed on communication, but new network formation is limited (Q7). The existing networks could always benefit from more vivid and wide participation. Maximization of contacts and engagement of different views can empower the community toward coming up with innovative solutions and faster norms’ change, benefiting from Malmö′s cultural diversity.
5. Resources may be adequate, but more local, creative resource mobilization, e.g. advice for promotion of volunteer work, or even a community ‘inequality tax’ could generate local funds for equity programs, promoting local ‘ownership’ of the plan’s components (Q9).

According to this evaluation, an issue where the plan can be improved from empowerment’s perspective is on promotion of specific and targeted employment and entrepreneurship (Q6). The City has the capacity to promote equitable employment not only as an employer but also through its regulations, services and programs. It can be argued that unemployment levels in the city are relatively low. However, disadvantaged groups certainly exist, such as within ethnic, age groups, or with specific professional orientations, penal and addiction histories. Part time or temporary work is also problematic for sustainable career development, economic independence and empowerment.

## Discussion

We conducted a pilot evaluation of a Swedish gender equity plan using an empowerment index, based on diverse evidence sources, ranging from official documents, statistics and peer reviewed articles to published and electronic news and debates. Our aim was to provide feedback to the policymakers on the GEIDP as well as to test and develop the PEI for future policy evaluations for empowerment.

There is a clear need for a richer policy discourse and feedback on policy design (25). Public policies are most commonly evaluated after their implementation, whenever this is possible. This is easier for some policy types, such as monetary and health policies, where the impacts can often be measured. Few others have focused on various models of policy document content analysis according to predefined criteria, which apply best to gender equity policies (19–21). Thus, they have been able to compare gender equity provisions and gender mainstreaming among health policies. Such approaches are obviously valuable for policy discourse and development; however, more systematic and generally accepted and applicable, synoptic tools such as indexes should be developed to turn the art into science. To date, no evaluations of constituent empowerment by policies have been found other than our previous evaluation of the South African traditional medicine policy plan (18). That policy was found to be less empowering that the GEIDP [PEI score 42% (27–57)], mainly due to minor peripheral participation in the planning and implementation; however, comparisons among policies with such different scopes and contents using the PEI, although of interest from an overall empowerment perspective, are in need of further validation and standardization of the index and its methodology. We argue that empowerment of the constituents is important for democratic policy planning and that planning stage evaluations using standardized tools can contribute toward better-designed policies. The PEI is an effort in that direction.

This evaluation has significant limitations. Concessions were made in regards to the number of evaluators and the sampling of stakeholder responses due to time and staff availability constraints. The PEI evaluation can optimally be performed by three or more evaluators including members of the constituency, and combined with more extensive surveys.

However, an extensive review of both official and lay documents on the world’s broadest discussion forum, the World Wide Web, strengthens the validity of the findings. The use of both official and unofficial sources in the evaluation enriches the discourse about the plan, including sources that may be ignored by policymakers. We also communicated with a member of the policy-making team who responded to six out of the 10 PEI questions, suggesting a score of 80%, where our score for those questions was 66%. Similar feedback from several policy stakeholders could further validate our findings.

The PEI may be inadequate for comprehensive feedback to policy planners. However, it can be combined with existing and further developed socioeconomic, political, and health indicators such as the Gini income inequality index, OECD indicators (26), the gender equity index (Jämindex) (27), the Gender-related Development Index (GDI), the Gender Empowerment Measure (GEM), the Gender Gap Index (GGI), the Gender Status Index (GSI), The African Women’s Progress Scoreboard (AWPS), and the UNDP Gender Mainstreaming Scorecard (28, 29). More comprehensive indexes and indicators, for example, also measuring well-being and sustainability, are needed for a more holistic understanding of social welfare and progress (30).

Also, a single policy plan evaluation may be inadequate. Complementary plans and strategies may increase the overall policy’s empowerment potential. Therefore, a multi-sectorial policy ‘cluster’ evaluation is proposed as the next step.

## Conclusions

An empowerment evaluation of the Malmo City’s policy plan to increase gender equity in all aspects of city life showed that the plan has a strong potential to empower its constituency, being, according to the PEI terminology, ‘supportive’ and potentially even ‘enabling’ of empowerment’ with a PEI score of 64% (46–78%) (see Appendix 1). The plan’s main strong point according to this evaluation lies in the protection against gender discrimination in employment, education and distribution of resources and agency. Proposals that emerge from the analysis are toward proactive job creation for vulnerable groups and more delegated policy conception and planning.

Comprehensive policy evaluations can contribute toward a more participatory, community enriching and effective policy planning, facilitating the achievement of developmental and public health goals. The PEI can be a part of such evaluations.

## Appendix 1 Results of the Policy Empowerment Index Evaluation of the Malmö city’s Gender Equity Integration Development Plan

The full report can be found at the Malmö city’s web site: http://www.malmo.se/download/18.31ab534713cd4aa921379ea/Prooposal+inc+evalutation.pdf

Q1. How many policy-affected constituents are informed and concerned with the problem being addressed?

  0) No one

  1) <10%,

  2) 10–50%,

  3) 50–75%,

  4) 75–90%,

  5) 90–100%, of constituents

According to the evidence search:

The problem that the GEIDP addresses is the persistence of gender inequity in Malmö, for example, the inequities in employment, living conditions, education and health, power and influence in society, wages, expectations, unpaid work distribution and parental leave, sexual harassment and gender-based violence.

According to recent surveys, over 75% of Swedish men and women support a balanced distribution of unpaid work and salaries, and 86% of employees believe that gender salary differences are wide (18, 32), although the populace does not prioritize gender equity over other issues (33–35). Sweden is one of the most gender equal societies (36–38), despite differences in salaries and work positions (24, 39–45), unpaid homecare and parental leave (46–57), and in healthcare (58, 59). Women are concerned about unequal opportunities and one in five women feels gender-discriminated at work (60–62). Around 60% of both men and women want a fairer distribution of parental leave (63).

Many Malmö residents are concerned about gender equity, and gender issues feature in basic and higher education curricula (64–66); however, no local surveys were found. Malmö has a higher percentage of residents with foreign background than the rest of Sweden: 30% born abroad, 41% with foreign background (20). Immigrants may have different views from natives on gender equity, but, according to global surveys, a large proportion of them are expected not only to know and be concerned but also to agree that men and women should have equal rights (60–97%), although fewer people globally believe that there is more to do about gender equity (from 35% in Egypt to 81% in France) (67, 68).

The GEIDP addresses the lack of gender equity in Malmö, beginning with safeguarding equal opportunities, conditions and rewards by the city as employer, and through the city departments′, administrations′ and companies′ services and distribution of resources (17).

In the absence of recent local statistics on Malmö resident views on gender equity, the above evidence suggests that between 50 and 90% of Malmö residents are currently concerned with gender inequality at home and work, and with associated issues such as gender-based violence, which the GEIDP aims to address. A local sampling of opinion, such as through the PSA survey, can be useful to narrow the confidence interval. *PEI Score: 3.5 (range 3–5)*

Q2. How was the political agenda set? Did the plan in question start from a discourse in the community and local grassroots movement advocacy, or by professional experts and politicians at the City level and above (or both in interaction)?

By:

  0) Some non-legitimate leader/authority, non-transparently

  1) experts, centrally

  2) experts, multi-level or peripherally

  3) mixed (experts and lay stakeholders), centrally

  4) mixed, multi-level

  5) mixed, peripherally

According to the evidence search:

Gender Equity was added to Swedish political agenda after the economic transformation and the global feminist movement (1960s), and gained political support in the 1970s (69, 70).

The current plan is a response to international guidance (71) and more specifically an EU proposal signed by national representatives (12, 72). Although many local, national and international groups with local representation and political party branches have gender equity high in their agenda (37, 71, 73–84), a relative delay in implementation in Malmö may be the result of satisfaction with the current situation and maybe even some controversy on gender equity’s social and health effects (34, 35, 63, 85–92). An interesting report suggests that evolution of family gender norms has lagged behind policy makers’ ideas (63). On the contrary, significant evidence was found for a national and international agenda for gender equity, supported by human rights, macroeconomic and macro-demographic priorities (91, 93–104).

The gathered evidence therefore suggests that gender equity is of widespread concern in Sweden and internationally (see also Q1) and thus the agenda for this policy appears to have been set beyond the city level, although non-expert local constituents (e.g. party members) have raised the issue in Malmö. The EU and State roles in driving the issue toward the City Council and Board seem to dwarf the local community contributions. However, this conclusion can be contested by survey input and further discussion with the policy stakeholders. *PEI Score: 3 (2–4)*

Q3. How was the policy planned? Did peripheral agencies and interest groups contribute significantly to the planning?

The plan/policy was drafted by:

  0) Some non-legitimate leader/authority, non-transparently

  1) experts, centrally

  2) experts, multi-level or peripherally

  3) mixed (experts and lay stakeholders), centrally

  4) mixed, multi-level

  5) mixed, peripherally

According to the evidence search:

The planning was done at the City of Malmö (105) after a stakeholder opinion evaluation and significant contributions from peripheral agencies and stakeholders, such as the community councils, the educational, technical and cultural agencies, the local house construction company (MKB) and unions (such as the teachers’) among others, through meetings and written responses to a plan draft (106).^1^

However, the planners were city employees with experience in gender issues, thus considered experts, without lay community stakeholders among them. The peripheral feedback was significant but not game-changing for the plan’s final formulation (25, 107). The plan firmly complies with the pre-existing city plan, the EU charter and the Swedish government and agencies’ guidance (12, 72, 107–111). No evidence of significant Malmö or other residents’ or agencies’ participation was found in those documents.

In addition, peripheral city departments appear to have followed the city in their planning instead of contributing their own plan proposals to the final plan formulation (112–118), although one department had already formulated a gender equity plan based on the previous guidance (113) and another had significant relevant experience (114). This issue, together with Q2 can profit from further discussion and input from policy-planners. *PEI Score: 2.5 (1–3)*

Q4. What percentage of the development plan actions was delegated peripherally for implementation? Will the plan be implemented mainly by the central or peripheral City authorities?

  0) No delegation

  1) <10%

  2) 10–50%

  3) 50–75%

  4) 75–90%

  5) 90–100%

According to the evidence search:

The plan is to a very large extent delegated to the peripheral authorities, agencies, departments and businesses for implementation (17, pp. 8, 9, 13, 14, 15), especially in regard to the city services. Only a few of its measures will be implemented at the central city level, and those mainly concern the City working environment (17, p. 8).

The City is employing less than 10% of Malmö’s active workforce, total workforce or population (23, 119–122). This proportion can be used as an indicator of the magnitude of central vs. peripheral implementation needed for the measures required by the City as an employer, e.g. education–training, recruitment and overall adaptation of the working environment.

However, overall control, supervision and support remain with the central authority, the City Board and Council (17, pp. 10, 14). The municipal boards, agencies and companies will be equipped with one or more contact persons coordinating implementation and follow-up of the plan (17, pp. 13, 14) while leaders at all levels are held responsible for the implementation (17, p. 15).

A number of city departments and agencies have already designed their adapted plans (112–114, 116, 117, 119, 123). *PEI Score: 5 (4–5)*

Q5. Does the policy plan call for education/training of the constituents?

  0) <1%

  1) 1–10%

  2) 10–50%

  3) 50–75%

  4) 75–90%

  5) >90%

According to the evidence search:

The plan relies significantly on education on gender equity issues among the City employees, at all levels but mainly coordinators and leaders (17, pp. 7, 10–12, 17, 25) and mention is made for education as a means for more equitable social integration (17, p. 19).

Commitment for advancement of gender equity can be found in the last City budget (124, Q9). The City department of education has already formulated its equity plan, with a significant number of activities for City employees, leaders and support to schools by gender educators, and more such personnel with experience in gender issues, gender equitable budget work and communication, is sought by public announcements (123, 125). In 2011, 9,000 employees were trained through role-plays at work, and 141 people were trained as play leaders ((122, 123) p. 13) on diversity issues. The total number of employees on December 31st 2011 was 20.521, increased by 545 from the previous year and the City had an annual employee turnover of 1560 (122, pp. 8, 10). If similar numbers are maintained, a little over 40,000 city employees may be employed and, according to the plan’s objectives receive some kind of training for gender equity over the plan’s implementation decade, not counting potential interns (900 in 2011) and day-workers. This accounts for approximately 18% of residents aged 6–64 (see footnote 1) (23, 126).

Regarding the plan’s provisions for the City as a service provider, evidence was found that gender education targeted toward students of all levels, from primary school to university (127–130), city employees (122, 130, 131) and leaders (125, 131), is already advancing. Clearly education on gender issues is needed to address norms that hinder gender equity at home and work (55, 132–134) and to protect women from violence and harassment (134). Even in public educational institutions, study materials (133), training programs (135, 136), and educators’ knowledge and attitudes can improve (126). The city′s population enrolled in educational programs in 2010–11 amounted to 27,328 aged 6–15 and 34,911 aged 16–64, totaling 62,239, or 27.3% of total residents aged 6–64 (see footnote 1) (23, 126, 136–139).

The question arises if more education and training can increase population empowerment in Malmö. There evidently exists a high degree of gender equity regarding both on-going education and education levels in Malmö and overall in Sweden (126, 136, 139), but significant differences exist at different areas of training, with higher proportions of men in technical positions and lower in care trainings, and a lower proportion of women in research (139). Women are also lagging behind in top job positions and entrepreneurship (39, 40, 139) and some ethnic groups of women have very low levels of official employment (24). It is thus obvious that some more targeted training (such as leadership management and entrepreneurship courses for women) and an overall correction of these differences could widen the career opportunities for significant additional population numbers and thus empower the Malmö community. However, no specific programs for targeted training in areas where women or men are lagging behind are planned (17, 123), although some especially vulnerable groups are supported for job seeking by publicly supported programs (140). Only around 17.4% of residents aged 16–64 are receiving education at the moment (23, 126, 136–139).

Thus, mainly employees and current students are eligible to receive some extra education by the plan before 2020, which accounts for around 46% of the total that is eligible to study resident population (see footnote 1) (2011). *PEI Score: 2 (1–3)*

Q6. Are peripheral employment and entrepreneurship being strengthened? Will the plan create jobs or business opportunities for women or men?’

  0) No

  1) employment/entrepreneurship for <1%

  2) employment for 1–10%

  3) employment for >10%

  4) some entrepreneurship and self-employment for 1–10%

  5) broad entrepreneurship and self-employment for >10%

According to the evidence search:

There is some direct creation of employment through this plan, mainly in its implementation.

The plan aims to eliminate gender discrimination by raising awareness on gender issues among employees and leaders, controlling unequal recruitment and remuneration, and promoting equal employment conditions and opportunities (17, p. 8) as well as guaranteeing equitable activities, services, treatment, exercise of public authority, distribution of resources, power and influence (17, p. 7). Leaders and employees will be educated on gender, gender sensitive budgeting and communication and gender disaggregated statistics and reporting will be implemented (17, p. 8–11). Educators with experience in gender issues will thus be needed and relevant provisions have already been made (123, 125). Given the large number of employees and occupations (124, p. 2), these tasks will probably require a significant amount of extra work-time.

Extra funds are earmarked for gender equity in the 2012 budget (23, pp. 26, 48). All administrations and municipal companies will assign at least one contact person responsible for the equity integrations coordination (17, p. 13). There are 10 peripheral administrations and 14 municipal companies, so this requirement could create a few new jobs. However, no explicit provision for extra jobs was found in the current budget and revision of personnel (23, 124). In any case, new educators and coordinators together will not account for more than 0.5% of the eligible to work resident population of 201,123 aged 16–64 (see footnote 1) (137, 138).

Additional employment and entrepreneurship may be created by facilitating access to ‘work, housing, culture, information–communication as well as social and medical help’ (17). Although to a large extent these services are already in place, more targeted activities are well needed (131, 140, 141) and some programs targeting young or marginalized women have already been put in place (39, 142). However, in the absence of an explicit call for job creation, the impact of such programs is expected to be proportionally low.

The plan is also unlikely to create new jobs through its goal for an equitable balance of recruitment and employment. Among the city’s 20,521 non-day-worker employees, 77.2% were women and 22.8% were men, with women also occupying more leadership positions (124. pp. 12). Sweden is now one of the few European countries with increasing gender desegregation in its labor, though a decade ago was regarded a high-employment and high-gender-segregated country in EU (143, p. 18). However, the European labor market analysis of gender segregation reports that there is a gender polarization of jobs where women, although with a higher employment rate, are more associated with ‘feminized’ jobs relating to teaching, care giving, etc., while men are involved in computing and technical jobs (143, p. 8). This could contribute to narrow skill development, labor supply and resultant biased employment opportunities (143, p. 43). This gender imbalance in favor of women implicitly serves to balance inequalities in favor of men in the private sector (40–46, 60, 61, 144, 145), and some ‘structural’ work inequalities in Swedish society (such as in leadership roles, gender biased training, work orientations and unpaid home care) (46–51, 55) and thus a significant change seems unlikely. In any case, any new positions for one sex will be at the cost of the other, so no new jobs can be thus created.^2^

There are four other ways through which this plan may increase employment:

1. More generous parental leave regulation by the City (17, p. 18) will create gaps to be filled with part or full-time workers (128, 146)
2. Academic programs and research (17, p. 11, 128, 147) can create jobs and careers.
3. Gender equity has been shown to enhance growth in the long-term (98, 100, 148, 149), which in turn may create jobs and business opportunities.
4. The private sector may follow the city in providing education on gender, more generous parental leaves and better balanced recruitment and employment. Evidence was found of a good corporate predisposition toward gender equity in Sweden and the province of Scania (Skåne) where Malmö is the capital (60, 145, 149, 150), and research on favorable impact on workers′ physical and mental health may further support this trend (57, 151).

However, given the absence of a direct call for job creation, the overall contribution of the plan on employment is very unlikely to exceed 1% of the city′s 201,123 residents (see footnote 1) aged 16–64 (24, 137, 138), of whom over 40% (2010) are not employed (23, 24, 39, 40, 131, 137, 138, 140, 141).^3^ *PEI Score: 1 (1–2)*

Q7. Does the plan promote constituent participation in horizontal and vertical networks? Does the plan create links between the community members/citizens/residents and between them and the authorities of the city?

Promotion of networking of constituents:

  0) No

  1) all for <1%

  2) indirect for 1–10%

  3) indirect for >10%

  4) explicit/direct for 1–10%

  5) explicit/direct for >10%

According to the evidence search:

The plan calls directly for the responsibility of the City to support gender equity contact persons of peripheral administrations and companies through networking. This will happen through personal communication, an existing website (malmo.se/jamstalldhet) and the city intranet (KomIn) (17, p. 13). In this way, the existing horizontal and vertical networks of the City will be enhanced for the coordinators and leaders involved in the project which will probably not account for more than a few hundred constituents (less than 1% of the constituency) (23).

Indirectly, GEIDP aims to put new emphasis on the issue, by spreading knowledge and serving as a model for wider equity in the society. The discussions and seminars are likely to improve the communication between the many groups concerned with gender equity, institutions, agencies and businesses active in Malmö and between those and the City. Around 100 women’s and men′s groups actively pursue gender equity in Scania, with over 10,000 local members (23, 73–75, 77). Although this could include duplicate memberships, the number may easily exceed 1% of the constituency (3,000 members).

Given the large number of existing groups, it is unlikely that many new groups and networks will be formed because of the plan. Therefore, the proportion of constituents that the plan will indirectly motivate to participate in these links is unlikely to exceed 10% of the resident population (30,000 members) (23, Q5). More accurate statistics of residents involved in groups and networks in Malmö would be useful in this regard. *PEI Score: 2 (1–3)*

Q8. Are hard to reach, vulnerable or disadvantaged populations being considered and affirmatively protected and empowered (including vulnerable gender and age groups, socially/physically/economically disadvantaged individuals, groups and communities)?

  0) No

    Yes,

  1) some for <1%

  2) indirect for 1–10%

  3) indirect for >10%

  4) explicit/direct for 1–10%

  5) explicit/direct for >10%

Despite living in one of world’s most equitable societies, many women in Sweden can still be considered disadvantaged and vulnerable. Seventy two percent of all leaders and 82% of board members of publicly listed companies are men. Only 16% of professors are women and a woman’s average salary is 8% lower than a man’s. The aggregate total income of women is 70% of men’s while they contribute three times more in unpaid services (55, 152). One in five women feels she is being treated unequal at work and one in four is unhappy with gender equity in this country (60, 62). There are over 25,000 crime reports, mostly by intimate partners, and around 6,000 rapes against women every year (55).

Malmö is 37th among 290 municipalities on the SCB’s gender equity index (Jämindex), last of the major cities (28). Some recent prominent crimes have raised public attention to violence against women (153–157). Forty percent of its population has an immigrant background and 30% has actually been born abroad (23). Immigrant women often face even more challenges toward employment and empowerment (24).

Some men are also disadvantaged. Only 22% of parental leaves go to men (18, 55), who are also more likely to spend holidays away from family (146). Some feel unhappy with the state’s ‘affirmative action’ for gender equity, and demand same ‘game rules’ instead of equitable ones (63).

The plan calls directly for improvement in the both genders situation among the City’s employees first, and through the handling of public matters to all the gender-disadvantaged residents of Malmö, clearly more than 10% of the population (122, Q5). *PEI Score: 5 (4–5)*

Q9. Does the policy provide for or will there most likely be adequate financial, human and other resources?

  0) no provision

  1) inadequate, central

  2) inadequate, mixed or peripheral

  3) adequate, central

  4) adequate, mixed (central and peripheral)

  5) adequate, peripheral

According to the evidence search:

Central vs. Peripheral: According to the plan, the City Council has the overall responsibility for the funding of the plans goals by the annual budget (17, p. 14). The peripheral administrations and boards are made responsible to allocate budget and other resources for their own gender equity targets and instructions are given for gender budgeting (17, pp. 14, 23) but the funds are provided by the City budget (124, p. 47).

There is no provision for peripheral mobilization of funds for the implementation and follow up of the integration of gender equity at the city departments and agencies. However, the peripheral administrations and companies are asked to assign contact persons responsible for the coordination, and educators will be needed for the necessary training on gender issues (see Q5 and 6). According to the City′s recruitment report, many of these human resources are recruited among local young people and minorities (122, p. 5). No mention is made for any prioritization of members of the recruiting agencies’ communities, where in any case it may be difficult to find the expertise needed for these positions (125).

In conclusion, the plan calls mainly for central financial and human resources.

Sufficient vs. insufficient: Significant funds have already been allocated to advance the plan’s aim for education (25, 125, 128, 130, 147). Malmö City has sufficient financial assets to fund this plan (124, p. 61) and relevant commitments are made in the 2012 budget document (124, p. 8, 24, 37). Given the current economic situation and political will (80–82, 130, 158) in Malmö, funds and fixed assets are thus likely to be sufficient. Given the local and national academic environment′s focus on gender issues (65, 66), knowledge and human resources are also most likely to be available. *PEI Score: 3 (2–4)*

Q10. How will the policy plan be evaluated and adapted?

  0) By some non-legitimate leader/authority, non-transparently

  1) no plan

  2) evaluated and adapted centrally, quantitative methodology

  3) evaluated and adapted centrally, quantitative/qualitative methodology

  4) evaluated and adapted centrally and peripherally, quantitative methodology

  5) evaluated and adapted centrally and peripherally, quantitative/qualitative and/or participatory methodology

According to the evidence search:

The plan calls for annual, gender disaggregated statistics of measurable targets and consequence reports from the city boards and administrations to be continuously created, and an annual revision update report and analysis will be presented by the city board (25, p. 14). The City will be responsible for the overall follow-up of gender equity integration′s progress by the city administrations and also provide support and coordination (25, p. 15). In addition to the statistics, the plan explicitly asks for qualitative equity analyses, in order to sample the ‘residents and users experiences through different kinds of surveys’ (25, p. 24).

In addition, the importance of both quantitative and qualitative aspects are raised in the national policy document on gender equity (16, p. 86) and previous City, State and private evaluations of the gender equity situation have included both qualitative and quantitative methods (159–162), and the same applies for local and national related academic studies (64). Thus, it can be assumed with relative certainty that the plan will be evaluated holistically.

The City Council must approve changes in the plan (25, p. 14). However, as adequate authority is delegated peripherally for implementation, several city departments and administrations have already formulated and adapted the plan to their situations (112–117), so this is also likely to happen after the evaluations. *PEI Score: 5 (4–5)*

Total score: 32 (23–39) or 64% (46–78%). The policy plan can be best described as supportive of empowerment, ranging between the supportive and enabling PEI policy categories.

**Complete Evaluation Report Bibliography**

1. World Health Organization (WHO) (2012). Women and gender equity. Available from: http://www.who.int/social_determinants/themes/womenandgender/en/index.html [cited 5 August 2012].
2. Sen G, Östlin P, George A. Unequal, unfair, ineffective and inefficient – gender inequity in health: why it exists and how we can change it Report of the Women and Gender Equity Knowledge Network of the Commission on Social Determinants of Health; 2007.
3. Fung A, Wright E. Deepening democracy: innovations in empowered participatory governance. Politic Soc 2001; 29: 5–41.
4. Pratchett L, Durose C, Lowndes V, Smith G, Stoker G, Wales C. Empowering communities to influence local decision making: a systematic review of the evidence. London: Department for Communities and Local Government 2009.
5. Kabeer N. Resources, agency, achievements: reflections on the measurement of women’s empowerment. Dev Change 1999; 30: 435–64.
6. Kim J, Paul P, Barnet T, Watts C. Exploring the role of economic empowerment in HIV prevention. AIDS 2008; 22: 57–71.
7. Woodall J, Raine G, South J, Warwick-Booth L. Empowerment and health & well being: evidence review. Leeds: Centre for Health Promotion Research, Leeds Metropolitan University; 2010.
8. Briones-Vozmediano E, Vives-Cases C, Peiro-Perez R. Gender sensitivity in national health plans in Latin America and the European Union. Health Policy 2012; 106: 88–96.
9. Stewart DE, Dorado LM, Diaz-Granados N, Rondon M, Saavedra J, Posada-Villa J, et al. Examining gender equity in health policies in a low- (Peru), middle- (Colombia), and high- (Canada) income country in the Americas. J Public Health Policy 2009; 30: 439–54.
10. Garcia-Calvente MM, Castano-Lopez E, Mateo-Rodriguez I, Maroto-Navarro G, Ruiz-Cantero MT. A tool to analyse gender mainstreaming and care-giving models in support plans for informal care: case studies in Andalusia and the United Kingdom. J Epidemiol Community Health 2007;61(Suppl 2): ii32–38.
11. European Commission (EC) (2007). Report on equality between women and men – 2007. Luxembourg: Office for Official Publications of the European Communities.
12. Council of European Municipalities and Regions and its Partners (2006). The European charter for equality of women and men in local life.
13. United Nations Development Programme (UNDP) (1995). Human Development Report. Available from: http://hdr.undp.org/en/reports/global/hdr1995/chapters/ [cited 8 June 2012].
14. Central Intelligence Agency (CIA) (2012). The World Factbook. Available from: https://www.cia.gov/library/publications/the-world-factbook/rankorder/2172rank.html [cited 8 June 2012].
15. United Nations Development Programme (UNDP) (2011). Human Development Report 2011. Sustainability and equity: a better future for all.
16. Statens Offentliga Utredningar (SOU 2005:66). Makt att forma samhället och sitt eget liv – jämställdhetspolitiken mot nya mål. Slutbetänkande av Jämställdhetspolitiska utredningen. Stockholm; 2005.
17. Malmö Stad (2011). Utvecklingsplan för jämställdhetsintegrering.
18. TNS-SIFO Surveys (2001). Jämställdhet. Available from: http://www.tns-sifo.se/rapporter-undersokningar/senaste-undersokningarna/2001/jaemstaelldhet-/ [cited 5 August 2012].
19. Malmö Stad (2012). Malmöfakta. Available from: http://malmo.se/Kommun--politik/Om-oss/Statistik-om-Malmo.html [cited 7 August 2012].
20. Malmö Stad (2012). Malmöbor med utländsk bakgrund. Available from: http://www.malmo.se/download/18.6e1be7ef13514d6cfcc800030664/Malm%C3%B6bor+med+utl%C3%A4ndsk+bakgrund.pdf [cited 25 July 2012].
21. Gavriilidis G, Östergren P. Evaluating a traditional medicine policy in South Africa: phase 1 development of a policy assessment tool. Glob Health Action 2012; 5: 17271.
22. Stigendal M, Östergren P-O, editors. Malmö’s path towards a sustainable future: health, welfare and justice. Commission for a socially sustainable Malmö. Malmö: Commission for a socially sustainable Malmö; 2013.
23. Malmö Stad (2012). Aktuellt om: Malmös befolkningsutveckling 2011 Stadskontoret.
24. Borg A. Dagen. Sekulär stat inte alltid bäst; 2012. Available from: http://www.dagen.se/nyheter/borg-sekular-stat-inte-alltid-bast/ [cited August 2012].
25. Malmö Stad (2012). Framsteg i arbetet för ett jämställt Malmö. Available from: http://www.malmo.se/Nyheter/Centrala/3-21-2012-Framsteg-i-arbetet-for-ett-jamstallt-Malmo.html [cited 5 August 2012].
26. Sanderson I. Evaluation, policy learning and evidence-based policy making. Publ Admin 2002; 80: 1–22.
27. United Nations Development Programme (UNDP) (2012). Human Development Reports, Indices and Data OECD. Available from: http://hdr.undp.org/en/statistics/indices/gdi_gem/ [cited 7 August 2012].
28. SVT.se (2006). Jämindex – Hela Landet (SvT). Available from: http://www.svt.se/content/1/c6/67/88/72/jamindexhelalandet.pdf [cited 4 August 2012].
29. Organisation for Economic Co-operation and Development (OECD) (2012). Gender indicators: what, why and how?; Available from: http://www.oecd.org/social/genderequalityanddevelopment/43041409.pdf [cited 7 August 2012].
30. Spinakis A, Anastasiou G, Panousis V, Spiliopoulos K, Palaiologou S, Yfantopoulos J. Expert review and proposals for measurement of health inequalities in the European Union – Full Report. European Commission Directorate General for Health and Consumers. Luxembourg; 2011. Available from: http://ec.europa.eu/health/social_determinants/docs/full_quantos_en.pdf [cited 7 August 2012].
31. Stiglitz J. The Stiglitz Report: reforming the international monetary and financial systems in the wake of the global crisis, with members of the commission of experts of the President of the United Nations General Assembly on Reforms of the International Monetary and Financial System; 2012.
32. Landsorganisationen i Sverige (2009). Ny LO-undersökning – kraftigt stöd för jämställda löner. Available from: http://www.lo.se/home/lo/home.nsf/unidView/EF9F61E9223FC5AAC125728A002BDA58 [cited 5 August 2012].
33. Åsblad M. Dagensarena. Svenskar vill ha jämställdhet – men väljer det inte; 2012. Available from: http://www.dagensarena.se/innehall/svenskar-vill-ha-jamstalldhet-men-valjer-det-inte [cited August 2012].
34. Aftonbladet (2010). Bara två procent tycker jämställdhet är viktigt. Available from: http://www.aftonbladet.se/wendela/article12285311.ab [cited 5 August 2012].
35. Tro och politik. Sista ordet: Dags att ta fajten om jämställdheten. October 2011. Available from: http://troochpolitik.se/?p=212031 [cited 5 August 2012].
36. Jakobsson N, Kotsadam A. Do attitudes toward gender equality really differ between Norway and Sweden? J Eur Soc Policy 2010; 20: 142–59.
37. Kvinnojouren (2012). Jämställdhet i Sverige. Available from: http://www.kvinnojouren.se/fakta/feminism-och-jamstalldhet/jamstalldhet-i-sverige [cited 5 August 2012].
38. Adman P. Investigating political equality: the example of gender and political participation in Sweden. Acta Politica 2011; 46.
39. Statistiska Centralbyrån (SCB) (2012). Entreprenörskap och företagande. Available from: http://www.scb.se/Pages/ThematicTables.aspx?id=334633 [cited August 2012].
40. Statistiska Centralbyrån (SCB) (2012). Ekonomisk jämställdhet. Available from: http://www.scb.se/Pages/ThematicTables.aspx?id=334771 [cited 5 August 2012].
41. Carlsson-Kanyama A, Julia I, Rohr U. Unequal representation of women and men in energy company boards and management groups: are there implications for mitigation? Energ Pol 2010; 38: 4737–40.
42. Seierstad C, Healy G. Women’s equality in the Scandinavian academy: a distant dream? Work Employ Soc 2012; 26: 296–313.
43. Hearn J, Nordberg M, Andersen K, Balkmar D, Gotzen L, Klinth R, et al. Hegemonic masculinity and beyond: 40 years of research in Sweden. Men Masc 2012; 15: 31–55.
44. Lindberg L, Riis U, Silander C. Gender equality in Swedish higher education: patterns and shifts. Scand J Educ Res 2011; 55: 165–79.
45. Sainsbury D, Bergqvist C. The promise and pitfalls of gender mainstreaming the Swedish case. Int Fem J Polit 2009; 11: 216–34.
46. Enberg B, Stenlund H, Sundelin G, Ohman A. Work satisfaction, career preferences and unpaid household work among recently graduated health-care professionals – a gender perspective. Scand J Caring Sci 2007; 21: 169–77.
47. Tunberger P, Sigle-Rushton W. Continuity and change in Swedish family policy reforms. J Eur Soc Policy 2011; 21: 225–37.
48. Thomas J, Hildingsson I. Who’s bathing the baby? The division of domestic labour in Sweden. J Fam Stud 2009; 15: 139–52.
49. Thornqvist C. Family-friendly labour market policies and careers in Sweden – and the lack of them. Symposium on Critical Perspectives on Careers and Family-Friendly Policies. Br J Guid Counsell 2006; 34: 309–26.
50. Rothstein B. The reproduction of gender inequality in Sweden: a causal mechanism approach. Gender Work Organ 2012; 19: 324–44.
51. Bernhardt E, Noack T, Lyngstad T. Shared housework in Norway and Sweden: advancing the gender revolution. J Eur Soc Policy 2008; 18: 275–88.
52. Olah L, Bernhardt E. Combining childbearing and gender equality. Demogr Res 2008; 19: 1105–44.
53. Langvasbraten T. A Scandinavian model? Gender equality discourses on multiculturalism. Soc Polit 2008; 15: 32–52.
54. Ahlberg J, Roman C, Duncan S. Actualizing the ‘Democratic family’? Swedish policy rhetoric versus family practices. Soc Polit 2008; 15.
55. Statistiska Centralbyrån (SCB) (2010). Jämställdhetsstatistik. På tal om kvinnor och män. Lathund om jämställdhet.
56. Kotsadam A, Finseraas H. The state intervenes in the battle of the sexes: causal effects of paternity leave. Soc Sci Res 2011; 40: 1611–22.
57. Norström L, Lindberg L, Månsdotter A. Could gender equality in parental leave harm off-springs’ mental health? A registry study of the Swedish parental/child cohort of 1988/89. Int J Equity Health 2012; 11: 11–19.
58. Jonsson P, Schmidt I, Sparring V, Tomson G. Gender equity in health care in Sweden – Minor improvements since the 1990s. Health Policy 2006; 77: 24–36.
59. Haglund B, Köster M, Nilsson T, Rosén M. Inequality in access to coronary revascularization in Sweden. Scand Cardiovasc J 2004; 38: 334–9.
60. Manpower (2011). Undersökning Work Life om jämställdhet i Norge och Sverige: Var fjärde kvinna missnöjd med jämställdheten. Available from: http://www.manpower.se/mpnet3/Content.asp?NodeID=57728&ref=SWEDEN_NORDIC [cited 18 July 2012].
61. Ekonomi Sverige (2011). Hur jämställda är vi? Available from: http://www.ekonomisverige.se/nyheter/hur-jamstallda-ar-vi/ [cited 18 July 2012].
62. Jämställ.nu (2012). Var femte kvinna har någon gång känt sig könsdiskriminerad på arbetsplatsen. Available from: http://www.jamstall.nu/aktuellt_2/nyhetsarkiv/maj/var-femte-kvinna-har-nagon-gang-kant-sig-konsdiskriminerad-pa-arbetsplatsen. [cited 15 July 2012].
63. Pelle Billing. Pelle Billing; 2012. Available from: http://www.pellebilling.se/ [cited 7 July 2012].
64. Uppsatser.se (2012). Sökning: “Undersökning jämställdhet Malmö.” Available from: http://www.uppsatser.se/om/unders%C3%B6kning+av+j%C3%A4mst%C3%A4lldhet+Malm%C3%B6/ [cited 29 July 2012].
65. Lund University (2012). Centre for Gender Studies. Available from: http://www.genus.lu.se/o.o.i.s/4737 [cited August 2012].
66. Malmö University (2012). Research themes. Available from: http://www.mah.se/english/Schools-and-faculties/Faculty-of-Culture-and-Society/DepartmentsSchools/Department-of-Global-Political-Studies/Research/Research-themes/ [cited August 2012].
67. Pew Research Center (2010). Pew global attitudes project. Gender equality Universally Embraced, but inequalities acknowledged. Available from: http://www.pewglobal.org/2010/07/01/gender-equality/ [cited 18 July 2012].
68. Kohei Y. Gender perceptions in Southeast Asian countries: findings from JICA-RI value surveys. World Development Report 2012. Gender Equality and Development, background paper; 2011; 2012.
69. Feministen (2012). Jämställdhetshistoria-Jämo. Available from: http://www.feministen.se/a/jamstalldhetshistoria-jamo [cited 8 August 2012].
70. Sveriges kvinno- och tjejjourers riksförbund (SKR) (2011). Historiska årtal i den svenska jämställdhetens historia. Available from: http://www.kvinnojouren.se/fakta/feminism-och-jamstalldhet/historiska-artal [cited 7 August 2012].
71. United Nations Development Programme Sweden (2012). TEMA: Jämställdhet och kvinnors rättigheter. Available from: http://www.undp.se/kvinnors-rattigheter/ [cited 10 August 2012].
72. Commission of the European Communities (2011). A Roadmap for equality between women and men, 2006–2010.
73. Interenationella Kvinnoföreningen i Malmö (IKF) (2012). Lokalt resurcentrum för kvinnor i Öresundsregion. Available from: http://www.ikf.se/ [cited 7 August 2012].
74. Femcenter (2012). Centrum för kvinnor utsatta för våld. Available from: http://www.femcenter.se/ [cited 5 August 2012].
75. Sveriges Kvinnolobby (2012). Nyheter från European Women’s Lobby. Available from: http://www.sverigeskvinnolobby.se/ [cited 15 August 2012].
76. Samarbeta (2012). Samarbeta. Available from: http://samarbeta.org/ [cited 5 August 2012].
77. Kvinnor nätverk I Skåne (2012). ResursCentrum för kvinnor i Skåne. Available from: http://www.skane.se/upload/Webbplatser/R_Centrum/Dokument/N%C3%A4tverkskatalogn%C3%A4tet30aug05.pdf [cited 7 August 2012].
78. Män för jämställdhet (2012). Malmö. Available from: http://www.mfj.se/malmo [cited 7 August 2012].
79. Ladyfest (2012). Bokare har stort ansvar för jämstäldhet. Available from: http://www.ladyfest.se/malmo/2012/07/13/bokare-har-stort-ansvar-for-jamstalldhet/ [cited 5 August 2012].
80. S-kvinnor i Skånedistriktet (2012). Socialdemokraterna. Available from: http://www.s-info.se/association/default.asp?id=404 [cited 7 August 2012].
81. Nya Moderaterna Malmö (2012). Jämställdhet. Available from: http://xn--malmmoderaterna-ctb.se/jamstalldhet-3/ [cited 7 August 2012].
82. Kristdemokraterna (2012). Jämställdhet. Available from: http://www.kristdemokraterna.se/varpolitik/politikomraden/jamstalldhet [cited 8 August 2012].
83. The Swedish Confederation for Professional Employees (TCO) (2012). Jämställdhet. Available from: http://www.tco.se/Templates/Page2____706.aspx [cited 10 August 2012].
84. Actionaid (2012). Jämställdhet Available from: http://www.actionaid.se/omraden/kvinnor_och_flickor/ [cited 4 August 2012].
85. Sörlin A, Lindholm L, Ng N, Öhman A. Gender equality in couples and self-rated health – a survey study evaluating measurements of gender equality and its impact on health. Int J Equity Health 2011; 10: 37.
86. Westin M, Westerling R. Health and healthcare utilization among single mothers and single fathers in Sweden. Scand J Public Health 2006; 34: 182–9.
87. Galtry J. The impact on breastfeeding of labour market policy and practice in Ireland, Sweden, and the USA. Soc Sci Med 2003; 57: 167–77.
88. Floderus B, Hagman M, Aronsson G, Marklund S, Wikman A. Work status, work hours and health in women with and without children. Occup Environ Med 2009; 66: 704–10.
89. Floderus B, Hagman M, Aronsson G, Gustafsson K, Marklund S, Wikman A. Disability pension among young women in Sweden, with special emphasis on family structure: a dynamic cohort study. BMJ Open 2012; 2.
90. Sörlin A, Ohman A, Lindholm L. Sickness absence in gender-equal companies: a register study at organizational level. BMC Public Health 2011; 11: 548.
91. Östlin P, Eckermann E, Mishra U, Nkowane M. Gender and health promotion: a multisectoral policy approach. Health Promot Int 2006; 21: 25–35.
92. Backhans M, Lundberg M, Månsdotter A. Does increased gender equality lead to a convergence of health outcomes for men and women? A study of Swedish municipalities. Soc Sci Med 2007; 64: 1892–903.
93. Lister R. A Nordic Nirvana? Gender, citizenship, and cocial justice in the Nordic Welfare States. Social politics. Conference of the Nordic-Council-of-Ministers. May, 2006. Oslo, Norway. Nordic Council Ministers. Soc Polit 2009; 16: 242–78.
94. Forbes J, Ohrn E, Weiner G. Slippage and/or symbolism: gender, policy and educational governance in Scotland and Sweden. Gender Educ 2011; 23: 761–76.
95. Barry J, Berg E, Chandler J. Movement and coalition in contention: gender, management and academe in England and Sweden. Gender Work Organ 2012; 19: 52–70.
96. Gornick J, Meyers M. Creating gender egalitarian societies: an agenda for reform. Polit Soc 2008; 36: 313–49.
97. Tomlinson J. Gender equality and the state: a review of objectives, policies and progress in the European Union. Int J Hum Resour Manag 2011; 22: 3755–74.
98. The World Bank. World Development Report: the political economy of gender reform. World Dev Rep 2012; 2012: 330–58.
99. The World Bank News and Broadcast (2012). Setting international standards for gender equality in the private sector. The Gender Equity Model. Available from: http://web.worldbank.org/WBSITE/EXTERNAL/NEWS/0,,contentMDK:23203941~pagePK:34370~piPK:34424~theSitePK:4607,00.html [cited 5 August 2012].
100. OECD (2012). Bridge development-gender. Available from: http://www.oecd.org/dataoecd/46/47/43041409.pdf [cited 7 August 2012].
101. Olah L. Should governments in Europe be more aggressive in pushing for gender equality to raise fertility? The second ‘YES’. Demogr Res 2011; 24: 179–200.
102. Bernardi L, Hetze P. Preface to the Rostock debate on demographic change. Demogr Res 2011; 24: 175–8.
103. Neyer G. Should governments in Europe be more aggressive in pushing for gender equality to raise fertility? The second ‘NO’. Demogr Res 2011; 24: 225–50.
104. Ronsen M, Skrede K. Can public policies sustain fertility in the Nordic countries? Lessons from the past and questions for the future. Demogr Res 2010; 22: 321–46.
105. Malmö stad (2012). Så arbetar vi med Jämställdhetsintegrering. Available from: http://www.malmo.se/Kommun--politik/Sa-arbetar-vi-med.../Jamstalldhetsintegrering/Bakgrund.html [cited 10 August 2012].
106. Malmö Stad (2010). Rapport – om nuläget i Malmö stad inför framtagande av förslag till utvecklingsplan för jämställdhetsintegrering.
107. Malmö stad S (2011). Tjänsteutlåtande KS-KOM-2010-00632: Utvecklingsplan för jämställdhetsintegrering i Malmö stad 2011–2020.
108. Malmö Stad (2001). Övergripande plan för jämställdhetsarbetet i Malmö stad.
109. Regeringen (2011). Integration och jämställdhet.
110. Jämställdhetsombudsmannen (JämO) (2007). Grundbok – att arbeta fram en jämställdhetsplan/JämO.
111. Glans H, Rother B. Dragkraft! Om jämställdhetsintegrering och ESF Jämt; 2011.
112. Malmö Stad (2012). Jämställdhet och mångfaldsplan; Limhamn-Bunkeflo stadsdelsförvaltning.
113. Malmö Stad (2012). Jämställdhet och mångfaldsplan: Oxie Stadsdelsförvaltning. 2012–2014.
114. Malmö Stad (2010). Slutrapport JÄMSTÄLLDHET – En ny ambition Rosengård stadsdelsförvaltning.
115. Lund University (2009). Handlingsplan för jämställdhet, likabehandling och breddad rekrytering vid Teaterhögskolan i Malmö.
116. Nordregio (2009). Att stärka tryggheten i stads- och tätortsmiljöer ur ett jämställdhetsperspektiv.
117. Malmö Stads Gatukontoret (2010). Hållbar jämställdhet inom Framtidens kollektivtrafik.
118. Länsstyrelsen i Skåne Län (2012). Trygghet och jämställdhet i samhällsplaneringen. Available from: http://www.lansstyrelsen.se/skane/Sv/samhallsplanering-och-kulturmiljo/planfragor/aktuella-planeringsfragor/tryggt-och-jamnt/Pages/tryggt-och-jamnt.aspx
119. Job och uddannelse i Öresundsregionen (2011). Öresundsregionen: tillgänglighet och mobilitet av arbetskraften. Available from: http://jobuddannelse.net/files/dokumenter/artikler/oresundsregionen2c20tillganglighet20och20mobilietet20av20arbetskraften_0.pdf [cited 5 August 2012].
120. 120.Statistiska Centralbyrån (SCB) (2012). Arbetsmarknad, Befolkningen 16+ år efter kommun, sysselsättning (förvärvsarbetande), ålder och kön. Available from: http://www.ssd.scb.se/databaser/makro/visavar.asp?yp=duwird&xu=c5587001&lang=1&langdb=1&Fromwhere=S&omradekod=AM&huvudtabell=YREG1&innehall=Befolkningen&prodid=AM0208&deltabell=K1&fromSok=&preskat=O [cited 5 August 2012].
121. Malmö Stad (2011). Personal facta. Available from: http://www.malmo.se/Medborgare/Jobb--praktik/Jobba-hos-oss/Personalfakta.html [cited 5 August 2012].
122. Malmö Stad (2011). Malmö stads personalredovisning. Available from: http://redovisningar.malmo.se/redovisning/assets/personalredovisning.pdf [cited 5 August 2012].
123. Malmö Stad (2011). Utbildningsförvaltningen. Jämställdhetsplan.
124. Malmö Stad (2012). Budget 2012. Kommunfullmäktiges Bihang Nr 801/2012.
125. Malmö Stad (2011). Lediga-jobb. Utbildare/genuspedagog i genus- och jämställdhet till FoU Malmö-utbildning. Available from: http://webapps03.malmo.se/lediga-jobb/item/7505F3E0-DB22-42AB-B556-71404CC576B6 [cited 5 August 2012].
126. Statistiska Centralbyrån (SCB) (2011). Befolkningens utbildning. Utbildning för befolkningen efter hemkommun. Available from: http://www.scb.se/Pages/ProductTables____9575.aspx [cited 5 August 2012].
127. Malmö Stad (2010). Stort intresse för jämställdhet bland Malmös skolor och förskolor. 2010. Available from: http://www.malmo.se/Medborgare/Forskola—utbildning/Nyhetsarkiv-Forskola—utbildning/Arkiv/1-20-2010-Stort-intresse-for-jamstalldhet-bland-Malmos-skolor-och-forskolor-.html [cited 5 August 2012].
128. Malmö högskola (2009). Nästan 1,5 miljoner för att studera jämställdhet. Available from: http://www.mah.se/Nyheter/Nyheter/Nastan-15-miljoner-till-jamstalldhet/ [cited 5 August 2012].
129. Malmö högskola (2010). Delegationen för jämställdhet gästade Malmö högskola. Available from: http://www.mah.se/Nyheter/Nyheter-2010/Delegationen-for-jamstaldhet-gastade-Malmo-hogskola/ [cited 5 August 2012].
130. Skanskan.se (2012). Jämställdhet rörde upp politiska känslor; 2012. Available from: http://www.skanskan.se/article/20120412/MALMO/704129723/-/jamstalldhet-rorde-upp-politiska-kanslor [cited 5 August 2012].
131. Malmö högskola (2012). Chefskap – jämställdhet. Available from: http://mah.se/cta/ikea/chefskap_jamstalldhet [cited 5 August 2012].
132. Gottzén L, Jonsson R. Andra män (Ny). Maskulinitet, normskapande och jämställdhet. Gleerups; 2012. Available from: http://webbshop.gleerups.se/se/uni-_hog/socialt_arbete-_vardutbildningar/sociologi/andra_man.ecp?groupdetail=true&PositionId=15&product_category_id=1617 [cited 5 August 2012].
133. Malmövänstern v-blog (2012). Malmös läromedel håller inte måttet. Available from: http://malmovanstern.v-blog.se/2012/04/03/malmos-laromedel-haller-inte-mattet/ [cited 8 August 2012].
134. Sydsvenskan (2012). Utbildningar ska skydda kvinnor. Available from: http://www.sydsvenskan.se/malmo/utbildningar-ska-skydda-kvinnor/ [cited 5 August 2012].
135. Langréus A, Nilsson H. Flickor är pojkar är flickor är...- en undersökning om jämställdhet i förskola och skola; 2006.
136. Statistiska Centralbyrån (SCB) (2010). Befolkningens studiedeltagande. Studiedeltagande för befolkningen efter hemkommun. Available from: http://www.scb.se/Pages/ProductTables____9611.aspx [cited 4 August 2012].
137. Malmö Stad (2011). Folkmängd i Malmö efter kön och ålder. Available from: http://www.malmo.se/download/18.72bfc4c412fc1476e02800046044/Folkm%C3%A4ngd+i+Malm%C3%B6+efter+k%C3%B6n+och+%C3%A5lder%2C+1+jan+2011.pdf [cited 5 August 2012].
138. Statistiska Centralbyrån (SCB) (2011). Befolkning. Available from: http://www.scb.se/statistik/_publikationer/OV0904_2011A01_BR_05_A01BR1101.pdf [cited 5 August 2012].
139. Statistiska Centralbyrån (SCB) (2011). Befolkningens utbildning. Utbildningsnivå för befolkningen efter inrikes/utrikes född, kön och åldersgrupp. Available from: http://www.scb.se/Pages/ProductTables____9575.aspx [cited 5 August 2012].
140. JobbMalmö (2012). Resursenheten. Unga kvinnor. Available from: http://www.malmo.se/Medborgare/Jobb—praktik/JobbMalmo/Resursenheten/Unga-kvinnor.html [cited 5 August 2012].
141. Ledarna Sveriges chefsorganisation (2011). Agenda Jämställdhet: ‘Det börjar med chefen’.
142. JobbMalmö (2012). Resursenheten. Krami för kvinnor. Available from: http://www.malmo.se/Medborgare/Jobb—praktik/JobbMalmo/Resursenheten/Krami-for-kvinnor.html [cited 5 August 2012].
143. Bettio F, Verashchagina A. Gender segregation in the labour market: root causes, implications and policy responses in the EU; 2008; B14033.
144. Foretagarna. Jämställt Företagarindex 2012: VAr är företagandet mest (och minst) jämställt?; 2012.
145. Veckans affärer (2012). Bästa företagen på jämställdhet. Available from: http://www.va.se/ledarskap/sveriges-basta-arbetsplatser/basta-foretagen-pa-jamstalldhet-323065 [cited 5 August 2012].
146. Bloggportalen (2011). Jämställdhet nästa undersökning.
147. Malmö högskola (2009). Pengar för jämställdhet finns att söka. Available from: http://mah.se/Nyheter/Nyheter/Jamstalldhet-i-hogskolan/ [cited 5 August 2012].
148. Nilson M, Andersson B. Jämställdhet I Näringslivet. Samlade Erfarenheter Från Tolv Företag i Skåne; 2007.
149. Fiberoptic Valley (2009). Best practise: innovation och jämställdhet Malmö. Available from: http://fiberopticvalley.com/blog/nyheter/best-practise-innovation-och-jamstalldhet-malmo-9-juni/ [cited 5 August 2012].
150. Hemström O. Health inequalities by wage income in Sweden: the role of work environment. Soc Sci Med 2005; 61: 637–47.
151. Krantz G, Lundberg U. Workload, work stress, and sickness absence in Swedish male and female white-collar employees. Scand J Public Health 2006; 34: 238–46.
152. Region Skåne (2010). Regionalt Resurs Centrum för kvinnor. Available from: http://www.skane.se/sv/Webbplatser/Resurscentrum/Jamstalldhet/ [cited 5 August 2012].
153. Kvinnojouren (2012). Kriscentrum för kvinnor i Malmö. Available from: http://www.kvinnojouren.se/jour/kriscentrum-kvinnor-i-malmo [cited 5 August 2012].
154. Sydsvenskan (2012). Karin hjälper utsatta kvinnor. Available from: http://www.sydsvenskan.se/malmo/karin-hjalper-utsatta-kvinnor [cited 20 July 2012].
155. Svenska Dagbladet (2012). Kvinna knivskars till döds i Malmö. Available from: http://www.svd.se/nyheter/inrikes/kvinna-knivskars-till-dods-i-malmo_6861633.svd [cited 20 July 2012].
156. Sydsvenskan (2012). Kvinna misstänkt mördad. Available from: http://www.sydsvenskan.se/malmo/kvinna-misstankt-mordad [cited July 2012].
157. Sveriges Radio Malmö (2012). Misshandlad kvinna avled.
158. Malmovänstern (2012). Jämställdhet. Available from: http://malmovanstern.v-blog.se/category/jamstalldhet/ [cited 8 August 2012].
159. Malmö Stads jämställdhetspolitiken (2003). Utvärderingsrapport om förstudie till projektet Jämställdhet. Malmö.
160. Sveriges Kommuner och Landsting (2011). Program för Hållbar Jämställdhet, Resultatrapport För Perioden 2008–2010. Stockholm.
161. Riksidrottsförbundet (2011). Utvärdering av Riksidrottsförbundets jämställdhetsarbete mellan åren 2005–2010. Stockholm.
162. E(uro)QUALITY Jämställdhetskonsult (2011). Medarbetarenkät; jämställdhet och mångfald. Available from: www.euroquality.se, 2012.

## Appendix 2 Policy Empowerment Index Footnotes

The PEI was previously developed to assess policy elements that have a potential impact on empowerment. It applies logical discussions but, due to lack of evidence on empowering factors’ impacts, not evidence-weighted scores (from 0=minimum to 5=maximum) of empowerment potential (21) and confidence intervals (0–5). In order to make the results more visible) the PEI evaluators attach scores to logical statements. For example, <10% describes ‘a small minority, 10–50% ‘a significant minority’, 50–75% ‘a majority’, 75–90% ‘a big majority’ and 90–100% ‘a vast majority/ almost all’ as previously described (21).

Unless otherwise specified, the score of 0 should be applied when the policy does not benefit the constituents or has a negative impact.

In the case of insufficient information or non-applicable criteria the relevant questions should be removed and the score re-adjusted.

The final score is presented as a proportion of the maximum points (50).

According to the index, a policy can be classified as:

1. Dictative: In this policy type, the policy-makers are addressing a problem that a minority of their constituents know or are concerned about. Mainly expert opinion and central interests guide the policy agenda setting and planning. The plans call for top–down implementations, without significant adaptation to peripheral particularities, needs and participation. The policy does little to build community capacity through education, training or employment opportunities or to protect vulnerable populations. Human, financial and other resources are centrally managed and inadequate. No formative or summative impact evaluations are considered necessary and central, inflexible mechanisms are or are put in place for policy modifications. The policy is interdependent with other dictative policies. PEI: 0–20%

2. Directive: A specific, detailed policy is designed, based on expert opinion and firm strategic goals, after considering public opinion and possible opposition, but mainly based on partisan views (e.g. the ruling party political ideology). Central and peripheral, expert panel committees may contribute to the policy plan. The policy-experts provide a detailed guidance to reach the policy objectives, allowing little deviation in planning and implementation, however taking into consideration local particularities, input and voices of objection, also allowing for some peripheral participation, variation and adaptation to an extent such that the end outputs remain within the prescribed margins. Contrary to the previous level, peripheral authorities are respected although relatively passive partners in the policy planning and implementation, under the firm guidance of the higher legislative framework. The policy builds peripheral capacity to some extent through education, training and provisions for limited employment opportunities and participation in peripheral networks. It protects and empowers affirmatively a minority of vulnerable constituents. Adequate, mainly central resources are planned. The policy is evaluated centrally for predefined outputs and outcomes, using quantitative measurements and can be adapted by central or peripheral inputs, albeit rather inflexibly. It relates and interacts with directive policies. PEI: 21–40%

3. Supportive: The policy addresses a problem that the majority of its constituents know of and are concerned about. Lay representatives together with experts contribute in setting the policy agenda and in policy planning, albeit centrally. A significant part of the implementation depends on peripheral mechanisms and peripheral capacity building is provided. Also, the policy is facilitating wider employment and peripheral network formation and action through legislational provisions and affirmatively supports the majority of vulnerable constituents. The central authority is providing adequate funds, advice, and actively supports peripheral implementation, for example, by providing expert advisers, information and a permissive national policy framework. It evaluates the policy implementation and impact formatively using both quantitative and qualitative methods and flexible, central mechanisms for adaptation exist or are set. The policy relates mainly with other supportive policies. PEI: 41–60%

4. Enabling: An enabling policy frequently addresses a problem that the vast majority of those affected know of and are concerned with. The agenda is set by an interaction of central and peripheral actors, and the policy planning occurs at close collaboration between the center and the periphery. Implementation is mainly delegated to the periphery. The policy also encourages peripheral sustained participation in assessment of needs and relevant action through sustained training and education of constituents, and through promotion of peripheral entrepreneurship, networking and cooperation at and between different levels. Adequate financial, informational and legislative support are combined with local resources and used as catalysts for peripheral involvement, participation and leadership, thereby promoting peripheral capacity development, proactively supporting and empowering the vast majority of vulnerable populations. Peripheral formative evaluation occurs, mainly through quantitative data collection and the policy can be adapted at least at the peripheral level, flexibly. The policy is part of a wider enabling policy matrix. PEI: 61–80%

5. Empowering: The policy is inspired as well as planned and implemented by the constituents under an empowering central and peripheral legislative framework, in a representative, consensual, polyphonic, equitably participatory manner, addressing issues that nearly all the affected constituents are informed and concerned with. It promotes further peripheral achievement and a sustainable, social, democratic development through essential, broad capacity development, through knowledge generation and communication, innovative and expansive peripheral entrepreneurship and asset generation, and through broad, inclusive and active, vertical and horizontal networks/links. Most vulnerable groups are affirmatively empowered. Peripheral participatory needs, formative and summative evaluations are conducted in a holistic manner (quantitative and qualitative, participatory action research) and a continuous feedback drives adaptations flexibly at the peripheral and central-framework level. Related policies follow the same principles. PEI: 81–100% (21).

## References

[1] World Health Organization (WHO) (2012). Women and gender equity.

[2] Sen G, Östlin P, George A (2007). Unequal, unfair, ineffective and inefficient – gender inequity in health: why it exists and how we can change it Report of the Women and Gender Equity Knowledge Network of the Commission on Social Determinants of Health.

[3] Fung A, Wright E (2001). Deepening democracy: innovations in empowered participatory governance. Politic Soc.

[4] Pratchett L, Durose C, Lowndes V, Smith G, Stoker G, Wales C (2009). Empowering communities to influence local decision making: a systematic review of the evidence.

[5] Kabeer N (1999). Resources, agency, achievements: reflections on the measurement of women’s empowerment. Dev Change.

[6] Kim J, Paul P, Barnet T, Watts C (2008). Exploring the role of economic empowerment in HIV prevention. AIDS.

[7] Woodall J, Raine G, South J, Warwick-Booth L (2010). Empowerment and health & well being: evidence review.

[8] European Commission (EC) (2007). Report on equality between women and men – 2007.

[9] Council of European Municipalities and Regions and its Partners (2006). The European charter for equality of women and men in local life.

[10] United Nations Development Programme (UNDP) (1995). Human Development Report. http://hdr.undp.org/en/reports/global/hdr1995/chapters/.

[11] Central Intelligence Agency (CIA) (2012). The World Factbook. https://www.cia.gov/library/publications/the-world-factbook/rankorder/2172rank.html.

[12] United Nations Development Programme (UNDP) (2011). Human Development Report 2011. Sustainability and equity: a better future for all.

[13] Statens Offentliga Utredningar (SOU 2005:66) (2005). Makt att forma samhället och sitt eget liv – jämställdhetspolitiken mot nya mål. Slutbetänkande av Jämställdhetspolitiska utredningen.

[14] Malmö Stad (2011). Utvecklingsplan för jämställdhetsintegrering.

[15] TNS-SIFO Surveys (2001). Jämställdhet. http://www.tns-sifo.se/rapporter-undersokningar/senaste-undersokningarna/2001/jaemstaelldhet-/.

[16] Malmö Stad (2012). Malmöfakta.

[17] Malmö Stad (2012). Malmöbor med utländsk bakgrund. http://www.malmo.se/download/18.6e1be7ef13514d6cfcc800030664/Malm%C3%B6bor+med+utl%C3%A4ndsk+bakgrund.pdf.

[18] Gavriilidis G, Östergren P (2012). Evaluating a traditional medicine policy in South Africa: phase 1 development of a policy assessment tool. Glob Health Action.

[19] Briones-Vozmediano E, Vives-Cases C, Peiro-Perez R (2012). Gender sensitivity in national health plans in Latin America and the European Union. Health Policy.

[20] Stewart DE, Dorado LM, Diaz-Granados N, Rondon M, Saavedra J, Posada-Villa J (2009). Examining gender equity in health policies in a low- (Peru), middle- (Colombia), and high- (Canada) income country in the Americas. J Public Health Policy.

[21] Garcia-Calvente MM, Castano-Lopez E, Mateo-Rodriguez I, Maroto-Navarro G, Ruiz-Cantero MT (2007). A tool to analyse gender mainstreaming and care-giving models in support plans for informal care: case studies in Andalusia and the United Kingdom. J Epidemiol Community Health.

[22] Stigendal M, Östergren P-O (2013). Malmö’s path towards a sustainable future: health, welfare and justice. Commission for a socially sustainable Malmö.

[23] Gavriilidis G, Natarajan N, Östergren P-O (2013). ‘Empowerment evaluation of policies towards a socially sustainable Malmö’. http://www.malmo.se/download/18.31ab534713cd4aa921379ea/Prooposal+inc+evalutation.pdf.

[24] Malmö Stad (2012). Framsteg i arbetet för ett jämställt Malmö. http://www.malmo.se/Nyheter/Centrala/3-21-2012-Framsteg-i-arbetet-for-ett-jamstallt-Malmo.html.

[25] Sanderson I (2002). Evaluation, policy learning and evidence-based policy making. Publ Admin.

[26] United Nations Development Programme (UNDP) (2012). Human Development Reports, Indices and Data. OECD.

[27] SVT se (2006). Jämindex – Hela Landet (SvT). http://www.svt.se/content/1/c6/67/88/72/jamindexhelalandet.pdf.

[28] Organisation for Economic Co-operation and Development (OECD) (2012). Gender indicators: what, why and how?. http://www.oecd.org/social/genderequalityanddevelopment/43041409.pdf.

[29] Spinakis A, Anastasiou G, Panousis V, Spiliopoulos K, Palaiologou S, Yfantopoulos J (2011). http://ec.europa.eu/health/social_determinants/docs/full_quantos_en.pdf.

[30] Stiglitz J (2012). The Stiglitz Report: reforming the international monetary and financial systems in the wake of the global crisis, with members of the commission of experts of the President of the United Nations general assembly on reforms of the international monetary and financial system.

# Sub article: Gender equity and sexual and reproductive health in Eastern and Southern Africa: a critical overview of the literature

**DOI:** 10.3402/gha.v7.23717

**Published:** 2014-06-26

**Authors:** Eleanor E. MacPherson, Esther Richards, Ireen Namakhoma, Sally Theobald

**Affiliations:** 1 Department of International Public Health, Liverpool School of Tropical Medicine, Liverpool United Kingdom; 2 Research for Equity and Community Health Trust, Lilongwe, Malawi

**Keywords:** gender equity, sexual & reproductive health, Eastern and Southern Africa

## Abstract

**Background:**

Gender inequalities are important social determinants of health. We set out to critically review the literature relating to gender equity and sexual and reproductive health (SRH) in Eastern and Southern Africa with the aim of identifying priorities for action.

**Design:**

During November 2011, we identified studies relating to SRH and gender equity through a comprehensive literature search.

**Results:**

We found gender inequalities to be common across a range of health issues relating to SRH with women being particularly disadvantaged. Social and biological determinants combined to increase women's vulnerability to maternal mortality, HIV, and gender-based violence. Health systems significantly disadvantaged women in terms of access to care. Men fared worse in relation to HIV testing and care with social norms leading to men presenting later for treatment.

**Conclusions:**

Gender inequity in SRH requires multiple complementary approaches to address the structural drivers of unequal health outcomes. These could include interventions that alter the structural environment in which ill-health is created. Interventions are required both within and beyond the health system.

Eastern and Southern Africa (ESA) contains some of the poorest countries in the world. The burden of disease in these settings due to infectious diseases and diseases related to reproductive and sexual health is extremely high. Notably, all 10 countries with the highest prevalence of HIV are found in the region, and the high rates of population mortality related to the epidemic have had a devastating impact on socio-economic development. Further, as the region with the highest number of young people in the world, understanding the drivers of sexual and reproductive health (SRH), including gender inequality, could provide valuable insights for improving broader health and development outcomes.

This review of the literature draws on findings from a report commissioned by the Regional Network for Equity in Health in Eastern and Southern Africa (EQUINET) which highlighted areas of concern for gender equity in health in ESA, based on a review of the published and grey literature (1). The paper synthesises evidence from the academic and grey literature on key aspects of SRH from 16 countries in the Eastern and Southern African region and highlights neglected areas where further progress is urgently required.

Gender refers to how a person's biological sex is culturally valued and interpreted into locally accepted ideas of what it is to be female or male (2). Gender therefore describes all the socially given attributes, roles, activities, and responsibilities connected to being male or female in any given society (3). In most societies, these gendered social norms divest greater privileges (often power and resources) to men and boys over women and girls. Gendered social norms are dynamic, changing over time, and varying across cultures (2, 4). Gender differences in access to information and resources (both social and financial) can impact on nutrition, education, employment, and income. These are all important determinants of good health (5). There is therefore a need to better understand how gender shapes vulnerability to ill-health and health sector responses so that health services can address the needs of women, men, girls, and boys more equitably (i.e. through channelling resources where they are most needed). Gender equity denotes equivalence in life outcomes for women and men, recognising their different needs and interests, and requiring a redistribution of power and resources (2).

SRH relates to the health and well-being of people in matters related to sexual relations, pregnancy, and birth. The ability of women to realise their sexual and reproductive rights is vital to achieving gender equity in health as well as the empowerment of women. The aim of this paper is to critically review the published and grey literature on gender and SRH in ESA, identifying key areas of concern, areas of innovation and action, and charting the way forward. We discuss using a gender lens the following key components of SRH: access to abortion, maternal mortality, unmet contraceptive needs, gender-based violence, and HIV (including prevention, access to anti-retroviral therapy (ART), and caring responsibilities).

## Methods

During November 2011, we conducted a literature search of electronic databases for academic literature on gender equity and health in ESA. We developed key search terms which included sex, gender, men, women, reproductive health, disease, Southern and Eastern Africa. The two databases we used for these searches were Medline and Web of Knowledge. We also used a Google Search to ensure we included a range of literature.

We limited the searches to include the following 16 countries in ESA: Uganda, the Democratic Republic of Congo (DRC), Kenya, Tanzania, Zambia, Malawi, Mozambique, Madagascar, Mauritius, Angola, Namibia, Botswana, Swaziland, Lesotho, South Africa, and Zimbabwe. We focused on these countries because they provided a range of countries including those with more and less stable political and economic systems and higher and lower HIV epidemics. We included studies for the review that related to gender equity and health (both inside and beyond the health system) in the 16 countries and excluded those that did not relate to gender equity and health.

Once we had identified key papers, we reviewed each included paper through undertaking a thematic analysis. As a group, we discussed the key findings and identified broad themes to include in the final write up.

In this paper, we have focused specifically on our findings related to SRH. We identified key themes in this area including: measures of gender inequalities; maternal mortality; abortion; unmet contraceptive needs; HIV risk, treatment and care; and gender-based violence. We brought these findings together and discussed as a group to identify key priorities for action to improve the response to address gender inequalities in SRH. Key limitations of this review were: first, the lack of primary data available for the ESA settings related to gender and health, although there were more data available for South Africa and Kenya than for other countries; second, there was a lack of empirical work that identified positive gender equity action in health; thus, the review was largely reliant on descriptive case studies; third, while men and boys are also affected by gender power relations, there were few studies available that focused on men and how gendered norms and expectations can impact on their health; fourth, in reviewing the papers, there were limited papers relating to sexually transmitted diseases other than HIV/AIDS and hence the focus is on HIV rather than the broader set of sexually transmitted infections (STIs).

## Results

Using the search strategies, we identified 1,291 potentially relevant published articles. All titles and abstracts were read and any duplicates or papers that were not relevant to gender and health were omitted. This left 189 unique papers that were reviewed in full before removing a further 40 manuscripts with irrelevant or repeated data. We also identified 26 papers from the grey literature by conducting Google searches and reviewing references in journal articles. The results presented are key themes related specifically to SRH.

### Introducing the ESA countries through the Gender Inequality Index

The Gender Inequality Index (GII) is part of the United Nations Development Programme (UNDP) and is constructed based on three dimensions – reproductive health (maternal mortality and adolescent fertility), empowerment (female parliamentary participation and secondary level education), and women's participation in labour markets (6). A higher score on the index means that gender inequality is more pronounced in the country. While the index includes only a limited number of indicators of gender inequality, it provides a useful summary of gender inequities across countries. It indicates that in countries where human development is uneven there is also high inequality between men and women. Table 1 below presents UNDP GII for the 16 countries. Mauritius has the lowest gender inequality and the DRC has the highest. The civil war in DRC is likely to play an important role in explaining why the country performs poorly in this index. War can be especially damaging to the rights of women and children. In DRC, there have also been extensive reports of rape, sexual slavery, purposeful mutilation of women's genitalia, and killings of rape victims, particularly in eastern Congo (7). In comparison, Mauritius has a stable democracy with one of the highest per capita incomes in Africa.

**Table 1:** UNDP Gender Inequality Indices, 2011

EQUINET focal countries	UNDP Gender Inequality Index 2011
Democratic Republic of the Congo	0.71
Kenya	0.63
Zambia	0.63
Mozambique	0.60
Malawi	0.59
Tanzania	0.59
Uganda	0.58
Zimbabwe	0.58
Swaziland	0.55
Lesotho	0.53
Botswana	0.51
South Africa	0.49
Namibia	0.47
Mauritius	0.35
Angola	Data unavailable
Madagascar	Data unavailable

Source: UNDP (6).

### Maternal mortality

Women's access to antenatal services and support during labour are vital elements of SRH rights. Maternal mortality is defined as the death of a woman during pregnancy, childbirth, or in the first 42 days after giving birth (8). The maternal mortality ratio (MMR) is the number of maternal deaths in a population divided by the number of live births (9). It is one of the health indicators that shows the greatest gap between the rich and the poor – both between countries and within them (10). This is highlighted by the fact that 99% of all maternal deaths occur in developing countries, with more than half of these deaths occurring in sub-Saharan Africa (9). A woman's lifetime risk of maternal death is 1 in 7,300 in developed countries versus 1 in 75 in developing countries (10). Power dynamics within the household can mean that women are unable to take control over their reproductive health, putting them at increased risk of maternal death.

With the adoption of Millennium Development Goal 5 (MDG 5), countries have committed to reducing the MMR by three quarters between 1990 and 2015 (11). However, between 1990 and 2005, the MMR declined by only 5%. Achieving MDG 5 requires accelerating progress. With the exception of Mauritius, none of the ESA countries in this review are likely to achieve their MDG5 target (8).

In Table 2, the MMR of the 16 countries in the study are represented for 1980, 1990, 2000, and 2008. There has been a downward trend in mortality in some of these countries, including Madagascar, Angola, Mauritius, Uganda, Kenya, and Tanzania. Although in relation to Kenya the gains made are slight, and between 1990 and 2000 there was a significant increase in maternal mortality. Some of the improvements in maternal mortality in these countries may be explained by a number of factors. Hogan et al. (8) argue that globally total fertility rates have been declining since 1980 (3.70 in 1980 to 3.26 in 1990 and 2.56 in 2008). There has also been an increase of per capita income (although this is less marked in ESA countries than in other regions of the world). Rising per capita income can impact on maternal mortality through improving women's nutritional status and their financial access to health. Educational attainment may also play a role in these improvements (maternal educational attainment is a strong correlate of maternal mortality) and has been rising in sub-Saharan Africa since 1980 (12).

**Table 2:** MMR of the 16 countries in 1980, 1990, 2000, and 2008

	MMR per 100,000 live births			
		
Country	1980	1990	2000	2008
Angola	1,309	1,156	1,105	593
Botswana	424	237	655	519
DRC	498	616	850	534
Kenya	494	452	730	413
Lesotho	588	363	1,021	964
Madagascar	490	484	505	373
Mauritius	122	65	34	28
Malawi	632	743	1,162	1,140
Mozambique	411	385	505	599
Namibia	397	354	558	586
South Africa	208	121	155	237
Swaziland	559	359	609	736
Tanzania (United Republic of)	603	610	714	449
Uganda	435	571	604	352
Zambia	599	594	914	603
Zimbabwe	219	171	373	381

Source: Hogan et al. (8).

MMR, maternal mortality ratio.

The countries with upward trends of worsening maternal mortality include Botswana, DRC, Lesotho, Malawi, Mozambique, Namibia, South Africa, Swaziland, Zambia, and Zimbabwe. With the exception of DRC, all of these countries lie in Southern Africa and have some of the highest HIV prevalence rates in the world. The high HIV prevalence is likely to be a significant factor in shaping these poor outcomes in maternal mortality (12). Therefore, interventions to prevent HIV transmission and the early initiation of ART are likely to be important interventions to reduce maternal mortality. While HIV provides some explanation for persistent high levels of maternal mortality in the majority of ESA countries, it is clear that other factors contribute to this, which we discuss below.

Nearly half of all maternal deaths in developing countries, including the ESA region, occur during labour, delivery, or the immediate post-partum period (13). The ‘three delays’ model developed by Nour^1^ (14) reflects the important role that gender relations play within the household (14). Women face significant challenges in accessing health services at these critical stages which is shaped by their access and control of resources, as well as the prevailing gender norms and values. For example, economic resources and their distribution within the family may prevent or delay women from seeking treatment (14). Furthermore, cultural and religious norms may influence the availability and accessibility of key health interventions such as contraception as well as abortion procedures.

### Abortion

In 2003, there were an estimated 20 million unsafe abortions. Nearly 98% of them were in the Global South in countries which have restrictive abortion laws (15). Nearly 200 women die each day from abortion-related complications (16). Africa accounts for 25% of all illegal abortions performed worldwide and less than 1% of all legal abortions (17). An estimated 90% of deaths from unsafe abortions and 20% of obstetric mortality could be averted by universal access to modern family planning methods. As can be seen in Table 3 on abortion laws in the 16 ESA countries, every country allows abortion on the grounds of saving a woman's life (18). However, there is wide variation in whether women can get an abortion for any other reason. Angola, DRC, and Lesotho have the most restricted while South Africa has the most liberal abortion laws. In 1996, following the end of apartheid rule and the transition to democracy, the South African government introduced the Choice on Termination Act, No. 92, 1996, which granted abortions on a number of grounds, including on request.

**Table 3:** Legal grounds for abortion, 2007

	Grounds on which abortion may be permitted in 16 review countries						
	
Countries	To save a woman's life	To preserve physical health	To preserve mental health	Rape or incest	Foetal impairment	Economic or social reason	On request
Angola^a^	√	×	×	×	×	×	×
Botswana	√	√	√	√	√	√	×
Democratic Republic of Congo^a^	√	×	×	×	×	×	×
Kenya^b^	√	√	√	×	×	×	×
Lesotho	√	×	×	×	×	×	×
Madagascar^a^	√	×	×	×	×	×	×
Malawi^a^	√	×	×	×	×	×	×
Mauritius^a^	√	×	×	×	×	×	×
Mozambique	√	√	√	×	×	×	×
Namibia	√	√	√	√	×	×	×
South Africa	√	√	√	√	√	√	√
Swaziland	√	√	√	×	√	×	×
Tanzania	√	√	√	×	×	×	×
Uganda^b^	√	√	√	×	×	×	×
Zambia	√	√	√	×	√	√	×
Zimbabwe	√	√	×	√	√	×	×

Source: WHO (15).

a The abortion laws in these countries do not expressly allow abortions to be performed to save the life of the woman, but general principles of criminal legislation allow abortions to be performed for this reason on the grounds of necessity.

b The abortion laws in these countries expressly allow abortions to be performed only to save the life of the woman, or are governed by general principles of criminal legalisation which allow abortions to be performed for this reason on the ground of necessity.

In South Africa, clause 59.1 of the post-apartheid Constitution (1996) requires that Parliament facilitate public involvement in legislative and other processes of the assembly and its committees. Women's rights groups successfully used this clause to mobilise for legalisation of abortion. One of the key strategies was to allow women who have experienced unsafe abortions first hand to speak about the need for liberal abortion laws before Parliament. These women also provided quantitative evidence of costs to the government (that could be avoided) due to complications of unsafe abortion. As a result, South Africa thoroughly liberalised its abortion laws. The Choice on Termination Act No. 92, 1996, allows abortion on request up to the first trimester, permits midwives to conduct abortions, and allows adolescent girls the right to access abortion without parental consent.

Jewkes and Rees report a 91% reduction in deaths from unsafe abortion from 1994 (before the Termination Act had been passed) to 2000 (once the Act was in operation) (19). They compared abortion-related deaths from more than one source (Department of Health reports on confidential enquiries into maternal deaths from 1999 and 2003 and the national incomplete-abortion survey from 2000), with the estimates of pre-legislative reform mortality found in the national incomplete-abortion survey from 1994. The 1994 survey estimated that there were 425 deaths each year in public facilities from unsafe abortion. When the survey was repeated in 2000, no deaths were detected in the 3-week data collection period in any study hospital. Therefore, researchers were able to conclude that a significant decline in mortality had occurred although it was not possible to estimate the annual number of deaths (19, 20).

### Unmet contraceptive needs

Unmet contraceptive needs can be used as a proxy measure for accessing family planning services (21). This is because most unplanned pregnancies in the developing world are due to lack of access to family planning services. As shown in Fig. 1, where data are available, there is a gap in unmet family planning needs (22).

**Fig. 1:** Proportion of women with unmet family planning needs, 1990–1999 and 2000–2007. Source: WHO (22).

This is particularly pronounced in Uganda where unmet need has increased from 29% in 1990–1999 to 41% in 2000–2007. The studies we identified on this topic did not explicitly explore the reasons for this increase. However, women who desire to space or limit births may face multiple barriers both within their relationships and also related to the wider provision of health services they have access to. Meeting women's family planning needs is vital for empowering women. In countries where abortion is restricted, as shown in Table 3, unmet contraceptive need can mean women are more likely to have unsafe abortions. It can also mean that women are at an increased risk of maternal mortality.

It is critical that SRH services are accessible for all. In ESA countries this means improving the coverage of family planning services to widen their availability, particularly for hard-to-reach populations, as well as improving their accessibility and responsiveness to the needs of users. Ostlin (10) argues that in some countries the introduction of women and adolescent friendly services has helped to counteract judgmental attitudes of providers, and lack of privacy and confidentiality (10). Services could be adapted to include youth-only, men-only clinics or women-only services or outreach and community-based services (10).

### HIV risk, care, and prevention

Globally, the response to HIV and AIDS has triggered an unprecedented focus on gender inequality and how this shapes women's vulnerability (23). This response has not been mirrored in other aspects of disease prevention or within health systems more generally. One of the reasons for this unparalleled response is the statistics relating to HIV. Of the estimated 34 million people living with HIV (PLWHIV) worldwide (24), women constitute half of all adults living with the disease. However, in sub-Saharan Africa, there are 14 HIV-positive women for every 10 HIV-positive men (25). Seventy-five per cent of new HIV infections occur among young girls and female adolescents in Southern Africa (25). Women aged 15–24 are twice as likely to be infected with HIV than boys of the same age in the region (25). The higher risk in young women is due to both sociological and physiological risk factors – young women have immature genital tracts but are more likely to have older sexual partners partly due to gendered expectations of men's and women's roles as men are expected to have younger sexual partners (26).

As can be seen from Table 4, HIV prevalence in the 16 Southern and Eastern African countries varies widely from 0.2% for Madagascar and 1% for Mauritius to 25.9% for Swaziland and 23.6% for Lesotho (24). Southern Africa has borne the brunt of the HIV epidemic globally. Likewise, ART coverage varies significantly, with Botswana (93%) and Namibia (90%) performing best, and Madagascar (1%) the worst. South Africa scored a disappointing 55%, despite having the third highest HIV prevalence in the region (17.8%).

**Table 4:** Prevalence of HIV and percentage of ART coverage, 2010

Country	Prevalence (%)	ART coverage (%)
Angola	2	33
Botswana	24.8	93
DRC	No data available	No data available
Kenya	6.3	61
Lesotho	23.6	57
Madagascar	0.2	1
Malawi	11	No data available
Mauritius	1	16
Mozambique	11.5	40
Namibia	13.1	90
South Africa	17.8	55
Swaziland	25.9	72
Tanzania	5.6	42
Uganda	6.5	47
Zambia	13.5	72
Zimbabwe	14.3	59

Source: UNAIDS (24).

Gendered roles and relations often place women in a subordinate position to men and promote models of masculinity that justify and reproduce men's dominance over women (27). As Grieg et al. argue, ‘notions of masculinity prevalent in many parts of the world that equate being a man with dominance over women, sexual conquest and risk-taking are associated with less condom use, more STIs, more partners, including more casual partners, more frequent sex, more abuse of alcohol and more transactional sex’ (27). Women are often economically dependent on men. This dependence can leave women with less power in sexual relationships, particularly in negotiating the terms of sexual exchange including condom use (28). In many ESA countries, masculine and feminine ‘ideals’ reflect views that promote the sexual prowess of men through multiple sexual partnerships, but frown on women who do the same (29).

Recent work has identified violence against women as an independent risk factor for HIV in South Africa (30, 31). Within a sexual relationship, the threat of violence can influence women's power and their ability to negotiate conditions of sexual intercourse, especially condom use (32). Women who face violence within their relationships may be less likely to access HIV testing services because of the fear of disclosing their sero-status (33). Dunkle and Jewkes present research from India, South Africa, and the United States suggesting that ‘men who are violent towards their female partners or commit rape’ are more likely to have sex more often, to have sex with concurrent and/or casual sexual partners, to have higher total numbers of sexual partners, to practice anal sex, to participate in transactional sex, to father children, and to use alcohol and drugs’ (33). These are all factors that increase risk of HIV.

Prevention of HIV has relied largely on behaviour change at the individual level. However, behaviour change interventions have often been gender-blind and have failed to acknowledge the broader environment that sexual relationships are conducted within. Gender power relations and economic vulnerability mean that women are often unable to change their behaviour particularly relating to condom use. A study in a peri-urban area in Malawi found that women can do very little to influence condom use by their husbands to protect themselves from HIV due to the perception that condom use implies infidelity; nor can they space or stop having children without their male partner's permission (34). Studies in Kenya and South Africa have found that men control condom use in sexual exchanges (35, 36).

Nevertheless, there are examples from South Africa of promising HIV prevention interventions with both women and men. For example, Stepping Stones is a socially transformative programme that promotes gender equality through participatory learning and action (37). The intervention works with community members, including men and boys. In South Africa a Stepping Stones intervention was evaluated with a group of rural youth using a randomised controlled trial. The trial found that with 2 years follow-up, Stepping Stones lowered the incidence of herpes simplex virus 2 in men and women by approximately 33%, and men reported less perpetration of intimate partner violence across 2 years of follow-up, as well as changes in several other HIV risk behaviours (37).

The gendered differences in financial inequality, authority relations, and social identities of men and women influence how families, communities, and health care systems react towards HIV infection among men and women (38). Views of blame and accusation have in some societies been directed more towards female PLWHIV than male PLWHIV (38).

However, gender roles and relations also influence access to HIV testing, treatment, and care. Studies have shown that more women receive treatment than men in ESA, and that when men do access treatment they often do so at a more advanced stage (39). This may reflect dominant masculine norms that prescribe not only an avoidance of the sick role but also discourage men from seeking care out of fear of being labelled weak.

Finally, care for HIV in ESA has gradually been shifting away from providing hospital-based care to home-based care (40). While this model of care has clear benefits for patients who wish to remain at home, it has often relied on women's unpaid labour reinforcing the stereotype that women are responsible for caring for the sick and that work of this nature is not worthy of remuneration (41–43).

### Gender-based violence

While gender-based violence is pervasive in many ESA countries, it is often under-recognised as a public health challenge. The extent to which women are exposed to violence varies across countries. The data indicate, however, that violence against women is widespread and manifests in many forms – physical, sexual, psychological, and economic – both within and outside the home. Violence limits women's autonomy and their ability to make decisions about their bodies. It can also have wide ranging impacts on the short and long term physical, mental, and sexual health problems of women (44).

These health problems can range from physical injuries to depression and suicide. Living with the threat of violence can also leave women unable to negotiate the terms of sexual relationships as well as contraceptive use. This can leave women vulnerable to unwanted pregnancies as well as sexually transmitted diseases (45).

According to Duggan (45), perpetrators of violence against women are most often their intimate partners; therefore, violence may be considered one of the ‘most graphic expressions of unequal household power relations’ (45). Women are abused physically and sexually by intimate partners at different rates throughout the world – yet such abuse occurs in all countries or areas, without exception. There are limited data available on the occurrence of violence against women in sub-Saharan Africa, but the United Nations Children's Fund estimates 13–49% of women reported having been physically assaulted by an intimate male partner (46). Studies on sexual violence in Ethiopia, Kenya, Namibia, Tanzania, Zambia, and Zimbabwe estimate that 14–59% of women have experienced sexual violence at some point during their lives (46). Pregnancy can also be a trigger for violence with 10% of ever-pregnant women in Zimbabwe and 7% in South Africa having been attacked during pregnancy (46).

South Africa has one of the highest rates of rape in the world and in a recent study 27.6% of men interviewed admitted raping a woman (47). This involved an intimate partner, stranger, or acquaintance, and the rape was perpetrated either alone or with accomplices. Further, 4.7% men admitted raping a woman in the past 12 months. In DRC, there have been reports of rebels using rape as a weapon of war to humiliate women and girls as well as to humiliate the women's spouses. It has also been used as a tool to terrorise and demoralise whole communities (48).

Action on gender-based violence is required at all levels, including community, legal, and health system levels. There are examples of such action in ESA countries. For example, an assessment in Kenya in 2003 revealed limited post-rape services, lack of policy, and tensions between HIV and reproductive health staff at service delivery points. Facilities lacked protocols and confidential spaces for treatment. In response, a standard package of care was developed with Liverpool Volunteer Counselling and Testing (LVCT). This included the provision of HIV post-exposure prophylaxis (PEP), psycho-social support, and gender-sensitive counselling for survivors (49). By June 2007, there were 13 health facilities providing post-rape care services in Kenya including the national referral and teaching hospital. Between them they had delivered services to over 2,000 adults and children, with 96% of those eligible initiating PEP at presentation (49).

## Conclusions

Synthesising published and grey literature from across the 16 ESA countries reveals that biologically and socially determined gender roles and relations influence the different exposures and vulnerabilities to illness experienced by women and men. Access to SRH is important for women and men of different ages. In ESA where maternal mortality rates and HIV prevalence remains unacceptably high, there is an urgent need to provide effective and accessible maternity services and gender-transformative HIV programmes. Gender norms and values influence availability and accessibility of critical SRH interventions such as contraception and abortion procedures. Within the context of the ESA countries, gender roles and relations play an important role in producing vulnerability to unwanted pregnancy and STIs. Notions of masculinity often condone multiple sexual partnerships and unsafe sexual practices, such as sex without a condom. They also influence treatment-seeking behaviour with men presenting later for testing and treatment, which in turn can influence treatment outcomes. Gender roles, which influence women's access to economic resources, can also increase vulnerability to HIV and other STIs because women may be more economically dependent on men and therefore less able to negotiate the terms of sexual exchanges.

Globally, the politics of SRH have always been contentious, and within the ESA contexts this is illustrated, for example, by the majority of countries limiting women's safe access to abortion. Of all the public health interventions, those related to SRH are the most likely to be influenced by politics, tradition, and religion rather than scientific evidence, especially where gendered issues are concerned. The unease that many people feel about discussing sex and the low status of women in many countries has meant that SRH rights have not always been enacted (50–52).

Gender inequity in SRH in ESA underlines the need for deeper levels of action to address the structural drivers of inequities in health outcomes. These could include interventions to transform gender roles and relations. Programmes such as Stepping Stones have good potential here through changing broader gender norms. Other interventions to address social determinants include improved access to education and employment.

It is clear from these ESA findings that interventions to improve SRH for women and men need to be enacted both within and beyond the health system. Tolhurst et al. (53) provide pertinent insights on the importance of advocacy and inter-sectoral approaches (53). They argue for increased advocacy in policy-making, service provision and resource allocation to establish the sexual and reproductive rights of women, men, boys and girls and to ensure these are met (53). Intervening requires moving beyond simple service delivery through using inter-sectoral collaboration to improve women's bargaining power and increase their broader access to and control over resources (53).

The causes of gender-based violence point to the need for multi-pronged approaches, including action at the community level to change cultural norms, decrease the acceptability of violence against women and girls, and improve the linkages between the health system and the legal system. Changing such norms calls for political, civil, and social leadership to change institutional practice and to debunk underlying ideologies. A common indicator of improved governance is the increase in women's participation at all levels of decision-making, including at the household and community levels and in national legislative bodies. However, wider transformations are needed to strengthen institutional functioning to address gender equity. Such transformations rely on the development of institutional capacity and governance based on robust evidence and examples of effective good practice. This includes advocacy for health information systems disaggregated by gender and by other important factors that shape vulnerability and resilience to ill-health (poverty, age, literacy, disability).

In conclusion, SRH is central to gender equity in health in the ESA region. Women's and men's access to these services remains sub-optimal which has serious health consequences for all, but particularly for women and girls. Access to these services is particularly important in ESA countries where HIV prevalence remains unremittingly high. It is vital to audit gender equality within the health system, to encourage programmes to carry out such assessments, to implement gendered evaluations, and to consult and obtain feedback from women and men in the community to strengthen gender equity in sexual and reproductive health.

## Footnotes

1 Nour (14) argues that there are three key delays that increase maternal mortality in resource-poor settings. These are: delays in deciding to seek care; delays in reaching care in time; delays in receiving adequate treatment.

## References

[1] MacPherson E, Richards E, Namakhoma I, Theobald S (2012). Dimensions of gender equity in health in East and Southern Africa.

[2] Reeves H, Baden S (2000). Gender and development: concepts and definitions.

[3] March C, Smyth IA, Mukhopadhyay M (1999). A guide to gender analysis frameworks.

[4] Esplen E, Jolly S (2006). Gender and sex: a sample of definitions.

[5] Doyal L, Payne S, Cameron A (2003). Promoting gender equality in health.

[6] UNDP (2011). Gender Inequality Index (GII).

[7] Wakabi W (2008). Sexual violence increasing in Democratic Republic of Congo. Lancet.

[8] Hogan MC, Foreman KJ, Naghavi M, Ahn SY, Wang M, Makela SM (2010). Maternal mortality for 181 countries, 1980–2008: a systematic analysis of progress towards millennium development goal 5. Lancet.

[9] WHO, UNICEF, UNFPA, World Bank (2007). Maternal mortality in 2005.

[10] Ostlin P Transforming health systems and services for women and girls.

[11] United Nations (2000). Millennium development goals (MDGs).

[12] Rasanathan K (2011). Closing the gap: policy into practice on social determinants of health. Discussion paper for the World Conference on Social Determinants of Health. 19–21 October 2011.

[13] UN Millennium Project (2005). Taking action: achieving gender equality and empowering women.

[14] Nour NM (2008). An introduction to maternal mortality. Rev Obstet Gynecol.

[15] WHO (2003). Unsafe abortion: global and regional estimates of incidence of unsafe abortion and associated mortality in 2003.

[16] Gordon P, Boler T, Burgin J, Brett T (2010). What women want: meeting the global demand for medical abortion.

[17] Baggaley RF, Burgin J, Campbell OMR (2010). The potential of medical abortion to reduce maternal mortality in Africa: what benefits for Tanzania and Ethiopia?. PLoS One.

[18] UN (2007). World abortion policies 2007.

[19] Jewkes R, Rees H (2005). Dramatic decline in abortion mortality due to the choice on termination of pregnancy act. S Afr Med J.

[20] Murthy RK (2008). Strengthening accountability to citizens on gender and health. Glob Public Health.

[21] Ortayli N, Malarcher S (2010). Equity analysis: identifying who benefits from family planning programs. Stud Fam Plann.

[22] WHO (2011). Health situation analysis in the African region: atlas of health statistics 2011.

[23] Gupta GR, Ogden J, Warner A (2011). Moving forward on women's gender-related HIV vulnerability: the good news, the bad news and what to do about it. Glob Public Health.

[24] UNAIDS (2011). Report on the global epidemic.

[25] UNAIDS, WHO (2008). 2008 Report on the global AIDS epidemic.

[26] Luke N (2005). Confronting the ‘sugar daddy’ stereotype: age and economic asymmetries and risky sexual behavior family planning perspectives in Urban Kenya. Int Fam Plan Perspect.

[27] Greig A, Peacock D, Jewkes R, Msimang S (2008). Gender and AIDS: time to act. AIDS.

[28] Côté AM, Sobela F, Dzokoto A, Nzambi K, Asamoah-Adu C, Labbé AC (2004). Transactional sex is the driving force in the dynamics of HIV in Accra, Ghana. AIDS.

[29] Kaler A (2003). “My girlfriends could fill a yanu-yanu bus”: rural Malawian men's claims about their own serostatus. Demogr Res.

[30] Jewkes RK, Dunkle K, Nduna M, Shai N (2010). Intimate partner violence, relationship power inequity, and incidence of HIV infection in young women in South Africa: a cohort study. Lancet.

[31] Türmen T (2003). Gender and HIV/AIDS. Int J Gynecol Obstet.

[32] Gupta GR (2002). How men's power over women fuels the HIV epidemic. BMJ.

[33] Dunkle KL, Jewkes R (2007). Effective HIV prevention requires gender-transformative work with men. Sex Transm Infect.

[34] Ghosh J, Kalipeni E (2005). Malawi: vulnerability and risk to HIV/AIDS. J Soc Aspect HIV AIDS.

[35] Bühler C, Kohler HP (2003). Talking about AIDS: the influence of communication networks on individual risk perceptions of HIV/AIDS infection and favored protective behaviors in South Nyanza District, Kenya. Demogr Res.

[36] Maharaj P, Cleland J (2004). Condom use within marital and cohabiting partnerships in KwaZulu-Natal, South Africa. Stud Fam Plann.

[37] Jewkes R, Nduna M, Levin J, Jama N, Dunkle KL, Puren A (2008). Impact of stepping stones on incidence of HIV and HSV-2 and sexual behaviour in rural South Africa: cluster randomised controlled trial. BMJ.

[38] Mbonu NC, Van den Borne B, De Vries NK (2010). Gender-related power differences, beliefs and reactions towards people living with HIV/AIDS: an urban study in Nigeria. BMC Public Health.

[39] Muula AS, Ngulube JT, Siziya S, Makupe CM, Umar E, Prozesky HW (2007). Gender distribution of adult patients on highly active antiretroviral therapy (HAART) in Southern Africa: a systematic review. BMC Public Health.

[40] Akintola O (2004). The gendered burden of home-based caregiving.

[41] Kipp W, Tindyebwa D, Rubaale T, Karamagi E, Bajenj E (2007). Family caregivers in rural Uganda: the hidden reality. Health Care Women Int.

[42] Newman CJ, Fogarty L, Makoae LN, Reavely E (2011). Occupational segregation, gender essentialism and male primacy as major barriers to equity in HIV/AIDS caregiving: findings from Lesotho. Int J Equity Health.

[43] Tarimo EA, Kohi TW, Outwater A, Blystad A (2009). Gender roles and informal care for patients with AIDS: a qualitative study from an urban area in Tanzania. J Transcult Nurs.

[44] Heise L, Garcia-Moreno C, Krug EG, Dahlberg LL, Mercy JA, Zwi AB, Lozano R (2002). Violence by intimate partners. World report on violence and health.

[45] Duggan L, Visvanathan N, Duggan L, Wiegersma N, Nisonoff L (2011). Household, families and work: Introduction. The women, gender and development reader.

[46] UNICEF (2011). UNICEF Eastern and Southern Africa: gender and health.

[47] Jewkes R, Sikweyiya Y, Morrell R, Dunkle KL (2011). Gender inequitable masculinity and sexual entitlement in rape perpetration South Africa: findings of a cross-sectional study. PLoS One.

[48] Mukwege D, Nangini C (2009). Rape with extreme violence: the new pathology in South Kivu, Democratic Republic of Congo. PLoS Med.

[49] Kilonzo N, Theobald S, Nyamato E, Ajema C, Muchela H, Kibaru J (2009). Delivering post rape care services: Kenya's experience in developing integrated services. Bull World Health Organ.

[50] Theobald S, Tulloch O, Crichton J, Hawkins K, Zulu E, Mayaud P (2011). Strengthening the research to policy and practice interface: exploring strategies used by research organisations working on sexual and reproductive health and HIV/AIDS. Health Res Policy Syst.

[51] Buse K, Martin-Hilber A, Widyantoro N, Hawkes SJ (2006). Management of the politics of evidence-based sexual and reproductive health policy. Lancet.

[52] Oronje RN, Crichton J, Theobald S, Oye Lithur N, Ibisomi L (2011). Operationalising sexual and reproductive health and rights in sub-Saharan Africa: constraints, dilemmas and strategies. BMC Int Health Hum Rights.

[53] Tolhurst R, Raven J, Theobald S, Ehiri J (2009). Gender equity: perspectives on maternal and child health. Maternal and child health: global challenges, programs, and policies.

# Sub article: The first 500 days of life: policies to support maternal nutrition

**DOI:** 10.3402/gha.v7.23623

**Published:** 2014-06-06

**Authors:** John B. Mason, Roger Shrimpton, Lisa S. Saldanha, Usha Ramakrishnan, Cesar G. Victora, Amy Webb Girard, Deborah A. McFarland, Reynaldo Martorell

**Affiliations:** 1 Department of Global Community Health and Behavioral Sciences, School of Public Health and Tropical Medicine, Tulane University, New Orleans, LA, USA; 2 Hubert Department of Global Health, Rollins School of Public Health, Emory University, Atlanta, GA, USA; 3 Department of Social Medicine, Faculty of Medicine, Universidade Federal de Pelotas, Pelotas, Brazil

**Keywords:** maternal nutrition, women's health, intrauterine growth restriction, anemia, nutrition interventions

## Abstract

**Background:**

From conception to 6 months of age, an infant is entirely dependent for its nutrition on the mother: via the placenta and then ideally via exclusive breastfeeding. This period of 15 months – about 500 days – is the most important and vulnerable in a child's life: it must be protected through policies supporting maternal nutrition and health. Those addressing nutritional status are discussed here.

**Objective and design:**

This paper aims to summarize research on policies and programs to protect women's nutrition in order to improve birth outcomes in low- and middle-income countries, based on studies of efficacy from the literature, and on effectiveness, globally and in selected countries involving in-depth data collection in communities in Ethiopia, India and Northern Nigeria. Results of this research have been published in the academic literature (more than 30 papers). The conclusions now need to be advocated to policy-makers.

**Results:**

The priority problems addressed are: intrauterine growth restriction (IUGR), women's anemia, thinness, and stunting. The priority interventions that need to be widely expanded for women before and during pregnancy, are: supplementation with iron–folic acid or multiple micronutrients; expanding coverage of iodine fortification of salt particularly to remote areas and the poorest populations; targeted provision of balanced protein energy supplements when significant resources are available; reducing teenage pregnancies; increasing interpregnancy intervals through family planning programs; and building on conditional cash transfer programs, both to provide resources and as a platform for public education. All these have known efficacy but are of inadequate coverage and resourcing. The next steps are to overcome barriers to wide implementation, without which targets for maternal and child health and nutrition (e.g. by WHO) are unlikely to be met, especially in the poorest countries.

**Conclusions:**

This agenda requires policy decisions both at Ministry and donor levels, and throughout the administrative system. Evidence-based interventions are established as a basis for these decisions, there are clear advocacy messages, and there are no scientific reasons for delay.

The ‘first 1,000 days of life’, a critical period for growth and development, has become a global rallying cry.^1^ This has resulted in greater attention to children under two, including efforts to ensure safe delivery and increase survival, and the promotion of appropriate infant and young child feeding practices. Actions have been directed more to the child than to the mother. For the first 500 of these days, from conception to about 6 months of age, the infant is entirely dependent for its nutrition on the mother: via the placenta and then ideally via exclusive breastfeeding.

Although indisputably central to early life, maternal nutrition has only recently begun to be highlighted in government or agency policies and activities (1, 2). In 2012, the World Health Assembly endorsed the WHO ‘Comprehensive implementation plan on infant and young child nutrition’ which now prioritizes women's nutrition, anemia, intrauterine growth retardation (IUGR) and low birth weight (LBW) (3, 4) (paras 2–4). Moreover, there are now global targets:

1. ‘Global target 2: by 2025, a 50% reduction of anemia in women of reproductive age’;
2. ‘Global target 3: by 2025, a 30% reduction of LBW’; and
3. ‘Global target 5: by 2025, increase the rate of exclusive breastfeeding in the first 6 months up to at least 50%’.

This paper adds its voice to the attention to the first 1,000 days. By pointing to the earliest of these days, the first 500 days, we stress the unique role of maternal nutrition in moving towards WHO's targets.

We recently carried out a landscaping study of programs addressing maternal nutrition and birth outcomes, both globally and with country case studies (Ethiopia, India, and Northeast Nigeria): these included more than 100 focus group discussions, 140 key informant interviews, plus extensive literature searching and informal discussions with officials of government, UN, and non-governmental organizations (NGOs) (5–9). We focused on three major problems: maternal anemia, IUGR, and small body size (thinness and stunting) in adult women – much the same as in the WHO plan.

Nutrition problems affect half or more of women in many low- and middle-income countries (LMICs), with disproportionate risk among the poorest (10). In LMICs overall, anemia prevalence averages 40%, affecting 600 million women, with limited progress seen in the last two decades (11, 12). Anemia prevalence in women of reproductive age exceeds 50% in many parts of India and Nigeria, and is 17% in Ethiopia (6–8). The prevalence of thinness (body mass index (BMI) <18.5) in rural women is up to 28% in Ethiopia, 41% in India, and 14% in Nigeria (13). Current estimates of short stature (<150 cm, which is −2 Standard Deviations of height-for-age) are less widely available; existing national estimates are 39% for India, 13% for Ethiopia and 3% for Nigeria (6, 8, 14).

The effects of maternal malnutrition on the developing child *in utero* are extensive. Risk of IUGR and prematurity are increased in the offspring of women who are underweight at the beginning of pregnancy (15). There is increasing evidence that inadequate fetal growth and development (i.e. IUGR) can have lifelong effects. The fetal origins of disease, described in 1986 by Barker et al. (16) and the subject of much research since then (17, 18), through impaired glucose tolerance and other paths are a risk for metabolic syndrome and premature mortality in mid-life; this suggests an increasing burden for health services in poor but rapidly changing countries, where alterations in lifestyles and diets exacerbate this effect (11). Neurocognitive and related development is at risk from ‘intrauterine and neonatal insults’, with serious long-term implications (19). IUGR precedes and contributes to stunting, now a widely used biomarker of child growth and development (11). Maternal anemia during pregnancy increases the risk of prematurity and LBW, as well as of low iron stores in newborns (20). In turn, low iron stores at birth are a risk factor for postnatal iron deficiency and anemia, which impairs motor and mental development, and learning (21). Unless this malnutrition among women is addressed, malnutrition among children will not be overcome.

Malnutrition adversely affects women's health itself. Low BMI is associated with lethargy, reduced physical activity, immunosuppression and increased risk of morbidity and mortality (22, 23). Mothers’ lactation can be affected by malnutrition, with risks that exclusive breastfeeding may not be adequate for the first 6 months of life (14). Short adult stature, which is usually due to early childhood malnutrition, is associated with poor educational performance and is a risk factor for delivery complications, cesarean deliveries, and pregnancy-related morbidity and mortality (24, 25). Anemia is estimated to cause some 20% of maternal deaths (26). Anemia also has profound consequences beyond its role in mortality, including diminished work capacity and perhaps depression (27). Women's workload during pregnancy is itself a risk for her and her child's health and nutrition, worsened when she is already malnourished. Infections in women, especially during pregnancy, are common causes of morbidity and mortality, and will be mitigated by improved nutrition.

The combination of malnutrition in mother and child perpetuates the intergenerational transmission of malnutrition. Small mothers have small babies, who may grow up to be small mothers. On the other hand, interrupting this process can create a virtuous cycle: improving maternal nutrition helps future generations to thrive (11, 24).

## Effective interventions

Through a series of systematic reviews of the literature (published as a supplement in 2012 in *Pediatric and Perinatal Epidemiology*) (28), we assessed the efficacy of 17 maternal nutrition interventions. We also evaluated the evidence on the effectiveness of maternal nutrition interventions (i.e. in large-scale operational programs) through a systematic review of published and ‘gray’ literature (noting that rigorous evaluation is rare) (5) and from the country case studies (5–8). Six priority evidence-based interventions were identified in our review that should be implemented now to improve women's nutrition and birth outcomes. These are described in Table 1, together with a summary of the evidence, application, and suggested priority. It can be seen that in most of these the efficacy is established, and there is enough evidence from large-scale programs to support wider use. In no cases are there quick fixes or magic bullets applicable: mitigating the problems needs sustained attention, through systems reaching people at the most local levels; and the capacity developed to do this.

**Table 1:** Feasible evidence-based interventions for improving women's nutrition and birth outcomes in large-scale programs

	Efficacy, based on meta-analyses	Effectiveness of large-scale programs	Applicability
**What works during and before pregnancy**			
To decrease risk of maternal anemia and intrauterine growth restriction (IUGR)/low birth weight (LBW): a) Iron or IFA or b) multiple micronutrient (MMN) supplementation c) Fortification with iron	Moderate quality evidence for impact on maternal anemia (29). MMNs as good as iron–folate for anemia (30), plus increased birth weight (31). Efficacy not assessed.	*Examples*: Nicaragua (5), Thailand, Vietnam (32). Supplementation considered successful, although worst off especially in rural areas may be missed. *Case studies*^a^ : iron supply and distribution was a major constraint at all levels; acceptability and awareness less so. Fortification of staples: mixed results.	Supplementation is universally applicable – should be early intervention in all populations in low- and middle-income countries (LMICs). Flour fortification can be expanded to more countries.
Balanced protein energy supplementation: to increase birth weight and reduce risks of IUGR/LBW and stillbirth	Moderate/high quality evidence for impact on birth weight, greater in undernourished women (33).	*Examples*: Tamil Nadu Integrated Nutrition Project; Madagascar (5). *Case studies*: used when food insecurity exists, such as periodically in Ethiopia; supplements are mandated in India, although coverage varies.	When substantial resources are available; usually targeted; requires considerable logistics.
Iodine fortification of salt (or supplementation in rare cases): to decrease risk of cretinism and improves cognition	High quality evidence for effects on cognitive development (34).	*Examples*: Thirty-six African countries have over 70% coverage of iodized salt (5). *Case studies*: in Ethiopia major supply problems, iodized oil capsules for mothers used; Nigeria high coverage; India varying implementation.	Universally applicable – should be implemented in all populations.
Conditional cash transfers: to provide cash, and a platform for education, supplement provision	Efficacy in terms of access to and use of services, nutritional status and health outcomes; may be attributed to cash or other components (35).	CCTs are implemented in an increasing number of countries, Brazil and Mexico as examples. Evidence in Mexico programme for impact on birth weight. Evidence from Brazil on reduction of infant deaths due to undernutrition. See also (36).	Provide much greater resources than other programs relevant to nutrition: when initiated should be built on.
**What works before pregnancy**			
Increasing age at first pregnancy	Moderate quality evidence that young maternal age is risk for low birth weight and preterm birth; also for maternal anemia (37).	Interventions include legislation preventing marriage before 18 years, cash incentives, outreach programs to prevent harmful traditional practices. Effectiveness not reviewed. *Case studies*: India has legislation and incentives; Ethiopia also has combatting HTP outreach program	Basic intervention in most LMICs – should have legislation and outreach.
Increasing interpregnancy interval (IPI)	Moderate quality evidence that short IPIs are linked with preterm birth, LBW, and early neonatal mortality (38).	Family planning programs, not reviewed by us.	In family planning programs.

a Case studies: see text.

Three of these interventions apply both before and during pregnancy:

1. Supplementation with iron–folic acid (IFA), or iron with other multiple micronutrients (MMNs): this should be a basic intervention in all populations with anemia, along with control of infectious diseases; effective fortification of staple foods such as wheat and corn flour, and rice, may also contribute to improving micronutrient status in different settings.
2. Iodine fortification of salt, or supplementation in rare cases.
3. Balanced protein energy supplementation: for settings of seasonal or chronic food insecurity when substantial resources and adequate logistics are available, usually targeted; these supplements should be fortified with micronutrients.

IFA supplementation during pregnancy, and sometimes before, is covered in many program guidelines. It is known to be efficacious, but effectiveness is hampered by dysfunctional systems for supply and distribution (particularly at community level), low utilization of antenatal care (ANC), a lack of community demand for IFA requiring promotion and counseling, and barriers to access (5, 13, 29). Improvements are needed in all the processes for iron supplementation, including tablet supply and logistics, at each level of implementation, and importantly in the support and counseling to mothers.

Iodine is of profound importance for fetal and postnatal growth and development. Iodized salt provision – one of the big public nutrition success stories – should be supported as a priority to achieve sustained universal coverage. Presently about 70% of households in LMICs are estimated to consume iodized salt, but this figure averages 53% for Sub-Saharan Africa (11), and is only 20% in Ethiopia and 51% in India (13). Moreover, the poorest and most remote places tend to be the least covered (5). Goiter is estimated to affect 10–15% of the population in LMICs, and inadequate urinary iodine 16–40% (11). Continued efforts are needed to adequately iodize the salt supply and put in place regulatory systems to ensure and monitor its quality and coverage.

Provision of balanced protein energy supplements may be expensive and requires developing new distribution systems, especially if targeted. However, these supplements are a critical option when women simply cannot get enough food of the right quality, to attain good nutritional status, and thus adequate nutrition for the developing child. Distribution programs are implemented with food crises (e.g. due to drought in Ethiopia), or in the long-term through routine services, for example as in Tamil Nadu, India (7). Even in the USA, where some 2 million low-income pregnant, postpartum and breastfeeding women considered to be at ‘nutritional risk’ receive vouchers to purchase foods, benefits on birth outcomes have been demonstrated (39).

Three less direct types of interventions (‘nutrition-sensitive’) that affect the nutrition of women and their children are:

1. Legislation and outreach to reduce the numbers of births to teenagers
2. Family planning programs aiming at increasing interpregnancy intervals (IPIs)
3. Conditional cash transfers.

For the first two, strategies include promotion of girls’ education, enforcement of a legal marriage age of 18 years or older and combatting related harmful traditional practices, and promotion of appropriate family planning behavior. WHO has issued detailed guidelines, including strength of evidence for recommendations (40).

Cash transfer programs are expanding rapidly (41), with the primary aim of reducing poverty. They may be conditional on ANC or nutrition education attendance. Further, they can provide a route for direct distribution of fortified food or micronutrient supplements to pregnant women. Finally, by providing the cash to the mother, they empower women to improve health care or food intake for themselves and their children (5). Although the evidence of impact of cash transfer programs on the nutrition of women and children is mixed, these are likely to become of major importance in the future and should be used as a platform for nutrition-specific interventions.

National health insurance programs, recently reviewed by UNICEF (42), at different stages of development in most LMICs will become an important route to support interventions through the health system.

## Platforms

Means of reaching women with these direct and indirect programs are known, and policy decisions that lead to building and strengthening these are essential for achieving targets. This crucial element is often overlooked; aligning interventions with their intended target groups, how to reach these, with estimates of coverage (e.g. by area) and participation (e.g. of those intended within target areas) should be highlighted in planning and monitoring. These platforms, or routes, include:

1. Through the primary health care system and ANC: these are commonly under-resourced and of limited and inequitable coverage. For instance, the percentage of pregnant women receiving at least one ANC visit, and percentage with a visit in the first trimester, was 28% and 6% in Ethiopia, and 64% and 16% in Nigeria; in India we found a range from 80% in Tamil Nadu receiving ANC in the first trimester to 19% in Bihar (7). There are also important within-country inequalities, with interventions delivered through ANC being least likely to reach mothers who are poor and undernourished (43);
2. Through community-based programs that are typically initiated for children who are often brought by their mothers: such programs are expanding and offer new opportunities – a recent review by WHO identified over 60 large-scale or national programs that have been or are being implemented in LMICs (44);
3. Campaigns, such as child health days, during which services such as immunization, deworming, and other selected interventions are provided. These approaches are most appropriate where health service outreach is very limited, and could be used more to reach women who are typically present and can receive interventions such as micronutrient supplementation; interventions delivered through campaigns tend to be more equitable than those delivered in fixed facilities (43);
4. Emergency assistance, e.g. targeting fortified food supplements to pregnant women;
5. Safety net programs, e.g. targeted cash and/or or food supplements.

These different ways of reaching women are best integrated, and opportunities built on, by strengthening existing systems and incorporating priority maternal nutrition interventions. From the case studies, for example in Ethiopia, it is seen that community-based health and nutrition platforms have expanded rapidly. Health Extension Workers and Community Health Volunteers have had substantial impact on mothers’ access to and use of health services and on child nutritional status (45). The social safety net program (PSNP) supports livelihoods, and in times of drought and food insecurity it provides targeted food assistance to women and children.

In India, a number of routes provide income and food support, and incentives for improved use of health care. For example, nutrition related interventions such as prenatal IFA and/or MMN supplementation are integrated with conditional cash transfer programs; the policy is to promote early entry into ANC and hospital delivery as part of the National Rural Health Mission (NRHM). This provides an ideal opportunity to improve maternal and child health by addressing maternal nutrition. Similarly, recent programs such as the Rajiv Gandhi Scheme for Empowerment of Adolescent Girls (SABLA) that target adolescent girls through the Integrated Child Development Services (ICDS) provide an opportunity to improve preconceptional nutritional status, as well as deliver messages related to delaying age at first birth and spacing.

Many of the above-mentioned platforms hold promise, but rigorous and external evaluations are remarkably few. Scaling up these approaches must be accompanied by proper evaluation, which should usually have prospective designs. Decisions are needed to allocate resources to careful evaluations and to facilitate these as a matter of policy, at the risk of otherwise failing to learn why some programs succeed and others fail. This is needed for management decisions on current programs, and for policy decisions on future ones.

## Policy decisions needed

Setting higher priorities and supporting programs for maternal nutrition – especially within the first 500 days – requires recognition of the need, and decisions on action at several levels. First this applies to international policy-makers and their advisors, including leaders of the ‘first 1,000 days’ movement. Other initiatives may be highly relevant, such as ‘Scaling Up Nutrition’ (SUN), the US Global Health Initiative's Feed the Future, which includes women's anemia among its indicators (46, 47), and other nutrition-specific initiatives. Then at the national level, policy decisions are needed by Ministries and Ministers of Health; President's Office or equivalent, for budget and intersectoral issues; high level planning groups (such as the India Planning Commission); and representatives of major donors and implementing agencies. These should ensure that planning, budget allocations, and intersectoral strategies give priority to meeting maternal nutrition needs.

Progress in meeting Millennium Development Goals (MDGs) and the target for child malnutrition – halving the prevalence of underweight children between 1990 and 2015 – depends considerably on the success in reaching the MDGs for women, such as targets for achieving universal access to reproductive health care and reducing the adolescent birth rate.

## What must be communicated effectively

Clear messages need to be communicated to promote policy decisions and resource commitments for planning, implementing and evaluating of large-scale programs. The following suggestions are all based on current knowledge and evidence:

1. Maternal undernutrition in pregnancy can cause restricted intrauterine growth and development, with potential lifelong effects on the child. These include increased vulnerability to mid-life diseases including diabetes and cardiovascular disease, hence premature death; and impaired neurocognitive development whose effects may be irreversible.
2. Policies need to be decided, at high levels, with resource allocations, to prioritize improving women's nutrition, birth outcomes and early infant nutrition. The necessary interventions have been implemented at scale in a number of countries, and these should be expanded and strengthened. Existing guidelines for basic health care during pregnancy, childbirth, postpartum and newborn care (48) already include these interventions, but often these are not given the same priority as those for maternal or child mortality reduction, and a more integrated approach to the provision of maternal and child health and nutrition care is warranted (49). With such an integrated approach, targets for women's and children's nutrition – notably WHO's (anemia and LBW) and MDGs (child underweight or stunting) – could be met, but not otherwise.
3. Three nutritional interventions are of high priority for better resourcing and more effective implementation at scale:
  - IFA supplementation, with infection control, for anemia;
  - further expanding iodized salt use;
  - balanced protein energy supplementation for IUGR and women's malnutrition. Important non-nutritional interventions to improve maternal and child nutrition and health include delaying age of first birth and increasing IPIs. Conditional cash transfer schemes are rapidly expanding and can improve maternal nutrition through multiple mechanisms, empowering women.
4. Key elements of successful scaling up are established from reviewing programs globally and in specific country case studies: these include enabling factors such as strong government leadership, effective coordination, adequate financing, and implementation capacity. Resources have been committed, through the health, social welfare (e.g. women and child sector in India) and other sectors, with support from donors, and decentralized implementation.
5. Scaling up must be accompanied by rigorous, external evaluations, which should ideally be planned before programs are launched, allowing baseline data to be collected. A fraction of the program budget, ideally 5–10%, should be set aside for evaluation activities.
6. International agencies and national authorities need to recognize that neither children nor women's malnutrition – especially in the first 500 days – will be overcome without these commitments. Systems must be strengthened to reach all women, giving access to effective nutrition interventions.

Now that women's nutrition is rising in priority on the international agenda, to the point where goals for women's anemia and for low birth rate reduction are being set, it is crucial to take the next steps – establishing and communicating how to do this, and fostering the necessary policy decisions and resource commitments. The evidence-based interventions described here provide a basis for effective action.

The focus on the key link between the goals for women's and for children's nutrition should be made explicit and highlighted, so that the prerequisite of good maternal nutrition for child growth is well understood and integrated into programs. The WHO goals for children – 40% reduction in stunting (global target 1), 30% reduction in LBW (target 3) – depend substantially on meeting the maternal nutrition targets (50). This includes increasing exclusive breastfeeding in the first 6 months up to at least 50% (target 5) (50).

Good health and nutrition for the mother while pregnant (9 months) and breastfeeding for 6 months (exclusively is the aim) makes this first 15 months of life – roughly 500 days – the most important for her offspring's growth and development. The results of poor nutrition in pregnancy may not be reversed, and pregnancies cannot wait for help. We need to turn the spotlight on women's needs, for their own and for their children's sake, with focus on this earliest period.

There are no shortcuts or magic bullets. There is no escaping the need to build systems that reach all women, and provide access to known interventions that will benefit women's nutrition: both for its own sake, and as an unavoidable requirement in the first 500 days of life.

## Footnotes

1 http://www.thousanddays.org/

## References

[1] Shrimpton R (2012). Global policy and programme guidance on maternal nutrition: what exists, the mechanisms for providing it, and how to improve them?. Paediatr Perinat Epidemiol.

[2] Black RE, Victora CG, Walker SP, Bhutta ZA, Christian P, de Onis M (2013). Maternal and child undernutrition and overweight in low-income and middle-income countries. Lancet.

[3] World Health Organization (2012). Comprehensive implementation plan on maternal, infant and young child nutrition – Annex 2. A65/11.

[4] World Health Organization (2012). Maternal, infant and young child nutrition. Agenda item 13.3. 26 May 2012. WHA 65.6.

[5] Victora CG, Barros FC, Assuncao MC, Restrepo-Mendez MC, Matijasevich A, Martorell R (2012). Scaling up maternal nutrition programs to improve birth outcomes: a review of implementation issues. Food Nutr Bull.

[6] Saldanha LS, Buback L, White JM, Mulugeta A, Mariam SG, Roba AC (2012). Policies and program implementation experience to improve maternal nutrition in Ethiopia. Food Nutr Bull.

[7] Ramakrishnan U, Lowe A, Vir S, Kumar S, Mohanraj R, Chaturvedi A (2012). Public health interventions, barriers, and opportunities for improving maternal nutrition in India. Food Nutr Bull.

[8] Webb GAW, Dzingina C, Akogun O, Mason JB, McFarland DA (2012). Public health interventions, barriers, and opportunities for improving maternal nutrition in Northeast Nigeria. Food Nutr Bull.

[9] Mason JB, Saldanha LS, Ramakrishnan U, Lowe A, Noznesky EA, Girard AW (2012). Opportunities for improving maternal nutrition and birth outcomes: synthesis of country experiences. Food Nutr Bull.

[10] Balarajan Y, Ramakrishnan U, Ozaltin E, Shankar AH, Subramanian SV (2011). Anaemia in low-income and middle-income countries. Lancet.

[11] UN-SCN (2010). 6th Report on the world nutrition situation.

[12] Stevens G, Finucane M, De-Regil L, Paciorek C, Flaxman S, Branca F (2013). Global, regional, and national trends in haemoglobin concentration and prevalence of total and severe anaemia in children and pregnant and non-pregnant women for 1995–2011: a systematic analysis of population–representative data. Lancet Glob Health.

[13] Mason JB, Saldanha LS, Ramakrishnan U, Lowe A, Noznesky EA, Girard AW (2012). Opportunities for improving maternal nutrition and birth outcomes: synthesis of country experiences. Food Nutr Bull.

[14] Martorell R, Young MF (2012). Patterns of stunting and wasting: potential explanatory factors. Adv Nutr.

[15] Ramakrishnan U, Grant F, Goldenberg T, Zongrone A, Martorell R (2012). Effect of women's nutrition before and during early pregnancy on maternal and infant outcomes: a systematic review. Paediatr Perinat Epidemiol.

[16] Barker DJ, Osmond C (1986). Infant mortality, childhood nutrition, and ischaemic heart disease in England and Wales. Lancet.

[17] Hales CN (1997). Fetal and infant origins of adult disease. J Clin Pathol.

[18] Barker DJ, Gelow J, Thornburg K, Osmond C, Kajantie E, Eriksson JG (2010). The early origins of chronic heart failure: impaired placental growth and initiation of insulin resistance in childhood. Eur J Heart Fail.

[19] Mwaniki MK, Atieno M, Lawn JE, Newton CR (2012). Long-term neurodevelopmental outcomes after intrauterine and neonatal insults: a systematic review. Lancet.

[20] Scholl TO, Reilly T (2000). Anemia, iron and pregnancy outcome. J Nutr.

[21] Beard JL (2008). Why iron deficiency is important in infant development. J Nutr.

[22] Shetty PS, James W (1994). Body mass index. A measure of chronic energy deficiency in adults.

[23] Whitlock G, Lewington S, Sherliker P, Clarke R, Emberson J, Halsey J (2009). Body-mass index and cause-specific mortality in 900 000 adults: collaborative analyses of 57 prospective studies. Lancet.

[24] Victora CG, Adair L, Fall C, Hallal PC, Martorell R, Richter L (2008). Maternal and child undernutrition: consequences for adult health and human capital. Lancet.

[25] Merchant KM, Villar J, Kestler E (2001). Maternal height and newborn size relative to risk of intrapartum caesarean delivery and perinatal distress. BJOG.

[26] Bhutta ZA, Das JK, Rizvi A, Gaffey MF, Walker N, Horton S (2013). Evidence-based interventions for improvement of maternal and child nutrition: what can be done and at what cost?. Lancet.

[27] Stoltzfus RJ (2011). Iron interventions for women and children in low-income countries. J Nutr.

[28] Imhoff-Kunsch B, Martorell R (2012). Improving maternal, newborn, and child health outcomes through better designed policies and programs that enhance the nutrition of women. Paediatric Perinat Epidemiol.

[29] Imdad A, Bhutta ZA (2012). Routine iron/folate supplementation during pregnancy: effect on maternal anaemia and birth outcomes. Paediatr Perinat Epidemiol.

[30] Bhutta ZA, Imdad A, Ramakrishnan U, Martorell R (2012). Is it time to replace iron folate supplements in pregnancy with multiple micronutrients?. Paediatr Perinat Epidemiol.

[31] Ramakrishnan U, Grant FK, Goldenberg T, Bui V, Imdad A, Bhutta ZA (2012). Effect of multiple micronutrient supplementation on pregnancy and infant outcomes: a systematic review. Paediatr Perinat Epidemiol.

[32] Mason J, Deitchler M, Mathys E, Winichagoon P, Tuazon MA (2004). Lessons from successful micronutrient programs. Part III: program impact. Food Nutr Bull.

[33] Imdad A, Bhutta ZA (2012). Maternal nutrition and birth outcomes: effect of balanced protein–energy supplementation. Paediatr Perinat Epidemiol.

[34] Zimmermann MB (2012). The effects of iodine deficiency in pregnancy and infancy. Paediatr Perinat Epidemiol.

[35] Lagarde M, Haines A, Palmer N (2009). The impact of conditional cash transfers on health outcomes and use of health services in low and middle income countries. Cochrane Database Syst Rev.

[36] Rasella D, Aquino R, Santos CA, Paes-Sousa R, Barreto ML (2013). Effect of a conditional cash transfer programme on childhood mortality: a nationwide analysis of Brazilian municipalities. Lancet.

[37] Gibbs CM, Wendt A, Peters S, Hogue CJ (2012). The impact of early age at first childbirth on maternal and infant health. Paediatr Perinat Epidemiol.

[38] Wendt A, Gibbs CM, Peters S, Hogue CJ (2012). Impact of increasing inter-pregnancy interval on maternal and infant health. Paediatr Perinat Epidemiol.

[39] Taylor YJ, Nies MA (2013). Measuring the impact and outcomes of maternal child health federal programs. Matern Child Health J.

[40] World Health Organization (2011). Preventing early pregnancy and poor reproductive outcomes among adolescents in developing countries.

[41] Bassett L (2008). Can conditional cash transfer programs play a greater role in reducing child undernutrition?.

[42] UNICEF (2012). National health insurance in Asia and Africa advancing equitable social health protection to achieve universal health coverage.

[43] Barros AJ, Ronsmans C, Axelson H, Loaiza E, Bertoldi AD, Franca GV (2012). Equity in maternal, newborn, and child health interventions in Countdown to 2015: a retrospective review of survey data from 54 countries. Lancet.

[44] World Health Organization (2013). Essential nutrition actions: improving maternal, newborn, infant and young child health and nutrition.

[45] White JM, Mason JB (2012). Assessing the impact on child nutrition of the Ethiopia Community-based Nutrition Program. Report to UNICEF of an evaluation study.

[46] SUN Road Map Task Team (2010). Roadmap for Scaling Up Nutrition. http://www.un-foodsecurity.org/node/768.

[47] U.S. Government (2012). Feed the future indicator handbook: definition sheets.

[48] World Health Organization (2006). Pregnancy, childbirth, postpartum and newborn care: a guide for essential practice.

[49] World Health Organization (2006). Promoting optimal fetal development: report of a technical consultation. Geneva.

[50] World Health Organizationa (2012). Comprehensive implementation plan on maternal, infant and young child nutrition.

# Sub article: Intimate partner sexual and physical violence among women in Togo, West Africa: Prevalence, associated factors, and the specific role of HIV infection

**DOI:** 10.3402/gha.v7.23456

**Published:** 2014-05-26

**Authors:** Juan Burgos-Soto, Joanna Orne-Gliemann, Gaëlle Encrenaz, Akouda Patassi, Aurore Woronowski, Benjamin Kariyiare, Annette K. Lawson-Evi, Valériane Leroy, François Dabis, Didier K. Ekouevi, Renaud Becquet

**Affiliations:** 1 INSERM, Centre INSERM U897 ‘Epidémiologie et Biostatistique’, Bordeaux, France; 2 Université de Bordeaux, Institut de Santé Publique Epidémiologie Développement (ISPED), Bordeaux, France; 3 COMPTRASEC, CNRS UMR 5114, Pessac, France; 4 Service des maladies infectieuses, Centre Hospitalier Universitaire Sylvanus Olympio, Lomé, Togo; 5 Département de Santé Publique, Faculté mixte de médecine et pharmacie, Lomé, Togo; 6 Service de pédiatrie, Centre Hospitalier Universitaire Sylvanus Olympio, Lomé, Togo

**Keywords:** intimate partner violence, gender, HIV infection, Africa

## Abstract

**Background:**

A substantial proportion of newly diagnosed HIV infections in sub-Saharan Africa occur within serodiscordant cohabiting heterosexual couples. Intimate partner violence is a major concern for couple-oriented HIV preventive approaches. This study aimed at estimating the prevalence and associated factors of intimate partner physical and sexual violence among HIV-infected and -uninfected women in Togo. We also described the severity and consequences of this violence as well as care-seeking behaviors of women exposed to intimate partner violence.

**Methods:**

A cross-sectional survey was conducted between May and July 2011 within Sylvanus Olympio University Hospital in Lomé. HIV-infected women attending HIV care and uninfected women attending postnatal care and/or children immunization visits were interviewed. Intimate partner physical and sexual violence and controlling behaviors were assessed using an adapted version of the *WHO Multi-country study on Women’s Health and Life Events* questionnaire.

**Results:**

Overall, 150 HIV-uninfected and 304 HIV-infected women accepted to be interviewed. The prevalence rates of lifetime physical and sexual violence among HIV-infected women were significantly higher than among uninfected women (63.1 vs. 39.3%, p<0.01 and 69.7 vs. 35.3%, p<0.01, respectively). Forty-two percent of the women reported having ever had physical injuries as a consequence of intimate partner violence. Among injured women, only one-third had ever disclosed real causes of injuries to medical staff and none of them had been referred to local organizations to receive appropriate psychological support. Regardless of HIV status and after adjustment on potential confounders, the risk of intimate partner physical and sexual violence was strongly and significantly associated with male partner multi-partnership and early start of sexual life. Among uninfected women, physical violence was significantly associated with gender submissive attitudes.

**Discussion and conclusions:**

The prevalence rates of both lifetime physical and sexual violence were very high among HIV-uninfected women and even higher among HIV-infected women recruited in health facilities in this West African country. Screening for intimate partner violence should be systematic in health-care settings, and specifically within HIV care services. At a time of increased investments in couple-oriented HIV prevention interventions, further longitudinal research to better understanding of HIV-serodiscordant couple dynamics in terms of intimate partner violence is needed.

According to the World Health Organization (WHO), intimate partner violence is defined as the behavior, within an intimate relationship, that causes physical, sexual, or psychological harm or suffering (1). Several population-based surveys have reported that among all forms of violence against women, intimate partner violence is the most prevalent and it is considered the most common human rights violation hitherto (2, 3). Although there is still insufficient consensus on the operational definition and measurement of intimate partner violence, available estimations of lifetime prevalence of intimate partner violence vary from 15 to 71% worldwide, with the highest rates documented in resource-constrained settings (4). Intimate partner violence is associated with poor health outcomes among victims and is therefore a major public health issue (5–7).

Besides its dramatic physical and mental consequences, intimate partner violence has also been identified as an important risk factor for sexually transmitted diseases acquisition and particularly HIV infection (8). Intimate partner violence and HIV infection are linked by a very complex association, including diverse pathways. An increased risk of HIV transmission within abusive relationships and a greater likelihood of acquisition of HIV infection by violent husbands have been reported (8, 9). Acts derived from intimate partner violence, such as coerced sexual intercourse, during which women are unable to protect themselves from transmission, place women at direct risk of HIV infection (10).

Sub-Saharan Africa is the region reporting the highest epidemiological burden of HIV worldwide, with women accounting for 58% of newly acquired HIV infections (11). Furthermore, HIV infection in these settings spreads principally through heterosexual transmission and a substantial proportion of newly diagnosed infections occur in HIV-discordant cohabiting couples in which only one person is living with HIV (12). HIV prevention interventions aiming at protecting both couple members are thus a public health priority to reduce HIV incidence in these regions and several approaches have been already proposed (10–14). In spite of their proven efficacy, the challenges in implementing couple-oriented HIV preventive approaches are tremendous, and most importantly because they are based on mutual disclosure of HIV serological status (14–17). Intimate partner violence as one important consequence of HIV serological disclosure is thus one major limit of couple-oriented HIV preventive approaches and understanding its magnitude, dynamics, and factors associated are a prerequisite to the roll out of these couple strategies (1, 10, 12, 13).

In Togo, a West African country with an estimated prevalence rate of HIV infection of 3.4% nationwide and a predominantly heterosexual epidemic (11), intimate partner violence among women and its association with HIV infection has never been studied so far using a quantitative methodological approach, to the best of our knowledge. Moreover, data on intimate partner violence according to serological status in the West African region are very scarce, although it is needed to improve the scaling-up of HIV prevention, care, and treatment programs. This study aimed at estimating the prevalence and associated factors of intimate partner physical and sexual violence among HIV-infected and -uninfected women of childbearing age attending a clinical facility in Togo. We also described the severity and consequences of this violence as well as care-seeking behaviors of women exposed to intimate partner violence.

## Methods

### Study design and population

This project was an international collaboration between the INSERM (National Institute of Health and Medical Research) Research Centre U897 based in Bordeaux (France), the Sylvanus Olympio University Hospital in Lomé, and the non-governmental organization *Espoir Vie Togo*, one leading organization providing care and psychological support to people living with HIV/AIDS in Togo.

A cross-sectional survey was conducted between May and July 2011 within the Lomé University Hospital. The study population was constituted by volunteer participants attending clinical services in the hospital. HIV-infected women attending regular HIV care visits constituted the first group. Women attending postnatal care and/or children immunization visits, and who had been diagnosed HIV-uninfected during their last pregnancy, constituted the second group. For these two study groups, women aged at least 18 years, declaring having a current intimate partner or having ever had one, were eligible to recruitment.

### Study procedures

The survey was conducted in three stages. The first preparatory phase consisted of a methodological workshop between Togolese and French researchers to discuss the study design, validate the survey instruments, and address relevant ethical issues. A local team of four psychologists was recruited to adapt concepts described in the questionnaires to the local context, train interviewers on the study tool, ethical and privacy terms, and supervise data collection.

Second, a pilot study was carried out to test the acceptability, adequacy, and understandability of the recruitment procedures and questionnaire. Survey feasibility data and interviewer’s observations were integrated within the final survey procedures and tools.

Finally, every working day during the study period, each eligible woman was systematically asked to participate in the survey and to provide signed informed consent to be sequentially enrolled. Trained female health-care staff administered the questionnaire during a 20-min face-to-face interview in a private room.

All interviews were carried out in French, and all participants were provided information about local support services dedicated to women experiencing intimate partner violence. HIV-infected women were informed about the psychological support available at *Espoir vie Togo*.

The study protocol was approved by the National Ethic Committee of the Ministry of Health of Togo (N°0125/2011/MS/CAB/DGS/DPLET/CBRS). Written informed consent was obtained from all participants; and all data collection tools were strictly anonymous.

### Intimate partner physical and sexual violence assessment

Our questionnaire assessing intimate partner physical and sexual violence and controlling behaviors was largely inspired by the *WHO Multi-Country Study on Women’s Health and Life Events* questionnaire (version 10) (4, 18, 19).

All women participating in the survey were asked about their life experience on specific acts of physical and sexual violence induced by their intimate partner. Physical violence was defined as the use of physical force causing bodily harm (1). Within the survey, women were also asked about physical consequences of physical violent acts induced by an intimate partner, as well as help-seeking behaviors. Sexual violence was defined as any situation where women faced forced or coerced sexual act or attitude (1). Prevalence of physical/sexual violence was estimated as the proportion of women declaring having been exposed to any kind of physical or sexual violent act by their current intimate partner or any previous partner.

Additionally, we explored attitudes toward partner controlling behaviors that we summarized in six statements, illustrating different gender submissive situations to which women were asked to agree or not. We developed a scoring system to define a ‘submission index’ summarizing women’s attitudes toward partner controlling behaviors based on the sum of the number of positive answers to six questions detailed in Table 1.

**Table 1:** Characteristics of women interviewed according to their HIV status: Lomé, Togo, May–July 2011

	HIV uninfected (N=150)		HIV infected (N=304)		
		
	*n*	*%*	*N*	*%*	*p*
Women’s sociodemographic profile					
Age					
≥33 y/o	55	36.7	185	60.9	<0.01
<33 y/o	95	63.3	119	39.1	
Instruction level					
Not instructed	8	5.3	49	16.1	0.01
At least primary level	142	94.7	255	83.9	
Contraceptive method^a^					
Yes	97	64.7	128	42.1	<0.01
No	53	35.3	176	57.9	
Financial autonomy					
Employment					
Yes	103	68.7	242	79.6	0.01
No	47	31.3	62	20.4	
Financial autonomy to support herself^b^					
Yes	59	39.3	170	56.0	<0.01
No	91	60.7	134	44.0	
Modalities of entry to sexual life					
Age of first sexual intercourse					
≥18 y/o	63	42.0	113	37.2	0.32
<18 y/o	87	58.0	191	62.8	
Conditions of first sexual intercourse					
Consented	121	80.7	209	69.4	0.01
Coerced	29	19.3	92	30.6	
Women’s mental health					
Loss of interest^c^					
Yes	103	68.7	225	74.0	0.23
No	47	31.3	79	26.0	
Suicidal thoughts^d^					
Yes	36	24.0	157	51.6	<0.01
No	114	76.0	147	48.4	
Suicidal attempts^d^					
Yes	8	5.3	23	7.6	0.37
No	142	94.7	281	92.4	
Partners profile					
Polygamous					
Yes	19	12.7	138	45.4	<0.01
No	131	87.3	166	54.6	
Concurrent relationships					
Yes	52	34.7	203	66.8	<0.01
No	98	65.3	101	33.2	
Alcohol consumption					
Never/occasionally	85	56.7	125	41.0	0.01
Frequently	65	43.3	179	59.0	
Frequently involved in fights/riots					
Yes	11	7.3	65	21.4	<0.01
No	139	92.7	239	78.6	
History of non-partner violence					
Physical violence after 15 y/o					
Yes	60	40.0	200	65.8	<0.01
No	90	60.0	104	34.2	
Sexual violence after 15 y/o					
Yes	1	0.7	11	3.6	0.06
No	149	99.3	293	96.4	
Sexual violence before 15 y/o					
Yes	6	4.0	41	13.5	0.01
No	144	96.0	263	86.5	
Controlling behaviors and submission index					
A good wife obeys her partner, even if she does not agree with him					
Yes	113	75.3	278	91.5	<0.01
No	37	24.67	26	8.55	
It is important that a man shows his wife who is the boss?					
Yes	104	69.3	240	78.9	0.02
No	46	30.7	64	21.0	
A woman may have the freedom to choose her friends, even if her partner does not agree?					
Yes	19	12.7	49	16.1	0.33
No	131	87.3	255	83.8	
Satisfying her husband’s sexual desire even if she does not want to is a women’s duty?					
Yes	46	30.7	151	49.7	<0.01
No	104	69.3	153	50.3	
If a man abuses his wife, people around must intervene?					
Yes	122	81.3	240	78.8	
No	28	18.6	64	21.1	
A man must strike his wife if he considers this necessary?					
Yes	49	32.7	174	57.2	<0.01
No	101	67.3	130	42.7	
Submission index					
Median	3		4		<0.01

a Use of any contraceptive method at the moment of the survey.

b Having a financial autonomy to support herself and household without her partner for at least 1 month.

c During at least two weeks over the last 12 months.

d At least once over the last 12 months.

We also documented women’s sociodemographic characteristics (age, instruction level, and contraceptive use), data on the women’s partner (alcohol consumption and frequency of involvement in fights) and couple relationships (polygamy and concurrent relationship), women’s employment and financial autonomy (having a financial autonomy to support herself and household without her partner for at least 1 month), and the modalities of women’s entry in sexual life (conditions and age at first sexual intercourse). We also investigated women’s mental health (loss of interest, suicidal thoughts, and suicidal attempts) and the history of non-partner violence (physical violence after 15 years and sexual violence before and after 15 years).

### Statistical analysis

Sociodemographical and psychosocial characteristics of women and partners were described and compared between HIV-infected and -uninfected women. Maternal age was dichotomized into two categories with a cut-off defined by the median in the overall population (e.g. 33 years). Age of first sexual intercourse was dichotomized into two categories with a cut-off defined by the age at sexual majority in Togo (e.g. 18 years). The prevalence of lifetime physical and sexual violence was estimated separately among HIV-infected and -uninfected women and also compared between the two groups. Chi-square tests were performed to determine statistically significant differences. Sociodemographic and behavioral factors associated with lifetime physical and sexual violence in both groups were then assessed using logistic regression. Both univariate and multivariate analyses were carried out. Variables found to be statistically associated with intimate partner violence with a *p*-value of <0.25 were included in the multivariable model. To select the final adjusted model presented in this paper, we used a backward elimination method using a *p*-value of 0.05 (20). Adjusted odds ratios (aORs) were estimated using multiple logistic regression modeling and statistical significance was considered at the 5% level. Statistical analyses were generated using SAS software (version 9.2 for Windows, Copyright 2013 for SAS Institute Inc., Cary, NC, USA).

## Results

Overall, 454 women attending the Sylvanus Olympio University Hospital were informed about the study and screened for eligibility; all of them accepted to be interviewed and were included in the study. One hundred and fifty women were HIV-negative and 304 were HIV-positive.

### Study population characteristics

Sociodemographical and behavioral characteristics of women in both groups are described in Table 1 and summarized as follows.

Concerning women’s sociodemographic profile and financial autonomy, HIV-infected women were significantly older than uninfected women (35 [32–37] and 30 [29–34] years old in median [interquartile range], respectively, *p*<0.01). The proportion of HIV-uninfected women having completed at least primary education was significantly higher than among HIV-positive women (94.7 vs. 83.9%, *p*=0.01). HIV-infected women were significantly more likely than uninfected women to report some degree of financial autonomy (56 vs. 39.3%, *p*<0.001). HIV-uninfected women were more likely to be using a contraceptive method at the time of the survey than HIV-infected women (64.7 vs. 42.1%, *p*≤0.001).

Concerning modalities of entry to sexual life, the proportion of women having entered sexual life before the age of 18 was higher among HIV-infected than HIV-negative women (62.8 vs. 58.0%, *p*=0.32). HIV-infected women were significantly more likely to start sexual life through a first forced sexual intercourse than uninfected ones (30.6 vs. 19.3%, *p*=0.01).

Concerning partner’s profile characteristics, HIV-infected women were more likely to live within polygamous households than uninfected women (45.4 vs. 12.7%, *p*<0.001) and to declare that their partner had concurrent relationships out of the household (66.8 vs. 34.7%, *p*<0.001). Reported rates of partner alcohol consumption (59.0 vs. 43.3%, *p*=0.01) and partner involvement in fights and riots (21.4 vs. 7.3%, *p*<0.001) were more frequent among HIV-infected women than uninfected women.

Concerning women’s mental health, suicidal thoughts during the past 12 months were significantly more frequent among HIV-infected women than among the uninfected ones (51.6 vs. 24.0%, *p*<0.001). The proportion of HIV-infected women reporting loss of interest (74.0 vs. 68.7%; *p*=0.23) and suicidal attempts (7.6 vs. 5.3%, *p*=0.37) during the past 12 months tended to be higher than among uninfected ones but differences were not statistically significant.

Finally, HIV-infected women were slightly more likely to agree to submissive statements than HIV-uninfected women (scoring of 4/6 and 3/6, respectively; *p*<0.001).

### Intimate partner violence: prevalence, severity, and care-seeking behaviors

As detailed in Table 2, the prevalence rate of lifetime physical violence among HIV-infected women was 63.1% (95% CI: 57.5–68.4), significantly higher than among uninfected women (39.3%; 95% CI: 31.1–46.8; *p*<0.01). Similarly, HIV-infected women reported a significantly higher prevalence rate of lifetime sexual violence compared to the uninfected ones (69.7%; 95% CI: 63.8–74.1 vs. 35.3%; 95% CI: 27.3–42.6; *p<*0.01). The lifetime prevalence rate of both types of violence combined (physical and sexual violence) was 51.6% (95% CI: 45.3–56.6) among HIV-infected women and significantly higher than among uninfected women (18.6%; 95% CI: 11.8–24.1; *p<*0.01; Table 2).

**Table 2:** Lifetime prevalence rates of intimate partner vionce) among women, according to their HIV status: Lomé, Togo, May–July 2011

	HIV uninfected, *N*=150			HIV infected, *N*=304		
			
	*n*	%	95% CI	*n*	%	95% CI
Any form of physical violence						
Yes	59	39.3	31.1–46.8	192	63.2	57.5–68.4
No	91	60.7	52.1–67.8	112	36.8	30.6–41.3
Any form of sexual violence						
Yes	53	35.3	27.3–42.6	212	69.7	63.8–74.1
No	97	64.7	56.3–71.6	92	30.3	24.8–35.1
Any form of physical and sexual violence combined						
Yes	28	18.7	11.8–24.1	157	51.6	45.3–56.6
No	122	81.3	74.7–87.2	147	48.4	42.3–53.6

HIV-infected women were more likely to report a history of physical violence after the age of 15 than HIV-uninfected women (65.8 vs. 40.0%; *p*<0.001) and the frequency of sexual violence during childhood (before 15 years old) was 13.5% among HIV-infected versus 4.0% among uninfected ones (*p*=0.01; Table 1).

Among women ever victims of intimate partner physical violence (*n*=251), 194 (77.2%) reported physical injuries as a consequence of this violence (Table 3). Most commonly reported injuries were scratches and bruises (80.9%), dislocation and sprains (62.9%), eardrums rupture and black eyes (54.6%), penetration injuries and deep cuts (28.9%), and gashes and bites (20.1), although less commonly reported were burns (5.7%), fractures (4.6%), and broken tooth (1.6%). There were no differences between the type of injuries reported by HIV-infected and -uninfected women (data not shown).

**Table 3:** Distribution of physical injuries and care-seeking cascade reported by women victims of physical violence: Lomé, Togo, May–July 2011

	N	%
Number of physically injured women among those victims of physical violence (n=251)	194	77.2
Types of physical injuries reported (n=194)		
Scratches, hematomas	157	80.9
Dislocation, sprains	122	62.9
Eardrums rupture, black eyes	106	54.6
Penetration injuries, deep cuts, gashes	56	28.9
Bites	39	20.1
Burns	11	5.7
Fractures, broken bones	9	4.6
Broken teeth	3	1.6
Hospitalizations	14	7.2
Number of injured women needing medical care after being injured (n=194)	160	82.4
Number of injured women having received medical care when injured (n=160)	93	58.1
Number of women that told medical personnel the real cause of injuries (n=93)	49	52.6
Number of women referred to a dedicated support group (n=49)	0	0.0

Among the 194 women reporting being injured by intimate partner violence, 160 (82.4%) reported needing medical care for their injuries. From those needing medical care, 93 (58.1%) received medical care and 14 (7.2%) were even hospitalized. Among injured women in care, 49 (52.6%) disclosed real causes of injuries to medical staff and none of them was referred to local organizations to receive appropriate psychological support (Table 3).

### Factors associated with physical violence

The only common factor associated with a history of intimate partner physical violence regardless of women’s serological status was having a partner maintaining concurrent relationships out of the household (HIV-uninfected: aOR: 2.5; 95% CI: 1.1–5.5; *p*=0.02, and HIV infected: aOR: 2.2; 95% CI: 1.3–3.6; *p*<0.001). Otherwise, the profile of HIV-infected and HIV-negative women reporting intimate partner physical violence was different (Table 4).

**Table 4:** Factors associated with intimate partner physical violence according to their HIV status: Lomé, Togo, May–July 2011

	HIV uninfected						HIV infected					
		
	OR	95% CI	*p*	aOR	95% CI	*p*	OR	95% CI	*p*	aOR	95% CI	*p*
Women’s sociodemographic profile												
Age												
>33 y/o	1			1			1					
<33 y/o	2.02	0.99–4.09	0.05	0.39	0.17–0.88	0.02	0.93	0.58–1.50	0.78			
Education												
Not instructed	1						1			1		
At least primary level	1.58	0.38–6.58	0.53				1.99	0.99–3.99	0.05	2.05	1.00–4.19	0.05
Contraceptive methods^a^												
Yes	1			1			1					
No	1.65	0.83–3.27	0.15	2.28	1.03–5.01	0.04	1.05	0.65–1.68	0.84			
Modalities of entry to sexual life												
Age of first sexual intercourse												
>18 y/o	1			1			1					
<18 y/o	0.45	0.22–0.89	0.02	0.48	0.21–1.03	0.06	0.77	0.47–1.24	0.28			
Conditions of first sexual intercourse												
Consented	1			1			1					
Coerced	2.67	1.16–6.10	0.02	2.65	1.06–6.58	0.04	1.74	1.02–2.95	0.04			
Mental health												
Loss of interest^b^												
No	1						1					
Yes	1.07	0.52–2.16	0.86				2.90	1.71–4.91	<0.0001			
Suicidal thoughts^c^												
No	1						1					
Yes	1.53	0.72–3.27	0.27				1.75	1.09–2.80	0.02			
Suicidal attempts^c^												
No	1						1			1		
Yes	2.72	0.62–11.82	0.18				4.22	1.22–14.54	0.02	4.53	1.29–15.91	0.02
Partners profile												
Polygamous												
No	1						1					
Yes	1.86	0.70–4.89	0.21				1.48	0.92–2.37	0.1			
Concurrent relationships												
No	1			1			1			1		
Yes	2.86	1.42–5.73	0	2.51	1.13–5.52	0.02	2.37	1.45–3.88	0	2.21	1.33–3.65	<0.001
Alcohol consumption												
Never/occasionally	1			1			1					
Frequently	2.08	1.06–4.05	0.03	1.74	0.81–3.71	0.15	1.68	1.04–2.69	0.03			
Involved in fights/riots												
No	1						1					
Yes	4.6	1.16–18.12	0.03				2.06	1.10–3.82	0.02			
History of non-partner violence												
Physical violence after 15 y/o												
No	1						1			1		
Yes	0.93	0.47–1.82	0.84				1.51	0.93–2.46	0.09	1.54	0.92–2.56	0.1
Sexual violence after 15 y/o^d^												
Yes							1					
No							1.58	0.41–6.07	0.51			
Sexual violence before 15 y/o												
No	1						1					
Yes	8.33	0.94–73.22	0.06				1.7	0.81–3.54	0.16			
Financial autonomy												
Employment												
No	1						1					
Yes	0.64	0.31–1.28	0.21				0.93	0.51–1.66	0.8			
Financial autonomy to support herself^e^												
No	1						1					
Yes	0.77	0.39–1.51	0.45				0.92	0.57–1.47	0.74			
Submission index												
	1.18	0.89–1.53	0.24	1.33	0.97–1.80	0.07	1.13	0.91–1.39	0.28			

a Use of any contraceptive method at the moment of the survey.

b During at least two weeks for the last 12 months.

c At least once during last 12 months.

d Not enough subjects for the analysis among uninfected women.

e Having a financial autonomy to support herself and household without her partner for at least 1 month.

Among women’s sociodemographic and sexual characteristics, age was associated with physical violence only for HIV-uninfected women (33 years old or below vs. older than 33 years: aOR: 0.4; 95% CI: 0.2–0.9; *p*=0.02), and education level only for HIV-infected women (at least primary level vs. never attended school: aOR: 2.0; 95% CI: 1.0–4.2; *p*=0.05). Uninfected women not using any contraceptive method at the time of the survey were more likely to be victims of intimate partner physical violence (aOR: 2.3; 95% CI: 1.0–5.0; *p*=0.04). Among uninfected women, the odds of intimate partner sexual violence were significantly higher among those reporting a first coerced sexual intercourse (aOR: 2.6; 95% CI: 1.1–6.6; *p=*0.04).

In terms of women’s mental health status, having ever attempted suicide was strongly associated with a history of intimate partner physical violence, for HIV-infected women only (aOR: 4.5; 95% CI: 1.3–15.9; *p=*0.02). In the univariate analysis, loss of interest during at least two weeks (OR: 2.9; 95% CI: 1.7–4.9; *p<*0.001) and having had suicidal thought at least once over the last 12 months (OR: 1.7; 95% CI: 1.1–2.8; *p*=0.02) were also associated with having experienced intimate physical violence among HIV-infected women.

### Factors associated with sexual violence

As detailed in Table 5, starting sexual life before the age of 18 appeared to be the only common factor associated with intimate partner sexual violence among both HIV-infected and -uninfected women (HIV-infected women: aOR: 2.3; 95% CI: 1.1–4.9; *p*=0.01, and uninfected women: aOR: 2.3; 95% CI: 1.2–4.4; *p*=0.03).

**Table 5:** Factors associated with intimate partner sexual violence according to their HIV status: Lomé, Togo, May–July 2011

	HIV-uninfected						HIV-infected					
		
	OR	95% CI	*p*	aOR	95% CI	*p*	OR	95% CI	*p*	aOR	95% CI	*p*
Women’s sociodemographic profile												
Age												
>33 y/o	1						1					
<33 y/o	0.93	0.46–1.86	0.84				0.68	0.41–1.11	0.13			
Education												
Not instructed	1						1					
At least primary level	1.1	0.25–4.81	0.9				1.41	0.69–2.86	0.34			
Contraceptive methods^a^												
Yes	1						1					
No	0.7	0.34–1.43	0.33				1.23	0.75–2.01	0.41			
Modalities of entry to sexual life												
Age of first sexual intercourse												
>18 y/o	1			1			1			1		
<18 y/o	2.84	1.37–5.89	0.01	2.31	1.08–4.92	0.03	1.78	1.08–2.93	0.02	2.28	1.19–4.37	0.01
Conditions of first sexual intercourse												
Consented	1			1			1					
Coerced	2.34	1.02–5.33	0.04	2.18	0.92–5.18	0.08	2.87	1.54–5.34	<0.001			
Mental health												
Loss of interest^b^												
No	1						1					
Yes	1.93	0.89–4.14	0.09				7.51	4.26–13.24	<0.001			
Suicidal thoughts^c^												
No	1						1			1		
Yes	1.67	0.77–3.57	0.19				2.21	1.33–3.64	0	1.98	1.16–3.38	0.01
Suicidal attempts^c^												
No	1						1					
Yes	1.9	0.45–7.91	0.38				3.09	0.89–10.66	0.07			
Partners profile												
Polygamous												
No	1						1					
Yes	1.08	0.39–2.92	0.88				1.54	0.93–2.53	0.09			
Concurrent relationships												
No	1			1			1					
Yes	2.63	1.30–5.30	0.01	2.38	1.14–4.94	0.02	2.67	1.60–4.44	<0.001			
Alcohol consumption												
Never/occasionally	1						1					
Frequently	0.7	0.35–1.38	0.31				1.58	0.96–2.59	0.07			
Involved in fights/riots												
No	1						1			1		
Yes	2.35	0.68–8.09	0.18				2.87	1.39–5.93	<0.001	2.6	1.23–5.51	0.01
History of non-partner violence												
Physical violence after 15 y/o												
No	1						1					
Yes	0.46	0.22–0.93	0.03				2.84	1.70–4.72	<0.001			
Sexual violence after 15 y/o^d^												
Yes							1					
No							4.51	0.56–35.71	0.15			
Sexual violence before 15 y/o												
No	1						1					
Yes	3.88	0.68–21.91	0.13				2.83	1.14–6.99	0.02			
Financial autonomy^e^												
Employment												
No	1						1					
Yes	0.64	0.31–1.29	0.21				1.35	0.74–2.43	0.32			
Financial autonomy to support herself^a^												
No	1						1					
Yes	0.68	0.32–1.39	0.29				0.7	0.42–1.15	0.16			
Submission index												
	1.05	0.80–1.37	0.72				1.66	1.30–2.10	<0.001	1.58	1.23–2.03	<0.001

a Use of any contraceptive method at the moment of the survey.

b During at least two weeks for the last 12 months.

c At least once during last 12 months.

d Not enough subjects for the analysis among uninfected women.

e Having a financial autonomy to support herself and household without her partner for at least 1 month.

Otherwise, the profile of women reporting intimate partner sexual violence differed according to HIV serological status. HIV-uninfected women reporting having partners maintaining concurrent relationships out of household were more likely to report intimate partner sexual violence (aOR: 2.4; 95% CI: 1.1–4.9; *p*=0.02). For HIV-infected women, intimate partner sexual violence was associated with reporting suicidal thoughts (aOR: 1.9; 95% CI: 1.2–3.4; *p*=0.01), having partners who were involved in fights and/or riots with other men (aOR: 2.6; 95% CI: 1.2–5.5; *p*=0.01), and reporting a higher submission index (aOR: 1.6; 95% CI: 1.2–2.0; *p*<0.001).

## Discussion

We reported here on the prevalence of lifetime intimate partner physical and sexual violence according to HIV status among women recruited in health facilities in Togo. Our main finding is that the prevalence rates of both lifetime physical and sexual violence in 2011 were very high among HIV-uninfected women and even higher among HIV-infected women in this West African country. The prevalence rates we documented among uninfected women are similar to those estimated using the same methodology within population-based surveys from East Africa, both in terms of physical violence (32–49%) and sexual violence (23–58%) (4). On the contrary, more than half (63.1%) of the HIV-infected women in Togo reported lifetime intimate partner physical violence, which is almost twice as high than among uninfected women interviewed (39.3%) and much higher than the rates observed among HIV-infected women in Nigeria (6%) (21), or in eastern Africa (17%) (22). In terms of lifetime intimate partner sexual violence, the prevalence rate we documented among HIV-infected women in Togo (69.7%) is twice the rate of that among uninfected women and considerably higher than among other reported estimations in Africa, such as in Uganda (12%) (22). We reported here as well that more than half (51.6%) of the HIV-infected women had been victims of both types of violence, while this proportion was 18.6% among uninfected women.

Bruises were overall the most frequent injuries reported among African women victims of physical violence (23), but the proportion of Togolese women reporting serious and disabling injuries such as dislocations (62.9%) and deep cuts (28.9%) is alarming. The severity of the consequences of intimate partner violence is often underestimated. Indeed, in our study, a substantial proportion of women reported needing medical care after being severely injured, but only a few actually accessed medical care and, if they did, they rarely disclosed the real causes of their injuries. Finally, none of the injured women in our sample had been referred to existing organizations providing psychological support. These findings suggest that case detection of intimate partner violence should be systematically done by medical staff, and particularly within HIV care services. Clinical care evaluation checklists could include items related to physical and sexual violence to actively detect intimate partner violence. In addition, to ensure adequate case management, medical staff should be sensitized about intimate partner violence and consequences management and should be able to refer women to the appropriate supporting structures.

Although the prevalence of physical and sexual violence varied according to the HIV status among women of our sample, we identified three associated factors that are common to both groups. Reported partner multiple concurrent relationships were associated with higher rates of physical and sexual intimate partner violence regardless of serological status. Other African studies have reported similar findings, whereby women whose partner had several female partners were more likely to report sexual intimate partner violence and women suspecting their partner’s infidelity were at higher risk of any kind of violent act perpetrated by their male partner (12, 24–26). In Tanzania and South Africa, men acknowledging having multiple female partners confessed that being questioned about their fidelity could trigger physical and sexual violent acts against their female partner (27, 28). Gender norms in many African cultures expect masculine men to be in control of women, and this control can take the form of sexual multi-partnership and violent acts. On the other hand, the prevailing ideal of femininity in such contexts may prevent women from refusing these sociocultural patterns, and on the contrary seems to promote the acceptance of this behavior, increasing their risk of contracting HIV infection through sexual assaults (29). Multi-partnership, mostly among men, is an increasing HIV risk behavior in West African countries and the need to intensify behavior change efforts have been already pointed out (11). We believe that such efforts should focus on tackling masculinity construction, fostering women’s respect, and reducing gender inequality, all of which should be part of a comprehensive HIV behavior change preventive package.

We observed that starting sexual life before 18 years old was very frequent and appeared to be a risk factor of intimate partner violence for all women, regardless of their serological status. The high proportion of HIV-infected women experiencing a first forced sexual intercourse in our study is consistent with findings in South Africa (30). Since sexual abuse most often means unprotected sexual intercourse, these women may have been at the same time exposed to the risk of contracting HIV infection since very early ages and led into the vicious cycle of intimate partner violence (31). Age-appropriate sexuality education contributes to more responsible sexual behavior; nevertheless, gaps in basic knowledge about HIV and its transmission among young men and women remain important challenges (11). Sexuality education should be considered as a gateway to prevent HIV infection through a change of traditional gender norms based on fostering responsible and respectful sexual behaviors as early as possible in life.

Finally, prevailing submissive attitudes among Togolese women, expressed by an overall high acceptance of partner controlling behaviors and high submission index score, were associated with intimate partner violence, principally among HIV-infected women. Many women fearing intimate partner violence, even when aware of an HIV risk, may feel powerless to discuss infidelity, condom use, and HIV testing with their male partner (31). Renewed efforts are needed to foster women empowerment, including negotiation skills for safe-sexual practices addressed to women victims of intimate partner violence.

Several limits to our study need to be acknowledged. First, intimate partner violence may be a very sensitive subject for women and data collection was based on past experiences. We thus should not rule out a potential recall bias. To reduce information bias, however, interviewers were trained before conducting the survey and interviews were carried out in a private office. Further, due to the cross-sectional design of our study, we were not able to demonstrate a causal link between HIV infection and intimate partner violence or to explore the dynamic among these factors; however, our findings may have confirmed some of the factors to target when aiming at preventing intimate partner violence within clinical care services. Moreover, we did not present data on intimate partner psychological violence, as this would have required a thorough psychological assessment that could not be performed at the time of the survey. Finally, our study was conducted among a specific population of women attending a hospital facility in Lomé, and having been tested for HIV at least once in their life; thus, our results are not representative of all women in Togo.

One of the main strengths of our study, however, is its contribution to the pool of data available on intimate partner violence, data that can be compared to other settings as it was based on the use of *WHO Multi-Country Study on Women’s Health and Life Events* questionnaire. Our study highlights that intimate partner violence is a true public health issue in Togo, with a high social burden and severe health consequences on women, and especially among HIV-infected women. Our findings argue, in particular, for systematic case detecting of intimate partner violence – as well as any form of violence – within HIV services, to provide adequate medical care to women in need and to advise them about help-seeking strategies. Taking into account the high rate of HIV-discordant stable couples in sub-Saharan Africa, couple-oriented interventions are a priority among primary HIV preventive strategies (13), and because intimate partner violence, highly prevalent in West African contexts and one important consequence of HIV serological disclosure among HIV-infected women, is a major barrier to the acceptability of such approaches (12, 13), the assessment of intimate partner violence should be included in couple-oriented HIV preventive strategies.

Finally, tackling cultural representations and the social construction of masculinity and traditional gender norms in sub-Saharan African contexts is an important challenge to achieve behavior change in terms of sexual health and must be addressed from adolescent ages. Further longitudinal research is needed to understand HIV-serodiscordant couple dynamics with regard to intimate partner violence in African contexts and thus improve the acceptability and efficacy of couple-oriented HIV preventive interventions. Intimate partner violence, being highly prevalent in resource-constrained settings and a major public health concern, global health policies must turn more firmly to this issue.

## Statements:

The study protocol was approved by the National Ethic Committee of the Ministry of Health of Togo. All participants provided written informed consent.

This study was funded through the core grant of the INSERM U897 Research Centre in Bordeaux. Additional financial and logistical support was provided by the French National Agency on AIDS and viral hepatitis Research (ANRS, grant #12200). Juan Burgos-Soto was a PhD fellow of the French Ministry of Research. The sponsors had no role in study design and the collection, analysis, interpretation of data, the writing of the article, and the decision to submit it for publication.

The authors are indebted to the women who accepted to participate in this study. We wish to thank the staff of the Sylvanus Olympio Hospital for their assistance and particularly Rabiatou Darago who supervised the local team of psychologists to conduct the study.

This study was previously presented in part: Juan Burgos-Soto et al. Intimate partner sexual and physical violence according to HIV-status among women in Togo: prevalence and associated factors. 19th Conference on Retroviruses and Opportunistic Infections, Seattle, United States, March 2012 [Abstract # U-108].

## References

[1] WHO (2002). World report on violence and health.

[2] United Nations (1993). United Nations declaration on the elimination of violence against women.

[3] Garcia-Moreno C, Watts C (2011). Violence against women: an urgent public health priority. Bull World Health Organ.

[4] Garcia-Moreno C, Jansen HA, Ellsberg M, Heise L, Watts CH (2006). Prevalence of intimate partner violence: findings from the WHO multi-country study on women’s health and domestic violence. Lancet.

[5] Campbell J, Jones AS, Dienemann J, Kub J, Schollenberger J, O’Campo P (2002). Intimate partner violence and physical health consequences. Arch Intern Med.

[6] Ellsberg M, Jansen HA, Heise L, Watts CH, Garcia-Moreno C (2008). Intimate partner violence and women’s physical and mental health in the WHO multi-country study on women’s health and domestic violence: an observational study. Lancet.

[7] Deyessa N, Berhane Y, Ellsberg M, Emmelin M, Kullgren G, Hogberg U (2010). Violence against women in relation to literacy and area of residence in Ethiopia. Glob Health Action.

[8] Silverman JG, Decker MR, Saggurti N, Balaiah D, Raj A (2008). Intimate partner violence and HIV infection among married Indian women. JAMA.

[9] Decker MR, Seage GR, Hemenway D, Raj A, Saggurti N, Balaiah D (2009). Intimate partner violence functions as both a risk marker and risk factor for women’s HIV infection: findings from Indian husband-wife dyads. J Acquir Immune Defic Syndr.

[10] WHO (2010). Addresing violence against women and HIV/AIDS: what works?.

[11] UNAIDS (2012). UNAIDS Report on the global AIDS epidemic.

[12] Curran K, Baeten JM, Coates TJ, Kurth A, Mugo NR, Celum C (2012). HIV-1 prevention for HIV-1 serodiscordant couples. Curr HIV/AIDS Rep.

[13] WHO (2012). Male involvement in the prevention of mother-to-child transmission of HIV.

[14] Orne-Gliemann J, Balestre E, Tchendjou P, Miric M, Darak S, Butsashvili M (2013). Increasing HIV testing among male partners. The Prenahtest ANRS 12127 multi-country randomised trial. AIDS.

[15] Desgrees-du-Lou A, Orne-Gliemann J (2008). Couple-centred testing and counselling for HIV serodiscordant heterosexual couples in sub-Saharan Africa. Reprod Health Matters.

[16] Orne-Gliemann J, Desgrees-Du-Lou A (2008). The involvement of men within prenatal HIV counselling and testing. Facts, constraints and hopes. AIDS.

[17] Aluisio A, Richardson BA, Bosire R, John-Stewart G, Mbori-Ngacha D, Farquhar C (2011). Male antenatal attendance and HIV testing are associated with decreased infant HIV infection and increased HIV-free survival. J Acquir Immune Defic Syndr.

[18] WHO (2005). WHO multi-country study on women health and life events questionnaire (Version 10).

[19] WHO (2005). WHO multi-country study on women’s health and domestic violence against women. Intial results on prevalence, health outcomes and women’s responses. Full report.

[20] Hosmer DW, Lemeshow S (2000). Applied Logistic Regression.

[21] Ezeanochie MC, Olagbuji BN, Ande AB, Kubeyinje WE, Okonofua FE (2011). Prevalence and correlates of intimate partner violence against HIV-seropositive pregnant women in a Nigerian population. Acta Obstet Gynecol Scand.

[22] Osinde MO, Kaye DK, Kakaire O (2011). Intimate partner violence among women with HIV infection in rural Uganda: critical implications for policy and practice. BMC Womens Health.

[23] Antai D (2011). Traumatic physical health consequences of intimate partner violence against women: what is the role of community-level factors?. BMC Womens Health.

[24] Townsend L, Jewkes R, Mathews C, Johnston LG, Flisher AJ, Zembe Y (2011). HIV risk behaviours and their relationship to intimate partner violence (IPV) among men who have multiple female sexual partners in Cape Town, South Africa. AIDS Behav.

[25] Abramsky T, Watts CH, Garcia-Moreno C, Devries K, Kiss L, Ellsberg M (2011). What factors are associated with recent intimate partner violence? findings from the WHO multi-country study on women’s health and domestic violence. BMC Public Health.

[26] Were E, Curran K, Delany-Moretlwe S, Nakku-Joloba E, Mugo NR, Kiarie J (2011). A prospective study of frequency and correlates of intimate partner violence among African heterosexual HIV serodiscordant couples. AIDS.

[27] Lary H, Maman S, Katebalila M, McCauley A, Mbwambo J (2004). Exploring the association between HIV and violence: young people’s experiences with infidelity, violence and forced sex in Dar es Salaam, Tanzania. Int Fam Plan Perspect.

[28] Abrahams N, Jewkes R, Laubscher R, Hoffman M (2006). Intimate partner violence: prevalence and risk factors for men in Cape Town, South Africa. Violence Vict.

[29] Jewkes R, Morrell R (2010). Gender and sexuality: emerging perspectives from the heterosexual epidemic in South Africa and implications for HIV risk and prevention. J Int AIDS Soc.

[30] Mathews C, Aaro LE, Flisher AJ, Mukoma W, Wubs AG, Schaalma H (2009). Predictors of early first sexual intercourse among adolescents in Cape Town, South Africa. Health Educ Res.

[31] Christofides N, Jewkes R (2010). Acceptability of universal screening for intimate partner violence in voluntary HIV testing and counseling services in South Africa and service implications. AIDS Care.

# Sub article: Thai men’s experiences of alcohol addiction and treatment

**DOI:** 10.3402/gha.v7.23712

**Published:** 2014-05-19

**Authors:** Kulnaree Hanpatchaiyakul, Henrik Eriksson, Jureerat Kijsompon, Gunnel Östlund

**Affiliations:** 1 School of Health Care and Social Welfare, Mälardalen University, Eskilstuna, Sweden; 2 Basic Concept of Nursing Practice Department, Boromarajonani College of Nursing Changwat Nonthaburi, Nonthaburi, Thailand; 3 Swedish Red Cross University College, Stockholm, Sweden; 4 Praboromarajchanok Institute for Health Workforce Development (PIHWD), Ministry of Public Health, Nonthaburi, Thailand

**Keywords:** alcohol addiction, homo-social, hegemonic masculinity, alcohol treatment, barriers

## Abstract

**Background:**

Men are overrepresented with regard to alcohol addiction and in terms of alcohol treatment worldwide. In Thailand, alcohol consumption continues to rise, but few of those afflicted with alcohol addiction attend alcohol treatment programs, even though there is universal care for all. No comprehensive studies have been done on men’s experiences with addiction and alcohol treatment programs in Thailand.

**Objective:**

The aim of this study was to explore men’s experiences in terms of the ‘pros and cons of alcohol consumption’ in order to identify the barriers that exist for Thai men with regard to alcohol addiction and the decision to stop drinking.

**Design:**

Purposive sampling was applied in the process of recruiting participants at an alcohol clinic in a hospital in Thailand. Thirteen men with alcohol addiction (aged 32–49 years) were willing to participate and were interviewed in thematic interviews. The analysis of the data was done with descriptive phenomenology.

**Results:**

Through men’s descriptions, three clusters of experiences were found that were ‘mending the body’, ‘drinking as payoff and doping related to work’, and ‘alcohol becoming a best friend’ as ways of describing the development of addiction.

**Conclusions:**

The results highlight the importance of addressing concepts of masculinity and related hegemonic ideas in order to decrease the influence of the barriers that exist for Thai men with alcohol addiction with regard to entering treatment and to stop drinking.

Globally, excessive alcohol consumption has had a major impact on the lives of individuals, their families and communities, and on their physical and mental health. It is a causal factor in more than 60 major types of diseases and injuries (1). Men have a five-time higher prevalence for alcohol consumption and related problems than women; however, this trend is changing, with an increase in drinking by more young women in recent years in Thailand (2–4). The most common problems were identical for both men and women drinkers, namely the effect of drinking on work and finances, a lack of study or employment opportunities leading to increased drinking, and feeling guilt or remorse about drinking. For adolescents, males and females experienced different problems: fighting was the most frequent problem among males while drinking, and females suffered feelings of guilt or experienced a sense of remorse about drinking alcohol (2, 4).

Currently, in Thai society the consumption of alcohol is a widely accepted social activity. Thai people use alcohol as a mode of recreation and to enhance interactions among people in the community, specifically on holidays and at parties (4). In general, there is a forgiving attitude about social drinking with regard to men, though a more socially strict attitude exists toward women who drink. Research has found that women mostly drink at home and at parties, while men often drink at the workplace and in bars (4, 5). Moulinsart and Jongudomkarn et al. (6, 7) explained that the extent of a man’s daily consumption was an ingrained part of northeastern culture in Thailand and was related to various functions. For instance, these people believe that alcohol can be used for medicinal purposes such as a sleep inducer, appetite enhancement beverage, muscle relaxer, or for improved blood circulation. It is also commonly consumed as a refreshment to quench thirst and to accompany meals, particularly in the evening.

Although Buddhism, the main religion in Thailand, requires that good persons abstain from intoxicating their minds, drinking is overt and easily accessible (8, 9). Thamarangsi (8) pointed out that Chinese drinking culture has influenced Thai drinking patterns ever since the Ayuthaya period (1350–1767). At that time, Chinese migrants established the alcohol market and introduced the distillation technique for manufacturing spirits. Since then the alcohol market has been growing and has a huge impact on Thai society (8), such that drinking alcohol is now part of the cultural traditions in both China and Thailand. Obviously, Chinese traditional medicine has made use of alcohol as a major solvent in herbal medicine preparations (10).

Thailand has provided universal health care since 2002. As a result, people have had access to health care services through registered government facilities or non-government facilities with very little cost to themselves (11). Although the universal coverage scheme includes alcohol and substance treatment, only a small number of alcohol dependent patients, most of whom are men, actually receive specialty treatment for alcohol abuse (12). It seems as though the majority of the treatment for alcohol addiction is focused on physical disease rather than addiction, which requires long-term and continuing care (1).

## Men’s alcohol consumption

Health beliefs and behaviors among Thai men are heavily influenced by culture, as are the consumption of alcohol and patterns of seeking treatment for alcohol dependence. Men use general practitioner care service less frequently than women, and when they do seek health care services they are more likely than woman to focus on physical problems and less likely to discuss mental or emotional problems (13). ‘Authentic’ masculinity is constructed in part through the sharing of drinking stories within homo-social groups, in which the body’s ability to tolerate alcohol is seen as unlimited (14, 15). Drinking ‘too little’, or not at all, is also connected to subordinate constructions of masculinity, suggesting ‘weakness’ (16–19).

Gender differences in alcohol consumption are universal. More frequent and heavy drinking occurs among men, while more long-term abstention occurs among women; no cultural differences or historical changes have entirely erased these differences (20). According to De Visser and Smith (21), alcohol consumption is seen to be a specifically masculine behavior, and men may use alcohol consumption to demonstrate a specific pattern of masculine competence. Homo-social interaction can therefore explain how alcohol consumption connects to the constructions of the male identity. Emslie et al. (17) have shown that middle-aged men believe that the shared consumption of alcohol is an integral part of creating and maintaining male friendships. Drinking with friends was constructed, and indeed justified, as a way of helping men talk to each other, and as a way of providing social support and improving moods.

## Barriers to accessing treatment for alcohol dependence

There seems to be negative public stigma and attitudes toward alcohol addiction, labeling those who are addicted as dangerous, unpredictable and untrustworthy (22). Certain impacts of this public stigma include the fear of being labeled as an addicted person, embarrassment, and the worry of being looked down upon by others (23). The individuals who are at risk of excessive drinking believe that the general public reacts negatively to any individual who seeks help for alcohol treatment within the primary and specialty care sectors (24). On the contrary, Zakrzewski and Hector (25) demonstrated that men’s justification for alcohol consumption involved the desire to find a nice feeling, at least temporarily, as drinking alcohol was thought to be fun and to help them to cope with their lack of self-esteem. Research has found that stigmatizing behavior by health care providers has a potential to increase patient frustration and to increase the risk of patients leaving treatment impulsively, acting inappropriately, or refusing to return to particular alcohol treatment settings (26). Studies of patients with alcohol dependence indicate that the stigmatizing behavior they experience when entering the detoxification unit negatively influences the person’s self-esteem and self-efficacy (27).

Barriers to alcohol treatment, as seen from the patients’ perspective, included concerns of privacy and the belief that treatment was either unnecessary or not beneficial, as well as other practical and economic impediments to participation (28). The most frequent claim by participants was that they ‘wanted to handle the problem on my own’ (25, 29–31). In addition to the issue of privacy concerns, time difficulties and a fear of treatment were also reported by several studies (30–32).

It is difficult to raise public awareness regarding alcohol-related health-risk issues, as alcohol consumption among men in Thailand is connected to public acceptance and cultural involvement. In order to provide suitable and effective treatment for alcohol addiction, it is necessary to have extensive knowledge of the pros and cons of drinking, the accessibility of alcohol treatment and the social context in which this alcohol use is grounded. The authors of this study have experience in treating alcohol addiction in both Thailand and Sweden, with relevant backgrounds in health research from the fields of medical anthropology, nursing and social work. Few studies of alcohol addiction have been conducted in Thailand, and it is important to uncover why many resist accessing available treatments. This study aimed to investigate the barriers to alcohol treatment, with regard to the ‘pros and cons of drinking’, in order to identify the relevant barriers that exist before, during and after alcohol treatment. A bottom-up approach was used in order to explore this topic through Thai men’s lived experiences.

## Method

The present study was conducted at a hospital in Thailand that treated alcohol addiction. Data were collected from December 2012 to January 2013. After receiving a formal letter from the researcher responsible for collecting the data, the director of the hospital granted permission for the study to be conducted. The head nurse assisted the researcher with selecting the specific patients who could describe their life experiences and provide rich data. The participants obtained both oral and written information on the aim and procedure of the study from the principal researcher.

### The sample

Purposive sampling was used with the following inclusion criteria: taking part in alcohol treatment, diagnosed as having an alcohol abuse and dependence disorder, and over 20 years of age. Those diagnosed with psychotic disorders were excluded from the study. During the 2 months of data collection, 18 potential participants were asked to participate in the study, 14 of which agreed. Of these 14 individuals interviewed, one was a woman who we later decided to exclude. Thus, 13 men with alcohol dependence took part in the study, aged 32–49 years. Seven men were single, four were divorced, and the remaining two were married (Table 1). All participants gave informed consent.

**Table 1:** Number of male interviewees undergoing alcohol treatment in Thailand (2012–2013)

No.	Age	Number being treated at hospital	Program of treatment	Status
1	40	2	Detoxification	Married
2	34	Over 30	Detoxification	Single
3	34	3	Rehabilitation	Single
4	41	3	Detoxification	Divorce
5	36	3	Detoxification	Single
6	38	3	Detoxification	Single
7	39	1	Detoxification	Single
8	49	3	Rehabilitation	Divorce
9	34	3	Rehabilitation	Divorce
10	34	1	Detoxification	Single
11	43	3	Detoxification	Married
12	36	8	Detoxification	Divorce
13	32	6	Detoxification	Single

### The interviews

The interviews were conducted in a quiet room at the alcohol clinic. The researcher made contact with the participants by using ‘small talk’ and by asking permission from the patients to record the conversation. The questions asked were ‘Could you tell me about your experiences with regard to drinking alcohol and alcohol treatment?’ The interviewer (KH) kept an open-mind, listened carefully and encouraged them to reflect on their experiences more concretely by asking questions like ‘How do you feel?’ or ‘Could you please give me an example?’ Five participants were interviewed twice in order to get sufficient and rich data from each person; the other eight men were interviewed only once. Ten men had been admitted to the Detoxification Unit and three men had been admitted at the Rehabilitation Unit at the time the interviews took place. Each interview lasted at least 1 hour and 30 min. The 18 interviews were transcribed verbatim, resulting in a transcript of 203 pages.

### Data analysis

Phenomenological descriptive methodology was used to analyze the data. The method aims to find subjective experiences through established methods of bracketing, intuiting, analyzing, and describing. Such a philosophical framework is used to create meaning from actual lived experiences as revealed by the participants (33–35).

The interview transcripts were read several times to gain a sense of all of the material before starting the analysis. The first author listened to the tapes while reading the transcription and noted the various aspects captured during the interviews (34–36). During the reading of the transcripts, the overall meaning was identified through the differences and similarities recognized among the utterances. The text was then divided into small parts, so called ‘meaning units’. The meaning units were then classified into different subcategories. In this part of the analysis, the first author analyzed all the interviews as a whole, but there was continual movement from the whole to the parts and movement back to the whole. The data were kept at a concrete level, as far as possible, in order to minimize the effects of pre-understanding on the analysis. All meaning units were then translated into English and discussed with the co-authors. We reflected on all the meaning units by asking the specific question ‘What is this man really telling us?’ During these repeated discussions, subcategories and main categories began to emerge. As the analysis moved from a concrete to an abstract level of understanding, the essence was categorized in relation to each interview and in relation to the meaning units and categories (34–37).

### Ethical consideration

The study was approved in Thailand by Thanyarak Hospital Ethical Committee and in Sweden by the Uppsala Ethical Vetting Board, number 2012/493.

## Results

Thirteen Thai men described the pros and cons of drinking during hospitalization. They suggested that drinking is normative for men and that drinking was necessary in working culture in order to engage with colleagues and during occasions of celebration. They also described specific benefits from alcohol consumption, including relieving pain, reducing stress, and achieving balance their bodies that lead to increase work output. The cons of drinking described including being labeled by others, a loss of control, and being forced into treatment. Moreover, they also claimed that they received little benefit from the treatment.

By entering treatment in order to solve physical problems indicates that the patients were more interested in getting relief from their withdrawal symptoms than anything else. The treatment process had little influence on them, as they still believed that they could handle their drinking and that it was possible to return to drinking after treatment. This was also based on another important aspect of the men’s lived experiences of alcohol addiction, which included trying to keep alcohol as a part of their lives. Relapses and excessive consumption occurred when the men used alcohol during social activities or at work.

Three categories emerged in the men’s descriptions of the obstacles experienced before, during and after alcohol treatment: alcohol treatment as a ‘tool for mending the body’, alcohol as a method for ‘payoff and doping’, and alcohol as a best friend. These categories are described in detail below.

### Alcohol treatment as a ‘tool for mending the body’

Most of the men reported negative associations from being forced to attend various treatments by family members due to crisis situations. Although they recognized that heavy drinking was harmful for them, they often wanted to get through treatment in order to obtain control over drinking, not in order to stop drinking. Earlier experiences of treatment in the temple clinic provided by Thai traditional methods included the use of herbs, meditation, prayer, and spiritual Buddhist practices. One man claimed that he was concerned about having falsely promising the monks that he would stay sober; ‘If you wrongly swore it meant that you have sinned; I returned to drinking and never turn back to the temple anymore’ (Rew). Some men shared stories of friends that had returned to drinking after making such false promises and had died as a result of their drinking; these men never again swore to stop drinking, as they were worried about committing sin. Others compared their way of drinking with their relatives or friends who drank even more, but still appeared to be healthy.

Although treatment was effective in coping with withdrawal symptoms and improving body relaxation, it seemed to have little influence on long-term drinking habits. Most participants believed that they could control their drinking; for instance, if they restricted drinking to after work, they could satisfy their family’s expectations:

I believed it (alcohol) is a part of my life. I tried hard to control it, to the extent that it bears the least effect upon me. Although, I am here (the alcohol ward) I know I can’t stop drinking, but need to relax my physical state. So, I should drink in the evening, to compromise. (Arun)

Many of the men had previously attended alcohol treatment programs in the health care departments of hospitals due to a loss of bodily control or experiencing physical disorders. One man compared the treatment unit to a kind of quarantine center rather than a treatment facility; he did not believe it to be curative. The negative descriptions of treatment at the hospital included argumentative statements, such as the program is not useful, there is a lack of privacy, the treatment stay is boring, the professionals lack competence, the method in the alcohol treatment program is wrong or not clarified enough, and that the process is difficult to understand.

The program used was only for a short term period, and it was above my ability to understand. For the program to be effective, one need to have an able mind and more time, it was certainly a waste of time for them to get my attention and interest. They will never understand that. (Chon)

During treatment, the patients were most concerned with mending their bodies and relieving withdrawal symptoms and they wanted medicine offered for these purposes. The patients’ main intentions were to achieve bodily control and to be cared for according to their own wishes.

### Alcohol as a method for ‘payoff and doping’

While working, men noted that drinking alcohol was permitted during working hours, since that helped them work a bit more, at least in the short term. The patients worked as agricultural laborers, employees at the factory, on-site laborers, gardeners, and landscapers. According to these men, consuming alcohol helped them to relieve their muscle pain, stop shaking and reduce stress such that they managed to work even more. Some of the men stated that when they worked, they continually drank approximately 50–100 ml, four times per day, as can be seen in the following example:

I have worked in the rubber plantation and drank homemade white whisky to get a good feeling and muscle relaxation. After purchasing alcohol in the evening, it was divided into four little bottles and drunk four times during the day, in the evening, before bedtime at 11.00 p.m., all before work and during work hours. (Chuchai)

These men thought that the consumption of alcohol was a reasonable act in connection to their work. Several men with low-socioeconomic work as farm laborers consumed alcohol during the day and in the evenings in order to create a pleasant atmosphere, for refreshment and complete their work. Men with higher socioeconomic work, such as employers and officers, explained that they drank to maintain their social network and to satisfy their own cravings. Some men reported having muscle pains or shaking and they drank alcohol to balance their body and mind, and in order to be able to continue working; it could therefore be said that alcohol was used as a form of self-medication. During work, it was an act of generosity from a boss or a customer to provide alcohol as a payoff for work done, as well as a method for doping to ensure that each man worked to his full capacity. Several men explained that they receive alcohol and food from customers when working in the cadastral survey, paddy-farming, planting, or in gardening. One man stated that being a boss entails being friendly to the laborers, providing alcohol to make them get more work done. In addition, the boss wanted to create a good relationship with the workers by drinking with them, as the following example shows:

For those people, look at me, drunk all the time. The fact is that I just drank with my workers, purchased 200 baht worth of alcohol, for the work force to raise more money. So, I have to pay for them. (Tee)

As can be seen, drinking was regarded a method for ‘payoff and doping’ utilized by men of higher socio-economic for engaging with their employees. The tradition of giving alcohol is particularly used to stimulate laborers to increase their level of work output, as has been described earlier in this text (e.g. doping them to work longer hours). Another reason for providing alcohol to the work force is to be labeled as a generous and kind boss.

### Alcohol as a best friend

During the interviews, the participants expressed shame and embarrassment at not being able to handle their drinking, and perceived it as a sign of worthlessness. They also stated that this lack of control over their drinking resulted in negative judgments by others, which left them feeling isolated. Most of the participants described feeling lonely, lacking trust or caring feeling toward others, and stated that drinking released them from certain negative feelings. They also stated that although drinking sometimes felt more like a job or a nuisance and was no longer fun, they needed alcohol to survive and relieve pain. Two men described their depression and suicidal thoughts, even attempted suicide during bouts of heavy drinking. Another participant expressed his hopelessness in stopping to drink and another felt guilty regarding his consumption behavior. They also described experiences of stigmatization, like feeling dishonored which further lowered their self-esteem and increased their drinking, as stated by one of the participants:

No close friends were trusted to tell my private matters to. I looked at the trees, the fish, talked with the invisible things and just imagined they could understand. I drank and began thinking about the mistakes that I have done with people over the last year. What were my mistakes and who were the people that gave back to me. Why have I done these things? Feeling disappointed by my unexpected unemployment, I began to lead a dog and cat life, drinking heavily when socializing … whenever I ran into a good friend. (Son)

According to this quote, the man labeled himself a dog or a cat, meaning that he felt worthless, as he was dependent on his parents, had no close friends, experienced a loss of family, felt dishonored, and was unemployed. Most men preferred to stay home and drink alone in order to avoid getting into trouble when drinking outside the home, though they also often interacted with others while drinking. Despite family support for all of the men interviewed in treatment, most had suffered multiple failures in life, including failed marriages, work and previous treatment attempts. These feelings of embarrassment and shame lowered their self-esteem. Drinking alone was a way to ease life when feeling different or marginalized. One man explained how he hid alcohol from others in order to protect his self-esteem:

I bought it and hid it inside my shirt, fastened by the belt. I don’t want to be seen drinking. I drink but can do my work, like other people do. I was often cited as a good example of a sober drinker, unlike those who drink otherwise. (Chuchai)

Drinking when unemployed is looked down upon by society, according to Thai men. For this reason, the participants prioritized maintaining their jobs while simultaneously continuing to drink.

## Discussion

The ‘pros and cons of drinking’, according to the experiences of 13 Thai men, were explored in order to identify the relevant barriers that exist before, during and after treatment for alcohol abuse. These pros and cons were segregated into three categories: alcohol as ‘payoff and doping method’ (mostly pros), alcohol treatment as a ‘tool for mending the body’ (both pros and cons) and ‘alcohol as a best friend’ (both pros and cons).

The general reasons for drinking alcohol included peer pressure, relaxation after work, and reducing stress (5–7, 32). The participants also emphasized the value of drinking in facilitating socializing and enhancing interpersonal relationships. Other functions of drinking in social and cultural occasions include celebration, hospitality, and reciprocity. Thus, drinking alcohol can serve more than one function on any given occasion (6, 7).

The advantages of drinking can be understood by the theory of homo-social grouping, where men normalize alcohol consumption as part of the construction of male identity. Thailand has been described as a society based on the concepts of a patronage-driven culture, which values preconceived notions of high masculinity that are somehow associated with alcohol consumption. Thai men who are active in society and employed outside of the home ‘have to drink’ in order to meet the skewed cultural values of obsessive masculinity as they relate to alcohol consumption (6). Furthermore, drinking is utilized as a mediation device in developing relationships among Thai men. Offering alcohol at work or after work is a social construction that, according to the present study, is considered a sign of generosity and respect; for example, participants argued that being a boss entails providing alcohol and drinks to his crew, as a way to respect and honor them as men (14, 15). Thus, the findings illustrate that men’s drinking is part of the notion of hegemonic masculinity, where men drink socially with men.

The participants described the disadvantages of drinking, including being forced into treatment and issues of self-stigmatization. Interestingly, a positive outcome of treatment was the relief of withdrawal symptoms; withdrawal symptoms have been found to be the major concern in the acute hospitalization of people with alcohol addiction (38). According to Jakobsson et al. (19), many men are forced to seek treatment and they expressed shame and embarrassment as a result of their inability to handle alcohol, perceiving this failure as a sign of weakness. However, having a comorbid affective disorder or other problems directly attributable to alcohol use increases the likelihood that such individuals will seek treatment (13).

Although the participants had made several failed attempts to stay sober, they still believed that they could control their drinking. Unlike another research study, previous treatment attempts did not seem to improve the patients’ knowledge (23). The interviewed men were rather ashamed of their earlier treatment attempts, particularly having made false promises of sobriety to monks, since Buddhist precepts dictate avoiding intoxication, the drinking of alcohol and the taking of drugs (8, 9). Embarrassment and shame further damaged the men’s already low self-esteem and they described suffering from the social stigma associated with alcohol addiction, as have been found in previous research (22, 23, 27). Shame and a lack of self-esteem could combine to become one major barrier to the treatment of alcohol addiction. Research has found that men use health care services and seek help less frequently than women and are more likely to refrain from disclosing mental or emotional problems (13).

The health care services need to develop a gender relational perspective of masculinities and health (13, 15). In relation to men suffering from alcohol addiction, this would include critical investigation of the division of labor and domestic work as well as identifying the influence of socioeconomic differences among groups. The present findings provide vital insight into how men construct the drinking culture through their stories and how its link to male bonding in homo-social grouping behavior seems to be a crucial barrier to alcohol treatment and to abstaining from drinking among certain groups of Thai men. Risk-taking plays a role in constructing masculine identities, and the sharing of drinking stories that include risk-taking is integral in creating and maintaining male friendships (13, 17, 18). The themes of men’s stories reflect the pressures of male culture in terms of being the breadwinner and continuing to work, as was pinpointed by Connell and Messerschmidt (14). Hegemonic visions of masculinity seem to be of vital importance in alcohol addiction and ‘knowledge of doing gender’ needs to be included when health care services consider appropriate treatment.

### Clinical implications

The research was conducted at a hospital in Thailand, which includes a detoxification and rehabilitation unit for alcohol treatment. One limitation of the study was the small sample; however, the participants were heterogeneous and representative of Thai men who typically enter alcohol treatment programs. The interviews also provided rich and in-depth descriptions of men’s experiences of alcohol addiction and treatment programs.

In listening carefully to the men’s descriptions of addiction and of their struggles in life, new knowledge of Thai men’s alcohol and working culture was found. One apparent barrier during treatment was that men spent the majority of their time focusing on physical problems and not the social or psychological aspects of their addiction. This raises questions with regard to the level of the patients’ engagement in the treatment. However, the hegemonic masculinity construction and the act of drinking alcohol as a part of homo-social grouping at work among groups of Thai men can be understood as a barrier before, during and after treatment that needs to be further studied. Moreover, the act of entering alcohol treatment programs among Thai men is related to self-stigma.

The barriers found on an individual level included focusing on the body instead of the addiction behavior, drinking alcohol during work, and self-stigmatization and shame in relation to the treatment or the treatment attempts. These barriers highlight the importance of addressing masculinity and hegemonic ideas in order to decrease the influence of the barriers before, during and after treatment for Thai men with alcohol addiction.

## References

[1] World Health Organization (2011). Global status report on alcohol and health. http://www.who.int/substance_abuse/publications/global_alcohol_report/msbgsruprofiles.Pdf.

[2] Office of Narcotics Control Board (2007). The National household survey for substance and alcohol use. The Administrative committee for substance Abuse Research Network (ACSAN) of the Office of Narcotics Control Board (ONCB), Ministry of Justice.

[3] National Statistical Office (2011). Statistical year book, Thailand. National Statistical Office, Ministry of information and communication Technology.

[4] Assanangkornchai S, Sam-Angsri N, Rerngpongpan S, Lertnakorn A (2010). Patterns of alcohol consumption in the Thai population: results of the National Household Survey of 2007. Alcohol Alcohol.

[5] Rungreangkulkij S, Kotnara I, Chirawatkul S (2012). It is easy to start drinking, but too difficult to stop. J Nurs Sci Hum.

[6] Jongudomkarn D, Pultong S, Pongsiri K, Chawalisan P, Chanchum K, Singhawara P (2012). Effects of the ‘Kumpavapi model’, community-based interventions on undesirable drinking conditions in the communities. http://cas.or.th/index.php/research.

[7] Moolasart J (2011). The social construction of alcohol consumption in the North-east of Thailand.

[8] Thamarangsi T (2006). Thailand: alcohol today. Addiction.

[9] Thamarangsi T, Puangsuwan A (2010). Why Thailand should have the pictorial warning label on alcoholic beverage package. http://ihppthaigov.net/DB/publication/attachresearch/257/chapter1.pdf.

[10] Cochrane J, Chen H, Conigren KM (2003). Alcohol use in China. Alcohol Alcohol.

[11] Suraratdecha C, Saithanu S, Tangcharoensathien V (2005). Is universal coverage a solution for disparities in health care? Findings from three low-income provinces of Thailand. Health Policy.

[12] Tanyarak Hospital (2013). The inpatient and outpatient statistic. http://thanyarak.go.th/thai/.

[13] Schofield T, Connell RW, Walker L, Wood JF, Butland DL (2000). Understanding men’s health and illness: a gender-relations approach to policy, research, and practice. J Am Coll Health.

[14] Connell RW, Messerschmidt JW (2005). Hegemonic masculinity: rethinking the concept. Gend Soc.

[15] Courtenay WH (2000). Constructions of masculinity and their influence on men’s well-being: a theory of gender and health. Soc Sci Med.

[16] Bird SR (1996). Welcome to the men’s club: homo-sociality and the maintenance of hegemonic masculinity. Gend Soc.

[17] Emslie C, Hunt K, Lyons A (2013). The role of alcohol in forging and maintaining friendships amongst Scottish men in midlife. Health Psychol.

[18] Peralta RL (2007). College alcohol use and the embodiment of hegemonic masculinity among European American men. Sex Roles.

[19] Jakobsson A, Hensing G, Spak F (2008). The role of gendered conceptions in treatment seeking for alcohol problems. Scand J Caring Sci.

[20] Wilsnack RW, Wilsnack SC, Kristjanson AF, Vogeltanz-Holm ND, Gmel G (2009). Gender and alcohol consumption: patterns from the multinational GENACIS project. Addiction.

[21] De Visser RO, Smith JA (2007). Alcohol consumption and masculine identity among young men. Psychol Health.

[22] Ahern J, Stuber J, Galea S (2007). Stigma, discrimination and the health of illicit drug users. Drug Alcohol Depend.

[23] Saunders SM, Zygowicz KM, D’Angelo BR (2006). Person-related and treatment-related barriers to alcohol treatment. J Subst Abuse Treat.

[24] Fortney J, Mukherjee S, Curran G, Fortney S, Han X, Booth BM (2004). Factors associated with perceived stigma for alcohol use and treatment among at-risk drinkers. J Behav Health Serv Res.

[25] Zakrzewski RF, Hector MA (2004). The lived experiences of alcohol addiction: men of alcoholics anonymous. Issues Ment Health Nurs.

[26] Sleeper JA, Bochain SS (2013). Stigmatization by nurses as perceived by substance abuse patients: a phenomenological study. J Nurs Educ.

[27] Schomerus G, Corrigan PW, Klauer T, Kuwert P, Freyberger HJ, Lucht M (2011). Self-stigma in alcohol dependence: consequences for drinking-refusal self-efficacy. Drug Alcohol Depend.

[28] Tucker J, Vuchinich R, Rippens P (2004). A factor analytic study of influences on patterns of help-seeking among treated and untreated alcohol dependent persons. J Subst Abuse Treat.

[29] Cunningham JA, Sobell LC, Sobell MB, Agrawal S, Toneatto T (1993). Barriers to treatment: why alcohol and drug abusers delay or never seek treatment. Addict Behav.

[30] Rapp RC, Xu J, Carr CA, Lane DT, Wang J, Carlson R (2006). Treatment barriers identified by substance abusers assessed at a centralized intake unit. J Subst Abuse Treat.

[31] Siebert DC (2005). Help seeking for AOD misuse among social worker. Soc Work.

[32] Barman R, Mahi R, Kumar N, Sharma KC, Sidhu BS, Singh D (2011). Barriers to treatment of substance abuse in a Rural Population of India. Addiction.

[33] Gray MT (2004). Philosophical inquiry in nursing: an argument for radical empiricism as a philosophical framework for the phenomenology of addiction. Qual Health Res.

[34] Dahlberg K, Drew N, Nyström M (2008). Reflective life world research.

[35] Wojnar DM, Swanson KM (2007). Phenomenology: an exploration. J Holist Nurs.

[36] Giorgi A (1997). The status of Husserlian phenomenology in caring research … Paley J. Husserl, phenomenology and nursing. J Adv Nurs.

[37] Dahlberg K (2006). The essence of essences – the search for meaning structures in phenomenological analysis of life world phenomena. Int J Qual Stud Health Well-being.

[38] Pollini RA, O’Toole TP, Ford D, Bigelow G (2006). Does this patient really want treatment? Factors associated with baseline and evolving readiness for change among hospitalized substance using adults interested in treatment. Addict Behav.

# Sub article: Women and NCDs: Overcoming the neglect

**DOI:** 10.3402/gha.v7.23742

**Published:** 2014-05-05

**Authors:** Ruth Bonita, Robert Beaglehole

**Affiliations:** University of Auckland, Auckland, New Zealand

**Keywords:** women, gender bias, global health targets, non-communicable diseases, risk factors

## Abstract

Two in every three deaths among women are caused by non-communicable diseases (NCDs) – largely heart disease, stroke, cancer, diabetes and chronic respiratory diseases. The global discourse on health, however, largely views women in terms of their reproductive capacity, a persisting myth reflecting gender bias that shifts the focus away from NCDs, violence, and other injuries. Risk factors for NCDs are similar for men and women. Because fewer women actively smoke than men, and drink in less harmful ways, in most parts of the world, the impact of major NCD risk factors is far less in women than in men. In the area of diagnosis and treatment, gender bias can result in women being asked fewer questions, and receiving fewer examinations and fewer diagnostic tests for coronary heart disease and other NCDs compared with men with similar symptoms. In response to a UN meeting in September 2011, member states of WHO have agreed to a global goal to reduce avoidable NCD mortality by 25% by 2025 (‘25 by 25’). A set of voluntary targets and indictors have been agreed upon, although none of them are gender specific. Most require changes at the policy level that will ensure that women – and children – will also benefit. As the 2015 deadline for the Millennium Development Goals approaches, women and NCDs should be central to the sustainable human development agenda.

Four major transitions are occurring in global health: age, sex, and cause-specific death rates are generally declining; life expectancy is increasing; populations are aging; and there is a dramatic shift from deaths at younger ages, principally due to infectious diseases, to non-communicable diseases (NCDs) – principally cardiovascular diseases (CVDs), cancer, chronic respiratory diseases, and diabetes. NCDs have been the leading causes of death among women globally for at least the past three decades and are now responsible for two in every three deaths among women each year (1).

Several enduring myths have contributed to the neglect of NCDs in women (2). First, there is a strong and persistent view that only health-related issues of importance to women are defined through their reproductive capacity (3), yet two thirds of all deaths and disabilities in women relate to chronic diseases, violence, and other injuries. This myth in particular, reflects, in part, a gender bias (4). Second, NCDs, especially CVDs, have been considered primarily as diseases of men. Although age-specific NCD death rates in women lag behind the rates in men by about 10 years, the absolute number of NCD deaths in women (16.2 million) is similar to that of men (18.4 million) because women live longer – on average, between 6 and 8 years (5). The third myth is that NCDs in women are an issue only in high-income countries. In fact, most NCD deaths in women occur in low- and middle-income countries and the rates in these countries are much higher than in wealthy countries. The fourth and the most distressing myth is that NCDs cause deaths only in older people and ‘we have to die of something, so why bother with complicated conditions like NCDs’. Unfortunately, and especially now in low- and middle-income countries, NCD deaths are frequent in people under the age of 70 years, including women, and many of these deaths are slow and miserable. A large proportion of NCD deaths, especially before old age, are avoidable with cheap, cost-effective, and in some cases, cost-saving interventions.

The good news is that, despite the overall increasing number of NCD deaths in women due to increasing population size and aging, the age-specific death rates, especially for CVD, are declining, and in some countries, surprisingly quickly (6–8). In many countries, the decline began before governments or non-governmental organizations mounted awareness and prevention campaigns. It is likely that the initial decline in death rates was due to the diffusion of information from the early epidemiological studies, notably the Framingham Heart Study, and the powerful evidence on the relationship between tobacco use, lung cancer, and CVD that began to be disseminated in the 1960s.

More recently, treatments – especially drugs to manage high levels of blood pressure and cholesterol – have also played an important part in continuing the decline in death rates which now include lung cancer in men, but not yet women, as well as stomach, breast, and cervical cancers. The challenge now is to apply globally the interventions that have been so beneficial to women in high-income countries.

Risk factors for heart disease and stroke, the two leading causes of death among women, are similar for men and women. Factors such as age and family history play a role, but the majority of CVD deaths are due to modifiable risk factors such as tobacco use; diets high in fat, salt, and sugar; high blood pressure; high cholesterol; obesity; and diabetes. Because fewer women actively smoke – or have smoked for a shorter time – than men, and because they drink less and in less harmful ways than do men, in most parts of the world the major NCD risk factors cause less of a burden in women than in men (9) and therefore more ambitious risk factor targets will be required for women than for men to achieve the ‘25 by 25’ premature NCD mortality target set by WHO (10). Dietary risk factors have broadly similar effects for both women and men. With the exception of high body mass index and high fasting plasma glucose, most of the leading risk factors for NCDs have declined, especially in high-income countries (3). Obesity levels are a concern for women; almost everywhere, women are more obese than men. There are also significant differences in the way both symptomatic and asymptomatic women are treated compared to men. For example, gender, but not age, race, or social class of a patient significantly influenced doctors’ diagnostic and management activities in a study that controlled for these variables simultaneously. Women were asked fewer questions, received fewer examinations, and had fewer diagnostic tests ordered for coronary heart disease (11, 12). These differences are a reflection of the strong gender bias against equitable prevention and treatment of women (13).

The future of women and the treatment of NCDs is encouraging. There is an increasing recognition of the importance of a life course approach to the prevention of NCDs beginning with the health of girls and young women before and during pregnancy (14). The integration of NCD prevention activities into maternal and women-centric health programs has considerable potential since there is generally poor access to care for women, girls, and other vulnerable groups affected by NCDs (15). In many societies, women lack control over resources and, hence, cannot afford quality care for NCDs. Women also face sociocultural, geographic, and economic barriers to access to care. They are less recognized and catered to in terms of accessibility, comprehensiveness, and responsiveness of healthcare systems.

Furthermore, there have been major global initiatives to overcome the general neglect of NCDs. The UN High-Level Meeting on the Prevention and Control of NCDs in September 2011 was a turning point. Heads of state and governments made many major commitments to reverse the neglect of NCDs, and the health of women figures prominently in the political declaration from this meeting (16). In response to the UN meeting, member states of WHO have agreed upon a global goal to reduce avoidable NCD mortality by 25% by 2025 (‘25 by 25’), and a set of voluntary targets and indictors have also been agreed upon, although none of them are gender specific (17).

The targets agreed upon by WHO member states cover nine areas. The Lancet NCD Action Group has proposed a smaller set of priority targets – three to five – in the belief that it is important for countries, especially those beginning their prevention and management efforts, to concentrate on interventions which are among the ‘best buys’ in terms of health impact and will thus ensure rapid progress toward the ‘25 by 25’ goal (18). Of course, once experience and capacity builds, a broader range of interventions should be implemented. The Lancet NCD Action Group proposes a stepwise approach to NCD prevention beginning with sustained high-level leadership; multisectoral action; a focus on tobacco control and salt reduction; and the identification in primary health care of women and men at high overall risk of CVD (>30% over 10 years) and their treatment with cheap generic combination drugs (19).

Tobacco control is an excellent entry point for NCD prevention, and accelerated implementation of the Framework Convention on Tobacco Control is top priority. Tobacco use in high-income countries is at comparable levels among women and men, and in most of these countries, the prevalence of smoking is declining slowly as an increasing range of evidence-based cost-saving control measures are introduced. The most effective intervention is regular and substantial increases in the price of tobacco; plain packaging of tobacco products, first introduced in Australia in 2012, has opened a new phase in the fight against the tobacco industry.

Fortunately, in many low- and middle-income countries, for example, China and Indonesia, smoking rates are substantially lower in women than in men. This market represents a major opportunity for the tobacco industry, and it is doing its best to recruit new smokers, including enticing young women. Women use tobacco in a variety of forms, not just smoked tobacco. For this reason, the proposed WHO voluntary target is to reduce tobacco use by 30% by 2025. This target is not sufficiently ambitious and, following the lead of New Zealand which is committed to being essentially tobacco-free by 2025 (that is, a smoking prevalence of <5%), the Lancet NCD Action Group is proposing a tobacco-free world by 2040 (20). Secondhand smoke is a particular issue for women in low- and middle-income countries that do not yet have effective smoke-free environments (21). Effective legislation to protect people, especially women and children, from secondhand smoke is a priority.

Salt reduction will have an important effect on reducing population blood pressure levels through its impact on CVDs (22). Although the dominant approach in wealthy countries to lowering blood pressure is through medical treatment, this approach is not practical or affordable in most countries (23). Because about half the CVD events occur in people with ‘normal’ blood pressures, that is, below a systolic blood pressure of 140 mmHg, population salt reduction has the major advantage of leading to fewer CVD deaths in these people. Women will play a vital role in cultures where salt is overused; they can more easily take a leadership role at the household level in terms of reducing discretionary salt use. The agreed upon voluntary target is a 30% reduction in per capita salt intake by 2025; the original WHO target was a per capita intake of 5 g per day (24).

The priority treatment goal is to increase the coverage in primary health care of generic drugs for men and women at high risk of CVD. The dominant approach to the medical management of high risk is to treat individual elevated risk factors based on arbitrary cut points for ‘abnormality’. A much more efficient approach is to manage people at elevated overall risk based on their age, gender, and combined risk factor status; this approach will lead to a lower number of women on treatment – because of their lower overall risk – and better use of limited resources for the same or greater health impact. However, for this approach to reach its potential, it will need to be embraced by health professionals trained in the traditional medical model.

Assuring progress toward the NCD goal ‘25 by 25’, requires the establishment of an independent accountability mechanism, monitoring trends, reviewing progress, and acting as indicated to accelerate progress (25). A useful precedent has been set by the establishment of an independent expert advisory group to oversee progress toward the goals for Women's and Children's health; this model has relevance to NCDs. It is desirable for NCDs to be incorporated into the accountability mechanisms for other global health priorities.

As the 2015 deadline for the Millennium Development Goals approaches, discussions are underway on the post-2015 development agenda. It is critical that women and NCDs are central to the new human development agenda. So far, the prospects are promising with NCDs being recognized in the health consultations as being central to human development. If NCDs assume their rightful place post-2015, women throughout the world will benefit enormously.

## References

[1] Lozano R, Naghavi M, Foreman K, Lim S, Shibuya K, Aboyans V (2012). Global and regional mortality from 235 causes of death for 20 age groups in 1990 and 2010: a systematic analysis for the Global Burden of Disease Study 2010. Lancet.

[2] WHO (2005). Preventing chronic diseases: a vital investment.

[3] Bustreo F, Knaul FM, Bhadelia A, Beard J, de Carvalho IA (2012). Women's health beyond reproduction: meeting the challenges (editorial). Bull World Health Organ.

[4] Ruiz-Cantero MT, Vives-Cases C, Artazcoz L, Delgardo A, Garcia Calvente M, Miqueo C (2007). A framework to analyses gender bias in epidemiological research. J Epidemiol Community Health.

[5] Salomon JA, Wang H, Freeman MK, Vos T, Flaxman AD, Lopez AD (2012). Healthy life expectancy for 187 countries, 1990–2010: a systematic analysis of the Global Burden of Disease Study 2010. Lancet.

[6] Berg J, Bjork L, Lappas G, O'Flaherty M, Capewell S, Rosengren A (2014). Continuing decrease in coronary heart disease mortality in Sweden. BMC Cardiovasc Disord.

[7] Capewell S, O'Flaherty M (2011). Rapid mortality falls after risk factor changes in populations. Lancet.

[8] Hotchkiss JW, Davies CA, Dundas R, Hawkins N, Jhund PS, Scholes S (2014). Explaining trends in Scottish coronary heart disease mortality between 2000–2010 using IMPACTSEC model: retrospective analyses using routine data. BMJ.

[9] Lim SS, Vos T, Flaxman AD, Danaei G, Shibuya K, Adair-Rohani H (2013). A comparative risk assessment of burden of disease and injury attributable to 67 risk factors and risk factor clusters in 21 regions, 1990–2010: a systematic analysis for the Global Burden of Disease Study 2010. Lancet.

[10] Kontis V, Mathers CD, Rehm J, Stevens GA, Shield KD, Bonita R (2014). Contribution of six risk factors to achieving the 25×25 non-communicable disease mortality reduction target: a modelling study. Lancet.

[11] Chang AM, Mumma B, Keara L, Sease K, Robey JL, Shofer FS (2007). Gender Bias in Cardiovascular Testing Persists after Adjustment for Presenting Characteristics and Cardiac Risk. Academic Emergency Medicine.

[12] Arber S, McKinlay J, Adams A, Marceau L, Link C, O'Donnel A (2006). Patient characteristics and inequalities in doctors’ diagnostic and management strategies relating to coronary heart disease: a video-simulation experiment. Soc Sci Med.

[13] Risberg G, Johansson EE, Hamberg K (2009). A theoretical model for analyzing gender bias in medicine. Int J Equity Health.

[14] Power C, Kuh D, Morton S (2013). From developmental origins of adult diseases to life course research on adult disease and ageing: insights from birth cohort studies. Annu Rev Public Health.

[15] Maina WK (2011). Integrated non-communicable disease prevention into maternal and child health programs: can it be done and what will it take?. Int J Gynaecol Obstet.

[16] United Nations Political declaration of the high-level meeting of the general assembly on the prevention and control of non-communicable diseases. http://www.who.int/nmh/events/un_ncd/political_declaration_/en.pdf.

[17] World Health Organization NCD global monitoring framework: ensuring progress on non-communicable diseases in countries. http://www.who.int/nmh/global_monitoring_framework/en.

[18] Bonita R, Magnusson R, Bovet P, Zhao D, Malta DC, Geneau R (2013). Country actions to meet the UN commitments on non-communicable diseases: a stepwise approach. Lancet.

[19] Lim SS, Gaziano TA, Gakidou E, Reddy KS, Farzadfar F, Lozano R (2007). Prevention of cardiovascular disease in high-risk individuals in low-income and middle-income countries: health effects and costs. Lancet.

[20] Beaglehole R, Bonita R, Horton R, Ezzati M, Bhala N, Amuyunzu-Nyamongo M (2012). Measuring progress on NCDs: one goal and five targets. Lancet.

[21] Oberg M, Jaakkola MS, Woodward A, Peruga A, Pruss-Ustun A (2011). Worldwide burden of disease from exposure to second-hand smoke: a retrospective analysis of 193 countries. Lancet.

[22] He FJ, Brinsden HC, MacGregor GA (2013). Salt reduction lowers cardiovascular risk: meta-analysis of outcome trials. Lancet.

[23] Tang JL, Hu YH (2005). Drugs for preventing cardiovascular disease in China. Br Med J.

[24] World Health Organization (2012). Sodium for adults and children: guidelines. http://www.who.int/nutrition/publications/guidelines/sodium-intake/en.

[25] Beaglehole R, Bonita R, Horton R (2013). Independent accountability for NCDs. Lancet.

# Sub article: Inconsistent condom use among Ugandan university students from a gender perspective: a cross-sectional study

**DOI:** 10.3402/gha.v7.22942

**Published:** 2014-04-10

**Authors:** Devika Mehra, Per-Olof Östergren, Björn Ekman, Anette Agardh

**Affiliations:** Division of Social Medicine and Global Health, Department of Clinical Sciences, Lund University, Malmö, Sweden

**Keywords:** condom efficacy, gender, peer norms, Uganda, condom use, HIV

## Abstract

**Background:**

Feminization of the HIV/AIDS epidemic has been a prominent phenomenon in sub-Saharan Africa. Inconsistent condom use among young people is one of the major risk factors in the continued propagation of the epidemic. Therefore, it is of importance to increase knowledge of gender aspects of condom use among young people.

**Objective:**

To investigate whether gender differences regarding individual and social factors determine the association between condom efficacy and inconsistent condom use with a new sex partner, among Ugandan university students.

**Design:**

In 2010, 1954 Ugandan students participated in a cross-sectional survey, conducted at Mbarara University of Science and Technology in southwestern Uganda. A self-administered questionnaire assessed socio-demographic factors, alcohol consumption, sexual behaviors (including condom use and condom efficacy), and peer norms. The data were stratified by sex and examined by multivariate logistic regression analysis.

**Results:**

A total of 1,179 (60.3%) students reported having had their sexual debut. Of these, 231 (37.4%) males and 209 (49.2%) females reported inconsistent condom use with a new sex partner. Students with low condom efficacy had a higher risk of inconsistent condom use with a new sex partner, even after adjusting for the potential confounders. A synergistic effect was observed between being a female and low condom efficacy with inconsistent condom use.

**Conclusion:**

The association between inconsistent condom use and low condom efficacy was found among both males and females, but females were found to be at a higher risk of inconsistent condom use compared to their male counterparts. Therefore, gender power relations should be addressed in policies and interventions aiming at increasing condom use among young people in sub-Saharan settings. Programs could be designed with intervention strategies that focus on interactive and participatory educational activities and youth-friendly counseling of young people, which in turn may improve their interpersonal communication and condom negotiation skills with their partners.

The feminization of the HIV/AIDS epidemic has been a prominent phenomenon in sub-Saharan Africa (1). Individuals between the ages of 15 and 24 have been severely affected, and 75% of all new HIV cases in this age group occur in women (2). In sub-Saharan Africa, the epidemic has increased due to socio-cultural factors, behind which are gender inequalities (3). Uganda has one of the world's youngest populations, with a high national prevalence of HIV (7.3%) (4). The prevalence in the age group cited above differs from males (2.1%) to females (4.9%). Furthermore, a greater increase was seen in females (7.1%) than in males (2.8%) between the ages of 20 and 24 years (4).

Risky sexual behaviors among young people in Uganda have been measured in a recent survey that showed 24% had more than one sexual partner during the past 12 months, 79% had a non-regular partner, and 10% had been affected by STI (sexually transmitted infections)-related symptoms (5). This population is therefore at high risk for STIs and unintended pregnancies. Results from studies targeting university students in Uganda showed a higher risk of inconsistent condom use among female students, in comparison to their male counterparts (5, 6).

Condom use among young people is determined by individual and social factors. Perceived self-efficacy is one of the individual factors that can influence condom use. It is a concept derived from social cognitive theory and is considered as a factor that could potentially lead to health-related behavioral change (7). Perceived self-efficacy is defined as confidence in one's ability to exhibit motivation and capability to achieve a given goal (8). Condom efficacy is a person's confidence in his or her ability to successfully use a condom during sexual intercourse (9). Such efficacy requires risk reduction and self-regulation skills, but possessing the skills and being able to transform them into action under difficult circumstances are two different matters (10).

In the Ugandan context, where gendered cultural norms and inequitable power relations prevail, women have less control in a sexual relationship (11). Interpersonal communication along with behavioral skills between partners is an integral part of a relationship that determines behavior (12, 13). A positive attitude towards condoms and a greater confidence in one's ability to use them consistently in various circumstances corresponds to higher levels of condom use (14, 15).

There are various factors that influence condom use. In prior studies ‘intention to use a condom’ has been shown to be an important predictor (16, 17). The theory of planned behavior (18) and its extended versions (19) have suggested that in the absence of environmental barriers, any behavior is more likely to occur if there is a strong intention and ability to carry it out. Behavioral intentions in turn are determined by attitudes, subjective norms, and self-efficacy which account for considerable variation in actual condom use in different situations (18).

Individual factors affecting behavior change should not be seen in isolation. Numerous social factors influence the behaviors of young people. Peer norms can exert considerable pressure on young people and affect their decisions (20). According to studies conducted among US university students, discussions about romantic relationships, alcohol consumption, and sexual behaviors can shape perceptions of what their peers consider normative behavior (21, 22). Normative perceptions of sexual experiences can be an important influence in student's decision to engage in risky sexual activities such as having multiple sexual partners, using condoms inconsistently, and alcohol consumption in conjunction to sex (22). Studies conducted among Ethiopian, Cambodian, and Laotian adolescents have supported the notion that peer influences can affect risky sexual practices (23, 24). Another study in South Africa of young people, showed that higher self-efficacy to communicate with peers, increased the likelihood of condom use (25). The same study showed that more females communicated with their peers than did their male counterparts.

Consistent condom use is determined by a number of factors, some of which are linked to gender in more or less obvious ways. Some of these factors prevent inconsistent condom use, while others may work in the opposite direction. Knowledge of whether these factors contribute to feminization of the HIV/AIDS epidemic in sub-Saharan Africa appears to be incomplete. Although some African studies have documented the association between self-efficacy and condom use (25, 26), gender differences are not very well examined. Therefore, the aim of this study is to investigate whether gender differences regarding individual and social factors (peer norms) determine inconsistent condom use with a new sexual partner, particularly with regard to condom efficacy among university students in Uganda.

## Methods

### Study design and setting

The study was conducted at the Mbarara University of Science and Technology (MUST), a public institution that is the second largest university in Uganda. It is located in the center of Mbarara, approximately 350 km to the southwest of the capital city, Kampala. In 2010, the number of universities in Uganda expanded resulting in 29 new institutions of higher learning. A greater number of students are now being enrolled in universities than in the past. Those students receiving government scholarships live on campus during their entire course of study, while others remain on campus during their first and second years and then move on to privately run hostels.

We analyzed a cross-sectional data set of undergraduate students from the university's four faculties: science, medicine, computer science, and development studies. The sample consisted of 1,954 participating students out of a total enrolment of 2,706, representing 72% of all undergraduates. As the outcome of the study was risky sexual behavior, the analysis was based on a subset of 1,179 students who stated that they had debuted sexually. Of the respondents, 58.8% were male (*n*=693) and 41.2% female (*n*=486). The Institutional Review Committee at MUST granted ethics approval for the project.

### Data collection and analysis

The data were collected by means of an 11-page self-administered questionnaire with 132 questions based on socio-demographic factors, academic progress, social capital, mental health, sexual behavior (condom efficacy and condom use), alcohol consumption, and other lifestyle variables. The questionnaire was also used in previous studies of university students in this setting (6, 27, 28).

The entire undergraduate student body at MUST was invited to take part in the survey. Prior to the questionnaire distribution, a consent form was circulated describing the purpose of the study, and students were asked to sign if they agreed to participate. The research team informed the students that participation in the survey was voluntary and anonymity would be assured. The contact details of the project's principal investigator and the research assistant were provided in case students had any personal questions. The signed consent forms, and completed questionnaires were deposited by each student in a sealed box.

### Definition of variables

#### Background variables

*Socio-demographic variables (individual level)*. Age was categorized as ≤23 (‘younger’) and >23 (‘older’). The cut-off was based on the median age of our study sample.

Area of growing up was dichotomized into rural or urban. The latter option combined peri-urban and small town.

Educational level of head of the household during childhood was categorized as ‘did not complete primary school’ or ‘completed primary school’, which were coded as ‘≤primary school’ and ‘>primary school’.

The role of religion in the family while growing up was dichotomized into major role (‘religion played a big role or was relatively important’) or minor role (‘religion was not so important or not important at all’).

#### Sexual behavior variables

Pleasure of using a condom was based on the question ‘How do you compare the degree of pleasure using a condom during intercourse with not using one?’. The responses were ‘no difference’ and ‘more pleasure with a condom’ and coded as: ‘same or more pleasure’ and ‘less pleasure’ remained coded as ‘less pleasure’.

Intention to use a condom was based on the statement ‘I intend to use a condom whenever I have intercourse with a new sex partner’ Those who responded were coded as ‘yes’ and others were classified as ‘no’.

Multiple sexual partners was determined by the response to the question ‘How many sexual partners have you had during the last 12 months?’ and was dichotomized into ‘0 to 1’ and ‘≥2’. The respondents in the latter category were classified as having multiple sexual partners.

#### Exposure variable

Condom efficacy was constructed by combining two statements: ‘I am satisfied with my ability to use a condom correctly’ and ‘I believe I can persuade a new sex partner to use a condom’. The respondents who indicated ‘yes’ regarding both statements were coded as ‘high efficacy’ and the other responses were categorized as ‘low efficacy’.

*Social level (peer norms)*. Peers using a condom with a new sex partner was based on the statement ‘My friends at the university always use a condom with a new partner’. The response alternatives were ‘yes’ or ‘no’.

Peers having difficulty demanding condom use was based on the statement ‘My friends at the university have difficulty demanding condom use with a new partner’. The response alternatives were ‘yes’ or ‘no’.

Alcohol consumption on the occasion of sexual intercourse had the following response alternatives: ‘always or almost always’, ‘more often than on half of the occasions’, ‘about half of the occasions’, ‘more seldom than a quarter of the occasions’ and ‘almost never or never’. The first three options were coded as ‘frequent user’ and the last two as ‘infrequent user’.

#### Dependent variable

Inconsistent condom use was ascertained by asking the question ‘How often do you use a condom with a new sexual partner?’ The response option ‘always’ was coded as ‘consistent condom use’ and the other alternatives (‘often’, ‘sometimes’, ‘never’, ‘does not apply to me’) were coded as ‘inconsistent condom use’.

### Statistical analysis

Statistical analysis was conducted using PASW (SPSS) statistical package Version 21.0. We first measured the prevalence of the variables we used within our sample population. Logistic regression analysis then calculated the crude odds ratio (OR) with 95% confidence interval (CI) for investigating the association between socio-demographic factors, condom efficacy, intention to use a condom, pleasure of using a condom, alcohol consumption in relation to sex, and peer norms in conjunction with inconsistent condom use with a new sexual partner. Multivariate logistic regression was used step-wise to control for the potential confounders of age, area of origin, pleasure of using a condom, intention to use a condom, multiple sexual partners, alcohol in relation to sexual intercourse and peer norms. Estimates of effect modification (synergy) were done as ‘departure from additivity of effects on the chosen outcome scale’ and calculation of synergy index (SI) was carried out to disclose effect modification between the chosen variables as proposed by Rothman and Greenland (29).

The following algorithm was used, whereby SI>1 signifies a synergistic effect (representing a positive effect modification) and SI<1 an antagonistic effect (representing a negative effect modification):

$$\text{SI}=\frac{\left(\text{O}{\text{R}}_{\left(1+1\right)}-1\right)}{\left(\text{O}{\text{R}}_{\left(1+0\right)}-1)+(\text{O}{\text{R}}_{\left(0+1\right)}-1\right)}$$

where: OR_(1+1)_=odds ratio for dummy variable exposed to both factors

OR_(1+0)_=odds ratio for dummy variable exposed to one factor

OR_(0+1)_=odds ratio for dummy variable exposed to other factor

OR_(0+0)_=odds ratio for the dummy variable unexposed to both factors

Significant effect moderation between two variables indicates that when both are present there is an amplified effect, i.e. the combination of the two indicators has a stronger effect, which is higher than added effect of the variables in question.

## Results

Table 1 gives the prevalence of all the socio-demographic factors, condom efficacy, intention to use a condom, pleasure of using a condom, peer norms, and other sexual behavior variables of the total sample. The stratification of the sample was done on the basis of sex.

**Table 1:** Prevalence of socio-demographic factors, alcohol consumption, sexual behaviors (including condom efficacy), and condom use among Ugandan university students

		All *n*=1,179 (%)	Male *n*=693 (%)	Female *n*=486 (%)	*X* ^2^ p*
Individual level					
Age	≤23	743 (65.0)	407 (60.5)	336 (71.5)	0.001
	>23	400 (35.0)	266 (39.5)	134 (28.5)	
	Missing	(36)	(20)	(16)	
Area of growing up	Urban	607 (51.7)	336 (48.8)	271 (56.0)	0.015
	Rural	566 (48.3)	353 (51.2)	213 (44.0)	
	Missing	(6)	(4)	(2)	
Educational level of	>Primary	820 (71.0)	465 (68.6)	355 (74.4)	0.018
head of household	≤Primary	335 (29.0)	213 (31.4)	122 (25.6)	
	Missing	(24)	(15)	(9)	
Religion	Major role	731 (62.5)	400 (58.2)	331 (68.5)	0.001
	Minor role	439 (37.5)	287 (41.8)	152 (31.5)	
	Missing	(9)	(6)	(3)	
Condom efficacy	High	764 (75.4)	485 (77.8)	279 (71.5)	0.025
	Low	249 (24.6)	138 (22.2)	111 (28.5)	
	Missing	(166)	(70)	(96)	
Intention to use a condom	Yes	393 (33.3)	267 (38.5)	126 (25.9)	0.001
with any new sex partner	No	786 (66.7)	426 (61.5)	360 (74.1)	
Pleasure using a condom	No difference/More pleasure	319 (33.2)	175 (30.0)	144 (38.2)	0.009
	Less pleasure	641 (66.8)	408 (70.0)	233 (61.8)	
	Missing	(219)	(110)	(109)	
Condom use with a new partner	Consistent	603 (57.8)	387 (62.6)	216 (50.8)	0.001
	Inconsistent	440 (42.2)	231 (37.4)	209 (49.2)	
	Missing	(136)	(75)	(61)	
Sexual partners in the last 12 months	0–1	680 (66.4)	356 (58.7)	324 (77.5)	0.001
	≥2	344 (33.6)	250 (41.3)	94 (22.5)	
	Missing	(155)	(87)	(68)	
In a relationship	Yes	923 (80.2)	519 (77.0)	404 (84.7)	0.001
	No	228 (19.8)	155 (23.0)	73 (15.3)	
	Missing	(28)	(19)	(56)	
Alcohol on the occasion	Infrequent users	391 (79.0)	245 (76.6)	146 (83.4)	0.084
of sexual intercourse^a^	Frequent users	104 (21.0)	75 (23.4)	29 (16.6)	
	Missing	(95)	(68)	(27)	
Social Level (peer norms)					
Friends always use condom	Yes	660 (67.4)	400 (66.8)	260 (68.4)	0.625
with new partner	No	319 (32.6)	199 (33.2)	120 (31.6)	
	Missing	(200)	(94)	(106)	
Friends have difficulty demanding	Yes	501 (50.7)	311 (51.4)	190 (49.5)	0.558
condom use with new partner	No	488 (49.3)	294 (48.6)	194 (50.5)	
	Missing	(190)	(88)	(102)	

* *p*-value in table is analyzed based on sex.

a Analyzed only for those who consumed alcohol.

A higher percentage of females in our sample (71.5%) were younger than 23 years compared to their male counterparts (60.5%). A greater proportion of females (28.5%) reported low condom efficacy than males (22.2%). Intention to use a condom with a new sex partner was higher in females (74.1%) than in males (61.5%). A larger majority of male respondents (70%) reported less pleasure with a condom as compared to females (61.8%). Inconsistent condom use with a new sexual partner was higher among females (49.2%) than males (37.4%). Approximately two thirds of the students (67.4) reported that their friends use a condom with a new sex partner; there was not much difference between males and females.

Table 2 provides an analysis of the associations between socio-demographic factors and condom efficacy in relation to inconsistent condom use with a new sex partner. Growing up in a rural environment was significantly associated with inconsistent condom use. This association was significant for males (OR crude 1.81, 95% CI 1.30–2.52) and females (OR crude 1.67, 95% CI 1.13–2.47). Our main exposure, low condom efficacy, had a significant association with inconsistent condom use, for males (OR crude 4.66, 95% CI 3.08–7.07) and females (OR crude 6.45, 95% CI 3.84–10.81). Intention to use a condom with a new sex partner did not show an association (OR crude 0.98, 95% CI 0.76–1.27), with no significant results found in males and females. Less pleasure using a condom was significantly associated (OR crude 1.43, 95% CI 1.07–1.90), with no gender difference in males and females. Multiple sexual partners in the last 12 months had a significant negative association with inconsistent condom use among males (OR crude 0.69, 95% CI 0.48–0.97), and the point estimate was similar for females (OR crude 0.70, 95% CI 0.43–1.11). Frequent consumption of alcohol on the occasion of sexual intercourse showed a significant association (OR crude 1.64, 95% CI 1.05–2.57), but there was no obvious gender difference. The response that friends do not always use a condom with a new partner was associated with the outcome among males (OR crude 1.48, 95% CI 1.03–2.13) and females (OR crude 1.81, 95% CI 1.14–2.87). Reporting that friends would have difficulty demanding that a condom be used with a new sex partner was associated with inconsistent condom use (OR crude 1.40, 95% CI 1.06–1.82) but our study found no gender differences in this regard.

**Table 2:** Association between socio-demographic factors, alcohol consumption, sexual behavior, condom efficacy, and inconsistent condom use among Ugandan university students

		All *n* (%)	OR (95% CI)	Male *n* (%)	OR (95% CI)	Female *n* (%)	OR (95% CI)
Individual level							
Sex	Male	231 (37.4)	1 (Ref)				
	Female	209 (49.2)	**1.62 (1.26–2.08)**				
Age	≤23	138 (38.9)	1 (Ref)	79 (33.1)	1 (Ref)	59 (50.9)	1 (Ref)
	>23	285 (43.5)	1.21 (0.93–1.57)	145 (40.1)	1.35 (0.96–1.90)	140 (47.6)	0.88 (0.57–1.35)
Area of growing up	Urban	200 (36.4)	1 (Ref)	94 (30.5)	1 (Ref)	106 (43.8)	1 (Ref)
	Rural	239 (48.9)	**1.67 (1.30–2.14)**	136 (44.3)	**1.81 (1.30–2.52)**	103 (56.6)	**1.67 (1.13–2.47)**
Educational level of head of	>Primary	298 (41.0)	1 (Ref)	148 (36.2)	1 (Ref)	150 (47.3)	1 (Ref)
household	≤Primary	136 (45.9)	1.22 (0.93–1.60)	80 (40.8)	1.22 (0.86–1.72)	56 (56.0)	1.42 (0.90–2.23)
Religion	Major role	272 (42.4)	1 (Ref)	126 (35.8)	1 (Ref)	146 (50.3)	1 (Ref)
	Minor role	164 (41.7)	0.97 (0.75–1.26)	102 (39.2)	0.86 (0.57–1.30)	62 (46.6)	1.16 (0.83–1.61)
Condom efficacy	High efficacy	206 (28.9)	1 (Ref)	125 (27.7)	1 (Ref)	81 (30.9)	1 (Ref)
	Low efficacy	157 (68.6)	**5.38 (3.90–7.42)**	82 (64.1)	**4.66 (3.08–7.07)**	75 (74.3)	**6.45 (3.84–10.81)**
Intention to use condom	No	283 (42.4)	1 (Ref)	129 (35.6)	1 (Ref)	154 (50.3)	1 (Ref)
with new partner	Yes	157 (41.9)	0.98 (0.76–1.27)	102 (39.8)	1.20 (0.86–1.66)	55 (46.2)	0.85 (0.55–1.30)
Pleasure using a condom	No difference/More pleasure	103 (34.3)	1 (Ref)	51 (30.7)	1 (Ref)	52 (38.8)	1 (Ref)
	Less pleasure	255 (42.8)	**1.43 (1.07–1.90)**	148 (39.3)	1.46 (0.99–2.15)	107 (48.9)	1.51 (0.97–2.33)
Sexual partners in	0–1	281 (45.4)	1 (Ref)	132 (41.0)	1 (Ref)	149 (50.2)	1 (Ref)
the last 12 months	≥2	115 (34.6)	**0.64 (0.48–0.84)**	78 (32.2)	**0.69 (0.48–0.97)**	37 (41.1)	0.70 (0.43–1.11)
In a relationship	Yes	358 (42.5)	1 (Ref)	179 (37.5)	1 (Ref)	179 (49.0)	1 (Ref)
	No	71 (39.7)	0.89 (0.64–1.23)	45 (36.3)	0.95 (0.63–1.43)	26 (47.3)	0.93 (0.53–1.64)
Alcohol on the occasion of	Infrequent user	143 (38.3)	1 (Ref)	82 (35.0)	1 (Ref)	61 (43.9)	1 (Ref)
sexual intercourse^a^	Frequent user	49 (50.5)	**1.64 (1.05–2.57)**	34 (47.2)	1.66 (0.97–2.83)	15 (60.0)	1.99 (0.80–4.57)
Social Level (peer norms)							
Friends always use a condom	Yes	217 (36.6)	1 (Ref)	121 (33.8)	1 (Ref)	96 (40.9)	1 (Ref)
with a new partner	No	141 (47.6)	**1.58 (1.19**–**2.09)**	81 (43.1)	**1.48 (1.03–2.13)**	60 (55.6)	**1.81 (1.14–2.87**)
Friends have difficulty	No	159 (36.5)	1 (Ref)	88 (33.3)	1 (Ref)	71 (41.3)	1 (Ref)
demanding condom use with new partner	Yes	207 (44.4)	**1.40 (1.06–1.82)**	118 (40.7)	1.37 (0.97–1.94)	89 (50.6)	1.46 (0.95–2.22)

a Analyzed only for those who consumed alcohol.

Table 3 presents the adjusted OR with 95% CI for association between condom efficacy and inconsistent condom use (adjusted for the confounding factors of sex, age, rural origin, friends who always use a condom with a new partner, friends who have a difficulty demanding a condom, less pleasure using a condom, multiple sex partners, and alcohol consumption in conjunction to sexual intercourse). In the fully-adjusted model, the statistically significant association persisted between low condom efficacy and inconsistent condom use (OR adjusted 3.94, 95% CI 2.20–7.05).

**Table 3:** Association (adjusted odds ratio, 95% CI) between condom efficacy and inconsistent condom use among Ugandan University students

	Model 1	Model 2	Model 3	Model 4	Model 5	Model 6
Low condom efficacy	**4.34 (2.51–7.52)**	**4.03 (3.29–7.08)**	**4.02 (2.28–7.08)**	**3.87 (2.19–6.86)**	**4.00 (2.24–7.11)**	**3.94 (2.20–7.05)**
Female	1.27 (0.79–2.04)	1.29 (0.79–2.10)	1.29 (0.79–2.10)	1.32 (0.80–2.15)	1.25 (0.76–2.07)	1.33 (0.80–2.21)
≤23 years		**1.66 (1.01–2.73)**	**1.66 (1.01–2.73)**	**1.76 (1.07–2.91)**	**1.75 (1.06–2.90)**	**1.79 (1.08–2.97)**
Rural		**2.10 (1.31–3.36)**	**2.09 (1.30–3.35)**	**2.15 (1.33–3.45)**	**2.12 (1.31–3.42)**	**2.18 (1.34–3.54)**
Friends use condom with new partner			1.03 (0.63–1.67)	0.99 (0.60–1.61)	0.98 (0.60–1.61)	0.94 (0.57–1.55)
Friends have difficulty demanding condom use with new partner			1.05 (0.66–1.69)	1.06 (0.66–1.70)	1.08 (0.67–1.75)	1.09 (0.67–1.76)
Less pleasure with using a condom				**1.74 (1.04–2.92)**	**1.75 (1.04–2.93)**	**1.78 (1.06–3.00)**
Multiple sexual partners					0.79 (0.49–1.28)	0.69 (0.41–1.13)
Alcohol in relation to sex						**2.13 (1.19–3.82)**

In Table 4, we formally tested gender as an effect modifier regarding the association between condom efficacy and inconsistent condom use. The result confirmed the possible synergistic effect of gender, i.e. female gender aggravated the impact of low condom efficacy on inconsistent condom use.

**Table 4:** Analysis of effect modification between condom efficacy and gender regarding inconsistent condom use among Ugandan university students (*n*=1,179), presented as adjusted odds ratio (OR) with 95% CI

	*n* (%)	Cases	OR (95% CI)	
High condom efficacy/Male	485 (47.9)	125 (27.7)	1 (ref)	
High condom efficacy/Female	279 (27.5)	279 (27.5)	1.17 (0.84–1.63)	
Low condom efficacy/Male	138 (13.6)	138 (13.6)	**4.66 (3.08–7.07)**	
Low condom efficacy/Female	111 (11.0)	111 (11.0)	**7.55 (4.62–12.33)**	**Synergy index=1.71**
Missing	166			

## Discussion

Our study found inconsistent condom use to be more prevalent among females as compared to males. This may partially be explained by the lower prevalence of condom efficacy, an important determinant of consistent condom use among females. Moreover, the impact of low condom efficacy on inconsistent condom use was considerably higher among females, compared with the impact among males. All this suggests that condom efficacy is an independent determinant for consistent condom use among both males and females, but to a higher degree among females. This could be a significant factor behind the feminization of the HIV/AIDS epidemic in the study setting. The results also show that frequency of alcohol consumption in relation to sexual intercourse was a mediating variable between condom efficacy and inconsistent condom use, but no particular gender differences were observed regarding this determinant.

Young people in Tanzania showed gender differences regarding the predictors of condom use and its association with condom efficacy (30). For males, condom use depended on perceived self-efficacy, perceived self-efficacy for condom use with a long-term partner and having discussed condom use among friends. The predictors for females were discussing condom use with a sex partner and the perceived self-efficacy to refuse sex if the sex partner does not wish to use a condom (30). Therefore, it is evident that different factors effect condom use among men and women, and among them gender power relations do make a difference in condom use negotiation by women. This can also be supported by previous studies conducted on 18–49 year-old women in South Africa and Botswana (31) and on young people in Angola (32). A possible explanation for the gender differences regarding inconsistent condom use might be that condom use may be equated with lack of trust by men, leading to a fear of rejection on the part of women, which might result in non-use of condom (31). This in turn may expose women to the risk of unwanted pregnancies and STIs, including HIV.

Along with condom efficacy, other individual factors, such as reduced pleasure in sexual intercourse with a condom, showed a significant association with inconsistent condom use. This determinant was more common among men in our study. Our finding is supported by a meta-analysis, which shows that reduced pleasure is a robust predictor of non-use of condoms, where gender differences were observed, men reporting that using condoms reduces pleasure had a higher likelihood of non-use (33). There is evidence from previous research that explains the experience of sexual pleasure as a subjective reflection of a complex interplay of emotions, tactile sensations, and cognition, which limits its use among young people (34, 35). However, the difference between males and females in our study was not enough to make it a major explanatory factor for inconsistent condom use, especially since the impact on this behavior was of the same magnitude for males and females.

We found that *intention* to use a condom did not show a significant association with inconsistent condom use. This may be explained by the theory of planned behavior, which posits that behavioral intentions are determined by attitudes, subjective norms, and perceived control, leading to considerable variation in actual behavior under different circumstances (18, 19). Empirical evidence from studies of high school and university students in South Africa similarly found that intention to use a condom is determined by normative beliefs, attitudes, and subjective norms (17, 26). Gender differences with regard to intention have been observed among university students in a study that showed attitude as a better predictor of intention for young women, whereas men rely on subjective norms and their perception of communication and persuasion skills (36). Thus, behavioral intentions may depend more on individual factors for women, while for men social factors are more decisive (36). It appears that merely having the intention to use a condom may not translate into behavior, especially in a Ugandan context where attitudes and socio-cultural norms exert a strong influence.

In our study, alcohol consumption in relation to sex was found to mediate the association between low condom efficacy and inconsistent condom use with a new sex partner. University students who engage in risky alcohol consumption may thereby limit their ability to use a condom. As explained by alcohol myopia theory (37), a person who consumes alcohol experiences a restriction of their cognitive capacity. Such an individual focuses on salient situational cues of sexual initiation and ignores peripheral ones, making them less likely to identify potential dangers, like the risk of an unintended pregnancy or STIs. The consumption of alcohol on the occasion of sexual intercourse was infrequent in our sample, and the impact on inconsistent condom use was relatively similar in men and women. Thus, this factor did not seem to contribute to the observed gender differences in inconsistent condom use that we found.

### Strengths and limitations

One of the strengths of our study was that we addressed gender differences at the individual and social level in relation to condom efficacy and inconsistent condom use. This has not been well investigated previously in a Ugandan university population. A limitation of our study was the cross-sectional study design, as a result of which we could not judge the causal direction. According to our calculations of statistical power, the sample size was adequate for the main analyses, although somewhat small for assessing synergy, but no formal test of statistical significance was made for those analyses. Our relatively high response rate (72%) leaves room for a selection bias in our study. However, the reasons for non-participation do not seem to be linked to the main exposures or to the outcome, but were mostly caused by logistical circumstances. To reduce response bias in our study, the anonymity of the respondents was assured, which may have increased truthful reporting.

Another limitation of the study could be that sensitive questions regarding sexual behaviors might have been underreported, due to the issue of social desirability. If this was the case we believe it would bias the results towards the null, since it would more likely represent a case of non-differential misclassification than differential misclassification. In addition, our study results might not be fully generalizable to all countries, but we feel they may apply to university students in similar settings. In our analysis, we adjusted for the potential confounding factors, and therefore we believe that residual confounding would be of minor importance. Since our assessment of the exposure of condom efficacy was limited to two items, it is possible that if more items were added to the questionnaire we might have had greater disclosure on the efficaciousness of the participants.

## Conclusion

We found that low condom efficacy had an association with inconsistent condom use among male and female university students in Uganda. Females with lower condom efficacy were at a higher risk of inconsistent condom use compared to males. These findings have implications for policy formulation of young people's sexual and reproductive health in Uganda. Gender differences need to be taken into account in order to gain a deeper understanding of the factors that influence condom use in this region. With such knowledge, we can design and implement effective interventions against the spread of HIV/AIDS. It is imperative to acknowledge gender aspects when working towards improving condom efficacy of young people. The issue of gender equality should be addressed when designing intervention strategies that focus on sex education and counseling. These programs should aim at improving interpersonal communication that includes building condom use negotiation skills between partners.

## References

[1] Harman S (2011). The dual feminisation of HIV/AIDS. Globalizations.

[2] Gary B, Christine R (2005). Young men and construction of masculinity in sub-Saharan Africa: implications of HIV/AIDS conflict and violence. In: Social development papers, conflict prevention and reconstruction.

[3] Kaye DK (2004). Gender inequality and domestic violence: implications for human immunodeficiency virus (HIV) prevention. Afr Health Sci.

[4] Ugandan Ministry of Health and ICF International (2012). 2011 Uganda AIDS indicator survey: key findings. http://health.go.ug/docs/UAIS_2011_KEY_FINDINGS.pdf.

[5] EAC/EALP (2010). HIV sero-behavioural study in six universities in Uganda. http://195.202.82.11:8080/jspui/bitstream/123456789/216/1/UGANDA%20UNIVERSITIES%20HIV%20STUDY%20REPORT%20250112.pdf.

[6] Agardh A, Cantor-Graae E, Ostergren PO (2012). Youth, sexual risk-taking behavior, and mental health: a study of university students in Uganda. Int J Behav Med.

[7] Bandura A (2004). Health promotion by social cognitive means. Health Educ Behav.

[8] Bandura A (1986). Social foundations of thought and action.

[9] Black DS, Sun P, Rohrbach LA, Sussman S (2011). Decision-making style and gender moderation of the self-efficacy-condom use link among adolescents and young adults: informing targeted STI/HIV prevention programs. Arch Pediatr Adolesc Med.

[10] Bandura A, Peterson J, DiClemente E (1994). Social cognitive theory and exercise of control over HIV infection. Preventing theories and methods of behavioral interventions.

[11] Nalwadda G, Mirembe F, Byamugisha J, Faxelid E (2010). Persistent high fertility in Uganda: young people recount obstacles and enabling factors to use of contraceptives. BMC Public Health.

[12] Baele J, Dusseldorp E, Maes S (2001). Condom use self-efficacy: effect on intended and actual condom use in adolescents. J Adolesc Health.

[13] Walusaga HA, Kyohangirwe R, Wagner GJ (2012). Gender differences in determinants of condom use among HIV clients in Uganda. AIDS Patient Care STDs.

[14] Dilorio C, Dudley WN, Soet J, Watkins J, Maibach E (2000). A social cognitive-based model for condom use among college students. Nurs Res.

[15] Lindberg CE (2000). Knowledge, self-efficacy, coping, and condom use among urban women. J Assoc Nurses AIDS Care.

[16] Schaalma H, Aaro LE, Flisher AJ, Mathews C, Kaaya S, Onya H (2009). Correlates of intention to use condoms among Sub-Saharan African youth: the applicability of the theory of planned behaviour. Scand J Public Health.

[17] Mashegoane S, Moalusi KP, Peltzer K, Ngoepe MA (2004). The prediction of condom use intention among South African university students. Psychol Rep.

[18] Ajzen I (1991). The theory of planned behavior. Organ Behav Hum Dec.

[19] Conner M, Armitage CJ (1998). Extending the theory of planned behavior: a review and avenues for future research. J Appl Soc Psychol.

[20] Halpern-Felsher BL, Kropp RY, Boyer CB, Tschann JM, Ellen JM (2004). Adolescents’ self-efficacy to communicate about sex: its role in condom attitudes, commitment, and use. Adolescence.

[21] Perkins HW (2002). Social norms and the prevention of alcohol misuse in collegiate contexts. J Stud Alcohol Suppl.

[22] Melissa AL, Christine ML, Megan EP, Nicole F (2007). Gender-specific normative misperceptions of risky sexual behavior and alcoholrelated risky sexual behavior. Sex Roles.

[23] Amsale C, Yemane B (2012). Peer pressure is the prime driver of risky sexual behaviors among school adolescents in Addis Ababa, Ethiopia. World J AIDS.

[24] Le TN, Kato T (2006). The role of peer, parent, and culture in risky sexual behavior for Cambodian and Lao/Mien adolescents. J Adolesc Health.

[25] Sayles JN, Pettifor A, Wong MD, MacPhail C, Lee SJ, Hendriksen E (2006). Factors associated with self-efficacy for condom use and sexual negotiation among South African youth. J Acquir Immune Defic Syndr.

[26] Peltzer K (2000). Factors affecting condom use among senior secondary school pupils in South Africa. Centr Afr J Med.

[27] Agardh A, Emmelin M, Muriisa R, Ostergren PO (2010). Social capital and sexual behavior among Ugandan university students. Glob Health Action.

[28] Agardh A, Tumwine G, Asamoah BO, Cantor-Graae E (2012). The invisible suffering: sexual coercion, interpersonal violence, and mental health—a cross-sectional study among university students in south-western Uganda. PloS One.

[29] Rothman KJ, Greenland S, Lash TL (2008). Modern epidemiology.

[30] Babalola S (2006). Gender differences in the factors influencing consistent condom use among young people in Tanzania. Int J Adolesc Med Health.

[31] Langen TT (2005). Gender power imbalance on women’s capacity to negotiate self-protection against HIV/AIDS in Botswana and South Africa. Afr Health Sci.

[32] Prata N, Vahidnia F, Fraser A (2005). Gender and relationship differences in condom use among 15–24-year-olds in Angola. Int Fam Plan Perspect.

[33] Norton TR, Bogart LM, Cecil H, Pinkerton SD (2005). Primacy of affect over cognition in determining adult men’s condom-use behavior: a review. J Appl Soc Psychol.

[34] Randolph ME, Pinkerton SD, Bogart LM, Cecil H, Abramson PR (2007). Sexual pleasure and condom use. Arch Sex Behav.

[35] Abramson PR, Pinkerton SD (2002). With pleasure: thoughts on the nature of human sexuality.

[36] Munoz-Silva A, Sanchez-Garcia M, Nunes C, Martins A (2007). Gender differences in condom use prediction with theory of reasoned action and planned behaviour: the role of self-efficacy and control. AIDS Care.

[37] Steele CM, Josephs RA (1990). Alcohol myopia. Its prized and dangerous effects. Am Psychol.

# Sub article: ‘Men value their dignity’: securing respect and identity construction in urban informal settlements in South Africa

**DOI:** 10.3402/gha.v7.23676

**Published:** 2014-04-08

**Authors:** Andrew Gibbs, Yandisa Sikweyiya, Rachel Jewkes

**Affiliations:** 1 Health Economics and HIV/AIDS Research Division (HEARD), University of KwaZulu-Natal, Durban, South Africa; 2 Gender and Health Research Unit, Medical Research Council, Pretoria, South Africa

**Keywords:** masculinity, HIV, violence, gender, multiple sexual partners, livelihoods, IPV, unemployment

## Abstract

**Background:**

Urban informal settlements remain sites of high HIV incidence and prevalence, as well as violence. Increasing attention is paid on how configurations of young men's masculinities shape these practices through exploring how men build respect and identity. In this paper, we explore how young Black South Africans in two urban informal settlements construct respect and a masculine identity.

**Methods:**

Data are drawn from three focus groups and 19 in-depth interviews.

**Results:**

We suggest that while young men aspire to a ‘traditional’ masculinity, prioritising economic power and control over the household, we suggest that a youth masculinity emerges which, in lieu of alternative ways to display power, prioritises violence and control over men's sexual partners, men seeking multiple sexual partners and men's violence to other men. This functions as a way of demonstrating masculinity and their position within a public gender order.

**Discussion:**

We suggest there are three implications of the findings for working with men on violence and HIV-risk reduction. First, there exist a number of contradictions in men's discourses about masculinity that may provide spaces and opportunities for change. Second, it is important to work on multiple issues at once given the way violence, alcohol use, and sexual risk are interlinked in youth masculinity. Finally, engaging with men's exclusion from the capitalist system may provide an important way to reduce violence.

Rapid urbanisation has led to burgeoning informal settlements, as cities and states have been unable to effectively create permanent infrastructure for growing populations (1, 2). WHO and UN-HABITAT estimated in 2010 that 63% of urban dwellers in sub-Saharan Africa lived in informal settlements (3). In South Africa, the 1980s saw a rapid growth of urban informal settlements, when the apartheid government stopped controlling the mobility of the Black majority with repeal of the influx control legislation, but failed to meet permanent housing needs (4). Current estimates for South Africa suggest that 4.4 million people live in informal settlements, approximately 23% of all households (5). In eThekwini district, KwaZulu-Natal – the location of this study and the third largest city in South Africa – an estimated 25% of the population live in informal housing (5).

Urban informal settlements are often settings with high levels of violence, poverty, poor health, and HIV (4, 6–8). UNAIDS estimates that 28% of people living with HIV/AIDS in southern and eastern Africa live in 14 cities in the region (approximately 15% of the global epidemic) and in South Africa the HIV prevalence in informal settlements is twice that of people in formal housing (9, 10). While there remains little comparable data on rates of gender-based violence in urban and rural areas (11), one study from Cape Town, South Africa, explored rates of homicide within the city, comparing different settlement ‘types’ and showed informal urban settlements had rates over four times that of wealthier, formal settlements (3).

There has been considerable debate about why urban informal settlements have particularly high levels of violence and ill-health and the role of place in health outcomes more generally (12). One strand of this argument emphasises the experience of living in high-density communities, leading to stress and an inability to control aspects of life, as a key factor shaping violence (11). Another argument is that in informal settlements there is less social cohesion, caused by poverty and mobility, creating less stable forms of power, in which violence becomes a necessary resource to wield as previously stable configurations of power – particularly gender power – get challenged (3, 11).

## Masculinities and violence

Globally, researchers are increasingly studying constructions of masculinity, including men's perpetration of violence, which place them and their partners at increased risk of acquiring HIV (13, 14). Firmly located within a critique of gender inequalities, studies have observed and sought to explain the clustering of men's violence and HIV-risk practices (15). In South Africa, a representative population-based study of South African men found those who had been violent to a partner to have less gender equitable masculinities, more likely to have raped and more likely to have engaged in transactional sex (16). Specifically in the study, among those under 25 years, those who had been violent to a partner had a higher prevalence of HIV (16). Similar links between violence, rape, and gender inequitable masculinities have also been shown in the Asia–Pacific region (17) and Latin America (18, 19).

Theorising this clustering of risk, violence and gender inequitable masculinities researchers have largely drawn on Connell's (20) notion of hegemonic masculinity, building a relational construction of gender inequalities (21). Within a context of patriarchal privilege, Connell argues that in any social setting there is a collectively held understanding of ideal male practices. The majority of men view the ideal as an aspiration, something that influences their practices and structures men's understandings of themselves and their behaviours, without necessarily being achievable or desired in its entirety for all men (20). In response, men construct a range of masculinities allowing them to establish viable alternatives to the hegemonic masculinity, while at the same time often supporting its overall logic (20).These hegemonic ideals also influence the behaviour of women as although they are subordinated by men, they shape their views of a desirable ideal and thus men who do not aspire to adopt the hegemonic masculinity may be penalised in their attractiveness to women.

Men's behaviours, including violence and HIV-risk-related practices, can be understood as men attempting to position themselves both individually and publically in relation to hegemonic masculinity, which forms a gender hierarchy (19, 22, 23). Critical men's studies also point towards how such health behaviours actively constitute forms of masculinity (24). From a social–psychological perspective, some researchers are concerned with how these broad macro-processes become embedded in individual's psyches and how these are internalised and resisted (25). In the context of high levels of poverty, a strong argument has been made that young men construct a subordinated masculinity, focused on heterosexual performance and violence as a way of building their sense of self-worth and positioning themselves within the gender and broader social order of these socially subordinated spaces (20, 26, 27). Less often commented on is how gender hierarchies intersect with age hierarchies and violence can often be seen as situated at the intersection of these axes as well (27).

While much work accentuates men's power, dominance, and use of violence against women and other men, another set of work emphasises the emotional lives and vulnerability many of these men living in poverty feel (28, 29). This has led some to suggest that men's violence emerges from a profound sense of powerlessness (30) with men seeking power in ways that are accessible to them and socially condoned. Some researchers have sought to trace men's ‘long histories of violence’, through exploring men's childhoods that are harsh and leading to ‘attachment disorders’, which tend to reduce men's empathy and guilt. In so doing, they suggest that the patterns of violence and other risk behaviours are setup in childhood psychological development processes but then enabled through social process and contexts – such as patriarchy – to support men's violence against women and other men (31).

## Masculinities in South Africa

Within South Africa a number of ethnographies have sought to understand how men construct and sustain masculine identities and respect in a variety of contexts. Hunter's (4) work suggests that in the 1970s and 1980s a new ‘traditional masculinity’ emerged for Black South African men employed in working class jobs as industrialisation occurred. This masculinity centred on a benign heterosexual patriarchy in which masculine respect was underpinned by male economic provision (4). This reworked older notions of masculinity locating them in urban settings. Central to this was men's ability to provide for a household with homes becoming a measure of masculinity (4, 32). Male power was also articulated through asserting social control over women and children. According to Hunter (4) this masculinity continues to dominate the gender hierarchy for many working class Black South Africans, potentially forming a hegemonic masculinity (20).

As much research on masculinities has emphasised, for the majority of men (if not all), the ‘hegemonic masculinity’ cannot be achieved and a multiplicity of masculinities flourish (21). Studies in South Africa have explored alternative ways of building masculine identity and respect. Reihling (33) looks at how men living with HIV construct new forms of what he calls ‘relational dignity’ through health activism, creating a new form of masculinity in so doing. A small number of studies have sought to explore youth masculinities and health in contemporary South Africa. Wood and Jewkes (34), for instance, in the Eastern Cape Province of South Africa argue that economic marginalisation of young men has led to a distinctive youth masculinity emerging, where masculinity became centred on controlling main female sexual partners, with violence used if necessary. Similarly, Ragnarsson et al.'s (8) work in peri-urban communities emphasises how small male groups are the central locus for this production of a patriarchal youth masculinity, in which men in lieu of alternative sources of power and dignity turn to seeking multiple sexual partnerships as a way of securing their masculinity among other men. While Hunter's work (4, 35) also exploring younger men, emphasises how young men negotiate the tensions between their expected roles as providers in romantic relationships and their lack of economic power through subtle negotiations and an emphasised heterosexuality.

In this study, we build on this body of work to explore how young Black South African men, living in contexts of poverty in urban informal settlements, seek to construct, and sustain a viable sense of respect and masculine identity through their relationships with others focused on the intersections of sexuality and violence. We are concerned throughout with how men evaluate themselves and position themselves within gender and age hierarchies.

## Methods

### Setting

The young men in the study lived in two urban informal settlements in eThekwini District, KwaZulu-Natal, South Africa. Broadly informal settlements in South Africa have poor services; 2001 data suggested that only 26% of dwellings in informal settlements had piped water in their dwelling or yard and 32% had electricity (5) and the two settlements reflected this. The majority of young men came from a slightly older and more established settlement, Little Japan. Little Japan had a number of government provided single room houses (called RDP-houses) sitting alongside smaller shacks and single room dwellings. It was located alongside a main highway, which ran past a shopping mall and large township, approximately 10 min away by taxi. There was a regular public taxi to the centre of Durban taking about 25 min. Despite this Little Japan's roads were primarily untarred and there was little formal electricity and no inside toilets. The second community was Mbazwana and significantly poorer than Little Japan. This was a new settlement, only settled in the previous 10 years, located on a steep hillside. Transport links into Durban and to industrial areas were weak. Residents of Mbazwana had to catch two public taxis to central Durban, taking about 45 min. There was also no formal electricity, pathways, or toilets in Mbazwana.

### Participants

Men were aged between 18 and 27 years, with the majority under 25. A few had formally finished education with a high school qualification, but most had exited education early, and few had further skills training. None of the men in the study had permanent work; rather the majority relied on temporary formal work (primarily shop work or construction), informal work (such as selling small items at the side of the road or working on public taxis), or a variety of illegal activities (selling drugs or petty crime). This work was poorly paid and highly precarious. Nationally representative household data from 2006 highlight the casualised nature of work in informal settlements (36). These data also suggest that average wages in informal settlements were R1,703 per month compared to R2,945 in formal housing (36). Many of the men also relied on their family to support them financially. All of the men reported that they had a main female partner at the time of the interviews and a number had a child with this partner or a previous partner.

### Data collection

Data for this paper come from three focus group discussions (FGDs) conducted with 44 men and 19 in-depth interviews (IDIs) conducted over 2 months in 2012. FGDs enable collectively held views and understandings of salient issues to emerge – what we may call public transcripts – while IDIs enable the complexities and ambivalences of real lives to emerge, without men feeling compelled to construct public identities (37).

Data were collected at baseline for a formative evaluation of a behavioural and structural intervention – Stepping Stones and Creating Futures (38). Participants were recruited by Project Empower, an NGO based in eThekweni, which ran the intervention. Open community meetings were held at which the intervention was explained and flyers circulated. As such, participants self-selected to participate in the study. A convenience sample was used for the FGDs; we approached all of the men who enrolled in the study in the first three days and requested their participation in FGDs, 44 men agreed. While FGDs were large (ranging from 12 to 20 participants), it enabled an exchange of views and ideas to emerge. As Tang and Davis (39) suggest there is no optimal size for FGDs as long as sufficient time and facilitation is in place to enable a meaningful exchange of ideas to occur. From the 110 men who enrolled in the intervention, we randomly selected 20 men to participate in IDIs – 19 men agreed. We randomly chose men for IDIs as we then followed men up over the course of 1 year to understand their overall experience of the intervention and did not wish to introduce bias into our selection.

The IDIs and FGDs covered similar topics. They focused on the intersection between masculinities and livelihoods and how this shaped men's lives and relationships. Specifically they included discussions on how men made money and survived on a daily basis and what they aspired to do in the future. Questions probed what men felt it meant to be a man in their community and whether they achieved this or not. The topic guide then moved onto relationships with women, especially sexual partners before asking about violence in the community and in their relationships. IDIs typically lasted about 45 min, ranging from 20 min to 1.5 hours. FGDs lasted between 1 and 1.5 hours. All FGDs and IDIs were conducted in isiZulu, the dominant language in the study locations, and were digitally recorded and translated and transcribed by the male fieldworker who undertook them.

### Data analysis

Thematic content analysis was conducted drawing on Attride-Stirling's approach of thematic network analysis (40). Broadly, transcripts were read repeatedly before initial codes were developed (based on words or short ideas) (41). Codes were then clustered into groups focused on how men understood respect and sought to achieve it. Triangulation was achieved by comparing and contrasting FGDs and IDIs to examine both public and private understandings and expressions of masculinity. These were then centred on two networks identified as ‘traditional masculinity’ and ‘youth masculinity’. Such an approach allows the researcher to make connections between different ideas and link to theory rather than simply describe data (40).

### Ethics

Ethical approval was given by the South African Medical Research Council (EC003-2/2012) and the University of KwaZulu-Natal's Human and Social Science Ethics Committees (HSS/0789/011 and HSS/1273/011D). Written informed consent was obtained from all participants. The names of study participants and locations have been replaced by pseudonyms to protect the identity of the participants. No payment was given to participants for participating in the intervention or FGDs. However, for IDIs a small meal was bought by the research assistant and shared as a way of building rapport.

## Findings

The men identified with a ‘traditional’ masculinity premised on economically providing in relationships, in which men were positioned as benevolent patriarchs. Yet young men's inability to secure work, left them socially positioned as children. As a reaction to this, the men were drawn to a particular youth masculinity that emphasised respect through violence against partners, control of partners, seeking multiple sexual partners, and violence against other men.

### ‘Traditional’ masculinity

Young men aspired to a ‘traditional’ masculinity, closely linking masculinity to provision for a family and partner and control over them. Gwedi, for instance, saw manliness as embodied by having a home and control over the family:

Interviewer: What characteristics does a person need to have in order to be described as a man in your community?

Gwedi: You know there is no other way my brother, you must have a wife, a house, and money, and again to see how well behaved you are when you are a man you must be straight [strict]. (IDI, 24, petty drug seller)^1^

Economic independence was prized by men as it enabled them to set up a household. Borrowing money, rather than working for it, as many of the young men did, was seen as a sign of failure as Thokozani commented:

Interviewer: What does it mean to be a man?

Thokozani: I have to be responsible and be independent, respectable in the community.

Interviewer: What do you mean by independence?

Thokozani: Like having my own house. Not being a person that is always borrowing money. (IDI, 19, supported by parents)

Among informants, the use of violence to settle disputes among men was discussed. For some violence, owning guns and knives and a willingness to wield violence remained important. However, for most the ‘traditional’ masculinity was gentler and prioritised aspects of love, kindness, and engagement with children, as well as limiting violence as Bongani emphasised:

Interviewer: What makes a successful man in your community?

Bongani: It is the way he carries himself [the way he behaves], having respect…

Interviewer: How is he to his family?

Bongani: He is a disciplined man. He has a wife and it does not mean just because you have a wife you cannot wash dishes, a man is able to talk well with his wife, not violently, and his kids love him as a father. (IDI, 25, informal shop)

Broadly, men in the study still aspired to a ‘traditional’ masculinity in which power was conferred to them through economic independence and social dominance, essentially creating a hegemonic masculinity.

### Men without respect

Young men, however, were aware that the ‘traditional’ masculinity was aspirational and something they struggled to achieve. Men described how they were often highly dependent on their families for financial support – primarily mothers or grandmothers. As Thabo described, this dependency undermined his sense of confidence and masculinity:

Thabo: The thing is my grandmother, she buys me food, she dresses me and she supports my child. Now to think of asking her for money, let's say me and my friends want to buy booze and party, to me that is a problem.

Interviewer: Has asking money from your granny caused you any problems?

Thabo: I'm too dependent on her, whilst I should be independent. (IDI, 23, piece work)

Without formal work, young men spent much time ‘hanging around on streets’. This enabled public ‘devaluation’ of the men by others in the community, who did not take them seriously as they did not work. As Mboniswa suggested, men without work were viewed as useless, as less than men, as they could not support a family:

Interviewer: How do they view a man who does not work?

Mboniswa: They view him as a useless man. Like someone you cannot depend on or look up to. They would ignore him, not take him seriously and look down upon him, or as someone that does not exist in the community. (IDI, 23, informal work)

Within the public gender and age hierarchy that existed within the communities, a lack of access to work placed young men low down. Indeed many, including Wiseman, stressed how they were seen as children as they did not conform to the ideals of masculinity:

Interviewer: How does the community treat you if you don't meet the characteristics of being that man?

Wiseman: Okay, yes, you are undermined. Like you are just a man because you wear pants [trousers] nothing more. You are looked down upon, even little boys undermine you, they treat you like you are at their age, because you are useless. (IDI, 18, temporary work)

Of particular concern for young men was their inability to provide in sexual relationships, as they felt was expected of them. Sandile described both the frustration and embarrassment that was caused when he could not provide basic items and how women looked down upon young men like himself:

Interviewer: What problems are there for a man when he does not have money?

Sandile: Most of the time women depend on men, so if you are a man and you don't have money, even when a woman is asking for something to wear or a perfume and you are not able to provide with that, it becomes a problem. It is an embarrassment.

Interviewer: What happens to you as a man when that happens?

Sandile: Your dignity is crushed and women badmouth you, like saying: ‘that man is just using me, he does not give me money, he doesn't do anything for me, he is just using me [for sex].’ (IDI, 24, temporary jobs)

Within urban informal settlements, young men were acutely aware of how others positioned them within the gender hierarchy and how they were positioned as children for failing to achieve what was expected of men.

### Building respect in informal settlements

In their communities, men struggled to establish themselves both as men in public settings and build their own sense of self-confidence and respect. In turn, men sought to construct an alternative identity predicated on the sources of power that they could access, primarily located around heterosexuality and violence. We identify four main aspects informing a dominant youth masculinity: 1) men's main sexual relationships, 2) violence and control over female partners, 3) having multiple partners and thus demonstrating desirability to women, 4) public violence. Each of these, in their own ways, enabled men to achieve a sense of respect in public and private contexts.

#### Men's main long-term sexual relationships

The majority of men said they had a long-term female sexual partner. As men spoke about these relationships, they sought to frame them in similar terms to how they had spoken about relationships within the ‘traditional’ masculinity they aspired towards, even if they could not achieve this. Almost all interviewees identified a woman they saw as a main partner, often someone they had a child with, and specifically someone they saw as having a future together with. They were able to distinguish these women and the relationships they had with them, from other relationships they had with other women, which were often shorter and more focused on sexual exchange.

Men were emotionally invested in these long-term relationships. Many reflected on how they would feel if these relationships ended, emphasising the emotional pain they would feel. Gwedi had two girlfriends; the first was his main partner with whom he had a child. The second was a younger woman who he saw occasionally. He described the different responses he would feel when asked to imagine what would happen if these relationships ended:

Interviewer: If one of your girlfriends wanted to leave you, what would happen? Let's say your baby mama [main partner and mother of his first child]?

Gwedi: Without a reason?

Interviewer: Whether or not without a reason. I want to know what would happen if one of them wanted to leave you?

Gwedi: I would be sad if it is my baby mama because you know I have invested my future with her since I want to go far with her. My heart would be broken but I would try and ask her not to leave me, but everything would be up to her because the person with the last decision would be her.

Interviewer: And the second one?

Gwedi: The second one if she wanted to leave me?

Interviewer: Yes.

Gwedi: The second one if she wanted to leave me it's not like I would be too heartbroken. Though I would be sad because she is the one close by for booty call [sex], I must say that would be sad in that sense, but she is not like that important to me. (IDI, 24, petty drug seller)

Men placed significant emphasis on trust and love in main relationships, symbolised by women and men typically not wanting to use condoms: ‘I will make an example with the guys I hang out with, they say they don't use condoms with their main partners because they trust them, then the other girlfriends they don't care about, they use condoms with them’ (Participant, focus group 1). Introducing condoms into these relationships signalled a breakdown in trust and love, tantamount to admitting these relationships were not the monogamous idealised relationships men sought to portray and sustain.

#### Violence and control over female partners

In the FGDs and interviews, men spoke openly about how they used a range of techniques to control their female partners, including violence. Men's use of violence against their partners was closely linked to a range of controlling behaviours and almost always positioned as an active strategy by men to achieve respect and social position that they felt they had been denied.

Men's controlling behaviours towards their main partners attempted to limit women's autonomy. Often this was done, according to men, because they feared women would ‘cheat’ on them with other men; an inability of men to control their partners, devalued men's sense of themselves. Controlling behaviours included checking cell phone messages, screening calls, and making calls throughout the day and night and expecting immediate answers. Sandile explained he trusted his main girlfriend because no matter what time he called she would answer her phone and talk to him:

Sandile: Since my girlfriend stays very far from my community, so like every time I call her she will always pick up my calls, and we talk for a very long time. It does not matter what time I call, she does not have a problem, like making excuses if she has a man around her you know and all that. I have never caught her doing anything wrong, like with a man [cheating], and all the silly things. (IDI, 24, temporary jobs)

When men's controlling behaviours failed to achieve what was wanted, young men readily described using violence as a way of re-establishing both the gender order – women's subordination to men – as well as re-establishing men's respectability within a social hierarchy, as Sandile emphasised when asked why men were violent to their partners:

Sandile: I may not explain exactly why but, from what I have observed, it is because of the girlfriends that misbehave, then that leads to them getting a beating, like a man would say: ‘You are misbehaving, you don't respect me’. (IDI, 24, temporary jobs)

Participants identified a wide-range of ways in which they felt women disrespected them and where violence could legitimately be used to reassert men's respect and dignity. Many focused around men's concerns that women would cheat on them. Other ‘reasons’ included women's growing economic autonomy and a concern that this would lead to women disrespecting men, with violence used to reassert male power:

Mthobisi: When a woman, like she is working, and I am not working, and she starts disrespecting and being rude to me, we then fight then like I end up hitting her because I try to defend my dignity as a man. (IDI, 22, rents a room, sister supports)

Women refusing to have sex with a male partner also was potentially a source of violence, reflecting ideas of sexual entitlement, although many men said this was something they accepted. One participant, Gwedi, described how one evening his second female partner (not his main partner) came over, but did not want to have sex with him. Gwedi felt that the only way of dealing with this affront (which also implied that she had another partner) was to beat her as he had been humiliated:

Gwedi: I had to lay a hand on her [hit her] because of what she did. She came to my house at night drunk, and I wanted to have sex with her, and she denied me sex because she was drunk … then I waited until the morning, and at that point it had been days since I had had sex with her, so like now in the morning like I wanted some, because I had been longing to have sex with her, so she pretended she was going outside to pee [there are only outside toilets] … I realised she was not coming back, she was going home. So I chased after her I then grabbed her, I slapped her for the fact that she was running away, but I ended up not sleeping with her, because she was then talking about police and all that [laughing]. So I beat her up for making me a fool, because she should have said she does not want to have sex with me straight up, you see what I mean? (IDI, 24, petty drug seller)

Violence and controlling behaviours enacted by men against their female partners were widely described by men as an attempt to reassert their dignity and respect in relation to women.

#### Multiple sexual partners

The central role for young men living in urban informal settlements in seeking multiple sexual partners to establish their identity was evident. Having multiple partners was normalised. For Thokozani, it was something that men just needed:

Thokozani: But you know a woman can have one or two partners. But men cannot live without having more than one partner and there are very few of them that can live with only one. (IDI, 19, supported by parents)

While a few participants suggested a ‘cultural’ basis for multiple sexual partnerships, the major emphasis was that multiple sexual partners were a way of earning respect from their peer-group. One focus group participant commented on why men had many partners: ‘they can be complimented for being a real man’ (focus group 3). Another, China, similarly suggested that having multiple girlfriends earned you respect, affirmation, and dignity from your peers:

China: If you have one girlfriend you are a coward; most of them do it for pride and they do it so that they can get respect and for the dignity and when you have many girlfriends it means you get respect. (Focus group 3)

The performative nature of seeking and securing multiple sexual partners was particularly evident in the way short-term, one-off sexual encounters were described by these men. Mthobisi described how these were linked to parties and drinking alcohol and proving to your friends that you were able to be successful sexually:

Mthobisi: You know at the parties, condoms are the last thing people think of when they are drunk and then you go and have sex with the girls and end up contracting HIV because of the fact that you were trying to please friends. (IDI, 22, rents a room, sister supports)

Having multiple sexual partners was a public performance of heterosexuality, proof of desirability, and thus masculinity. As such, they provided a pathway open to these young men for building up a sense of respect.

#### Defence of honour: men's violence to other men

A final way men talked about achieving public respect and proving masculinity was demonstrating a readiness to defend their honour through violence towards other men. Typically alcohol was also involved; however, violence occurred when men felt they had been slighted by another man and needed to defend their dignity. Mthobisi described how fighting emerged because men felt the need to not lose face or back down if they had been disrespected:

Interviewer: Who do men get violent towards?

Mthobisi: Towards other men, if like you have lowered his dignity as a man…

Interviewer: Can you give me an example?

Mthobisi: If you come and look down upon me and be rude, swear or talk nuisance to me, obviously I will have to defend my dignity I will then stand up and confront you and if we fight, we fight.

Interviewer: Why do men get violent?

Mthobisi: Most of the time it's because they are drunk or it is because they are just short tempered, there are those that are like that who when you speak to them they just answer you for the sake of just answering you, they are not open.

Interviewer: Why do they fight with each other?

Mthobisi: It's pride my brother you know men value their dignity, I will also return the favour hurt and injure you just so I can get my dignity back as a man. (IDI, 22, rents a room, sister supports)

Similarly Goodman described how an argument could easily escalate into a fight, particularly if alcohol was involved:

Interviewer: So who are men violent towards?

Goodman: Each other.

Interviewer: Why?

Goodman: You know you will find that one person steps on the other and the one being stepped on would say ‘can't you see you stood on my toe’ and the second guy would say: ‘I'm sorry’, then the first one would try and provoke the other one since he is drunk and because maybe he has a grudge with the second guy or something like that. And maybe the second one would end up saying: ‘I said I'm sorry, what do you want me to say’ and if the first one keeps pushing, then the second one would say, ‘what are you going to do’ then the fight starts over that little incident. (IDI, 25, supported by mother)

Men's violence to one another was very public and linked closely to men's overarching concern to position themselves within a dominant gendered hierarchy. While alcohol often fuelled this violence, men felt they could not ‘back down’ without losing respect.

## Discussion

In this study, we have sought to understand how young men in two urban informal settlements in eThekweni, South Africa, construct and maintain one particular set of social and sexual identities in the face of high levels of unemployment and poverty, recognising the relational nature of masculinities and their multiplicity in any given setting. Broadly we have suggested that while these young men aspired to a ‘traditional’ worker masculinity forged in the 1970s industrialisation in South Africa, with its emphasis on economic power to setup and sustain a household, including assertion of power over women and children (4), their ability to do so was severely compromised because they lacked the material power to do so.

In turn, young men sought out other ways of building their sense of power and respect in response to the life challenges they faced and their inability to obtain other sources of respect. Principally young men ‘on the wrong side of history’ (42) established a subordinated masculinity, much the same as outlined by Wood and Jewkes (34) in the Eastern Cape of South Africa. This youth masculinity prioritised, in lieu of power through setting up and sustaining a home, education, or wealth, power in spaces that young men could achieve, most evidently through asserting power and control over women, particularly main sexual partners and seeking multiple sexual partnerships and violence towards other men. These practices, similarity to those described by Wood and Jewkes (34), suggest a commonality of how marginalised youth in South Africa attempt to position themselves within a gender hierarchy in contexts of poverty and unemployment.

That these sources of power are the only ones available to young men in these contexts, emerges from a long history of economic, political, and social exclusion of, and violence directed towards, young Black South Africans (43), and the continued dominance of conservative patriarchies in South Africa, as well as the inter-generational production of trauma and violence, experienced by many young men (31).

Of note, however, is that young men expressed significant emotional investment in their long-term relationships with main female sexual partners. This contrasts sharply to much writing on young men, which emphasises the extractive nature and lack of emotional engagement in men's relationships (8, 28). It also points to how men draw on a range of discourses of masculinity in different relationships (19) suggesting that there may be discourses and opportunities for change already embedded in men's everyday practices. However, as Wood and Jewkes (34) suggest, men's investment in these relationships is also a way of demonstrating masculinity, with men's ability to retain and control women being critical to them.

The youth masculinity described in the data also contrasts with that described by Hunter (4, 35). Hunter suggests that in the face of HIV, young men in KwaZulu-Natal are starting to modify their sexual behaviours as organic responses to risk. However, our data suggest that for many men, this is not happening, with the pressure to achieve respect and social positioning in the gender order outweighing other priorities.

For young men in urban informal settlements, their sense of masculinity and positioning within the gender hierarchy was very publicly achieved and evaluated; something men ‘wore on their sleeve’ and performed. In very different contexts Vandello and Bosson (44) suggest that masculinity is, in the most part, extremely precarious, something that is ‘Hard Won and Easily Lost’. While not directly emphasising masculinities performative nature, such an argument resonates with Butler's (45) notion of gender being a performative category (albeit one performed within material and political constraints). Indeed, the young men in this study certainly continued to perform their masculinity on a daily basis, recognising how they were publicly evaluated. This may have been compounded by the very public nature of everyday life in the two informal settlements. As young men lived in small, one room shacks, often shared with others, they had few private spaces into which they could retreat and enact alternative forms of masculinity, outside the gaze of dominant social and gender norms. While much writing has explored how place shapes health (6, 11, 12, 29), little has considered how the public nature of life in urban informal settlements and the lack of private spaces may contribute to certain configurations of gender practices emerging.

The argument set out in this paper has three implications for working to reduce violence and HIV risk more broadly with men. First, even within the youth masculinity that we describe, there existed a number of contradictions and opportunities to support more gender equitable – or at least less harmful – masculinities, ones emphasising trust, love, and long-term commitment. As has been pointed out (19), these provide discourses for interventions to draw on and build from and point to the fluid and multiple nature of masculinities in any given setting.

Second, given the way a youth masculinity coalesces around a number of particular practices, interventions need to work around multiple issues if they are seeking to reduce violence and HIV risk. It is unlikely that changing men's violent behaviour will occur outside of working with them around alcohol use, drug use, multiple sexual partnerships, because these all coalesce around a particular form of youth masculinity.

Finally, as other studies have suggested, violence and other HIV-related risk practices partially emerge from young men's exclusion from the global capitalist economy 46. With a dominant approach to achieving respect cut off for these young men, they cast around for alternative pathways; one of which included what we describe as a youth masculinity. Work from the global peripheries of the capitalist system, including Mozambique (26) and Brazil (18), all point towards how men's violence is implicated in these processes of exclusion (often overlapping with racism). Yet similar dynamics are also seen within the heart of global capitalism. As Bourgois (27) outlines in his ethnography of drug dealers in New York, young Porto Rican men excluded from the capitalist economy secure respect through the only available pathways, dealing drugs and public and private uses of violence. Given these global processes are inflected with local dynamics, there remains much to be learnt about what building young men's livelihoods would look like and whether this would have any bearing on violence and HIV-risk behaviours. More work is also required on how best to work with young men, invested in a contemporary form of youth masculinity which prioritises violence, control, and multiple sexual partnerships to support these young men to change and develop less harmful forms of masculinity in the contexts of poverty and significant life challenges.

## Footnotes

1 Information provided: IDI (in-depth interview), age, primary source of income.

## References

[1] Myers G (2011). African cities: alternative visions of urban theory and practice.

[2] WHO (2008). Our cities, our health, our future: acting on social determinants for health equity in urban settings.

[3] WHO, UN-Habitat (2010). Hidden cities: unmasking and overcoming health inequities in urban settings.

[4] Hunter M (2010). Love in the time of AIDS: inequality, gender, and rights in South Africa.

[5] Housing Development Agency South Africa (2011). Informal settlements – KwaZulu-Natal.

[6] Hunter M (2007). The changing political economy of sex in South Africa: the significance of unemployment and inequalities to the scale of the AIDS pandemic. Soc Sci Med.

[7] Thomas L, Vearey J, Mahlangu P (2011). Making a difference to health in slums: an HIV and African perspective. Lancet.

[8] Ragnarsson A, Townsend L, Ekstrom A, Chopra M, Thorson A (2010). The construction of an idealised urban masculinity among men with concurrent sexual partners in a South African Township. Glob Health Action.

[9] van Renterghem H, Jackson H (2009). AIDS and the city: intensifying the response to HIV and AIDS in urban areas in sub-Saharan Africa.

[10] Shisana O, Rehle T, Simbayi L, Zuma K (2009). South African national HIV prevalence, incidence, behaviour and communication survey, 2008.

[11] McIlwaine C (2012). Urbanization and gender-based violence: exploring the paradoxes in the global South. Environ Urban.

[12] Davidson R, Mitchell R, Hunt K (2008). Location, location, location: the role of experience of disadvantage in lay perceptions of area inequalities in health. Health Place.

[13] Gupta GR, Ogden AS, Warner A (2011). Moving forward on women's gender-related HIV vulnerability: the good news, the bad news and what to do about it. Glob Public Health.

[14] Phillips A, Pirkle C (2011). Moving beyond behaviour: advancing HIV risk prevention epistemologies and interventions (A report on the state of the literature). Glob Public Health.

[15] Jewkes R, Morrell R (2010). Gender and sexuality: emerging perspectives from the heterosexual epidemic in South Africa and implications for HIV risk and prevention. J Int AIDS Soc.

[16] Jewkes R, Sikweyiya Y, Morrell R, Dunkle K (2011). Gender inequitable masculinity and sexual entitlement in rape perpetration South Africa: findings of a cross-sectional study. PLoS One.

[17] Fulu E, Jewkes R, Roselli T, Garcia-Moreno C (2013). UN Multi-Country Cross Sectional Study on Men and Violence. Prevalence of and factors associated with male perpetration of intimate partner violence: findings from the UN Multi-country Cross-sectional Study on Men and Violence in Asia and the Pacific. Lancet Glob Health.

[18] Barker G (2005). Dying to be men: youth, masculinity and social exclusion.

[19] Fleming PJ, Andes K, DiClemente R (2013). ‘But I'm not like that’: young men's navigation of normative masculinities in a marginalised urban community in Paraguay. Cult Health Sex.

[20] Connell R (1999). Masculinities.

[21] Connell R (2012). Gender, health and theory: conceptualizing the issue, in local and world perspective. Soc Sci Med.

[22] Courtenay WH (2000). Constructions of masculinity and their influence on men's well-being: a theory of gender and health. Soc Sci Med.

[23] Hirsch J, Hirsch J, Wardlow D, Smith D, Phinney H, Parikh S, Nathonson C (2009). The geography of desire: social space, sexual projects, and the organization of extramarital sex in rural Mexico. The secret: love, marriage, and HIV.

[24] Lohan M (2007). How might we understand men's health better? Integrating explanations from critical studies on menand inequalities in health. Soc Sci Med.

[25] Campbell C, Gibbs A, Boesten J, Poku N (2009). Stigma, gender and HIV: case studies of inter-sectionality. Gender and HIV/AIDS: critical perspectives from developing countries.

[26] 
Gores-Green C (2009). Hegemonic and subordinated masculinities: class, violence and sexual performance among young Mozambican men. Nord J Afr Stud.

[27] Bourgois P (2002). In search of respect: selling crack in El Barrio.

[28] Seidler V (2005). Transforming masculinities: men, cultures, bodies, power, sex and love.

[29] Meth P (2009). Marginalised men's emotions: politics and place. Geoforum.

[30] Ratele K (2013). Subordinate Black South African men without fear. Cahiers d'Etudes africaines.

[31] Mathews S, Jewkes R, Abrahams N (2011). ‘I had a hard life’: exploring childhood adversity in the shaping of masculinities among men who killed an intimate partner in South Africa. Br J Criminol.

[32] Banks L (2011). Home spaces, street styles: contesting power and identity in a South African City.

[33] Reihling H (2013). Positive men: searching for relational dignity through health activism in a South African township. Soc Dynam.

[34] Wood K, Jewkes R, Morrell R (2001). ‘Dangerous’ love: reflections on violence among Xhosa township youth. Changing men in Southern Africa.

[35] Hunter M (2005). Cultural politics and masculinities: multiple-partners in historical perspective in KwaZulu-Natal. Cult Health Sex.

[36] Hunter M, Posel D (2012). Here to work: the socioeconomic characteristics of informal dwellers in post-apartheid South Africa. Environ Urban.

[37] Pattman R, Chege F (2003). ‘Dear diary I saw an angel, she looked like heaven on earth’: sex talk and sex education. Afr J AIDS Res.

[38] Jewkes R, Gibbs A, Willan S, Misselhorn A, Jama N (2013). Stepping stones and creating futures intervention: outcomes of a formative evaluation of behavioural and structural pilot intervention for young people.

[39] Tang KC, Davis A (1995). Critical factors in the determination of focus group size. Fam Pract.

[40] Attride-Stirling J (2001). Thematic networks—an analytical tool for qualitative research. Qual Res.

[41] Braun V, Clarke V (2006). Using thematic analysis in psychology. Qual Res Psychol.

[42] Morrell R, Jewkes R, Lindegger G, Hamlall V (2013). Hegemonic masculinity: reviewing the gendered analysis of men's power in South Africa. South Afr Rev Sociol.

[43] Seedat M, Van Niekerk A, Jewkes R, Suffla S, Ratele K (2009). Violence and injuries in South Africa: prioritising an agenda for prevention. (vol 374, pg 1011, 2009). Lancet.

[44] Vandello J, Bosson JK (2013). Hard won and easily lost: a review and synthesis of theory and research on precarious masculinity. Psychol Men Masc.

[45] Butler J (1999). Gender trouble: feminism and the subervision of identity.

[46] Gibbs A, Willan S, Misselhorn A, Mangoma J (2012). Combined structural interventions for gender equality and livelihood security: a critical review of the evidence from southern and eastern Africa and the implications for young people. J Int AIDS Soc.

# Sub article: Gender inequalities in health: exploring the contribution of living conditions in the intersection of social class

**DOI:** 10.3402/gha.v7.23189

**Published:** 2014-02-14

**Authors:** Davide Malmusi, Alejandra Vives, Joan Benach, Carme Borrell

**Affiliations:** 1 Centre for Biomedical Network Research on Epidemiology and Public Health (CIBERESP), Spain; 2 Agència de Salut Pública de Barcelona, IIB-Sant Pau, Barcelona, Spain; 3 Unitat Docent de Medicina Preventiva i Salut Pública PSMAR-UPF-ASPB, Barcelona, Spain; 4 Health Inequalities Research Group – Employment Conditions Network (GREDS-EMCONET), Universitat Pompeu Fabra, Barcelona, Spain; 5 Departamento de Salud Pública, Escuela de Medicina, Pontificia Universidad Católica de Chile, Santiago, Chile; 6 Department of Experimental and Health Sciences, Universitat Pompeu Fabra, Barcelona, Spain

**Keywords:** gender, health inequalities, self-rated health, intersectionality, material resources, social class

## Abstract

**Background:**

Women experience poorer health than men despite their longer life expectancy, due to a higher prevalence of non-fatal chronic illnesses. This paper aims to explore whether the unequal gender distribution of roles and resources can account for inequalities in general self-rated health (SRH) by gender, across social classes, in a Southern European population.

**Methods:**

Cross-sectional study of residents in Catalonia aged 25–64, using data from the 2006 population living conditions survey (n=5,817). Poisson regression models were used to calculate the fair/poor SRH prevalence ratio (PR) by gender and to estimate the contribution of variables assessing several dimensions of living conditions as the reduction in the PR after their inclusion in the model. Analyses were stratified by social class (non-manual and manual).

**Results:**

SRH was poorer for women among both non-manual (PR 1.39, 95% CI 1.09–1.76) and manual social classes (PR 1.36, 95% CI 1.20–1.56). Adjustment for individual income alone eliminated the association between sex and SRH, especially among manual classes (PR 1.01, 95% CI 0.85–1.19; among non-manual 1.19, 0.92–1.54). The association was also reduced when adjusting by employment conditions among manual classes, and household material and economic situation, time in household chores and residential environment among non-manual classes.

**Discussion:**

Gender inequalities in individual income appear to contribute largely to women's poorer health. Individual income may indicate the availability of economic resources, but also the history of access to the labour market and potentially the degree of independence and power within the household. Policies to facilitate women's labour market participation, to close the gender pay gap, or to raise non-contributory pensions may be helpful to improve women's health.

Sex differences in health are usually described as a paradox, where women have longer life expectancy than men but poorer health status, in terms of indicators such as mental health, chronic illness, disability, or self-rated general health (1, 2). It has been shown that women are not more prone to report illness (3), and that, once adjusted for other measures of morbidity, gender differences in self-rated health (SRH) are eliminated (4). Men's shorter life expectancy can be attributed to both biological differences in disease susceptibility and gendered patterns of health-related behaviours and risk taking (5). Here we focus on women's poorer health status, which has been interpreted as unfair and avoidable gender inequalities resulting mainly from patriarchy, the systematic domination of women by men (6).

There is a growing recognition of the existence of large, avoidable, and unacceptable social inequalities in health among population groups, and of the need to understand and tackle their causes and mechanisms (7). Following the conceptual framework of the World Health Organization Commission on the Social Determinants of Health (WHO CSDH), in the pathway between the individual's position in the social structure according to gender, social class and ethnicity, among others, and his or her health, lie the ‘intermediary determinants’: factors that influence health outcomes and that are unequally distributed (8). A large body of scientific literature has focused on the study of intermediary determinants of health inequalities by socio-economic position and/or social class. Among these intermediary determinants, material factors and resources such as material standards of living, financial difficulties or insecurity, economic resources, neighbourhood characteristics, employment status, or physical and organisational working conditions have been shown to largely explain inequalities in health status (9–12) and mortality (13).

Relatively fewer studies have studied the intermediary determinants of gender inequalities in health. A group of studies in North America (14–17) and a nationwide Indian survey (18) coincided in partially or totally explaining women's poorer health (in indicators such as SRH, disability, chronic illness, or health-related quality of life) with inequalities in economic resources and social roles. Less studies of this nature have been conducted in Europe until 2012, when in a pooled multinational analysis of the World Health Survey with relatively few explanatory variables, employment status was the single most powerful explanatory factor with a contribution of 20% (19). In a study in central Sweden, adjustment by financial difficulties and condescending treatment rendered gender differences in SRH non-significant (20). Comparative studies across Europe have situated Southern countries as having some of the largest gender inequalities in SRH (21) and depression (22). Spain has experienced a rapid change after Franco's dictatorship, during which patriarchy and its stereotypes were reinforced by the regime (23) and was recently ranked No. 13 in United Nations’ Gender Empowerment Measure global index of social gender equity (24). However, deep gender inequalities persist in aspects such as labour market participation or the share of domestic work (25).

The intersectional analysis of the different mechanisms of power relations in society such as social class and gender has emerged as a priority for future health equity research (26, 27). The interaction of gender and social class (28) and the differential suitability of socio-economic indicators in men and women (29) have long attracted the interest of researchers, and several studies on the intermediary determinants of social class inequality stratify their analyses by sex (9–11). However, we are not aware of studies exploring how the contribution of intermediary determinants to gender inequalities in health differs across different social classes.

Given this background, the present study aims to explore the contribution of intermediary determinants of inequalities in SRH by gender, across social classes, in a Southern European population.

## Present investigation

### Study population, sample, and data collection

The study population was the 2006 non-institutionalised population of Catalonia, Spain (around 7,000,000 inhabitants). Estimations for Spanish regions of the Gender Development Index situated Catalonia slightly above the country average. We used data from a cross-sectional survey: the 2006 ‘Enquesta de Condicions de Vida i Hàbits de la Població’ (Population Living Conditions and Habits Survey) (30). While this socio-economic survey lacks information on so-called psychosocial and behavioural risk factors, it allows for a deep exploration of material conditions. The sample was stratified by territory; in each territory, random census tracts were selected, and within each, individuals were selected randomly from the population census (response rate for contacted subjects, 72.7%; non-responders were replaced by subjects of the same census tract, age and sex); weights were provided to ensure representativeness of the sample for the population of Catalonia (for instance, to revert the oversampling of less populated territories) (30). Information was collected during face-to-face interviews at home: a total of 10,397 were completed.

For this study, the sample was restricted to subjects aged 25–64 (n=7,179) in order to include the population in working age, that has largely completed its studies and achieved its own occupational social class. To prevent reverse causality to SRH for income, employment status, and household chores variables, we excluded subjects declaring inability to work (n=283), having left their last job for health reasons (n=113) or with a dependency (difficulty to move within the house, get dressed, wash themselves, or eat on their own; n=81). Subjects without coded social class (n=18) were also excluded, resulting in a total sample of 6,683 subjects. Further sensitivity analyses were also conducted restricting to employed subjects that were married or cohabiting (n=3,857).

### Indicators and variables

The dependent variable was SRH, measured with a single question: ‘Would you say your overall health is …?’ with a 5-point Likert-type answer scale, ranging from ‘very good’ to ‘very poor’. Answers were dichotomised into fair/poor (fair, poor or very poor) and good (good or very good) health.

The independent variable, gender, was approached through the sex of the respondent (man or woman).

Social class, used as a stratification variable, was based on the current or last occupation of the subject or, for the never employed subjects (0.2% of men and 5.4% of women in our sample), the occupation of the partner or household reference person. The Spanish adaptation of the British Registrar General classification (31) was used to create two broad social class groups: ‘non-manual’ including class I, II, and III-non-manual; and ‘manual’ including III-manual (self-employed and supervisors in manual occupations), IV and V.

As for the intermediary material determinants, following the categories detailed by the Spanish Commission to Reduce Social Inequalities in Health in its adaption and expansion of the WHO CSDH conceptual framework (8), the following variables were analysed:

1. Employment conditions: a variable was created combining employment status (with the following categories: employed; unemployed but seeking job; dedicated to housework; student; early retired) and type of contract (with those employed additionally divided into: employer or self-employed; wage worker with permanent; temporary; or no contract).
2. Individual income: the respondent was shown a card with several monthly income ranges and was asked to indicate the range of his or her own income.
3. Household economic and material resources:
  - Household financial difficulties. An index was created by adding four items: difficulty in making it through the month, savings capacity, economic difficulties during the past 5 years, and need to reduce household expenditure during the last 5 years (Cronbach's alpha 0.72; factor analysis confirmed that all items loaded positively onto one factor). Scores range from 0 (minimal difficulties) to 4 (maximum difficulties).
  - Household material assets. An index was created by adding 10 items: dishwasher, vacuum cleaner, dryer, personal computer, internet connection, DVD player, video camera, stereo, holidays during last year (away from home for at least four consecutive days), and monthly spending for leisure over 50 euros (Cronbach's alpha 0.72; factor analysis confirmed that all items loaded positively onto one factor). Scores range from 0 (no assets) to 10 (all assets).
4. Residential environment: respondents were asked to rate, on a scale of 0–10, their:
  - Neighbourhood quality of life
  - Perception of safety problems in the neighbourhood.
5. Household tasks: average daily hours dedicated to household tasks, calculated based on information for a regular weekday and weekend.

### Analytical strategy

All analyses were carried out using the Stata 10 statistical package and included sampling weights (30). First, we described the distribution of study variables (age, SRH, and intermediary determinants) across the four subgroups derived from combining gender and social class. Second, within each of the four subgroups, robust Poisson regression models were adjusted to estimate the association between each intermediary determinant and SRH as age-adjusted prevalence ratios (PRs).

Then, to estimate the contribution of intermediary factors, we first calculated age-adjusted PRs of fair/poor SRH by gender (within each of the two social class groups). Each intermediary factor was added separately into this baseline model: its individual contribution was estimated as the percentage change in the regression coefficient of gender between the baseline (PR_model 1_) and adjusted model (PR_model 2_), using the formula (PR_model 1_ − PR_model 2_)/(PR_model 1_ − 1). Finally, the preceding analysis was repeated but factors were added sequentially to the model, following a semi-causal sequence (see Fig. 1).

**Fig. 1:** Prevalence ratio (PR) and 95% confidence intervals of fair/poor self-rated health (SRH), women versus men, in different social classes, adding to the model groups of intermediary determinants. Population aged 25–64 residing in Catalonia.

## Results

Table 1 shows the distribution of the study variables in the four subgroups derived from combining gender and social class. Non-manual women were the youngest group (mean age 40.3) and manual women the oldest (43.3). Women had worse SRH than men in both social classes. More women than men had a low or no individual income, were dedicated to housework, and spent more than three hours on household tasks each day: all of these differences were more marked in manual social classes. Among those in employment, women were more likely than men to have no contract. There were small gender differences also in household financial difficulties, material assets and in the reporting of safety problems.

**Table 1:** Description of the study variables in subgroups of gender and social class (population aged 25–64 residing in Catalonia)

Gender		Men		Women	
		
Social class		Non-manual	Manual	Non-manual	Manual
*N (weighted)*		*1,361*	*2,044*	*1,483*	*1,795*
Mean age		41.9	41.8	40.3	43.3
Fair/poor self-rated health	%	9.9	17.9	12.9	25.8
Employment conditions					
Self-employed or employer	%	24.7	19.6	14.2	11.1
Employed. Permanent contract		58.5	53.0	56.1	31.9
Employed. Temporary contract		6.5	14.8	10.1	12.3
Employed. No contract		0.0	0.4	0.4	4.3
Unemployed		3.9	6.2	4.6	7.5
Housework		0.1	0.0	12.7	30.1
Retired		5.4	5.6	1.2	2.2
Student		0.9	0.4	0.7	0.6
Individual monthly income					
None	%	1.2	1.1	12.4	25.5
450€ or less		2.6	4.0	7.2	22.1
451–900€		6.2	17.8	14.8	26.2
901–1,500€		34.9	51.0	35.7	14.2
>1,500€		43.5	14.2	20.3	1.3
Not declared		11.6	11.9	9.6	10.7
Household economic resources					
Financial difficulties	Mean no. (0–4)	1.1	1.8	1.3	2.0
	%>2	16.7	35.6	20.9	39.2
Material assets	Mean no. (0–10)	7.6	5.6	7.4	5.3
	%<5	7.9	32.4	8.3	38.6
Residential environment					
Quality of life	Mean rating (0–10)	7.0	6.8	7.0	6.9
	%<6	13.9	22.0	15.6	20.9
Safety problems	Mean rating (0–10)	4.4	4.4	4.7	4.8
0–5	%	64.5	64.1	59.7	56.9
6–9		31.5	28.3	32.7	34.0
10		4.0	7.6	7.6	9.1
Household tasks					
None	%	9.7	14.7	1.7	0.7
Up to 3 hr/day		82.6	78.4	66.5	45.8
More than 3 hr/day		4.5	4.4	28.8	50.2
Not declared		3.2	2.6	3.0	3.3

The associations between intermediary variables and SRH in each of the four subgroups are shown in Table 2. The association between employment conditions and SRH only reached statistical significance for unemployment among non-manual men and women. Individual income generally showed a graded association with SRH. Financial difficulties, material assets, neighbourhood quality of life and a very high rating of safety problems were consistently associated with SRH in all subgroups (not significantly in non-manual men). Manual men making no household tasks and non-manual women dedicating more than 3 hours a day to these tasks had a significantly increased risk of fair/poor SRH.

**Table 2:** Age-adjusted prevalence ratios (PRs) of fair/poor self-rated health (SRH) according to intermediary determinants in subgroups of gender and social class. Population aged 25–64 residing in Catalonia

Gender		Men		Women	
		
Social class		Non-manual	Manual	Non-manual	Manual
*N (weighted)*		*1,361*	*2,044*	*1,483*	*1,795*
Employment conditions					
Self-employed or employer		1.34	0.80	1.03	1.12
Employed. Permanent contract (ref)		1	1	1	1
Employed. Temporary contract		0.51	1.10	0.78	0.97
Employed. No contract		n/a	n/a	n/a	1.03
Unemployed		2.25*	1.20	1.76*	1.05
Housework		n/a	n/a	1.31	1.06
Retired		1.20	0.93	1.33	0.84
Student		0***	n/a	1.94	n/a
Individual monthly income					
None		2.44	0.52	1.05	1.69**
450€ or less		2.71**	2.11***	1.26	1.75**
451–900€		0.61	1.71***	1.07	1.59*
901–1,500€ (ref)		1	1	1	1
>1,500€		0.81	0.77	0.52*	0.98
Not declared		1.03	1.00	0.91	1.27
Household economic resources					
Financial difficulties	0–2 (ref)	1	1	1	1
	3–4	1.49	1.56***	2.14***	1.54***
Material assets	5–10 (ref)	1	1	1	1
	0–4	1.53	1.62***	1.67**	1.54***
Residential environment					
Quality of life	6–10 (ref)	1	1	1	1
	0–5	1.53	1.62***	1.67**	1.54***
Safety problems	0–5 (ref)	1	1	1	1
	6–9	0.70	1.04	1.24	1.33**
	10	1.80	1.50*	2.50***	1.40*
Household tasks					
None		1.57	1.41**	1.65	0.51
Up to 3 hr/day	(ref)	1	1	1	1
More than 3 hr/day		1.36	0.77	1.48*	1.10
Not declared		0.09*	1.33	0.75	0.96

* p<0.05

** p<0.01

*** p<0.001.

(ref)=Reference category; n/a (not applicable) indicates that less than 10 subjects belonged to the selected category in that subgroup: PR was omitted for statistical instability.

Table 3 shows PRs of fair/poor SRH for women compared to men within subgroups of social class, and the estimated percent contribution of each intermediary determinant. When adjusting by employment conditions, reductions of the association are small (7% among non-manual, 13% among manual). Adjustment by individual income eliminated the association among manual classes, and halved and rendered non-significant the association among non-manuals. Household financial and material resources reduced the association by 10–15%. Adjustment by neighbourhood quality variables and household tasks reduced gender inequalities among those in non-manual (16 and 21%, respectively) but not among those in manual social class.

**Table 3:** Prevalence ratio (PR) of fair/poor self-rated health (SRH), women versus men, in different social classes, adjusting separately by groups of intermediary determinants. Population aged 25–64 residing in Catalonia

Sub-group	Non-manual		Manual	
	
Adjustment variables	PR	% change	PR	% change
Baseline (age-adjusted)	1.39**		1.36***	
Employment conditions^a^	1.36*	7	1.32**	13
Individual income^a^	1.19	50	1.01	98
Household economic resources^b^	1.31*	20	1.30***	17
Financial difficulties^c^	1.33*	15	1.33***	10
Material deprivation^c^	1.35*	10	1.32***	13
Residential environment^b^	1.32*	16	1.37***	0
Quality of life^a^	1.37**	4	1.38***	−4
Safety problems^a^	1.33*	15	1.35***	4
Household tasks^a^	1.31	21	1.39***	−7
All determinants^d^	1.18	53	1.05	86

* p<0.05

** p<0.01

*** p<0.00.

a Same categories as in Table 2.

b All variables of the group are included in the model.

c Linear index.

d All groups reducing the association on their own.

Figure 1 shows the effect of sequentially adding determinants to the baseline models of inequality in SRH. The pattern by gender is slightly different between non-manual classes, where individual income, neighbourhood quality and household tasks all bring small reductions, as compared to manual, where there is a small reduction by adding employment, a complete reduction when adding income, and a new onset of associations (not significant) when adding the rest of variables.

In Supplementary file, the analyses of Table 3 are replicated in a subsample of employed subjects that were married or cohabiting. Percent reductions of associations were similar in manual classes but substantially lower in non-manual classes (individual income 16%, all determinants 22%), yet gender lost significance after adjustments.

## Discussion and conclusion

To our knowledge, this is the first study that explores the contribution of intermediary determinants to gender inequalities in SRH across different social classes. The main finding of our study is that these inequalities were fully accounted for by gender inequalities in material resources, with a striking contribution of individual income, especially among manual classes.

Early North American studies had mainly focused on social roles as an explanation of gender inequalities in health (14, 15). More recent studies have found that household income largely explained gender inequalities in physical and mental health in the United States (16), that adjustment by income and income source halved the excess risk of poor SRH of US women (17), and that material assets and economic independence entirely explained gender inequalities in SRH in an Indian study (18). While we did not directly test the contribution of household income, we found that other measures of household resources such as financial difficulties were contributing much less than individual income to gender inequalities. Moreover, the explanatory power of individual income was larger in the whole sample than in the sub-analysis restricted to employed and cohabiting subjects. We hypothesise that individual income may be accounting for inequities suffered by women better than the household's economic situation because it is not only indicating availability of economic resources, but also the personal history of access to the labour market and the degree of women's autonomy and existing bargaining power within the household.

The analysis of intersections between gender and social class allowed for the detection of peculiarities in the pathways of producing these inequalities. For example, individual income totally explained gender inequalities in manual classes, among which most of women were unemployed or had a very low income; in contrast, among non-manual classes, and especially in the sub-analysis of employed subjects, a wider set of factors made independent contributions to the observed gender inequalities, and some degree of inequality still persisted (although non-significant) after all adjustments. This suggests that even when some key barriers are overcome, such as that of access to professional positions in the labour market, more subtle aspects of power, gender norms, discrimination, ‘glass ceiling’, and care responsibilities still may produce an unequal burden on women's health. Actually, it has been reported in Spain that perceived sexism is higher among employed than unemployed women, especially in managerial positions (32). Psychosocial factors, such as discrimination or condescending treatment, which have shown relevant contributions in previous investigations (20), were not collected in this survey and could warrant further exploration in future studies.

Other factors investigated in this study also played some role as a pathway to gender health inequality. Burden of housework and care has rarely been investigated as a determinant of health inequalities despite its salience to the health of women, especially in the working class (33). In the present study, however, only housework weekly hours could be measured; and the relationship between these and SRH is likely to be affected by reverse causality (poor health as a cause of less time spent in housework). Nevertheless, their contribution to health inequalities by gender was not negligible among non-manual class. Self-rated neighbourhood quality seemed to play a little but probably not independent role. While men and women are generally exposed to the same residential environment, it seems that women can be more vulnerable to some of their effects, as indicated by the higher prevalence of reported safety problems. Employment status and conditions seemed only partially relevant, as their association with SRH was marginal. Longitudinal data could probably make more evident the contribution of conditions such as unemployment and precarious contract arrangements, that are themselves major lifelong determinants of income or material resources.

The use of cross-sectional data is a bidirectional threat to the validity of estimations of the association between some material factors and health, and consequently to their contribution to health inequalities: on the one hand, for example, regarding income or employment conditions, the lack of information about lifetime exposures can lead to an underestimation of the association, whereas reverse causality, on the other hand, can lead to its overestimation. To limit the latter, several groups of subjects were excluded from analyses: those declaring inability to work, a health issue as the reason for leaving the last job or difficulties in activities of daily living.

The calculation of the percent change in the strength of association between models with and without one or more intermediary variables was our method of choice for estimating the contribution of determinants, following the majority of the aforementioned literature (9–12, 19). As in all of these studies, we did not present confidence intervals for these percentages, despite the existence of methods for calculating the standard error of the indirect effect, such as the Sobel's test or bootstrapping methods (34) that are of limited application with categorical variables and non-linear models. While point estimates of ‘explained fraction’ should be interpreted with caution, this technique is useful to approximate the relative importance of one factor over another.

One of this study's strengths lies in the inclusion of all working age respondents, and not only the employed, thus avoiding the exclusion of an important proportion of the population and especially women. Actually, the role of individual income is more evident when analysing data from the whole population than in the sensitivity analysis restricted to the working population living with a partner. However, it must be considered that this study was carried out with data from Catalonia, a region in the northeast of Spain, in 2006: the impact of intermediary determinants of inequalities may differ in different countries and welfare regimes (35), and at different points in time (for example, with changing economic contexts such as the current crisis affecting Southern Europe).

In conclusion, material resources, and especially individual income, account for observed health inequalities by gender, with slightly different pathways across social classes. The WHO CSDH recommendation for policymakers of tackling ‘the inequitable distribution of power, money and resources’ (7), is as relevant for gender as for other dimensions of social inequity. Policies to facilitate women's labour market participation, to close the gender pay gap, or to raise non-contributory pensions may help improving women's living standards and independence. Practitioners should take into account the social aetiology behind many women's illnesses which they are confronted with in their daily practice, coordinate with other community services to improve women's social and family context and facilitate their access to decent jobs, and advocate for local and national policies that foster gender equity.

## References

[1] Case A, Paxson C (2005). Sex differences in morbidity and mortality. Demography.

[2] Verbrugge LM (1985). Gender and health: an update on hypotheses and evidence. J Health Soc Behav.

[3] Gijsbers van Wijk CM, Huisman H, Kolk AM (1999). Gender differences in physical symptoms and illness behavior. A health diary study. Soc Sci Med.

[4] Malmusi D, Borrell C, Artazcoz L, Benach J (2012). Perception or real illness? How chronic conditions contribute to gender inequalities in self-rated health. Eur J Pub Health.

[5] Bird CE, Rieker PP (1999). Gender matters: an integrated model for understanding men's and women's health. Soc Sci Med.

[6] Doyal L What makes women sick: gender and the political economy of health.

[7] CSDH (2008). Closing the gap in a generation: health equity through action on the social determinants of health. Final Report of the Commission on Social Determinants of Health.

[8] (2012). Comision para reducir las desigualdades sociales en salud en España. [Proposal of policies and interventions to reduce social inequalities in health in Spain]. Gac Sanit.

[9] Aldabe B, Anderson R, Lyly-Yrjänäinen M, Parent-Thirion A, Vermeylen G, Kelleher CC (2011). Contribution of material, occupational, and psychosocial factors in the explanation of social inequalities in health in 28 countries in Europe. J Epidemiol Community Health.

[10] Borrell C, Muntaner C, Benach J, Artazcoz L (2004). Social class and self-reported health status among men and women: what is the role of work organisation, household material standards and household labour?. Soc Sci Med.

[11] Kaikkonen R, Rahkonen O, Lallukka T, Lahelma E (2009). Physical and psychosocial working conditions as explanations for occupational class inequalities in self-rated health. Eur J Public Health.

[12] Popham F, Bambra C (2010). Evidence from the 2001 English Census on the contribution of employment status to the social gradient in self-rated health. J Epidemiol Community Health.

[13] van Oort FV, van Lenthe FJ, Mackenbach JP (2005). Material, psychosocial, and behavioural factors in the explanation of educational inequalities in mortality in The Netherlands. J Epidemiol Community Health.

[14] Ross CE, Bird CE (1994). Sex stratification and health lifestyle: consequences for men's and women's perceived health. J Health Soc Behav.

[15] Denton M, Prus S, Walters V (2004). Gender differences in health: a Canadian study of the psychosocial, structural and behavioural determinants of health. Soc Sci Med.

[16] Cherepanov D, Palta M, Fryback DG, Robert SA (2010). Gender differences in health-related quality-of-life are partly explained by sociodemographic and socioeconomic variation between adult men and women in the US: evidence from four US nationally representative data sets. Qual Life Res.

[17] Prus SG (2011). Comparing social determinants of self-rated health across the United States and Canada. Soc Sci Med.

[18] Roy K, Chaudhuri A (2008). Influence of socioeconomic status, wealth and financial empowerment on gender differences in health and healthcare utilization in later life: evidence from India. Soc Sci Med.

[19] Hosseinpoor AR, Stewart Williams J, Amin A, Araujo de Carvalho I, Beard J, Boerma T (2012). Social determinants of self-reported health in women and men: understanding the role of gender in population health. PLoS One.

[20] Molarius A, Granstrom F, Feldman I, Kalander Blomqvist M, Pettersson H, Elo S (2012). Can financial insecurity and condescending treatment explain the higher prevalence of poor self-rated health in women than in men? A population-based cross-sectional study in Sweden. Int J Equity Health.

[21] Bambra C, Pope D, Swami V, Stanistreet D, Roskam A, Kunst A (2009). Gender, health inequalities and welfare state regimes: a cross-national study of 13 European countries. J Epidemiol Community Health.

[22] Van de Velde S, Bracke P, Levecque K, Meuleman B (2012). Gender differences in depression in 25 European countries after eliminating measurement bias in the CES-D 8. Soc Sci Res.

[23] Falcón O'Neill L (2003). Memorias políticas (1959–1999).

[24] UNDP (2007). Fighting climate change: human solidarity in a divided world.

[25] Voicu M, Strapcová K, Voicu B (2008). Housework's division in 24 European societies: a cross-national comparison. Calitatea Vietii.

[26] Kelly MP (2010). The axes of social differentiation and the evidence base on health equity. J R Soc Med.

[27] Östlin P, Schrecker T, Sadana R, Bonnefoy J, Gilson L, Hertzman C (2011). Priorities for research on equity and health: towards an equity-focused health research agenda. PLoS Med.

[28] Macintyre S, Hunt K (1997). Socio-economic position, gender and health: how do they interact?. J Health Psychol.

[29] Krieger N, Chen JT, Selby JV (1999). Comparing individual-based and household-based measures of social class to assess class inequalities in women's health: a methodological study of 684 US women. J Epidemiol Community Health.

[30] Institut d'Estadística de Catalunya (2007). Enquesta de condicions de vida i habits de la població 2006.

[31] 
Chilet-Rosell E, Alvarez-Dardet C, Domingo-Salvany A (2012). [Use of Spanish proposals for measuring social class in health sciences]. Gac Sanit.

[32] Borrell C, Artazcoz L, Gil-Gonzalez D, Pérez K, Pérez G, Vives-Cases C (2011). Determinants of perceived sexism and their role on the association of sexism with mental health. Women Health.

[33] Artazcoz L, Borrell C, Benach J (2001). Gender inequalities in health among workers: the relation with family demands. J Epidemiol Community Health.

[34] Miura K, Giskes K, Turrell G (2011). Contribution of take-out food consumption to socioeconomic differences in fruit and vegetable intake: a mediation analysis. J Am Diet Assoc.

[35] Kim I, Muntaner C, Vahid Shahidi F, Vives A, Vanroelen C, Benach J (2012). Welfare states, flexible employment, and health: a critical review. Health Policy.

# Sub article: Between desire and rape – narratives about being intimate partners and becoming pregnant in a violent relationship

**DOI:** 10.3402/gha.v6i0.20984

**Published:** 2013-12-05

**Authors:** Kerstin Edin, Bo Nilsson

**Affiliations:** 1 Epidemiology and Global Health, Umeå University, Umeå, Sweden; 2 Department of Nursing, Umeå University, Umeå, Sweden; 3 Umeå Centre of Gender Studies, Umeå University, Umeå, Sweden; 4 Culture and Media Studies, Umeå University, Umeå, Sweden

**Keywords:** spouse abuse, sexuality, pregnancy intention, gender identity, narratives, Sweden

## Abstract

**Background:**

Women subjected to intimate partner violence (IPV) experience different forms of abuse. Sexual violence is often under-reported because physically abused women, in particular, might see forced sex as an obligatory part of the sexual interplay. Accordingly, abused women have less sexual autonomy and experience unplanned pregnancies more often than other women.

**Objective:**

To describe and analyse nine Swedish women's retrospective stories about IPV with a focus on power and coping strategies as intimate partners, particularly regarding experiences of sex, contraception, and becoming pregnant.

**Design:**

Nine qualitative interviews were carried out with women who had been subjected to very severe violence in their intimate relationships and during at least one pregnancy. The stories were analysed using ‘Narrative method’ with the emphasis on the women's lived experiences.

**Results:**

Despite the violence and many contradictory and ambivalent feelings, two of the women described having sex as desirable, reciprocal and as a respite from the rest of the relationship. The other seven women gave a negative and totally different picture, and they viewed sex either as obligatory or as a necessity to prevent or soothe aggression or referred to it as rape and as something that was physically forced upon them. The women's descriptions of their pregnancies ranged from being carefully planned and mostly wanted to completely unwelcome and including flawed contraceptive efforts with subsequent abortions.

**Conclusions:**

Women subjected to IPV have diverse and complex experiences that have effects on all parts of the relationship. Intimacy might for some turn into force and rape, but for others sex does not necessarily exclude pleasure and desire and can be a haven of rest from an otherwise violent relationship. Accordingly, women may tell stories that differ from the ones expected as ‘the typical abuse story’, and this complexity needs to be recognized and dealt with when women seek healthcare, especially concerning contraceptives, abortions, and pregnancies.

In an international perspective, from one-third to as many as half of all women who were specifically asked say that they have been subjected to sexual violence (1, 2). Women who report sexual abuse most often accuse someone they know, especially their intimate partner (3, 4). The impact of sexual violence is most likely more traumatic for an intimate partner compared to a non-partner (5). However, women often fail to label themselves as being raped in an intimate relationship and might even see forced sex as an obligatory part of the relationship (6). Consequently, for many different reasons, sexual violence is highly under-reported to police and healthcare officials (3).

Women subjected to violence have unplanned pregnancies more often than other women (7, 8). This is a result of forced sex that often occurs in combination with an inability to practice birth control (9–11). For unintended pregnancies ending in a live birth, the risks are significantly increased for having a pre-term delivery and of having a baby with low birth weight (12). Thus, intimate partner violence (IPV) might have synergistic effects that lead to increased risk for adverse pregnancy outcomes (10).

Instead of attempting to overcome the barriers to seeking help, abused women in a relationship with ongoing violence often choose to endure their situation because they fear the unknown changes and subsequent consequences of seeking help (13). Accordingly, when abused women seek healthcare for diverse reasons, they often do not disclose the violence of their own accord, but still they generally appreciate and support the idea of being asked (14, 15). However, even if women honestly respond to direct questions, many do not trust authorities to interfere and, therefore, wish for no further support (16).

In order to deal with women who are subjected to IPV, there is much that is needed to be understood about their situation because abused women live in complex and often very bonding relationships (17). The caregivers’ behaviour and their methods for building trust are, therefore, of vital importance if their relationship with their patient is to have a successful outcome (18–21). The barriers are two-sided and while the women keep their distance afraid of what will happen if they disclose their situation, healthcare personnel might not manage to get close to their patient because of the sensitivity of the topic (21, 22), especially if the woman is pregnant.

This study is about women in Sweden who became pregnant in a violent relationship. Gender equality has been an important political issue in Sweden, but despite the attention paid to this issue, the prevalence of violence during pregnancy is in line with what has been reported from the United States, Australia, and Canada. Furthermore, abuse can occur regardless of the woman's socio-economic status, and women who are exposed to severe violence before pregnancy usually experience violence during pregnancy as well. This perhaps indicates the importance of screening for abuse in Sweden (23). However, the term *IPV screening* is problematic (21, 24), and healthcare providers using streamlined screening questions together with their simplified understanding of IPV might not identify components such as women being forced into pregnancy (8). For several years, there has been an international perspective that requests a deepened understanding and more comprehensive view of the complex life situations of abused women (25, 26).

The overall aim of this paper is to describe and analyse nine Swedish women's narratives about their violent relationships and about having sex and becoming pregnant. The primary focus of the paper is on power and coping strategies, especially the complexities and contradictions within and between the stories.

## Methods

### Qualitative methods

We carried out interviews that aimed to acquire the broadest and deepest understanding of women's experience with IPV, and we used narrative methods for the analysis. The most weight was put on understanding the meaning of the women's lived experiences, including their own reflections and considerations, and less weight was placed on how the stories were told linguistically (27). The interviews were treated as dialogues that supported diverse beliefs and assumptions in order to create shared constructions of meaning between the interviewer and the informants (28, 29). Such constructed knowledge is never constant but is always changing as a result of interpersonal, social, cultural, and political influences. This kind of knowledge is also often incomplete because the person providing the information has the power to decide what information is included or excluded (30). Accordingly, the narratives in this paper represent the stories that the women chose to give at that particular time and place (31, 32).

### Subjects, interviews, and analyses

We contacted the coordinators of shelters and a women's crisis centre in regard to this study and asked for their help in identifying women who earlier had received support and counselling from them and who matched the specific criterion for inclusion. This criterion was having been subjected to violence by an intimate partner in a relationship in which they had become pregnant. Ten women were invited to be interviewed, but one subsequently decided not to participate and dropped out of the study. The nine remaining women were interviewed by the first author, three in 2001 and six in 2003, and each interview lasted for about two hours. An interview guide with open-ended questions was used, and some questions could be omitted or expanded upon and the exact wording and sequence could differ (28, 33). The interviews resulted in a large amount of data, and the selections presented here were chosen based on their applicability to the main focus of the paper (see also 21).

The women's narratives about the violence were in general very detailed, lively, and dramatic. The interviewer was not only a partner in the dialogue, but often found herself attending a performance that through the details, laughter, tears, different voices, metaphors, and quotes transported us both back to the place and time where the events had taken place. Nevertheless, repressive mechanisms were apparently used by some women. They were often hesitant or had difficulties in recounting the memories and provided only partial and patchy recollections when it came to some especially tough details.

The interviewer, a mother with a background as an RN midwife, appeared to build a trusting relationship that enabled the women to include sensitive issues while adding statements like *as you know*. One woman cried a lot during the interview, but felt much relieved to have told certain parts of her story for the very first time, even if the subject matter was extremely sensitive. All of the women gave the impression that they found it of great value to share their stories. To assure support and someone to talk to, all of them received the interviewer's contact information after the interviews. Moreover, all of the women, because of ongoing or previous contacts with the shelters/centres, had a familiar helpline if needed.

The interviewer took notes and performed preliminary analyses for each interview, and the recordings were transcribed verbatim and coded into codes and categories to interpret the probable meaning as a whole and to identify crucial tracks to provide an analytical framework with narrative themes in which to present the stories (34, 28). We provide one example of the coding process in Table 1. Both of the authors took part in processing the entire interview material, and the initial analysis made by the first author (KEE) was discussed and interpreted by both authors together. The narrative themes could not be presented as just one coherent and typical story because all of the stories contained differences, contrasts, and contradictions (29). Instead, the different voices are either intertwined into representative narratives or written as individual stories (28). Direct quotes from the women are written in italics with the purpose of emphasizing or providing examples of selected narrative themes.

**Table 1:** Examples of selected codes and categories building up a narrative theme

Selected codes	Categories	Narrative theme
Adverse feelings, forced, all the time, spoiled her IUD contraception, sexual violence, abortions, never wanted a child, not accepting it to happen again, wanted to leave, he needed to find another woman	Forced, did not want to, never, wanted to escape from him, had contraception	*I did not want to become a parent*

### Ethics

The research process aimed to maintain ethical considerations regarding confidentiality, safety, and support by following WHO recommendations (35). The study was approved by the local Ethics Committee of the Faculty of Medicine, Umeå University.

## Findings

The nine women were approximately between 31 and 55 years old at the time of the interview. They had varied educational backgrounds and socio-economic status and different job positions; two were on sick leave. The women had a total of 25 children from 20 long-term non-violent and violent relationships, and five women had been married to their violent partner. They told stories about how they had been subjected to different forms of brutal physical and/or sexual violence and/or threats of severe violence during a total of 14 pregnancies. In five of the cases, the pregnancy had started or restarted the violence, and for the others the pregnancy occurred during ongoing violence that either became more aggressive or maintained its current level. Generally, the women expressed feelings of having been unable to escape from a situation that was chaotic and very, very strange. Two women left their violent partners while being pregnant because of life-threatening violence, and the others left when their last child was still a toddler. Even if some of the women had tried to recommence their relationships, all of them appeared to have left their partners permanently (from about 8 months up to 16 years ago) at the time of the interview (*cf*. 21).

The following presentation deals with the women's narrated experiences of their intimate relationships and is divided into the following three sections: the relationship, having sex, and becoming pregnant and includes eight narrative themes: ‘Like in a prison’; ‘As if they were worthless’; ‘To always be on one's guard’; ‘Sex as violence and as a part of the violent relationship’; ‘Sex as the only thing that actually worked’; ‘I did not want to become a parent’; ‘So I became pregnant’; and ‘A wanted child’.

### The relationship

The interviewed women portrayed their partners as tormentors and called them a *devil* or described them as *Jekyll and Hyde* as they changed from good to bad. This *Jekyll* was present in the narratives as the charming and nice man that they had fallen in love with. Some nurtured the hope of change and expressed wishful thinking that the relationship could return to how it was when they had first met.^1^

#### Like in a prison

The lives of these women became very restricted, and they were isolated from their former friends. Often the women did not even understand why they had to obey and follow certain strange rules or else they would be punished. The feelings this caused were not just emotional but were converted into physical sensations such as feeling that they were being kept in a prison from which they had small chances of escaping. Sometimes they had no possibility to use the telephone, and the car keys were hidden away or the car was never left for them to use.

(He) shut me in, kind of […], was not allowed to borrow the car, and took it to work even if he did not need to, just so that I would not be able to go anywhere. (W1)

The women also described how their partners withheld or controlled money. Two women were entirely financially dependent on the man with absolutely no money of their own, and others did not have enough money for food or they needed to borrow money to go shopping.

#### As if they were worthless

The women brought up many examples of how they were subjected to all kinds of severe physical and sexual violence and threats of such violence from their partners. The women also described psychological violence such as limitations, prohibitions, harassment, isolation, depreciation, subordination, manipulation, destruction of dear belongings, threats of losing custody of their children, and how the partner showed jealousy or tried to make the women jealous. The partners often used foul language, sometimes even in front of others, and would often tell the women to shut up and/or roar out such things as *bloody whore*. The women were criticized, humiliated, and felt worthless as a person.

Yes, he thought that almost everything I did was wrong […] yes I became smaller and smaller, you see… (W3)

The women were the recipients of so many negative statements in so many different ways, and felt depreciated as both partners and mothers, that they had lost almost all sense of self-worth (*cf*. 21).

#### To always be on one's guard

For all the women living in a relationship with ongoing violence, the constant anxiety of further violence seemed to be the worst part. The violence was like an omnipresent shadow and tension in the relationship even during days when nothing happened. The outbursts were often unpredictable, but sometimes there were certain *signals* indicating when it was important to be more watchful and when the woman might be able to keep calm and make her partner listen to reason. To further avoid the violence that was often felt to be life threatening, the women learned to get out of the way, to adapt, to behave in a certain way, and not to give free rein to their own personalities and to obey him unquestionably. However, all of this was often fruitless because the partners looked for reasons to get angry and to start conflicts, and then it was difficult to stop them. Thus, the women almost never felt safe.

… you don't know how to act… you can walk on tiptoes, but you know it makes no difference whatever you do, however you try to fit in with him, you never know how he will react…(W7)

One woman recalled an alternative way of dealing with her partner's violence. She sometimes reacted to his signals with a fit of anger of her own, such as throwing things at him, as a way of speeding up his violent outbursts. She then knew that the violence would be over soon and that this would be followed by a period of calm.

A variety of strategies were described as ways to handle the everyday struggles, and one woman said that she did everything to focus her mind in a certain way, to pretend that her strange life situation did not exist. Sometimes certain parts of the violent events were repressed and just fragmented memories remained such as *it happened in the living room* and *I was lying on the floor* and the details of the violence were forgotten.

### Having sex

#### Sex as violence and as a part of the abusive relationship

The women described how their lovers had turned into perpetrators and some also described how sex had changed from a matter of desire to one of aversion. In seven of the nine narratives, sex was described as something that had become more and more negative. One of the women described the situation as follows.

Yes, if I just get this done so I can avoid it later, then I will be let off that nagging […] but then finally it became harder and harder for the body[…] it did not want to, it did not work…(W4)

One woman told about her partner wanting to have sex after the violence as a way to say he was sorry, but she refused. Some women were physically forced to have sex while others viewed it as a necessity, something they had to take part in without any pleasure for themselves. Thus, sex was often used to avoid or postpone violence and even to calm him down after a violent act. To refuse sex was a risk, so even if the women did not have any desire for it, having sex could be used as a strategy to safeguard themselves and their children from something that might be even worse. One woman (W9) could not understand how her partner first could tell her how *fat, ugly, and disgusting she was, and, ugh, he did not even want to touch her with a bargepole* and then he suddenly wanted sex. It seemed to her as if men in general believe that sex and love are two totally separate matters. However, it was best just to agree to it, otherwise he would spank her. But she felt empty and disgusted, and as a way to endure she did not interact at all.

Yes, I did nothing and then I got to hear that I was inanimate, I was equivalent to sticking it into the mattress … (W9)

In general, to refuse sex or not was like choosing between the plague and cholera, and sex without warm feelings was nothing the women could get used to but rather it became worse and worse. One partner always wanted sex, and although the woman tried everything to escape, she felt she often just had to give in to his demands.

… I was so terribly disgusted with him. And then he said afterwards that ‘It felt just as if I had raped you.’ I thought to myself, ‘Can't you understand, that is precisely what you did except that I didn't lash out or fight’. (W7)

One of the women lived in a relationship where she was forced to have sex as often as three times every night. If she refused, he would discipline her by using violence such as pinching or kicking her out of the bed or he behaved badly the next day. He felt that if she did not allow him sex she did not have the right to sleep there, it was his bed. This even continued during pregnancy and immediately after she came home with the baby from the maternity ward (they were even discharged early from the hospital because he wanted her to come home).

Just for this reason, because if he was not allowed to sleep (have sex) with me three times, then it became a downright rape, yes the first…..is also a rape, you see… but… (W3)

One of the women told about how her partner, in the midst of a sexual act that had begun in a nice way, could just change it into an assault. Maybe she said it hurt and that resistance turned him on. She also told about being raped when she was at the end of one of her pregnancies, she was having a shower and he came in to her.

Then I chose to do nothing, that time I did absolutely nothing. I said I did not want to and then I just cried, then I did not do anything more. (W4)

For this woman, talking about the violence during pregnancy, and revealing the sexual aspects of it in particular, was like dragging the whole pregnancy through the dirt, and she did not even want to think about her children finding out about it.

#### Sex as the only thing that actually worked

Two of the nine women were able to maintain a positive give-and-take intimacy and were able to keep their sexual life separate from the violent relationship. It was like having two separate relationships with the same man. When having sex, the partners were suave, took an interest in them, and saw them as worthy individuals.

Our sex life functioned well; I have never felt threatened sexually, no, not at all. There he was like another person, then he was soft, it felt like I became close to him…no, he was never, he has never been thre….threatening in that way… (W1)

One of these two women said that when they were having sex, the partner was so aroused that he gave her the attention she never got otherwise, although she admitted that this was less pronounced during the really bad periods. However, sex was generally a relief for these two women, a breathing space, like it happened with him as another person, a gentle person. This made it possible for the women to feel closeness and intimacy.

### Becoming pregnant

#### I did not want to become a parent

Some narratives included a reluctance to become parents, and three pregnancies occurred despite a strong desire to avoid becoming pregnant and to enter parenthood. One woman had been living in a relationship with continuous sexual violence, and with a potential child in mind these are the thoughts she had at the time.

… So I sort of hit out ‘don't want to, don't want to, don't want to’ since he… I was on the pill, then suddenly they were just lost, you know, none left. When I had a loop (IUD) then he hurt me so terribly bad when I had that, so I had to have it removed because I could not manage … since he wanted me to become pregnant. And definitely, then when he found out that I had an abortion a new trial started, ‘how could I be so mean to him?’. (W3)

This woman knew that becoming a mother meant that she would have to take full responsibility for the child and she did not see herself as a good mother, but she felt forced to have a child. Finally, after she had used contraceptives, had abortions, and desperately tried to protect herself from her partner coercing her into pregnancy, she gave up her opposition. The pregnancy did not give her any respite; the partner continued the sexual violence just as before.

#### So I became pregnant

Most of the pregnancies described were unplanned and the women gave the impression of having a rather ambivalent stance or a laissez-faire attitude towards the pregnancies. One woman was reluctant to take contraceptive pills even though she had gone through an earlier pregnancy that had started the violence, and one did not mention using any contraceptives despite experiencing escalating violence.

… No, it was not planned. Actually I had these golden pills, since I took no contraceptive pills or anything and we should protect ourselves then, but at that point it was not so much of that then … (W4)

Regarding these unintended pregnancies, the women seemed to have left things more or less to chance, used no or ineffective protection, and had not really thought much about the possibility of becoming pregnant. When one of them told her partner that she was pregnant, he wanted her to have an abortion but she refused. From these women's point of view, sex was mainly something that was neither anticipated or planned for, but rather often happened in spite of themselves and was associated with very tense and violent circumstances. Consequently, contraception was not a natural subject of conversation and was not something that was easily negotiated.

#### A wanted child

Some women talked about a joint or individual desire to have a child. For two women, having a baby was a decision they made themselves and was something they really wanted; the child was supposed to be a love child (these two women were not the same two women who described sex as a positive experience in the previous section about having sex).

But then if one should talk about the pregnancy now, well, there was nothing happy about it, you know, unfortunately it was not so. You see, this was supposed to be a love child … (W6)

The women had this desire for a child despite their complicated relationships with steep ups and downs and where they had broken it off with their partner and then been reunited. One of the women even suffered ongoing severe violence while planning for a baby.

I actually had Olivia (not her real name) because I really wanted to. I really gave 100% because I really believed in our relationship. I really believed that … this (the violence) is not true, something like this can't happen. I thought I really was able to overcome this, I really believed that … (W7)

These two women did not actually believe that a baby would eliminate the negative aspects of their relationships. Instead, having a baby seemed to sanction the positive aspects of the relationship, their long-standing friendship, passion, love, strong feelings, and a belief that they were meant for each other.

## Discussion

According to the interviews, besides the threats and the physical and sexual violence, their partners also made the women feel criticized, humiliated, and worthless. The women also felt very restricted in their relationships, isolated from friends, and limited in their lives outside the household. Despite this and many contradictory and ambivalent feelings, two of the women in this study described having sex as a reciprocal desire and as a respite from the rest of the relationship, but the others gave a negative and totally different picture. These women viewed sex as either obligatory or as a necessity to prevent or soothe aggression, or brought it up as rape and something that was physically forced upon them. The women's descriptions of their pregnancies ranged from being carefully planned and mostly wanted to being completely unwelcome and as occurring after flawed contraceptive efforts and previous abortions.

### Methodological considerations

There might be some uncertainty regarding the selection of women because the selection was carried out by the coordinators of the women's crisis centres where all of the women had sought help at some point. Thus, the coordinators could have used some additional inclusion/exclusion criteria besides the written instructions from the interviewer. Moreover, the women told retrospective stories, some of which had occurred several years prior to the interview. However, this is not believed to be a severe limitation because research has found that memories of harrowing events remain rather stable over long periods of time (36, 37). While the first author did a detailed analysis shortly after the interviews that resulted in narrative themes, some years have passed since then, and that might be viewed as a limit. Conversely, we believe that both authors’ later discussions and comparisons between the initial narrative themes and the entire interview transcriptions have been important and might even strengthen the final analytical interpretations and the trustworthiness of the study.

Our narrative study gives a deepened understanding of abused women's life situations (*cf*. 21) and is also related to studies about complexity, paradoxes and opposite discourses in violent relationships (*cf*. 17, 38, 39). However, we have not found other studies about IPV in the range between the extremes of desire and rape and wanted versus unwanted pregnancies. We believe that the results of our study are transferable knowledge and can add useful insight into the complexity and manifold nature of abused women's intimate relationships.

### Analyses

During the analysis, it became obvious that in their narratives the nine women described (directly or indirectly) different power strategies used by their partners and different coping strategies that they themselves used to deal with their violent relationships (*cf*. 21). Furthermore, the narratives reported on turning points such as becoming pregnant and ‘breathing spaces’. The latter refers to a tendency in the narratives to keep positive aspects of the relationships separate from the negative aspects, which we analysed as a form of compartmentalization. Accordingly, the following discussion is divided into the following four sections: (1) Power strategies, (2) Coping strategies, (3) Turning points, and (4) Compartmentalization.

### Perpetrator power strategies

A significant amount of literature on IPV is related to power strategies and coping strategies. Power strategies are often categorized in terms of physical, sexual, and psychological violence (*cf*. 39). Male dominance, power, and control are major explanations that are given for violence and IPV (1, 40–45). Sexual violence is often considered the ultimate form of power and control (40), not least in relation to aspects of the relationship dealing with sexuality and fertility (46, 47). Furthermore, ‘sexual assault perpetrated by an intimate partner may be especially traumatic’ (5).

In this study, violence was a regular occurrence in the relationship and different power strategies were used by the male batterers. The women were often beaten or stabbed in addition to being subdued, harassed, isolated, and emotionally manipulated. Another recurring male power strategy was the use of undesired and forced sex, which was described by the women as repugnant and disgusting but also as an inescapable part of the relationship and the violence or as something that could be used to calm the partner down and even to prevent violence or end it quickly (*cf*. 48). The women often said that they interpreted the sexual acts differently compared to their partners (*cf*. 49).

### Coping strategies

The power strategies used by the men in the relationships did not result in a totally passive life for the women. Instead the women used different strategies to cope with the violence and to overcome their situation.

Coping strategies aim to preserve physical and psychological well-being in situations of stress and have been categorized in different ways. It is common to distinguish between problem-focused versus emotion-focused coping and between engagement versus disengagement. Whereas problem-focused strategies attempt to change the problem that causes the distress, emotion-focused strategies attempt to deal with emotional responses to the problem (50). A point of departure for this study is the index of different IPV coping strategies that was developed by Goodman and colleagues (51). The authors distinguish among six different categories of strategies. First, women can use a ‘formal network’ in trying to get help and deal with the violence. Second, calling the police or filing criminal charges are examples of ‘legal’ strategies. ‘Safety planning’ is a third category, and it includes different activities aimed at reducing the violence, for example, hiding weapons. Fourth, by turning to ‘informal networks’, women seek help from family and friends. ‘Resistance’ refers to women actively trying to stop or reduce the violence. Sixth, by the use of ‘placating’ strategies, women intend to change the abuser's behaviour but not challenge his sense of control.

We have used this index to identify two recurring strategies. For example, some of the women used *placating strategies* when they tried to please their partner in different ways – for example, by allowing him sex even if she did not want it, as a way to temporarily stop the physical violence. The interviews also reflected *resistance strategies*. The women were critical, obstinate, spoke their mind, and made their own decisions. For example, one woman refused to have sex after a violent situation, and another used contraceptives against her partner's will. However, there were limitations to their strategies of resistance, and they usually failed to prevent further violence (*cf*. 48).

### Turning points

For some women in this study, becoming pregnant was an aggravating circumstance. The pregnancy was often described as a fateful moment (*cf*. 52) and as a special life experience of importance for both the men's power strategies and the women's responses (i.e. their coping strategies). Pregnancy can also be described as a turning point because it was in relation to it that the violence started, restarted, or escalated (*cf*. 21). With two exceptions, the pregnancy just happened or was unintended as a result of the failure or the counteracting of contraceptive efforts. This is in agreement with other research (53) and is explained by forced sex and the obstruction of fertility control (9–11). The most extreme example in this study was the partner who systematically raped the woman and foiled her use of contraception in order to coerce her into pregnancy (*cf*. 47).

Pregnancy was not only a turning point regarding the male batterers’ use of violence but was also an emotional or physical turning point for the women (*cf*. 49). Two women left their partners during their pregnancies because their situation had become life threatening (*cf*. 54), but the others did not. Pregnancy in a relationship with ongoing violence may bring with it additional dreams about idealized images of parenthood and expectations of support for the baby instead of becoming a single mother (55). This appeared to be the case when some women described having a baby as a ‘choice’. The motivation was not actually to stop the violence, but by becoming pregnant the women sought to maintain or emphasize the good parts of their relationships. Here romantic (and utopian) ideas of passion and long-standing friendship played a major part – ideas that often characterized the abused women's stories about the beginning of their relationships (*cf*. 56). Moreover, a woman subjected to violence might still feed, consciously or unconsciously, her ingrained ideas about the nuclear family in which becoming a mother and having children plays a central role (17).

### Compartmentalization

The narratives in this study were characterized by contradictions and complexities such as the description of the partner being a *Jekyll and Hyde* who alternated between being good and being bad (*cf*. 57). This cannot be fully understood through power strategies, coping strategies, or with reference to pregnancy as a turning point. This is especially the case when it comes to sexuality and the range between desire and rape. The most extreme example of this was the two women who reported that they had a good sex life despite living under violent circumstances. To be able to comprehend this, we have introduced another theoretical concept, compartmentalization. This refers to a mechanism with which people try to avoid cognitive dissonance, and conflicting values, interests, and emotions can be dealt with by keeping them separate. Positive compartmentalization – which is more common – occurs when an individual's positive self-aspects, attributes, and self-beliefs are important and accessible, and it often leads to a positive mood. Negative compartmentalization means that an individual's negative self-aspects, attributes, and self-beliefs are important and accessible, and it results in negative mood and low self-esteem ((58); *cf*. (59)). Compartmentalization has been described as a sign of poor self-cohesion (60), but also as a very effective way of organizing self-knowledge, especially if negative attributes can be avoided (61). Integration is in contrast to this type of organization of self-knowledge and is characterized by a blend of positive and negative self-ideas (62).

In our view, compartmentalization works not only on a cognitive level but also on a physical level. This means that an individual can keep different sets of actions separate. One physical behaviour can be related to positive self-aspects under one circumstance, but connected to negative self-aspects in another. In other words, compartmentalization also has a physical or bodily dimension.

By connecting sex with positive feelings, associations, and bodily experiences, and by separating it from the violence, two of the women could continue to enjoy their sex life and feel intimacy and closeness. It also appears that their partners did the same thing, and violence and other power strategies were often replaced with more gentle verbal and physical behaviours. This physical and two-way compartmentalization laid the foundation for a good sex life despite the violent circumstances of the relationship.

### Conclusions and implications for practice

The results in this study are in line with other studies showing that women subjected to IPV have diverse and complex experiences that affect all parts of the relationship (17, 38, 57, 63, 64). According to the narratives in our study, intimacy might turn into force and rape for some, but for others, sex does not necessarily exclude pleasure and desire and can be a haven of rest from an otherwise violent relationship. Accordingly, women may tell stories that differ from the ones expected as ‘the typical abuse story’, and this complexity needs to be recognized and dealt with when women seek healthcare, especially concerning contraceptives, abortions, and pregnancies (*cf*. 38). Thus, further support and training can help practitioners in meeting with patients or abused women (65, 66) and to ask the right questions about intimacy and contraceptive use. This may help them to differentiate healthy relationships from unhealthy ones, and this can be helpful also for both women who do and do not identify themselves as having relationship problems (8). Such counselling might give the healthcare provider information about inconsistency and coercion in the relationship and might provide women with an opportunity to disclose and give clues about ongoing IPV (8). It might also be important to take mechanisms of compartmentalization into consideration, especially in terms of a physical or bodily dimension. Abused women can, as was the case in this study, report on positive and enriching sexual experiences while at the same time living under violent circumstances. Such reports can be interpreted as signs of a rather healthy relationship, when they actually could be signs of the exact opposite.

## Footnotes

1 One woman, after the recorder was turned off, said she was still in love with this man, the one she once had wished for.

## References

[1] Heise L, Ellsberg M, Gottmoeller M (2002). A global overview of gender-based violence. Int J Gynaecol Obstet.

[2] McFarlane J, Malecha A, Watson K, Gist J, Batten E, Hall I (2005). Intimate partner sexual assault against women: frequency, health consequences, and treatment outcomes. Am Coll Obstet Gynecol.

[3] Stockman JK, Lucea MB, Campbell JC (2013). Forced sexual initiation, sexual intimate partner violence and HIV risk in women: a global review of the literature. AIDS Behav.

[4] Tjaeden P, Thoennes N (2000). Full report of the prevalence, incidence and consequences of violence against women (NCJ 183781).

[5] Temple JR, Weston R, Rodriguez BF, Marshall LL (2007). Differing effects of partner and nonpartner sexual assault on women's mental health. Violence Against Women.

[6] Bennice JA, Resick PA (2003). Marital rape: history, research, and practice. Trauma Violence Abuse.

[7] Goodwin MM, Gazmararian JA, Johnson CH, Gilbert BC, Saltzmanand LE, PRAMS Working Group (2000). Pregnancy intendedness and physical abuse around the time of pregnancy: findings from the pregnancy risk assessment monitoring system 1996–1997. Matern Child Health J.

[8] Miller E, Jordan B, Levenson R, Silverman JG (2010). Reproductive coercion: connecting the dots between partner violence and unintended pregnancy. Contraception.

[9] Campbell JC, Woods AB, Chouaf KL, Parker B (2000). Reproductive health consequences of intimate partner violence. A nursing research review. Clin Nurs Res.

[10] Pallitto CC, O'Campo P (2005). Community level effects of gender inequality on intimate partner violence and unintended pregnancy in Colombia: testing the feminist perspective. Soc Sci Med.

[11] Pallitto CC, Campbell JC, O'Campo P (2005). Is intimate partner violence associated with unintended pregnancy? A review of the literature. Trauma Violence Abuse.

[12] Shah PS, Balkhair T, Ohlsson A, Beyene J, Scott F, Frick C (2011). Intention to become pregnant and low birth weight and preterm birth: a systematic review. Matern Child Health J.

[13] Petersen R, Moracco KE, Goldstein KM, Clark KA (2004). Moving beyond disclosure: women's perspectives on barriers and motivators to seeking assistance for intimate partner violence. Women Health.

[14] Chang JC, Decker MR, Moracco KE, Martin SL, Petersen R, Frasier PY (2005). Asking about intimate partner violence: advice from female survivors to health care providers. Patient Educ Couns.

[15] Plichta SB (2007). Interactions between victims of intimate partner violence against women and the health care system: policy and practice implications. Trauma Violence Abuse.

[16] Jones C, Bonner M (2002). Screening for domestic violence in an antenatal care clinic. Aust Coll Midwives Inc.

[17] Goldner V, Penn P, Sheinberg M, Walker G (1990). Love and violence: gender paradoxes in volatile attachments. Fam Process.

[18] Chang JC, Decker M, Moracco KE, Martin SL, Petersen R, Frasier PY (2003). What happens when health care providers ask about intimate partner violence? A description of consequences from the perspectives of female survivors. J Am Med Womens Assoc.

[19] Feder GS, Hutson M, Ramsay J, Taket AR (2006). Women exposed to intimate partner violence: expectations and experiences when they encounter health care professionals: a meta-analysis of qualitative studies. Arch Intern Med.

[20] Thackeray J, Stelzner S, Downs SM, Miller C (2007). Screening for intimate partner violence: the impact of screener and screening environment on victim comfort. J Interpers Violence.

[21] Edin KE, Dahlgren L, Lalos A, Högberg U (2010). Keeping up a front” narratives about intimate partner violence, pregnancy and antenatal care. Violence Against Women.

[22] Edin KE, Högberg U (2002). Violence against pregnant women will remain hidden as long as no direct questions are asked. Midwifery.

[23] Wedding-Hedin LW, Grimstad H, Moller A, Schei B, Janson PO (1999). Prevalence of physical and sexual abuse before and during pregnancy among Swedish couples. Acta Obstet Gynecol Scand.

[24] Spangaro JM (2007). The NSW health routine screening for domestic violence program. NSW Public Health Bull.

[25] Jasinski JL (2004). Pregnancy and domestic violence: a review of the literature. Trauma Violence Abuse.

[26] WHO (2005). WHO multi-country study on women's health and domestic violence against women.

[27] Crossley ML (2000). Introducing narrative psychology, self, trauma and the construction of meaning.

[28] Kvale S, Brinkman S (2009). Interviews, learning the craft of qualitative research interviewing.

[29] Riessman CK (1993). Narrative analysis. Qualitative Research Methods Series, No. 30.

[30] Burr V (2003). Social constructionism.

[31] Mishler EG (2004). Historians of the self: restorying lives, revising identities. Res Hum Dev.

[32] Riessman CK (2008). Narrative methods for the human sciences.

[33] Starrin B, Dahlgren L, Larsson G, Styrborn S (1997). Along the path of discovery.

[34] Dahlgren L, Emmelin M, Winkvist A (2004). Qualitative methodology for international public health.

[35] Watts C, Heise L, Ellsberg M, Moreno CGM (2001). Putting women first: ethical and safety recommendations for research on domestic violence against women.

[36] McNally RJ (2005). Debunking myths about trauma and memory. Can J Psychiatry.

[37] Williams LM (1995). Recovered memories of abuse in women with documented child sexual victimization histories. J Trauma Stress.

[38] Piispa M (2002). Complexity of patterns of violence against women in heterosexual partnerships. Violence Against Women.

[39] Bacchus L, Mezey G, Bewley S (2006). A qualitative exploration of the nature of domestic violence in pregnancy. Violence Against Women.

[40] Babcock JC, Waltz J, Jacobson NS, Gottman JM (1993). Power and violence: the relation between communication patterns, power discrepancies, and domestic violence. J Consult Clin Psychol.

[41] Jewkes R, Levin J, Penn-Kekana L (2002). Risk factors for domestic violence: findings from a South African cross-sectional study. Soc Sci Med.

[42] Heise LL (1998). Violence against women: an integrated, ecological framework. Violence Against Women.

[43] Antai D (2011). Controlling behavior, power relations within intimate relationships and intimate partner physical and sexual violence against women in Nigeria. BMC Public Health.

[44] Firestone JM, Harris RJ, Vega WA (2003). The impact of gender role ideology, male expectancies, and acculturation on wife abuse. Int J Law Psychiatry.

[45] Felson RB, Messner SF (2000). The control motive in intimate partner violence. Soc Psychol Q.

[46] Campbell JC, Alford P (1989). The dark consequences of marital rape. Am J Nurs.

[47] Coggins M, Bullock LF (2003). The wavering line in the sand: the effects of domestic violence and sexual coercion. Issues Ment Health Nurs.

[48] Kennedy Bergen R (1995). Surviving wife rape: how women define and cope with the violence. Violence Against Women.

[49] Edin K, Dahlgren L, Högberg U, Lalos A (2009). The pregnancy put the screws on: discourses of professionals working with men inclined to violence. Men and Masculinities.

[50] Flicker SM, Cerulli C, Swogger MT, Talbot NL (2012). Depressive and posttraumatic symptoms among women seeking protection orders against intimate partners: relations to coping strategies and perceived responses to abuse disclosure. Violence Against Women.

[51] Goodman L, Dutton MA, Weinfurt K, Cook S (2003). The intimate partner violence strategies index – development and application. Violence Against Women.

[52] Giddens A (1991). Modernity and self-identity. Self and society in late modern age.

[53] Gazmararian JA, Adams MM, Saltzman LE, Johnson CH, Bruce FC, Marks JS (1995). The relationship between pregnancy intendedness and physical violence in mothers of newborns. The PRAMS Working Group. Obstet Gynecol.

[54] Mezey GC, Bewley S, Friend J, Mezey GC (1997). Domestic violence in pregnancy. Violence against women.

[55] Lutz KF (2005). Abuse experiences, perceptions, and associated decisions during the childbearing cycle. West J Nurs Res.

[56] Boonzaier F (2008). If the man says you must sit, then you must sit’: the relational construction of woman abuse: gender, subjectivity, and violence. Feminism & Psychology.

[57] Enander V (2010). Jekyll and Hyde or “Who is this guy?” – battered women's interpretations of their abusive partners as a mirror of opposite discourses. Women’s Stud Int Forum.

[58] Showers CJ, Zeigler-Hill V (2007). Integration and compartmentalization: the evaluative organization of contextualized selves. J Pers.

[59] Zeigler-Hill V, Showers CJ (2007). Self-structure and self-esteem stability: the hidden vulnerability of compartmentalization. Pers Soc Psychol Bull.

[60] Radan A (2007). Exposure to violence and expressions of resilience in central American women survivors of war. J Aggress Maltreat Trauma.

[61] Showers CJ, Zeigler-Hill V, Limke A (2006). Self-structure and childhood maltreatment: successful compartmentalization and the struggle of integration. J Soc Clin Psychol.

[62] Limke A, Showers CJ (2010). Organization of parent knowledge: compartmentalization and integration in adult child–parent relationships. Pers Soc Psychol Bull.

[63] Peled E, Eisikovits Z, Enosh G, Winstok Z (2000). Choice and empowerment for battered women who stay: toward a constructivist model. Soc Work.

[64] Walker R, Logan TK, Jordan CE, Campbell JC (2004). An integrative review of separation in the context of victimization: consequences and implications for women. Trauma Violence Abuse.

[65] Beynon CE, Gutmanis IA, Tutty LM, Wathen CN, MacMillan HL (2012). Why physicians and nurses ask (or don't) about partner violence: a qualitative analysis. BMC Public Health.

[66] Committee on Health Care for Underserved Women (2013). Reproductive and sexual coercion. Committee opinion. Am Coll Obstet Gynecol.

# Sub article: Access to and use of sexual and reproductive health services provided by midwives among rural immigrant women in Spain: midwives’ perspectives

**DOI:** 10.3402/gha.v6i0.22645

**Published:** 2013-11-08

**Authors:** Laura Otero-Garcia, Isabel Goicolea, Montserrat Gea-Sánchez, Belen Sanz-Barbero

**Affiliations:** 1 National School of Public Health, Institute of Health “Carlos III”, Spain; 2 GESEC, Lleida University, Lleida, Spain; 3 Epidemiology and Global Health Unit, Department of Public Health and Clinical Medicine, Umeå University, Umeå, Sweden; 4 Faculty of Nursing, Lleida University, Lleida, Spain; 5 Ciber of Epidemiology and Public Health (CIBERESP), Spain

**Keywords:** gender, health services accessibility, immigrants, midwives, primary health care, qualitative research, rural population, sexual and reproductive health, utilization, women's health

## Abstract

**Background:**

There is insufficient information regarding access and participation of immigrant women in Spain in sexual and reproductive health programs. Recent studies show their lower participation rate in gynecological cancer screening programs; however, little is known about the participation in other sexual and reproductive health programs by immigrant women living in rural areas with high population dispersion.

**Objectives:**

The objective of this study is to explore the perceptions of midwives who provide these services regarding immigrant women's access and participation in sexual and reproductive health programs offered in a rural area.

**Design:**

A qualitative study was performed, within a larger ethnographic study about rural primary care, with data collection based on in-depth interviews and field notes. Participants were the midwives in primary care serving 13 rural basic health zones (BHZ) of Segovia, a region of Spain with high population dispersion. An interview script was designed to collect information about midwives’ perceptions on immigrant women's access to and use of the healthcare services that they provide. Interviews were recorded and transcribed with participant informed consent. Data were analyzed based on the qualitative content analysis approach and triangulation of results with fieldwork notes.

**Results:**

Midwives perceive that immigrants in general, and immigrant women in particular, underuse family planning services. This underutilization is associated with cultural differences and gender inequality. They also believe that the number of voluntary pregnancy interruptions among immigrant women is elevated and identify childbearing and childrearing-related tasks and the language barrier as obstacles to immigrant women accessing the available prenatal and postnatal healthcare services.

**Conclusions:**

Immigrant women's underutilization of midwifery services may be linked to the greater number of unintended pregnancies, pregnancy terminations, and the delay in the first prenatal visit, as discerned by midwives. Future research should involve samples of immigrant women themselves, to provide a deeper understanding of the current knowledge, attitudes, and practices of the immigrant population regarding reproductive and sexual health to provide better health services.

Research on migrant access and utilization of health services has proliferated in the last decades due to the growth of immigrant population settling into Europe (1). Results point to a lower utilization rate of services by immigrants compared to native-born, although figures display great heterogeneity due to the diversity in host country, place of origin of the immigrants, and the specific healthcare services examined (2–4).

Several authors have associated this lower utilization rate to the commonly healthier status of the immigrant population, compared to the native-born, as well as to the existence of barriers to access and use of health services (5, 6). These barriers gain more relevance in rural areas with high population dispersion where the distance to reach health facilities alone limits access and use of services (4).

The fertility rate rebound observed in Spain from 2006 until today (1.38 children/woman) is a reflection of the recent substantial increase in immigration rates (7–11) Immigrant women residing in Spain exhibit different sexual and reproductive health patterns from native-born Spaniards: greater fertility, lower age at first birth, greater rates of premature births and more births to infants with low birth weight (7, 8, 12, 13), as well as a higher proportion of voluntary terminations of pregnancies (VTP) (7, 13).

Despite having quantitative data on these differences, information on access to and participation in sexual and reproductive health programs by immigrant women in Spain is very scarce. Recent studies show a lower rate of participation in gynecological cancer screening programs among these women (14), but little is known about participation in sexual and reproductive health programs, especially among immigrant women living in rural areas. Given this gap in the literature, the objective of our study is to examine the perceptions of the professionals providing these services, the midwives, on the topic. The actual implementation of public health programs is strongly dependent on service providers, who may observe, adapt or completely ignore the programs (15). Their attitudes and practices can enhance or hinder women's access to and use of services. With this purpose, midwives were interviewed regarding access and participation in sexual and reproductive health programs offered in an area with high population dispersion.

## Methods

The geographical context of this study is the rural area of Segovia, a province in one of Spain's Autonomous Regions, known as Castile and Leon. This is the largest region in the country and the one with the lowest population density (27 inhabitants/km^2^).

About 64% of the population of Segovia (104,895 inhabitants) (16) lives in rural areas composed of 208 municipalities and 17 local authorities. The area has a high population dispersion (28% of the municipalities report fewer than 100 inhabitants) and a low population density (23 inhabitants/km^2^) (16). Segovia is experiencing population loss and population aging: 21% of the population is over 65 years of age (16), though this figure is only 12.5% in the receiving immigrant population (16).

Segovia's health area is divided into 16 basic health zones (BHZ), of which three are urban and 13 are rural. Each BHZ has a primary care center where the midwife's office is located. Seven midwives cover the 13 rural BHZ, where one midwife may cover between one and three BHZs.

Based on an ethnographic design, this study focused on primary care and healthcare processes in the rural environment (17). The main author between February 2008 and November 2009 performed fieldwork, including interviews. The qualitative study design included in-depth interviews with seven midwives serving in rural areas. The interview script was designed to collect information about midwives’ views about access to and use of midwifery services (18).

After informing the participants about the goals of the study and guaranteeing confidentiality, their consent was secured for participating and recording the interviews. An external expert transcribed all the interviews. The transcriptions of these recordings were analyzed by two of the authors based on the qualitative content analysis approach (19).

The data analysis was developed by two of the authors who first read the transcripts. Then, they imported the text into Open Code software to manage the coding process (20). First, parts of the text relating to the research question were identified (meaning units) and short summarized versions of them were developed (condensed meaning units). From the condensed meaning units, codes were then produced. The codes were grouped together into two emerging categories, which relate to the manifest content of the text. Finally, a theme emerged that cut across both categories and refers to the latent content. During the analysis, consensus about the results was reached among three of the authors. To support this analysis, they also used notes collected during the fieldwork.

In this article, the word *midwife* includes both male and female midwives. The term *immigrant women* indicates women of foreign origin, whom midwives sometimes refer to according to their country of origin as *Moroccan*, *Bulgarian*, or *Rumanian*.

## Ethics approval

The research protocol was approved by the Ethics Committee of the Health Institute Carlos III (Spain). This study was funded by National Health Funding Research-project PI 080306.

## Results

Results regarding rural midwives’ perceptions are organized into two categories: (1) place of origin and socio-economic situation (being an economic immigrant) and gender (being a woman) affects family planning; (2) there are access barriers to and underutilization of available prenatal and postnatal healthcare services by immigrant women. The theme that combines both of these categories is: midwives’ perceptions of underutilization of sexual and reproductive services by immigrant women.

### Culture as the source of difficulties with family planning for immigrant women

According to rural midwives, immigrant populations residing in the rural areas of Segovia hardly engage in any family planning, which midwives interpret as a consequence of ‘cultural differences’.

In this area, immigrants are mostly Bulgarian and Romanian. They don't use contraception […] these are people who you explain things to and maybe you get them to agree with the analysis, or something, and then, they don't do it. They won't take the pill, they won't get an IUD, they don't use condoms … and I think it's cultural. (Midwife 4)

I think that immigrant women do not have the issue of prevention and family planning incorporated. It is true that until they are sick many do not come to me. It can be cultural. (Midwife 3)

When asked to elaborate on what they meant by *cultural differences*, midwives explained that it was the men who decided whether or not to use family planning methods, since they often held negative attitudes regarding taking any action around contraception and whether it is for the woman or themselves. Thus, the decisions of men prevail over women's decisions.

Many are influenced by their husbands, their partners. They tell you that their husbands are in control. Then, you feel very frustrated. I ask them-What about an IUD? No, my husband doesn't want me to get an IUD. –What about the pill?- No, my husband doesn't want me to take anything. –Well, then tell your husband to use a condom- But he doesn't want to use a condom either. (Midwife 2)

Immigrant women know about contraceptive methods because I explain to them when they come to see me, but for them it is easier not to use anything. Their partners don't even want to use a condom. (Midwife 5)

Less often, midwives perceived that immigrant women used family planning methods but without their partner's knowledge. This shows the decisions of men are above the decisions of women, gender inequality is evident in this case.

A few of my patients took the pill without their partners’ knowledge, but those are the exceptions. (Midwife 7)

Some women tell me they want to plan their families, but they do not want their partners to know, because they (the partners) do not want to plan. (Midwife 6)

Midwives asserted that if a few immigrant women and their partners used family planning methods, they would serve as an example within their close social circle.

If one started to use it they would encourage others, because here in Spain, 40 years ago no one took the pill and if anyone did, they were told: ‘Oh no, that′s really bad for you, it causes cancer, you grow hair, you gain weight.’ Whatever their friend tells them always works better than anything that I tell them. (Midwife 1)

When an immigrant woman starts to come to the office to plan then her sisters come, then her sistersin- law . . .. (Midwife 5)

When faced with an unintended pregnancy VTP is one of the options considered by immigrant women. Midwives reported that VTPs are more common among women from Bulgaria and Romania as a consequence of the family planning policies in these countries, which are based on easy accessibility to VTP. In this way, midwives made references to the social, cultural, educational differences in immigrant women's notion of VTP, pregnancy, and family planning.

I have realized that Bulgarian and Rumanian women use abortion as a method of family planning. (Midwife 2)

There are countries like Bulgaria and Romania, where family planning was based on voluntary abortion. Abortion was promoted, and I believe that makes it less important to go for a visit to check there are no problems with the pregnancy, similarly, they don't have the same take on contraception that we do. (Midwife 3)

Some of the midwives pointed out that immigrant women did not always go to specialized centers to carry out these procedures (VTP), among others, but instead, some searched for alternate strategies despite the risks.

There is a medication, not sure they all know about it, but I'm convinced they get it. It's sold on the internet. I visited the site once and they give you addresses and phone numbers. I bet that they are selling it. It's used in the hospital environment for the stomach, but, of course, you're in a controlled environment. There's a risk of hemorrhaging, they may start bleeding and, just imagine, they think they've got it all out but a portion stays in, and can cause an infection. I've been asked for it, and the ones that ask for it are foreign women. Not Spanish women, which doesn't mean they don't know about it. (Midwife 3)

Midwives explained that these VTPs were performed either in Spain or in the immigrant women's country of origin, and that they (either themselves or their partners) bore the expenses.

There are Bulgarian women who go to Bulgaria for abortions. (Midwife 1)

Sometimes they abort here. Others go to their own countries, because, it's probably cheaper in their own country. Of course, here they might not be eligible for a legal abortion and they have to go to private clinics. That is why I think it's cheaper for them to go to Bulgaria. (Midwife 7)

Finally, midwives perceive that most of the teenage pregnancies occur among immigrant women.

I do not see many teenage pregnancies, but the few I see if there are more percentage in immigrant women. (Midwife 1)

Most of the teenage pregnancies we see are Romanian women from Rumania. (Midwife 4)

### Rural midwives perceived difficulties of access and use of prenatal and postpartum services among immigrant women

Midwives perceive that immigrant women make use of midwifery services mainly during pregnancies.

The only time when you really see a much higher proportion of immigrant women is during pregnancy. You don't see them during menopause, nor for contraception, but you see a few in Pap Smear and cervical cancer prevention programs, however during pregnancy is when you see them most. (Midwife 4)

For instance, I see Moroccan women in my office during their pregnancy and for post natal consultations, but I see them a lot less for pap smears. (Midwife 7)

However, midwives detect an underutilization of prenatal visits, which translates into a delayed first prenatal visit.

When Spanish women know they're pregnant they have the habit of going to their doctor or to the nurse, or the midwife, but they go to the health center. Immigrant women sometimes leave it longer. (Midwife 5)

There are women, especially Moroccans, who leave it longer to come. That's my experience. I think that if they come from a place where healthcare is not as accessible, then they are not used to going for medical care, and miss the usual first prenatal visit. (Midwife 6)

Regarding the program offering maternal education classes, midwives perceive that immigrant women use this program to a lesser extent than native-born women. Some of them compared immigrant women to women of Romany ethnicity.

About childbirth preparation group, sometimes I get that some Bulgarian or Moroccan women come. It is difficult to grasp them for activities like that. (Midwife 6)

They hardly come to the childbirth courses I offer, and if they attend one class, then they drop out. I'm not sure whether it is because it's silly, or because they can't follow it. You talk to them and then ask: -Do you understand? - and they answer affirmatively, but … It's the same thing with the gypsies, they don't come either. Maybe they think it's useless information, or they have other children to look after, or they have other things that prevent them attending … (Midwife 4)

Once again, midwives explain away this underutilization of their services based on ‘cultural differences’ regarding prenatal care. Some report that immigrant women think of pregnancy as a natural process which requires little supervision. Additionally, some midwives link this idea of immigrant women exhibiting an underdeveloped preventative culture with the fact that the immigrant population living in rural areas has a low socio-economic level. Other midwives talk about how women, in particular Moroccan women, do not attend these group activities because their husbands do not allow them.

Moroccan women relate to their children, with her husband, and very little with the rest of the people. I think we have a hard time doing group activities. I think they have restrictions by such husbands to attend childbirth preparation classes. In their culture the woman is in the private sphere. (Midwife 1)

It could be that for them pregnancy is not such a big deal … I don't mean they don't think it's important, but that they don't see the need for so much vigilance; it's something natural, and nothing will go wrong. In their country of origin they do go to be seen, I mean that they do follow the prenatal care. In Romania and Bulgaria for example, they do go as after all those countries are not so underdeveloped. As many people point out, the issue is that this type of immigrant is not their country's average citizen, but come from a lower social background; therefore culturally, prevention and care are lower. (Midwife 5)

Midwives explain that immigrant women sometimes skip scheduled appointments with them as well as with obstetricians in specialized care, going without some of the diagnostic tests in the prenatal protocol.

Immigrant are less reliable with their appointments, often they don't turn up, then they arrive without an appointment expecting to see you whenever it suits them, and things can get a bit chaotic. Of course there are all sorts of people, but you do see this more often with immigrants. (Midwife 4)

They are not as reliable when it comes to appointments; they are less likely to show up. Then they come with no appointment to be seen when it suits them, and this creates a degree of chaos. Many people do it but it is more common among immigrants, and especially within the Bulgarian population. (Midwife 7)

Some of the midwives point out that these sets of behaviors distinguish immigrant women from native-born women, except in those cases when native-born women live in socially dysfunctional situations.

Access to healthcare is relatively easy. If they don't go it's because they don't want to, because as sometimes happens with these women (immigrants) they don't even show up for blood test and miss hospital appointments … It's not all of them, but you don't see Spaniards doing that, and if you do, it's usually an isolated case with a family with issues. […] If you have six such cases per year, five are foreigners and one is not. (Midwife 2)

Finally, midwives also refer to language limitations as an access barrier for women from non-Spanish-speaking countries. Language limitation also results in these women's partners or their own children assisting with any communication with health professionals.

We get many from Morocco, the majority. We always give them, books about pregnancy and all that for them to read, although we mostly communicate with the husbands who know more, are more up-to-date, or with the kids, who speak very well. (Midwife 1)

Today I started a childbirth preparation group which should have like ten women, some of them immigrants. […] It is difficult to grasp for group activities for different reasons. One is the difficulty with the language, the language barrier. (Midwife 6)

## Discussion

The reproductive patterns described by the rural midwives in our study reflect official figures (7, 8). Data published by INE (Spanish acronym for the Spanish National Institute of Statistics) support the perceived high rates of VTPs that midwives believe their patients endure. These data show that in 2010, 41.7% of all VTP were performed on immigrant women (21), which is a very high percentage considering that in 2010 only 13.4% of women residing in Spain were immigrants (16).

In addition, the number of VTPs recorded among these women may be underestimated since, as the midwives indicated, some of these procedures are performed in the immigrant's country of origin, or in Spain but outside the public healthcare system. Finally, midwives argued that women from Eastern Europe choose VTP because this was a common practice in their countries of origin, encouraged by the legislation originated in those popular democracies (22).

Considering that 31.9% of adolescent pregnancies occur among immigrant women (23); that immigrant women have higher rates of VTP and higher fertility rates than autochthonous women (1.61 births per immigrant woman vs. 1.33 children per Spanish-born woman); that birth rates are also higher among immigrant women (17 immigrant mothers per 1,000 inhabitants vs. 9.5 Spanish-born mothers), we can say that there are family planning deficiencies among immigrant women.

According to the midwives the underutilization of family planning services, also described in relation to the cervical cancer prevention program elsewhere (2, 3, 14) is, again, likely related to cultural differences, the main barrier being the male partner's opposition, due to gender inequality (24), and to certain family planning practices.

It is worth noting that rural midwives do not think that the distance from the immigrants' municipality of residence to the rural health center is a barrier when seeking midwifery services. The fact that this potential barrier, observed and documented in previous studies (4, 25), fails to be perceived as an obstacle may reflect the existence of the communication difficulties between midwives and immigrant women. It is also possible that the women who do go to the midwife's office are those with no access barriers or those who managed to overcome them. However, the fact that midwives fail to perceive certain barriers to access and use of healthcare services already identified by different studies carried out in the same geographic environment (14, 25) may indicate a ‘blaming the victim’ attitude toward immigrant women. This viewpoint could prevent the launching of strategies aimed at improving access as well as utilization.

In summary, the midwives in our study equated the pattern of delayed access and underutilization of care often found in immigrant women with that of women of Romany ethnicity. That is, they identified immigrant populations with other groups at risk for social exclusion, as reported in a recent study (14). Furthermore, the association that these professionals made between low socio-economic level characteristic of the immigrant population and an underdeveloped preventative culture (these last one originated by the fact that they proceeded from low-income countries with limited preventative programs) has also been reported in quantitative studies as an association between lower socio-economic level and a lower utilization of preventative services (8, 26).

Regarding the underuse of maternal education programs, midwives emphasized the communication difficulties, specifically the language barrier, as an obstacle to access and use of such programs, as previously described by other authors (27, 28). They also identified the burden of childcare and childrearing, mostly the woman's responsibility in these immigrant populations, as a potential access barrier to prenatal care and services. This finding may be an indicator of how immigrant women's health is negatively impacted by the absence of the support received from traditional family and social network (29, 30).

Finally, it is important to comment that while this study provides evidence about midwifes’ perceptions on access to and use of midwifery services, it does not include the perceptions of the immigrant women users of these services. It is important to underline that research about immigrant women's perceptions would provide key insights into areas glossed over under culture by the midwives, and it could be an interesting area for further research.

## Conclusions

This study revealed perceptions of an underutilization of midwifery care among immigrant populations residing in the rural area of Segovia. According to the midwives working in rural primary care, this underuse results in unintended pregnancies, possible VTPs, and in delayed prenatal care. Further research is needed to gain a deeper understanding of the current knowledge, attitudes, and practices of the immigrant population, both men and women, regarding family planning, voluntary pregnancy interruption, and prenatal care to better match the supply and demand. Therefore, it is also necessary as appropriate delivery of reproductive and sexual health services for the immigrant population residing in rural areas of Segovia.

## References

[1] Mladovsky P, Ingleby D, McKee M, Rechel B (2012). Good practices in migrant health: the European experience. Clin Med.

[2] Regidor E, Sanz B, Pascual C, Lostao L, Sánchez E, Díaz JM (2009). La utilización de los servicios sanitarios por la población inmigrante en España. Gac Sanit.

[3] Sanz B, Regidor E, Galindo S (2011). Influencia del lugar de origen en la utilización de pruebas de cribado de cáncer ginecológico en España. Rev Saúde Pública.

[4] Sanz-Barbero B, Otero-García L, Blasco-Hernández T (2011). The effect of distance on the use of emergency hospital services in a Spanish region with high population dispersion: a multilevel analysis. Med Care.

[5] Akhavan S (2012). Midwives’ views on factors that contribute to health care inequalities among immigrants in Sweden: a qualitative study. Int J Equity Health.

[6] Rué M, Cabré X, Soler-González J, Bosch A, Almirall M, Serna MC (2008). Emergency hospital services utilization in Lleida (Spain): a cross-sectional study of immigrant and Spanish-born populations. BMC Health Serv Res.

[7] Instituto Nacional de Estadística (2012). Movimiento Natural de la Población e Indicadores Demográficos Básicos. Natalidad. Instituto Nacional de Estadística. http://www.ine.es.

[8] Luque MA, Bueno-Cavanillas A (2009). La fecundidad en España, 1996–2006: mujeres de nacionalidad extranjera frente a españolas. Gac Sanit.

[9] Malmusi D, Pérez G (2009). IVE legal en mujeres inmigrantes en un entorno urbano. Gac Sanit.

[10] Márquez-Calderón S (2009). Influencia de la población inmigrante en la variabilidad de la tasa de IVEs entre comunidades autónomas. Gac Sanit.

[11] Zuriaga O, Martínez-Beneito MA, Galmés Truyols A, Torne MM, Bosch S, Bosser R (2009). Recourse to induced abortion in Spain: profiling of users and the influence of migrant populations. Gac Sanit.

[12] Río I, Castelló A, Jané M, Prats R, Barona C, Más R (2010). Reproductive and perinatal health indicators in immigrant and Spanish-born women in Catalonia and Valencia (2005–2006). Gac Sanit.

[13] Gispert R, Clot-Razquin G, Torné MM, Bosser-Giralt R, Freitas-Ramírez A (2008). Diferencias en el perfil reproductivo de mujeres autóctonas e inmigrantes residentes en Cataluña. Gac Sanit.

[14] Otero L, Sanz B, Blasco T (2011). Detección precoz de cáncer de cérvix según los discursos de las matronas de atención primaria en Segovia, España. Rev Saúde Pública.

[15] Walker L, Gilson L (2004). We are bitter but we are satisfied’: nurses as street-level bureaucrats in South Africa. Soc Sci Med.

[16] Instituto Nacional de Estadística (2008). Padrón. Población por municipios. http://www.ine.es.

[17] Otero-García L (2012). Campos de Soledad. Atención primaria y procesos asistenciales en pueblos de la provincia de Segovia. tesis doctoral.

[18] GRSCyL-SACYL Cartera de servicios de atención primaria; 2009. http://www.saludcastillayleon.es/institucion/es/catalogo-prestaciones/cartera-servicios-especializada.

[19] Graneheim UH, Lundman B (2004). Qualitative content analysis in nursing research: concepts, procedures and measures to achieve trustworthiness. Nurse Educ Today.

[20] ICT Services and System Development and Division of Epidemiology and Global Health (2013).

[21] Ministerio de Sanidad, Política Social e Igualdad Interrupción voluntaria del embarazo. Datos estadísticos. http://www.msps.es/profesionales/saludPublica/prevPromocion/embarazo/tablas_figuras.htm.

[22] United Nations Development Programme-Romania A decade later: understanding the transition process in Romania. National Human Development Report. Romania 2001–2002. http://hdr.undp.org/en/reports/nationalreports/europethecis/romania/romania_2001_en.pdf.

[23] Instituto Nacional de Estadística (2012). Movimiento Natural de la Población. http://www.ine.es.

[24] Scott-Samuel A (2008). Patriarchy, masculinities and health inequalities. Gac Sanit.

[25] Borda A, Sanz B, Otero L, Blasco T, García-Gómez FJ, de Andres F (2011). Tiempo de viaje y participación en el cribado del cáncer de mama en una región de alta dispersión poblacional. Gac Sanit.

[26] Regidor E, Gutiérrez-Fisac JL, Fernández de la Hoz K, Rodríguez C (1996). Diferencias socio-económicas en la utilización y accesibilidad de los servicios sanitarios en España. Med Clín.

[27] Woltman KJ, Newbold KB (2007). Immigrant women and cervical cancer screening uptake: a multilevel analysis. Can J Public Health.

[28] Seguí M (2005). El inmigrante en la consulta del médico de cabecera. Semergen.

[29] García-Calvente MM, Mateo-Rodríguez I, Eguiguern AP (2004). El sistema informal de cuidados en clave de desigualdad. Gac Sanit.

[30] Bernosky de Flores CH (2010). A conceptual framework for the study of social capital in new destination immigrant communities. J Transcult Nurs.

# Sub article: Performing masculinity, influencing health: a qualitative mixed-methods study of young Spanish men

**DOI:** 10.3402/gha.v6i0.21134

**Published:** 2013-09-16

**Authors:** Jorge Marcos Marcos, Nuria Romo Avilés, María del Río Lozano, Juan Palomares Cuadros, María del Mar García Calvente

**Affiliations:** 1 Institute for Women's and Gender Studies, University of Granada, Granada, Spain; 2 Andalusian School of Public Health, Granada, Spain; 3 Department of Didactics of M.P. and Body Expression, Faculty of Education Sciences, University of Granada, Granada, Spain

**Keywords:** gender, masculinity, men's health, young men, social theory, health inequalities, qualitative research

## Abstract

**Background:**

The literature shows how gender mandates contribute to differences in exposure and vulnerability to certain health risk factors. This paper presents the results of a study developed in the south of Spain, where research aimed at understanding men from a gender perspective is still limited.

**Objective:**

The aim of this paper is to explore the lay perceptions and meanings ascribed to the idea of masculinity, identifying ways in which gender displays are related to health.

**Design:**

The study is based on a mixed-methods data collection strategy typical of qualitative research. We performed a qualitative content analysis focused on manifest and latent content.

**Results:**

Our analysis showed that the relationship between masculinity and health was mainly defined with regard to behavioural explanations with an evident performative meaning. With regard to issues such as driving, the use of recreational drugs, aggressive behaviour, sexuality, and body image, important connections were established between manhood acts and health outcomes. Different ways of understanding and performing the male identity also emerged from the results. The findings revealed the implications of these aspects in the processes of change in the identity codes of men and women.

**Conclusions:**

The study provides insights into how the category ‘man’ is highly dependent on collective practices and performative acts. Consideration of how males perform manhood acts might be required in guidance on the development of programmes and policies aimed at addressing gender inequalities in health in a particular local context.

Nowadays, gender is considered to be largely a social construct with important direct implications for health. Thus, most social scientists agree that the definition of gender is not merely a personality trait but also a social system that restricts and influences patterned behaviour (1). This conception is widely connected with the development of explanatory models of inequalities in health research.

The study of gender inequalities received an important boost with the advent in the 1980s of the critical study of men and masculinity. This approach started as part of the feminist critique of sex role socialization theory, by not considering aspects of privilege and power within their analytical framework (2, 3). Similarly, the constructivist approaches began to establish that the constituent traits of masculinity may vary historically and culturally, which implies the discussion of multiple masculinities. This idea was primarily developed around the notion of hegemonic masculinity (4). While this has led many researchers to emphasize hegemonic masculinity as a cultural ideal, where the focus is placed on documenting the various ways of ‘being a man’, little research has been conducted on unpacking the interactional processes through which the most honoured way to be a man is locally constituted (5). That is, sight has been lost of the analysis of its consequences.

In recent decades, the understanding of gender and health issues has been widely favoured by the development of relational theory. The relational approach gives a central place to the patterned relations between men and women (and among women and men) that constitute gender as a social structure (6). Within the terms of this approach, West and Zimmerman's ‘doing gender’ perspective emphasizes the way gender is accomplished in personal interaction (7). At the same time, Butler's conceptualization of gender, from a different ontological perspective regarding the possibility of a self, has added to the discussion of ‘doing gender’ in critical ways, helping to sharpen the focus on performativity (1). Thus, taking the phenomenological perspective of ‘acts’, gender may be understood as a constructed entity that involves the repetition of acts that define shared experience as a form of ‘collective action’ (8). It is precisely this kind of gender display that has been highlighted in the literature as a characteristic element in understanding men and masculinity, describing how manhood acts as a form of subjectivity. These ‘manhood acts’ not only imply a claim to membership of the privileged gender group but also may lead to health damage (9). From a public health perspective, this damage has often been particularly associated with the behavioural aspects of ‘manhood acts’.

Scientific evidence shows that the largest gender gaps in morbidity and mortality are caused by behavioural differences between men and women (10). The literature emphasizes important differences between how men and women define and prioritize their health (11). In the case of men, one of the key factors in disease and death rates is related to their poor relationship with health care services (12, 13). Epidemiological research has consistently shown that men use health care services less than women (14) and that they are less involved in preventive and health-promoting initiatives (15). At the same time, the research has noted disparities in health literacy, in terms of how men and women access and apply the information available (16). The literature shows that many men are driven to not ask for help, as offering no sign of vulnerability is an essential trait of masculinity (6). Mental health is a significant case in point. Thus, for example, with regard to the symptoms of depression, men are often underdiagnosed and undertreated (12). This mental disorder is often associated with weakness and a loss of emotional control in men (17). At the same time, many men display their mental and emotional distress in a different way than women. This has been linked in the literature with the decline of social support networks (18), and especially with substance abuse.

In the vast majority of cultures, being considered to be ‘a man’ is a condition that must be ‘achieved’ (19). The excessive intake of recreational substances, especially alcohol, is a major cultural expression related to masculinity in the literature (20). Despite having a higher risk of falling into drug use, men of all ages perceive the risk of this activity as being significantly lower than women do (21). In parallel with drug use, masculinity has been also closely related to violent behaviour, unsafe driving, and risky sexual behaviour (22). More recently, within the studies on men's health, masculinity has been studied in relation to body image (23).

Self-confidence is strongly influenced by body image in youth populations (24). While this has been specifically studied in women, it is now also considered to be an important predictor of psychological well-being in young men (25). In Western societies, this has mainly been analysed from the perspective of the utilitarian symbolism of the muscular mesomorph. Thus, some researchers have shown a significant association between traditional masculine ideology, the pursuit of muscularity, and body image discrepancy (26). In this way, muscles become a representational sign of power and strength that, at the same time, can also lead to crash dieting, the consumption of nutritional supplements, and, in more extreme cases, the clinical symptoms of muscle dysmorphia (27).

Social expectations and stereotypes attached to gender in relation to the behaviour that is considered appropriate are factors with a great influence on the sexual behaviour of young men (28). The experience of masculinity is closely related to the exercise of sexuality (29), since heterosexuality is a key factor associated with hegemonic rules of masculinity (30). In this way, having an active sex life or ‘luck with the ladies’ may be interpreted as signs of possessing a masculine self, which has been described as being related to promiscuous behaviour and risky sexual practices (31, 32). Also, in Western societies, the vast majority of reckless drivers are young men (33). In Europe, statistics have shown that men are three times more likely to suffer an accidental death than women (34). Apart from other structural reasons, the greater tendency of men to, for example, exceed a speed limit is linked to the fact that risk is naturalized and promoted in men (35). Moreover, in all countries, men aged 15 to 45 years are both the main perpetrators and victims of homicide (36). In terms of socio-anthropological approaches, physical strength in males is at the epicentre of the association between masculinity and violence (37). The consideration of violence as a part of the global public health agenda, along with the development of ecological models of analysis, has reinforced the idea that this relationship is mainly the result of men's socialization to be dominant and the symbolic structures of the social power that perpetuate such domination (38). These last considerations gain special meaning in contexts such as the Spanish one.

After the early opening movements of the dictatorial Franco regime in the second half of the twentieth century, numerous Anglo-Saxon researchers focused on the study of society and Spanish culture. Their works constituted the basis of the study of masculinity in contemporaneous Spain (39–41). These studies, which focussed almost exclusively on rural Andalusia, described a strongly sexist masculinity where men were acknowledged as the only providers for the family unit. Men's reputations are especially described around considerations of sexuality. Thus, for example, while honour is presented as a multidimensional feature of social life, it is often associated with sexual control over women in the family, including the insistence on virginity in single people, fidelity in marriage, and the sexual abstinence of the widow. Despite the existence of these works, the study of men is still considered to be at an embryonic stage (42). This is particularly significant with regard to the study of health from a gender perspective. The vast majority of the studies that have followed this approach have focussed on comparing indicators of illness outcomes among men and women.

In Spain, the life expectancy of males is 78.5 years, 6 years shorter than that of women (43). The mortality rate from chronic diseases in people under 75 years of age is highly unfavourable to men (44) and includes more mortality from diseases that occur mainly as a result of bad health habits (such as an unbalanced diet or the use of illegal drugs, tobacco, and/or alcohol). This higher rate of mortality is also the result of external causes, such as accidents, falls, aggression, or suicides. For instance, 78.2% of deaths resulting from motor vehicle accidents occur in males, with the highest percentages being between the ages of 16 and 24 years (43). These considerations connect with the main idea of the last report by the European Commission regarding the state of men's health: there is a high level of preventable premature morbidity and mortality in men, which will be addressed only by targeted activity across their lifespans (45).

The way in which men position themselves in relation to different discourses on masculinity has important implications for the social processes of health and disease (46). In the case of young people, this theoretical basis provides a context for the idea that the higher health risks faced by men are the result of factors and behaviours that could be modified (47). Although the literature describes numerous projects and interventions designed from a gender perspective aimed at improving the health and well-being of men and women, many of these programmes have rarely shown an interest in the point of view of the men themselves (48). This emphasizes the need for an investigation of the experience of the processes of health and disease in men in different contexts. Thus, bringing out the characteristics of masculinity is to be understood as a way of contributing to the identification of local, regional, and global interactions (49). This project was designed to explore through men's narratives, from a gender perspective, the complexity of young men's worldview (their ‘lay knowledge’) and its relationship to their health; such narratives include the stories that youths express about themselves and others. The project's research focus was on the diversity of how masculinity and other practices related to health operate in the daily lives of men. In terms of men's health studies, this article takes a critical approach by carrying out an analysis of practices identified as ‘manhood acts’; that is, of what men do individually and collectively to signify their membership in the category of ‘man’ (9). This might be a useful way to reveal how men's health practices can be seen as mechanisms for constructing gender through a series of acts that are renewed, reviewed, and consolidated over time. Thus, the aim of this article is to describe the lay perceptions and meanings ascribed to the idea of masculinity, and identify ways in which young men relate gender displays to health.

## Methods

This qualitative study is based on fieldwork developed between March 2009 and April 2010 in Andalusia in southern Spain. Following intentional non-probabilistic sampling, 59 participants were recruited from youth centres and educational institutions. From the beginning, the research team tried to bring about the greatest possible control of bias when choosing sample units. This required the pursuit of multiple independent networks, unrelated to the research team. The size of the sample was strictly related to the search for different profiles. The general inclusion criteria were being a man aged 15–24 years and being born and raised in Andalusia. The sons of immigrants and young men from the Roma community were not included since the presence of specific characteristics within their cultural framework was assumed. In order to ensure the heterogeneity of the participants’ profiles, three criteria were established: age (15–17 or 18–24), origin (cities, towns, or villages), and level of education (ranging from primary education to university studies).

Nine individual interviews, eight focus group discussions, and a triangular group session were conducted. The combination of these methods enabled the socio-cultural factors and personal aspects of the object of study to be explored more deeply. The focus groups were particularly useful for identifying the lay perceptions of the expectations and signs of social desirability and behaviour that are considered appropriate. The individual interviews enabled the lay knowledge of the participants to be explored in depth. In the later part of the fieldwork, we conducted a triangular group (50). This qualitative technique, which involves three participants, was introduced because it allowed us to create a more interactive group dynamic, in which even the researcher–interviewer may have a more active role during the course of the discussion. In a triangular group, there is usually more tension between the particular positions of each participant and the common elements (or consensus view) than in an orthodox discussion group. This allowed us to confront certain emerging perspectives more exhaustively. This mixed-methods data collection strategy promoted the use of multiple information sources and different approaches to gain new insights into the object of study.

The fieldwork was developed in two consecutive and complementary stages. The first one was designed with regard to some general principles of the grounded hermeneutic approach (51). During this phase, an individual interview and a focus discussion group were carried out. With regard to the focus group, the selection of the participants was determined so that their profiles would contain certain types of heterogeneity. This criterion was established so as to contrast different positions with regard to the object of study. During this first phase, the discussion was stimulated by presenting the participants with a sequence of photographs related to the world of youth that gave them the possibility of expressing their ideas about each of them. The preliminary analysis of the data collected during the first phase of the fieldwork enabled the identification of significant topics and dominant discourses that were a basis for the elaboration of the topic guide that was developed for the second phase of the fieldwork. This guide was formulated around 10 conceptual dimensions: 1) men's typical, 2) women, 3) youth, 4) health, 5) risk, 6) violence, 7) sexuality, 8) body image, 9) feelings, and 10) homosexuality. During the second phase of the fieldwork, eight semi-structured interviews, seven focus discussion groups, and a triangular group session were carried out. In this phase, the discussion groups were formed so that they would involve homogeneous profiles. The completion of the data collection process was determined according to the principle of saturation (52). All the interviews were recorded and transcribed literally by a member of the research team. The transcriptions were completed using the interviewer's field notes.

We carried out a qualitative content analysis. Because of the aims and theoretical perspective of the study, the research team decided from the beginning that the analysis process would extract not only the manifest content but also the underlying meanings (53). Thus, after a general reading of the transcriptions, the text about the men's behaviour as being gendered was extracted and brought together into one text, which constituted the unit of analysis. Later, the text was divided into units of meaning that were condensed. At the same time, the condensed meaning units were abstracted and labelled with a code. The dimensions and sub-dimensions proposed for the analysis were applied by different members of the research team so that there was agreement upon their definition and how they would be applied, thus lending reliability to the analysis process. The conceptual classification of codes and categories was constructed by using both a deductive research approach and an inductive procedure. Thus, the process of analysis began with a predetermined list of topics to be explored and a category system that had previously been defined after the first phase of the fieldwork. In the same way, any concepts, categories, or sub-categories that emerged from the examination of the data during the analysis process were integrated into the analysis.

The revision of concepts and categories and the encoding process were carried out by the main researcher in collaboration with a specialist in qualitative methodology external to the research. This procedure was not only part of the triangulation process but also a way of adding higher confidence to the analysis. The texts were sorted into seven conceptual areas, which constituted the basis of the manifest content: 1) femininity versus masculinity, 2) health and well-being, 3) sexuality, 4) body image, 5) substance abuse, 6) driving, and 7) violence. During the last phase of the analysis process, these elements were integrated into two themes: 1) representing power: body image and violence; and 2) typically masculine: driving, drugs, and sex.

To facilitate the dual process of interpretation (manifest and latent content), the analysis was developed with the support of the QSR NVivo 8 program and took a hermeneutic approach involving the following steps: 1) a general reading of the texts to obtain a sense of the whole; 2) the review of any topics and examples arising, with an analysis of shared and unshared content and their significance in light of each profile; 3) the identification of interrelated topics; and 4) an analysis of the ways in which gender identity was related to life experiences that influence health (54). In this sense, the research team focused on manhood acts – the ways to signify masculine selves. Thus, we interpreted the data by looking for what participants said about how men learned to perform “manhood acts,” how and why these acts can vary, and how they can reproduce gender inequalities.

The research protocol that forms the basis of this article was approved by an internal commission established by the European Public Health Master ‘Europubhealth’. According to the current Spanish law (Organic Law 15/1999 of 13 December), the personal data provided by the participants were stored and kept in a safe file, and real names were replaced by fictitious ones. Participation was voluntary, and the participants received information about the objectives of the study as well as about the institutions involved. All participants signed an informed consent form. Parents or guardians’ consent was required for underage participants.

## Results

### Representing power: body image and violence

Body image and the aggressive use of force were aspects that were mainly linked to the social representation of power in men's actions. These were related to the perception of the body as a source for signifying manhood, emphasizing it as a tool for control, dominance, and activity. Men are socialized to use the body to symbolize manhood. In this process, according to the participants, the practice of sport was considered central. From childhood, men are more exposed to playing competitive games based on physical performance, which encourages them to display fortitude, control the experience of physical pain, and endure intense effort. For instance, in the specific case of football, ‘toughness’, ‘aggressiveness’, and ‘courage’ were words used to justify why a sport was seen as being more suitable for men.

CARLOS: If you are playing football and a player kicks you and you complain, somebody may tell you that this is a man's game. But the truth is that when you are participating in the competition you have to fight aggressively for the ball, because that can influence the final result. When somebody kicks you hard, rivalry arises. (Individual interview, town, 22 years old, enrolled at university)

These traits were considered to be factors with important health implications, especially in terms of risk-taking behaviour and the gender differences involved in coping with illness. With regard to the latter, reluctant attitudes towards the use of medicines and medical consultation were recognized by participants as behaviour traditionally embedded in ideologies of masculinity, which are related to physical and emotional toughness. Although some participants admitted to going to the doctor every time they perceived any anomaly in their health status, a large majority said that they only went ‘when it is really necessary’. This was also an attitude that the participants associated with the idea of being a responsible user of health care services. The following excerpt not only is a good example of this view but also suggests how men's underutilisation of medical services may be considered a ‘manhood act’.

PEDRO: All the girls have a little bag full [of] medicines at home. And as soon as they have the slightest headache, they take a tablet; a stomach ache, one pill; another sort of pain, they go to the doctor. And I have to be really sick to go to the doctor.… it's also true that if a friend [boy] goes to the doctor every day, we say: ‘What's wrong with this guy, he's always at the doctor! He gets sick so easily!’ (Group interview, city, 20–23 years old, enrolled at university)

Disruptive behaviour and episodes of vandalism or physical aggression were especially considered to be recurrent actions by men. This was a generalized perception of the participants in the study, including those who, like Ricardo, emphasized the need for speaking about plural masculinities.

RICARDO: You cannot really lump all men together. There are thousands of different types of men and each of them is different from the other. The only thing that differentiates us from women is physical appearance, but regarding attitudes, that depends on education. A man can be as crass as a woman. But it is true that I have seen boys jumping on cars or burning containers and I still haven't seen a woman doing that. I can't find an explanation as to why men have to fight and women don't. It's an attempt to show that I'm more than you are. For men, he who is the stronger is more so than the other. (Individual interview, town, 19 years old, enrolled at university)

During this study, references to violence against women were minimal. When they were made, the participants took positions of rejecting this type of violence. The more widely reported forms of violence were those that were established among men. Although most of the participants argued against the use of violence, they all declared that at some point they had witnessed a fight between men or had been directly involved in it. Violence was mainly described as a formula for proving superiority and gaining respect. Thus, the aggressive use of force reinforced the notion that to be a man is to be dominant. In this sense, violent behaviour was also connected with the idea of protection, widely considered to be a part of the masculine essence by the participants.

ÓSCAR: Men fight more often. I might have seen a few girls pulling each other's hair, but that's it. But men fighting …. As for me, when I get into fights I don't stop until I see the other guy bleeding. Why? Because my balls are bigger than his. (Group interview, village, 15–17 years old, vocational training)

RAÚL: I think we have the obligation to provide women with one thing: security. For instance, if I had a daughter with a boyfriend, and for any reason they came back home saying that they were mugged and I found out that he didn't do anything, he'll be in big trouble. He's the one who has to come back with a broken jaw. And the other way round too. If I found out that something happened to her and that my son did nothing! (Individual interview, village, 23 years old, musician)

Data suggest that concern about body image is a growing phenomenon among men within the context of the study. Whether for reasons of hygiene or aesthetics, references to practices such as depilation were common. Although these are still considered minority practices, the participants expressed the view that these customs are part of the social embodiment of contemporaneous masculinity. Less standardized practices were also identified, such as the use of make-up as a way to increase the possibilities of seduction. Thus, ‘feminine’ behaviour, such as cosmetic use, is acceptable by men if it has a performative function related to heterosexual conquest.

GUSTAVO: Some of my friends get waxed and use make-up. I see it as faggoty, though I'm talking about guys who like girls! They feel more handsome and that it attracts girls. (Individual interview, town, 21 years old, primary studies, currently unemployed)

However, our findings indicate that when men are interested in improving their body image, they focus mainly on muscle development. Muscles were considered a key element within current masculine subjectivity, especially related by participants as an element of the representation of power and strength and characterized as a symbol of seduction. This was linked by participants to the perception of an increasingly widespread use of nutritional ergogenic aids among young men. The use of protein supplements was particularly reported. Although some participants expressed opposition to the use of this type of dietary supplement, the vast majority of participants described these products as harmless, and they considered professional consultation and/or prescription to be unnecessary. Participants also reported cases in which, as a way of achieving the desired muscle growth, some men had turned to the use of anabolic steroids. Although the participants considered the number of young men who accessed these products to be low, the lay perceptions that emerged from the analysis are of great interest. In spite of being products that can only be purchased legally under medical prescription, the perception of ease of access to them in gyms or shops that specialize in sports nutrition was general across our informants.

JAVIER: Gyms supply proteins. There are many shops that sell them too. Proteins are legal, not anabolic steroids or any other chemical substances, but if you wanted them, you could get them easily. You can get them from your monitor in the gym. I'm sure he is a wrestler and he deals with this stuff. It is the same as if you are a police officer who knows where to go to get cocaine; isn't it? (Group interview, city, 20–23 years old, enrolled at university)

### Typically masculine: driving, drugs, and sex

During this research, the risks associated with driving were those most directly related to health. ‘Caution’ was a term that was widely used to define women's attitudes toward driving motor vehicles. Exceeding speed limits, driving under the influence of alcohol or (in the case of motorcycles) without a helmet, and showing skills like driving on a single wheel were described as typically male forms of behaviour; that is, forms of social representation of manhood through which men seek attention and recognition from their peer group.

CARLOS: We do wheelies because someone always starts – first to show off in front of us and then, if there are any girls around, to show off in front of them. It is a way of proving that you are the fittest or the cockiest; the most daring, because you don't ride like the rest. If everybody did it, it wouldn't be cool. It's like you have to try and do something different. (Individual interview, town, 22 years old, enrolled at university)

When participants referred to the consumption of recreational substances, their statements were dominated by references to alcohol, and more specifically to *botellón*.^1^ All the participants in the study, including minors, referred to *botellón* as being a common practice in their leisure time. Some young men showed resistance to traditional gender traits, positioning themselves as being in favour of responsible use and/or expressing awareness of the health risks of alcohol abuse; for others, those risks were not so obvious. Thus, heavy drinking per se was not considered risky. It was only seen as such when combined with other risky forms of behaviour.

FERNADO: I see that all of us got drunk but that's not risky, is it? Well, it is risky if you are drunk and you are riding a motorbike, which is quite dangerous. (Group interview, town, 15–17 years old, enrolled at high school)

Most of the participants acknowledged that alcohol abuse is still judged by society differently for men and women. Despite this double standard for alcohol use, the data indicated the existence of an established perception among participants: girls have become involved in a ‘masculinization’ process regarding alcohol abuse at weekends. On the other hand, binge drinking is a practice rooted in the process of construction of the meaning of ‘being a man’. It is seen as a practice symbolizing manhood; a performance of *until the body gives up* closely related to the process of achieving a feeling of belonging to a peer group. In some cases, as described by Raúl, the heavy consumption of alcohol as a ‘manhood act’ becomes a ‘revised’ gendered act over time.

RAÚL: When you were younger and went to a ‘botellón’ on an empty stomach you'd have seven or eight drinks. That's definitely no good for your health and you know it, but whether you want to or not, you do it because everybody does. I never drank as much as the others did because I didn't like it, but I drank because everybody else did. Now, when we go to play football, everybody drinks a pint of beer. I don't. I drink a Coca-Cola. Still today everyone goes: ‘Come on. You're a guy!’ Now I don't care, but when I was 16 …. (Individual interviews, village, 23 years old, musician)

With regard to expressions like *we men are more vicious*, the participants revealed their belief that the use of other recreational drugs is also a habit that is more prevalent among men. Of special significance were the references made to marijuana and hashish consumption. These recreational substances were considered to be in widespread use among young Andalusian men. Participants also expressed the opinion that the harmful effects of these substances were not significant. The idea of risk with regard to the use of psychoactive substances was particularly associated with ‘synthetic drugs’. Overall, the perception of participants was that the use of these types of substances was increasing. These drugs were connected to having a lack of inhibition and were described as being facilitators of casual relationships. The only informant who openly expressed having used the drug ecstasy throughout this research declared that he had done it as a way of experiencing new feelings during sex.

Sexuality was considered by the participants as being of vital importance to the construction of male subjectivity. Participants focused their attention on the tensions between the cultural and biological explanations of men's behaviour. The core element of the discussion revolved around the sexual appetite. Those who believed that sexual desire is greater in men resorted to arguments with a biological background by using concepts such as *instinct*, *essence*, or *nature*. However, other participants considered that if such beliefs are socially established, it is because men tend to be expected to be more open about their sexual desires. While for a man such openness is considered to be a sign of manhood, women tend to care more about such conduct since it may lead them to be judged more negatively by society in terms of their morality. Although our findings show that many girls no longer play the passive role that traditionally characterized them when establishing a relationship, the participants expressed the view that they are generally the ones who take the initiative, or they are aware that girls pretend not to do so in order that they are not labelled as ‘easy’.

LUIS: I think we have a sexual drive, I don't know if it's because of our age or because we are men, it's just not normal!

JUAN: But don't get me wrong; it's both men and women, isn't it? What happens is that women are more reserved than we are. We show it more openly. We're cheekier and chicks know it. (Focus group, 19–21 years old, enrolled at university)

The results point to a widespread lay perception that men live their sexuality in a less emotional and more genital-based way. This was frequently related to other perceptions: 1) the importance of satisfying the sexual expectations of a partner (either stable or occasional), and 2) the greater promiscuity of men. With regard to this, different ways of living one's sexuality and characterizing masculinity were expressed. The results highlight the need to consider multiple forms of masculinity in relation to sexual life. In fact, some participants distanced themselves from ‘masculine’ predatory heterosexuality, while others contributed to reinforcing it. These two opposing positions can be deduced from the words of Marcos, Víctor, and Pablo:

MARCOS: I think sex is the same as eating, it's a basic need. Why not do it if the occasion arises one night with a girl that you know wants what you want?

VÍCTOR: I don't like to sleep with a different girl every night. It's as if it were a jacket instead of a chick. It might sound a bit backwards, but I have my morals. When I was 16 I, like my friends, used to go for the first one that came by. Now I look for other things in a woman, not just sex. (Triangular group, 19–22, different educational background)

PABLO: I had a girlfriend until about three weeks ago, and I wasn't going around saying: ‘I screw her this way and that!’ But when I go out and get laid, the first thing I think of is to call a buddy to tell him about it. It's like if you don't tell anyone, it's as if it didn't happen. (Individual interview, 20 years old, electrician)

From a sexual point of view, all these considerations around men's identity were closely connected to issues related to risky sexual behaviour and the possible power relations between males and females. Although some participants mentioned the prevention of sexually transmitted diseases, the idea of risk in this field was mainly associated with potential pregnancy. Likewise, the debate about whether or not to use a condom was usually linked to the distinction between being a stable partner or an occasional partner. Not only were condoms associated with a loss of sensitivity, but also not using them was associated with a leap of confidence in an intimate relationship. Having unprotected sex was described as a sign of manhood among the peer group, and this was sometimes seen as an added motivation for not using a condom:

GUILLERMO: Men also use this behaviour as a way of asserting masculine superiority. If I tell some friends that I have never done it [without a condom,] they feel ‘superior’, and they boast that they do it without a condom. That's the reason for them to take the initiative to start doing it without a condom. (Individual interview, 24 years old, enrolled at university)

Moreover, during this work, most participants showed that with an occasional partner they tend to use a condom, but many of them (including some minors) admitted to having had unprotected sex at some point.

DAVID: With my regular partner, I have done it without a condom. Well, I've also done it with a girl I met on a trip. We did it without a condom because she was taking those pills every day. And I believed that. It seemed like the girl came from a good family. (Focus group, 18–22 years old, vocational training)

## Discussion

This article, which is based on a qualitative study of young Andalusian men, has explored the lay perceptions and meanings ascribed to the idea of masculinity, identifying ways in which gender practices are related to health. The gender differences in health outcomes between men and women are mainly linked to men's display patterns. Thus, a lower level of use of both health services and pharmaceutical drugs, as well as a greater tendency to practise physical activities and sports, were some of the core aspects of the behaviour of the participating men. The relationship between masculinity and health is mainly defined according to behavioural explanations with a clear performative character. Ideas about reckless driving and violent behaviour established important connections between the perceptions of health and ‘manhood acts’. In addition, different ways of understanding and performing men's identity were revealed through the data gathered on the attitudes to practices regarding male body image, the exercise of sexuality, and the use of recreational substances. There was also a clear relationship to the processes of change in the identity codes of men and women. Such changes appeared to have health implications and some significance in terms of the development of gender inequalities.

Our findings are consistent with those studies that identify muscle development as the main source of concern about body image among men (56). The literature shows that through muscle development, the male body image becomes a symbolic asset in the social representation of the hegemonic traits of masculinity (57). With regard to our findings, this is of special significance when aggressive behaviour is associated with a perception that supremacy is related to the tendency to demonstrate personal strength and protective ability. Our results also emphasize the importance of peer groups, and above all women, in this social representation process. For those who are labelled as ‘winners’, especially in practices of seduction and conquest, the mesomorphic body type is a source of respect and prestige, which has health implications for men. In this case, the findings have called attention to the increased consumption of nutritional ergogenic aids, particularly protein supplements and what have in recent years commonly come to be called *fat burners*. The use of anabolic steroids was also identified. This synthetic substance, designed to imitate the effects of testosterone, is associated with considerable risks for the reproductive and cardiovascular systems (58). The literature also describes how the difficulty of legal access to these substances often leads to the use of the black market, where products are purchased without any health and safety assessment (59). This is an issue of great interest in the public health field that has been barely studied. This is especially true if, as our findings indicate, easy access to this type of substance is widely assumed.

If gender is a historically and culturally constructed social category, and risk taking is understood as an element in this process, the use of substances should be a core aspect of this. This aspect has traditionally been emphasized as a trait that is close to the essence of what is considered masculine, especially in relation to alcohol consumption (60). Our findings show that the health risks arising from alcohol use are related not only to excesses but also to the sexual abuses and violent forms of behaviour that can ensue. Likewise, the results reveal the inverse functionality of alcohol to the identities of men and women. In Spain, some of the recent literature has shown how men interpret the assumption by women of lifestyles that have been considered traditionally more appropriate for men, such as ways of achieving a higher level of social equality and personal freedom (61). Recent studies have demonstrated that the traditional links between gender and alcohol consumption may be under revision (60, 62). In this way, while this process of the ‘behavioural migration’ of women is especially noticeable when it comes to alcohol use (63), the increase in the use of other psychoactive substances is another issue of interest in our context (64). Thus, for women, the development of consumption patterns similar to those of men could be considered to be a way of undoing gender, or of changing social interactions (65) by creating new forms of femininity that involve complicity with ‘manhood acts’. Using this approach, the dichotomy between doing and undoing gender could involve carrying out practices that for some people indicate perpetuating values and dominant beliefs, while others take them as a basis for contributing to their transgressions. According to our findings, the functioning of this gender differential is present not only in relation to the use of recreational substances but also in a proactive role in sex.

The study has identified the common lay perceptions of a type of sexuality in which men have less need for emotional intimacy. It has also indicated that sending signs of willingness to have sex is a ‘manhood act’ rooted in what is believed to be expected from a man. While the participants in the study have described a new form of femininity characterized by the expression of a more explicit and active form of sexual identity, the literature shows that when men want to regain the initiative and/or preserve their dominant position they often try to limit the sexual advances of women (66). This is another relevant issue with health implications for future research in this area, including research into the perspectives of the subjects involved – women and men alike. The literature highlights the influence of this on the social imagination, particularly with regard to the fact that men have generally been represented as being more proactive, that behaviour such as infidelity has been considered part of their biological essence, and that having unprotected sex is categorized as a ‘manhood act’ (9). These are practices that could be playing an important role as performative acts in the identity reaffirmation of heterosexual men, and in the development of power relations between males and females.

## Conclusion

This qualitative exploratory study provides information on the knowledge, attitudes, and practices that characterize young men's identities in the current Spanish context. The findings show that the category ‘man’ as a social construct is highly dependent on collective practice. Thus, focusing on ‘manhood acts’ from the point of view of their performative character provides the key to understanding men's health in a more holistic way, and it also helps to explain the inequalities in health between women and men. Similarly, information about forms of behaviour and ways of signifying masculinity that challenge the traditional codes has emerged. More research is needed, however, into how these non-hegemonic modes develop and operate in society, particularly with regard to how some young men establish a masculine identity while still rejecting ‘typically male’ stoic attitudes, dominant practices, and risky behaviour. In other words, their thoughts and practices do not act as mechanisms that signify and reproduce traditional manhood. This clearly also has implications for health outcomes in men and women.

The ‘manhood act’ perspective might provide a useful framework for the analysis of gender inequalities in health, using a life course approach. Our article highlights the importance of social representation in the public sphere in shaping masculinity. The way in which power relations are shaped and are at the root of gender inequalities has been described extensively in the literature; this reinforces the relevance of analysing not only the acts of the ‘builders of masculinity’ in a given context, but also how the notions and behaviours that constitute the meanings of masculinity are subject to change over time. From the perspective of a critical explanatory framework of men's health that is focused on ‘manhood acts’, the joint consideration of ‘practice’ and ‘performativity’ – in the terms described by Pierre Bourdieu and Judith Butler – may reinforce the relevance of studying how daily acts are gendered and socially located, and this could provide new insights in future research into how inequalities in health are embodied by men and women within a society. Some recent literature has emphasized that a key factor in advancing the understanding of men's health is located in the development of gendered epidemiology, so that through this we can begin to unpack how men's health practices can be mechanisms for ‘doing gender’ (67). With regard to this, our findings might improve the interpretation of epidemiological data in our particular context. In addition, the approach taken in this article, by directing attention to how men perform ‘manhood acts’, may facilitate awareness both of the complexity of the links between men, masculinity, and health, and of the norms, power dynamics, and practices that perpetuate health inequalities. We consider that the health sector can play a key role in the processes of social engineering to address these disparities. In this sense, it is important that health professionals are as responsive to the singular needs of men (and women) as they are to the gender-based barriers faced by them with regard to their health. In the same way, our findings suggest that although the practitioners have an important role in promoting male access and use of health care services, this engagement should go beyond simply giving attention to preventive physical health and lifestyle advice. It should address wider issues related to gender norms and social practices that perpetuate inequalities; that is, promoting responsible fathering and parenting, engaging men as caregivers, addressing gender-based violence, and so on. Thus, in order to increase the effectiveness of programmes and interventions that promote the questioning of attitudes and behaviours related to ‘unhealthy masculinity’, policies must facilitate the integrated development of gender that is mainstreamed into different social settings, without forgetting the health care system.

## Footnotes

1 *Botellón* refers to a social phenomenon that has become popular in public spaces in Spain since the end of the twentieth century. This consists of mass meetings of young people, mainly to chat while consuming some type of drink (55).

## References

[1] Risman RJ, Davis G (2013). From sex roles to gender structure. Curr Sociol.

[2] Lorber J (1994). Paradoxes of gender.

[3] Carrigan T, Connell B, Lee J (1985). Toward a new sociology of masculinity. Theory Soc.

[4] Connell RW, Messerschmidt JW (2005). Hegemonic masculinity. Rethinking the concept. Gen Soc.

[5] Schrock D, Padavic I (2007). Negotiating hegemonic masculinity in a batterer intervention program. Gen Soc.

[6] Schofield T, Connell RW, Walker L, Wood JF, Butland DL (2000). Understanding men's health and illness: a gender-relations approach to policy, research, and practice. J Am Coll Health.

[7] West C, Zimmerman DH (1987). Doing gender. Gen Soc.

[8] Butler J, Bial H (2004). Performative acts and gender constitution. The performance studies reader.

[9] Schrock D, Schwalbe M (2009). Men, masculinity, and manhood acts. Annu Rev Sociol.

[10] Stanistreet D, Bambra C, Scott-Samuel A (2005). Is patriarchy the source of men's higher mortality?. J Epidemiol Community Health.

[11] Emslie C, Hunt K (2008). The weaker sex? Exploring lay understandings of gender differences in life expectancy: a qualitative study. Soc Sci Med.

[12] Farrimond H (2011). Beyond the caveman: rethinking masculinity in relation to men's help-seeking. Health.

[13] O'Brien R, Hunt K, Hart G (2005). ‘It's caveman stuff, but that is to a certain extent how guys still operate’: men's accounts of masculinity and help seeking. Soc Sci Med.

[14] Noone JH, Stepherns C (2008). Men, masculine identities and health care utilisation. Sociol Health Illn.

[15] Robertson LM, Douglas F, Ludbrook A, Reid G, van Teijlingen E (2008). What works with men? A systematic review of health promoting interventions targeting men. BMC Health Serv Res.

[16] Peerson A, Saunders M (2011). Men's health literacy in Australia: in search of a gender lens. Int J Men's Health.

[17] Emslie C, Damien R, Ziebland S, Hunt K (2006). Men's accounts of depression: reconstructing or resisting hegemonic masculinity?. Soc Sci Med.

[18] House JS, Landis KR, Umberson D (1988). Social relationships and health. Science.

[19] Frosh S, Phoenix A, Pattman R (2002). Young masculinities: understanding boys in contemporary society.

[20] Courtenay WH (2000). Constructions of masculinity and their influence on men's well-being: a theory of gender and health. Soc Sci Med.

[21] Petersen A (2003). Research on men and masculinities. Some implications of recent theory for future work. Men Masc.

[22] Robertson S (2007). Understanding men and health: masculinities, identity and well-being.

[23] Kimmel SB, Mahalik JR (2004). Measuring masculine body ideal distress: development of a measure. Int J Men's Health.

[24] Perry DG, Pauletti RE (2011). Gender and adolescent development. J Res Adolesc.

[25] Tager D, Good GE, Bauer Morrison J (2006). Our bodies, ourselves revisited: male body image and psychological well-being. Int J Men's Health.

[26] Martin J, Govender K (2011). Making muscle junkies”: investigating traditional masculine ideology, body image discrepancy, and the pursuit of muscularity in adolescent males. Int J Men's Health.

[27] Grieve FG (2007). A conceptual model of factors contributing to the development of muscle dysmorphia. Eat Disord.

[28] Marston C, King E (2006). Factors that shape young people's sexual behaviour: a systematic review. Lancet.

[29] Hirsch JS, Muñoz-Laboy M, Nyhus CM, Yount KM, Bauermeister JA (2009). They “miss more than anything their normal life back home”: masculinity and extramarital sex among Mexican migrants in Atlanta. Perspect Sex Reprod Health.

[30] Phillips DA (2006). Masculinity, male development, gender, and identity: modern and postmodern meanings. Issues Ment Health Nurs.

[31] Lowe P (2005). Contraception and heterosex: an intimate relationship. Sexualities.

[32] Holmes D, Gastaldo D, O'Byrne P, Lombardo A (2008). Bareback sex: a conflation of risk and masculinity. Int J Men's Health.

[33] OECD (2006). Young drivers: the road to safety. http://www.internationaltransportforum.org/jtrc/safety/YDpolicyBrief.pdf.

[34] Evans J, Frank B, Oliffe JL, Gregory D (2011). Health, Illness, Men and Masculinities (HIMM): a theoretical framework for understanding men and their health. J Men Health Gend.

[35] Harre N, Foster S, O'Neill M (2005). Self-enhancement, crash-risk optimism and the impact of safety advertisements on young drivers. Br J Psychol.

[36] Krug EG, Dahlberg LL, Mercy JA, Zwi AB, Lozano R (2002). World report on violence and health.

[37] Mosse GL (1996). The image of man: the creation of modern masculinity.

[38] Bourdieu P (2001). Masculine domination.

[39] Brandes S (1980). Metaphors of masculinity: sex and status in Andalusian folklore.

[40] Driessen H (1983). Male sociability and rituals of masculinity in rural Andalusia. Anthropol Q.

[41] Gilmore DD (1987). Aggression and community: paradoxes of Andalusian culture.

[42] Valiente C (2002). An overview of research on gender in Spanish society. Gend Soc.

[43] INE – Instituto Nacional de Estadística (2011). Mujeres y hombres en España. http://www.ine.es/ss/Satellite?L=es_ES&c=INEPublicacion_C&cid=1259924822888&p=1254735110606&pagename=ProductosYServicios%2FPYSLayout&tittema=Sociedad.

[44] Artazcoz L, Moya C, Vanaclocha H, Pont P (2004). La salud de los adultos. Gac Sanit.

[45] White A, de Sousa B, de Visser RO, Hogston R, Madsen SA, Makara P (2011). The state of men's health in Europe.

[46] Robertson S (2006). ‘Not living life in too much of an excess’: lay men understanding health and well-being. Health.

[47] World Health Organization (2000). Boys in the picture: gender based programming in adolescent health and development in Europe.

[48] Barker G, Ricardo C, Nascimento M (2007). Engaging men and boys in changing gender-based inequity in health: evidence from programme interventions.

[49] Connell R (2012). Gender, health and theory: conceptualizing the issue, in local and world perspective. Soc Sci Med.

[50] Conde F, Gordo López AJ, Serrano Pascual A (2008). Los grupos triangulares como espacios transicionales para la producción discursiva: un estudio sobre la vivienda en Huelva. Estrategias y prácticas cualitativas de investigación social.

[51] Addison RB, Crabtree BF, Miller WL (1999). A grounded hermeneutic editing approach. Doing qualitative research.

[52] Strauss A, Corbin J (1990). Basics of qualitative research. Grounded theory procedures and techniques.

[53] Graneheim UH, Lundman B (2004). Qualitative content analysis in nursing research: concepts, procedures and measures to achieve trustworthiness. Nurse Educ Today.

[54] Diekelmann N, Allen D, Tanner C (1989). The NLN criteria for appraisal of baccalaureate programs: a critical hermeneutic analysis.

[55] Elzo J, Laespada MT, Pallarés J (2003). Más allá del botellón: análisis socioantropológico del consumo de alcohol en los adolescentes y jóvenes.

[56] McCabe MP, Ricciardelli LA (2004). Body image dissatisfaction among males across the lifespan: a review of past literature. J Psychosom Res.

[57] Kimmel MS, Hearn J, Connell RW (2005). Handbook of studies on men and masculinities.

[58] Özdemir E, Gültürk S (2008). Physiological and medical responses to anabolic androgenic steroids: review. J Med Sci.

[59] Choi PY, Pope HG, Olivardia R (2002). Muscle dysmorphia: a new syndrome in weightlifters. J Sports Med.

[60] de Visser RO, McDonnell EJ (2012). “That's OK. He's a guy”: a mixed-methods study of gender double-standards for alcohol use. Psychol Health.

[61] García-Calvente M, Marcos-Marcos J, del Río-Lozano M, Hidalgo-Ruzzante N, Maroto-Navarro G (2012). Embedded gender and social changes underpinning inequalities in health: An ethnographic insight into a local Spanish context. Soc Sci Med.

[62] Lyons A, Willott SA (2008). Alcohol consumption, gender identities and women's changing social positions. Sex Roles.

[63] EMCDDA – European Monitorin Centre for Drugs and Drug Addiction (2011). National report 2010: Spain. http://www.emcdda.europa.eu/html.cfm/index142386EN.html.

[64] Romo N, Marcos J, Rodríguez A, Cabrera A, Hernán M (2009). Girl Power: Risky Sexual Behaviour and Gender Identity amongst Young Spanish Recreational Drug Users. Sexualities.

[65] Deutsch FM (2007). Undoing gender. Gend Soc.

[66] Fagen J, Anderson PB (2012). Constructing masculinity in response to women's sexual advances. Arch Sex Behav.

[67] White A, Richardson N (2011). Gendered epidemiology: making men's health visible in epidemiological research. Public Health.

# Sub article: ‘Elastic band strategy’: women's lived experiences of coping with domestic violence in rural Indonesia

**DOI:** 10.3402/gha.v6i0.18894

**Published:** 2013-01-02

**Authors:** Elli Nur Hayati, Malin Eriksson, Mohammad Hakimi, Ulf Högberg, Maria Emmelin

**Affiliations:** 1 Faculty of Psychology, Ahmad Dahlan University, Yogyakarta, Indonesia; 2 Department of Public Health and Clinical Medicine, Epidemiology and Global Health, Umeå University, Umeå, Sweden; 3 Center for Health and Nutrition Research Laboratory, Gadjah Mada University, Yogyakarta, Indonesia; 4 Women's and Children's Health, Uppsala University, Uppsala, Sweden; 5 Department of Clinical Sciences, Social Medicine and Global Health, Lund University, Lund, Sweden

**Keywords:** domestic violence, coping, lived experience, Indonesia

## Abstract

**Background:**

Experiencing domestic violence is considered a chronic and stressful life event. A theoretical framework of coping strategies can be used to understand how women deal with domestic violence. Traditional values strongly influenced by religious teachings that interpret men as the leaders of women play an important role in the lives of Javanese women, where women are obliged to obey their husbands. Little is known about how sociocultural and psychosocial contexts influence the ways in which women cope with domestic violence.

**Objective:**

Our study aimed to deepen our understanding of how rural Javanese women cope with domestic violence. Our objective was to explore how the sociocultural context influences coping dynamics of women survivors of domestic violence in rural Purworejo.

**Design:**

A phenomenological approach was used to transform lived experiences into textual expressions of the coping dynamics of women survivors of domestic violence.

**Results:**

Experiencing chronic violence ruined the women's personal lives because of the associated physical, mental, psychosocial, and financial impairments. These chronic stressors led women to access external and internal resources to form coping strategies. Both external and internal factors prompted conflicting impulses to seek support, that is, to escape versus remain in the relationship. This strong tension led to a coping strategy that implied a long-term process of moving between actively opposing the violence and surrendering or tolerating the situation, resembling an elastic band that stretches in and out.

**Conclusions:**

Women survivors in Purworejo face a lack of institutional support and tend to have traditional beliefs that hamper their potential to stop the abuse. Although the women in this study were educated and economically independent, they still had difficulty mobilizing internal and external support to end the abuse, partly due to internalized gender norms.

In the early 1990s, violence against women became a focus of international attention and concern (1). It is now considered a major social and public health problem, as well as a human rights issue, in which governments have the right and obligation to intervene (2). Furthermore, the World Health Organization (WHO) as well as studies from other sites has concluded that domestic violence against women is a serious cause of physical and mental health impairment (3–7). The WHO multicounty study (4) not only confirmed the seriousness of domestic violence worldwide but also showed that the prevalence varied between countries. Lifetime and current physical violence ranged between 13–49% and 3–29%, respectively, whereas lifetime and current prevalence of sexual violence ranged between 6–50% and 1–29%, respectively. Studies around the globe have shown that multiple factors put women at risk of domestic violence, including the woman's and her partner's past history of violence, their current demographic, socioeconomic, and cultural situation, as well as their individual behavioral and relationship characteristics (4, 6, 8, 9).

Domestic violence against women refers to abusive acts that consist of three elements: boundaries of the relationship between the perpetrator and the abused, the norms of acceptable behavior, and the specific acts that constitute violence as the manifestation of subordination (10). In different cultural settings, women live in a wide variety of family structure arrangements. The violence that occurs is not limited to physical harm and may also be perpetrated by family members within the household other than the intimate partner. This definition acknowledges multiple explanations for the violence and could lead to more inclusive interventions that accommodate the experiences of all women (10).

Experiencing an abusive marriage is a complex phenomenon and is considered a chronic and stressful life event (11). Many studies have focused on identifying external factors that influence a woman's decision to leave or to stay in an abusive marriage (12–16). Others have emphasized the internal factors of female survivors (17–21) or focused on both external and internal factors (16, 21–24). However, the reasons why women decide to stay are less understood. A theoretical framework of coping strategies can be used to better understand how women manage the chronic stress associated with abuse. Coping is a cognitive and behavioral effort undertaken to manage the external and/or internal demands of a taxing situation (25). Thus, coping is a mechanism of overcoming a stressor, and this mechanism cannot be separated from what is called the coping resources because those resources directly determine the coping responses (21).

There are three important pairs of coping strategies described in the literature. The first is *problem-focused* and *emotion-focused* coping (25), the second is *approach* and *avoidance* coping (26) and the third is *engagement* and *disengagement* coping (27). The avoidance coping response is characterized by cognitive avoidance, resignation/acceptance, seeking alternative rewards, and emotional discharge (26). The approach coping response is characterized by logical analysis, positive reappraisal, seeking guidance/support, and problem solving. According to Tobin et al. (27), the concept of engagement/disengagement coping is a combination of the coping approaches proposed by Lazarus and Folkman (problem focused and emotional focused), where engagement coping includes problem solving, cognitive restructuring efforts, emotional expression, and social support, whereas disengagement includes problem avoidance, wishful thinking, self-criticism, and social withdrawal (27).

## Social context of women in Indonesia

The ideology of family harmony has been set as a priority by the Government of Indonesia, and women have been given a significant role in maintaining that harmony (28). Legally, the Marriage Law No. 1/1974 states that a husband and a wife have equal rights within the marital relationship, although husbands are stated to be the head of the household and wives are responsible for the household. Only in the last decade, under the new political reformation government was violence against women officially declared as a national problem. A Domestic Violence Act was endorsed in Indonesia in 2004. However, a national study conducted by Rifka Annisa (a non-government organization) in 2008 revealed that this Act had not been satisfactorily implemented and therefore not well understood by ordinary people (29).

## Domestic violence against Javanese women

Java is the main island of Indonesia, and Jakarta, the capital city of Indonesia, is located on this island. In Javanese tradition, women are constrained by the traditional feminine ideal that extols the virtues of submission and obedience. Javanese traditional values are strongly influenced by Islamic teachings that interpret men as the leaders of women and therefore require a woman to be obedient to her husband. Once married, a woman is bound to fulfill the socially prescribed roles of housekeeping, childbearing, and supporting her husband. The ideology of harmony is widely applied as a marriage norm and is referred to as *njaga praja*, meaning that the husband's honor must be protected from people outside the family (30). This spirit of harmony implies that conflicts and oppression within a family should not be discussed. Therefore, domestic violence often is hushed up, as acknowledging it would reveal a lack of harmony within both the family and the nation (31).

In a population-based study, using the WHO multicountry questionnaire, we found that the lifetime prevalence of physical and sexual violence among rural Javanese women in Purworejo District is 11 and 22%, respectively (5, 32). Of the women who had been physically abused, nearly 50% had told nobody about their experience of violence, while 33% had confided in their parents. The identified risk factors for experiencing lifetime violence included husband's age (>35 years), education (>9 years), and husband's psychosocial behavior, such as being unfaithful, drinking alcohol, fighting with other men, and having witnessed domestic violence as a child. From the women's side, being economically independent and having traditional views on gender norms were risk factors for domestic violence (32). However, little is known about how sociocultural and psychosocial contexts influence the interpretation of the meaning of abuse by the rural women of Purworejo and how that interpretation may influence their decision to take action. In this study, we aimed to deepen the understanding of how rural Javanese women cope with domestic violence, with a particular focus on violence perpetrated by the intimate partner. Focusing on rural women is important because they often have limited access to services, lower education, and more often live under socioeconomic constraints (33, 34). We hope that the study findings will enable policy improvements to increase the quality and accessibility of services for battered women in (rural) Indonesia in the future. Specifically, the aims of our study were to understand how the sociocultural context influences the coping dynamics of female survivors of domestic violence in rural Purworejo and to discuss the policy implications for care and prevention.

## Methods

### Study setting

We conducted the study in Purworejo District, Central Java Province, which is located 60 km west of Yogyakarta Province. According to the 2010 census (35), Purworejo District had a population of 695,427, with a total area of 1,035 km^2^, including coastal, lowland, highland, and hilly areas. Although urban centers are found in the district, 85% of the population lives in rural areas, with farming being the major occupation.

In 2000, the district government formally appointed the Office of Social Affairs and People Empowerment (OSAPE) to form a task force unit with the main function of providing a complaints desk for women survivors of domestic violence. Because of resource limitations, they only began documenting their activities in 2005. By the end of 2007, they had documented 27 domestic violence reports from women in the district.

### Study design

This study is based on a phenomenological approach, which is suitable for transforming lived experience into a textual expression of its essence (36). Phenomenology provides women survivors of domestic violence an opportunity to voice their own perspective of their life experiences (21). Within an empathetic interview, a woman survivor of violence is able to formulate her life history from the past to the present, and can explore, share, and validate her feelings and insights (21). Giving women survivors an opportunity to express their internal struggle in the face of abuse provides a valuable depiction of the development and use of inner resources that facilitate survival, strength, identity formation, and protection (20).

### Data collection

In-depth interviews were conducted with women survivors of domestic violence. The sampling technique used was criterion sampling, by which individuals who had been exposed to domestic violence and were willing and able to articulate their experiences were approached (37). In this study, we received information about possible informants (women survivors) from OSAPE. Only limited OSAPE staff members were available, one part-time and three full-time employees. In addition to other responsibilities, they were involved in counseling, referral, and outreach activities related to domestic violence. From the office, we were introduced to nine women who had been in contact with them for assistance. These women were all invited to participate in the study. After being informed of the research aims, seven women consented to be interviewed. The two that declined to be interviewed were not ready to discuss their domestic violence experience. All interviews were performed between December 2006 and August 2007, in places that the informants felt comfortable talking about their experiences. Two interviews took place at OSAPE, which some of the women were already familiar with. The other five interviews were conducted in other offices or in the informant's home. The interviews took between 1 and 1.5 hours, with only the woman survivor and the researcher present.

A semi-structured interview guide was developed and used during the interviews. The interview focused on the women's marriage history, the abuse experiences, efforts made to overcome the problems, social interactions, reactions and responses, the children's situation, and the women's reasoning for deciding to stay or to leave the marriage.

### Data analysis

The analysis followed a phenomenological guide developed by van Manen that combines features of descriptive and interpretative phenomenology (36, 38). According to van Manen, phenomenological inquiry methods cannot be formalized into a series of technical procedures; however, a variety of activities may fall into two types: empirical and reflective methods. Empirical inquiry activities aim to explore the range and varieties of pre-reflective experiential material that is appropriate for the phenomenon under study (39). Reflective inquiry activities aim to interpret the aspects of meaning or meaningfulness that are associated with this phenomenon. In this study, after reading the transcripts several times, we got a ‘sense’ of the data and a robust image was achieved. In the next step, we coded phrases, sentences, or statements that were significant for the purpose of this study (meaning units) and grasped the meaning of the identified meaning units by re-formulating them (descriptive level). Entering the reflective part of the analysis, we interpreted the descriptive formulated meanings and identified the subthemes (interpretative level). Finally, we clustered the subthemes into themes and integrated them into a comprehensive phenomenon (see Table 1).

**Table 1:** Data analysis structure (adapted from van Manen [34, 35])

Themes Sub themes	Reflective inquiry activities	Interpretative level
Formulated meaning unit		
Meaning unit	Empirical inquiry activities	Descriptive level
Transcript		
Interview with the respondent		

Table 2 below illustrates the analysis process and gives an example of how the subthemes emerged from the text.

**Table 2:** Data analysis examples

Empirical inquiry		Reflective inquiry
	
Meaning unit (from the transcripts)	Formulated meaning (descriptive analysis)	Subtheme (interpretative analysis)
I wore a Muslim veil because he wanted me to. I had to keep everything covered. I wasn't allowed to look beautiful and I wasn't allowed to get dressed up. As a wife I did what he wanted. If he told me what to do, I did what he said … the important thing to me was to maintain peace at home …	He directed her performance. She was obedient and complied with his orders to keep the marriage intact.	Being controlled on how to perform. Obedience and submissiveness as strategy to maintain the family harmony. Traditional belief about women's role in marriage.
		
I couldn't take it anymore so I told the head of the local Health Center when he asked, ‘Why are you always getting chicken pox?’ that actually they were really cigarette burns, not chicken pox.	She confessed during health attendance that she was burnt by his cigarette butts.	Self-disclosure during health attendance. Physically tortured.

### Ethical considerations

Ethical approval was obtained from the ethical review board at the Faculty of Medicine, Gadjah Mada University in Yogyakarta, and from the Purworejo District Government. Written consent was obtained from all participants prior to the interview. To protect confidentiality we used the codes S1–S7 to name our informants.

## Results

The seven women who consented to participate in the study had an average age of 39 years. Five of them had finished their bachelor's degree, while two had finished high school. The duration of marriage was 9–22 years and all had at least two children together with their abusive husband. Five women worked as civil servants at the government bodies in Purworejo, while two ran small shops (*warung*). Three women were divorced prior to the interview, while the other four were still living in an abusive marriage (see Table 3).

**Table 3:** Profile of the informants

Identity code	Age	Education	Occupation	Number of children	Types of violence experienced
S1 (divorced)	35 years	Bachelor degree	Primary health care staff	2	Physical, sexual, emotional & economic
S2	35 years	High school	Run a small shop at home	2	Physical, emotional & economic
S3 (divorced)	33 years	High school	Run a small shop at home	2	Physical, sexual, emotional & economic
S4	50 years	Bachelor degree	Civil servant (health district office)	2	Physical, emotional & economic
S5	43 years	Bachelor degree	Civil servant (teacher)	3	Physical, emotional & economic
S6	42 years	Bachelor degree	Civil servant (teacher)	3	Physical, sexual, emotional & economic
S7 (divorced)	38 years	Bachelor degree	Civil servant (sub district office)	2	Emotional & economic

Three women (S1, S3, and S6) suffered from all four forms of abuse (physical, sexual, emotional, and economic), three women (S2, S4, and S5) suffered from three forms of abuse (physical, emotional, and economic), and one women (S7) suffered from two forms of abuse (emotional and economic). The extent of abuse faced by these women was moderate to severe, with one woman suffering from severe injuries (S1).

Several of these women expressed feelings of being *financially abandoned*. S1 and S3 explained how they struggled to survive without enough economic support from their husbands and described the humiliating feelings this caused:

On about the 4th of March after the police force payday he came home after having disappeared for some days. He threw me three hundred rupiahs in front of my older sibling. I told him it wasn't enough … I asked for one hundred more. In front of the kids he said, “Go sell yourself … you should try it.” (S1)

S2, S4, S5, S6, and S7 also expressed their feelings about a strained financial situation, where they had to manage themselves to solve the financial needs within the household because of their husbands’ unfairness in sharing their income. Experiencing their husband stealing their savings or leaving no cash in the house for the family's daily needs were common problems for these women.

The women were exposed to various forms of *physical abuse* that caused minor as well as major injuries. S1 and S2 received major injuries when they were beaten, causing bleeding and fainting, while S3, S4, and S5 experienced mild to moderate physical abuse:

… every time he noticed me … put makeup on my face … when I prepared myself for the office, he made cynical comments … once he tried to remove the face powder from my face … he pushed me backward and roughly wiped a towel over my face to mess up my make-up … he did that because he was jealous, thinking I will have an affair with another man. (S5)

Jealousy caused physical abuse for S4, while S1 and S6 experienced physical abuse in addition to *sexual abuse*:

One night he woke me up and asked for sex … I woke up but was not ready to suddenly have sex with him … I mean … I need some time to prepare myself to have sex with him … but then he beat me up! It was just after midnight. His beating really made me awake, so I ran away from him and went into my kid's room. He said that wherever I run, I'm still his wife, so he might do anything to me! He did it several times … beat me … because of my refusal to his sexual requests. (S6)

Overall, the informants described a poor life situation and feelings of being destroyed by the abuse they experienced by their husbands.

Our analysis resulted in four main themes (italics) and six subthemes (bolded) that together illustrate the coping dynamics of these women survivors. First, the *ruined self* refers to the negative effects of domestic violence, that is, the overall loss of self-respect and confidence that the women had in common, even if they handled the situation in different ways. Second, the *inner realities* illustrate the dilemma the women felt when choosing between **fight to rescue** or **maintain the harmony**. Third, the *outer realities* show the influence of significant others and more formal institutions when functioning as **supporting actors** or **closed gates**. Fourth, the *elastic band strategy* demonstrates a coping strategy characterized by movement back and forth between **opposing** the violence or **surrendering** and accepting the situation. Below is a more detailed presentation of the results under the theme and the subtheme headings. Quotations from the interviews are given to illustrate how the informants own words have guided the interpretations.

### The ruined self

This theme describes the immediate effects as well as the long-term impacts of domestic violence on the person. The theme differs from the others in that it does not independently and on its own relate to coping. However, it describes the negative consequences of experiencing domestic violence, which thus creates the need to cope. Without ‘the ruined self’ there would be no need to cope. The women expressed a situation where they were totally disrespected, degraded, belittled, abandoned, and subordinated as human beings. Beatings, assaults, scolding, yelling, financial abandonment, hampers and control were overt expressions of their husbands’ power over them as wives. Because of this chronic abuse, these women suffered from immediate injuries as well as long-term negative impacts on their self-integrity. S1, S4, S5, and S6 described how they were accused by their husbands of having affairs with other men. This accusation had an almost fatal effect on S1's life, as that accusation was related to her husband's attempt to force her to sign divorce papers. As a result, S1 attempted to commit suicide:

Early in the morning, I was taken to hospital for two weeks. I was unconscious and on medication for a week. I took sleeping pills and poison used to exterminate pets. I felt like there was no way out. (S1)

Another of the informants, S4, developed gynecological problems because of her husband's adultery with a prostitute:

Last time I developed problems with my genitalia … painful and itchy like eczema down there … I showed it to him. I said, “You see, here is the bad thing again, from you! This must be caused by you and your prostitute!” (S4)

S5 never experienced any physical or sexual violence, but she suffered from emotional and economic abuse. However, she experienced physical symptoms because of her anger and frustration. Living in a marriage with chronic conflict had obvious effects on both her physical and mental health:

My only complaint is deep regret here (pointing her chest) … at the beginning I developed high blood pressure, and often got headaches. (S5)

S1, S3, and S4 expressed their concern about their children because they were also exposed to their father's abusive behavior toward their mother:

My eldest child was shocked to see me being beaten and bleeding. He immediately got a nosebleed … he was stressed. (S1)

Being a victim of domestic violence also caused negative psychosocial effects on these women's interactions with others. S6 lost her reputation after being slandered by her husband:

He accused me of having an extramarital affair, using my money to have fun with my extended family, and created a negative rumor about me among my colleagues. He directly came to the school principal and raised negative speculations that ruined my reputation. He said, “If something bad happened in the future at this school, don't blame me as I've already warned you about her.” So the next day the principal called me and cancelled my promotion for the master program. (S6)

The descriptions above clearly illustrate that domestic violence had a devastating impact on the women's physical and mental health, as well as their economic and psychosocial situation. Thus, experiences of ‘the ruined self’ led to a need to cope with this chronic stress.

### The outer realities: ‘supporting actors’ versus ‘the closed gates’

This theme and the subthemes describe how the informants accessed their outer resources to form their coping strategy. Apparently, these women oscillated between getting appropriate support and being denied either social or institutional support from their networks. Mostly, support came from family members and friends, but at times these social networks also behaved as ‘the closed gates’ because they did not give any sanction to the batterer. The formal actors, either affiliated to government or nongovernment organizations, also played a significant role, because women who had already successfully terminated their abusive marriage received institutional support, while those who were still in the relationship received nothing.

My big family, my father, mother, brothers and sisters were all on my side. (S7)

We were so poor that time, so my father kept sending me a monthly stipend because he was so worried. Life was very hard at that time. My husband always spent the stipend from my father. He tried to do a lot with the money, but nothing that he tried succeeded. (S4)

All of the children of the women in our study had witnessed their mother being abused by their father in various ways. Because they knew how their mother had been acting within the household, the children were on her side:

The older one … she shouted loudly at him one day as he treated me bad, she said to him, “What else do you want from Mama? What is her fault? Are you crazy or what?” He was so upset with her comment and yelled at her, calling her a sinful daughter for being that rough with her own father, “God will not grant you prosperity,” he said. She replied to him, “Prayers from a father such as you will be rejected by God.” (S4)

Besides support from significant others surrounding the women, S1 had received support from her husband's work place (employer). Her bravery in reporting her situation was followed up with her husband's employer intervening in her domestic violence problems:

I went to the head of the operational section, who was third in charge at the District Police Station. I said, “Sir, please help me … my husband is living with another woman.” He said, “Are you sure you are ready for this?” I said, “I'm ready … if possible sir, do something now … don't wait until tomorrow.” They immediately went in a car to arrest him. (S1)

S1 received a good response from her husband's institution. On the contrary, even though she often complained to OSAPE, she felt that this office could not really fight for her rights. S3 was pursuing her legal process in another city around 100 km north of Purworejo, where there was a nongovernment organization that had specific services for women survivors. In the end, she received tangible legal support and won her case. S7 was supported by her husband's office, which was committed to act because of his violation of the civil servants’ rules, that is, he married a second wife without consent from the first one.

However, some women informants were not defended by their immediate surroundings. S1 was in a big quarrel with her husband that ended in a fight, in which she was kicked and stomped in the abdomen and then expelled from the house along with her children. She asked one of the neighbors to mediate between them, but he refused to do anything to support her:

We slept at a mosque for three nights. Not even one person from my house helped us, not even one in the name of God. They saw me taking my things so they knew. I took clothes and a bag from the house. In Surorejo Village … no one helped me. (S1)

S2 was hit and kicked inside the house, and she screamed for help. Her mother came to help, while the neighbors did not do anything:

He hit and kicked me so hard that I collapsed … then he banged the door and went out … I screamed and my kid cried a lot … so my mother came and helped me, and the neighbors came over and asked my husband what had happened but he kept silent. And then he went to his uncle's house … just like that. (S2)

S6 received some material and financial support from her family, but no one saved her from her husband's beatings. Meanwhile, S5 complained to her mother-in-law about her husband's lack of contribution in financing the household, but her mother-in-law neglected her:

He had a job and projects, but he did not share his earnings for running the household. And when I complained to my mother-in-law, she said, “Why should you complain, don't you have your own job? Can't you can manage your life with the salary that you get?” (S5)

These descriptions illustrate that the outer realities, either individual or institutional, consisted of supportive actors as well as closed gates. The most reliable support came from family, while neighbors were less supportive and even tended to be ignorant toward domestic violence in the neighborhood. Concerning institutional responses, the experiences of S1, S3, and S7 of receiving institutional support showed the significant impact that this type of support could have. For these women, the institutional support gave them the strength to cope, and became a turning point, leading to the decision to leave their abusive husbands.

### The inner realities: fight to rescue versus maintain the harmony

This theme illustrates the internal/inner realities that influenced how the women coped with the domestic violence they experienced. On the one hand, their internal resources reflected a powerful self that was capable of fighting and rescuing themselves and their children. On the other hand, their inner resources also reflected a powerless self, influenced by internalized norms that require them to be a good wife and mother by maintaining the family harmony. A powerful self among these women was shown in their assertiveness and self-authority over their daily life:

The thing that drove me the most to divorce was that I wanted it to end it rather than continuing to live a life like that. I felt worried about the children, and secondly, I didn't want to live as a woman who is constantly being put down and belittled … I wanted the divorce. I wasn't afraid of the future. (S3)

Assertiveness was a quality that was possessed by all women in this study, and it was expressed differently according to personal style. This is actually a good modality for women to raise their voice within an intimate relationship.

Another quality that reflected a powerful self among these women was good self-regulation, which indicates a personal maturation among them:

One night he came home very late, and the next morning I still talked to him in a very pleasant manner … although he answered in a rough way … I talked to him politely, asked what he needed and wanted … I did that to protect the kids from imitating their father's rough behavior. (S7)

Another quality of these women that reflected a powerful self was self-confidence in maintaining their work performance outside the house. As we know, five out of seven women in this study were working at government bodies, and they all managed to perform their work well:

He is a civil servant just like me, but I think I had a better career than him at the time. I did many papers for different presentations, and got many points for that, while he didn't. So, actually I was more advanced than him in terms of career. I am a very independent person. (S5)

These women had good self-confidence, which could have functioned as an inner resource for managing domestic abuse. However, experiencing domestic abuse had weakened this powerful self, in favor of the more powerless self. The significance of the powerless self among these women was reflected in their beliefs about how to behave as a good wife in maintaining and keeping their marriage intact. Because of the internalized norms about being a good wife, they almost had no self-authority over their life:

“Why should you live like this? Why should you?” my father asked, and he asked me to move to his town several times, but it's so strange that I did not dare to do so … so strange that I always confirmed his (husband's) words, did what he asked, and had no bravery to act against him. (S4)

S5 and S6 expressed their powerlessness by waiting and asking their abusive husband to initiate a separation, which obviously would never happen:

That is what I feel … with everything he's done to me … he hurt me, betrayed me … I would very much indeed want to be away from him … divorce … but it's supposed to be his initiative … not mine. (S6)

S2 even denied that arguing and hitting are bad in a marriage, and therefore she did not really mind what her husband did to her:

Yeah … he did this to my head (pushing her head with her hand) … but it didn't really hurt. He always did that to my head. He always did it to the kids too. I think that it's just normal arguing between husband and wife. I think arguing is normal. (S2)

The next aspect that became an internal struggle to cope with domestic violence in this study was their concern about the children. S2 and S5 shared the experience of thinking about separation, but every time they started thinking about their children, they felt a deep concern about them:

That is exactly what is in my mind … if I leave him, I may be free and become a free woman … but what about my kids? (S5)

S1 and S6 were convinced that domestic violence had bad consequences for their children, and that is why they were concerned about how to protect their children from further exposure to the marital abuse:

At night I get scared that he's going to come and take the kids … I go and check my kids and just hold them both … I say, “Oh they are still here!” They are the most valuable thing to me. Let me be poor, but I don't want to lose my kids … I was stupefied by their father. I don't want that again … don't want him to meet with the little ones … he's not allowed to. (S1)

The above quotes describe how concern for their children pushed them from one side to another. Their concern for their children apparently strengthened them to fight and rescue both themselves and their children. However, at the same time, it weakened their inner power because of the internalized norms about their responsibility to maintain family harmony.

### The ‘elastic band strategy’: opposing the violence versus surrender and stay

This theme describes how the women in this setting coped with domestic violence. Analyzing their stories showed a situation whereby the women seemed to cope using what we have labeled the ‘elastic band strategy.’ This strategy implied a constant stretching, by making efforts to oppose the violence, for example, through spiritual framing, seeking outside support, being assertive, and trying to make a positive diversion. However, the stretching was often followed by withdrawal and surrender through submissiveness, keeping silent, or ignoring their husband's behavior.

An example of how women coped by being submissive is illustrated below:

I wore a Muslim veil because he wanted me to. I had to keep everything covered. I wasn't allowed to look beautiful and I wasn't allowed to get dressed up. As a wife, I did what he wanted. If he told me what to do, I did what he said … the important thing to me was to maintain peace at home. (S1)

S2 was also abused by her in-laws, as she lived in the same house as them. For her, being submissive was the most effective way to prevent further conflict with her husband and in-laws. A submissive act was usually followed by an act of silence, as expressed by S5:

My youngest kid saw it not so long ago … she said, “Mom, what happened?” I said to her, “He (husband) just broke down a door again.” But my neighbor never heard anything, because I always kept quiet about whatever he was mad about in our home. (S5)

Most of these women had noticed some strange behavior by their husband at an early stage of their marriage, but had ignored it:

I moved to another town with the kids, he was supposed to would follow later on. One day, I telephoned his neighbors and asked how he was. The neighbor said, “He does not live in the house any more … Oh, you didn't know that? He's living somewhere else now with another woman. You need to come here!” But I closed my eyes and ears. I thought, yeah … maybe it could happen if he was far from his wife, but I was sure that he wouldn't be living with another woman. I didn't believe it. (S1)

All informants described how they had ignored their husband's violent tendencies at the beginning of their marriage, but later they became aware that this did not improve their situation. These women used different methods to oppose their abusive husbands and thereby end the abuse.

Two women, S1 and S4, described how they framed their life in a spiritual way, something that is commonly practiced by people in this area:

I said that God never sleeps … I prayed … during the night, I just prayed, asking God to help me, to bring the truth to light. (S1)

Some of the women in this study undertook the brave action of initiating a report of their husband to the authorities. S1, S3, and S4 reported their husband's beatings to the police:

It was painful when he pushed me … my belly got hurt … I made a report to the police right away. (S3)

Other than making a legal report, some of the women were also active in seeking additional financial support:

I asked for assistance from the Office of Social Welfare and got some money to start a small business. Otherwise, where would the money come from for telephone, electricity and water? (S3)

Another way for these women to cope was to disclose the abuse to family or friends. All women in this study, except S5, eventually disclosed their abusive experience to family or friends:

Finally, after 10 days of keeping the secret to myself, I could not bear it any longer and I cried to my friend. (S7)

S5 undertook another brave action. She felt so frustrated by her husband's violent behavior, and she thought about how she could achieve a better future life by executing a positive diversion:

I became more and more frustrated to see what he did over and over again … and then I started to think about how to change my life into a happier one … I started to prepare myself for higher education, so that in the long run I will have a better income and a stable career. (S5)

Overall, the coping dynamics among the women in this study showed a complex pattern where the outer and inner realities could simultaneously act as factors that facilitate fighting against the violence and also as factors making the women surrender and tolerate the violence.

Figure 1 depicts how the women's experiences of chronic abuse (stressors) have ruined their personal life because of the associated physical, mental, psychosocial, and financial impairment. These chronic stressors led the women to evaluate and access their external and internal resources to form coping strategies. Apparently, both external and internal resources contained conflicting realities. Within their external resources, they found relatively good support from friends and family members, and from their husband's employers. However, at the same time, they received less support from their neighbors or the police. From their internal resources, these women also experienced a conflicting situation. They felt a need and a responsibility to rescue themselves and their children, which conflicted with the internalized norm of their role in maintaining family harmony by being an obedient wife and a good mother. This strong tension led to the *elastic band strategy*, which implies a long-term process of moving between actively opposing the violence and surrendering and abiding the situation a bit longer, before possibly, eventually leaving the relationship.

**Fig. 1:** Coping dynamics of women survivors of domestic violence in rural Purworejo.

## Discussion

All women in this study suffered from moderate to severe forms of abuse from their partners, which resulted in a decision to either terminate the marriage through divorce or stay in the relationship but struggle to cope with the abuse. The overall strategy was described as *an elastic band* that stretches in and out to adjust to the immediate needs related to the violent episodes. The informants’ lived experience shows that coping behavior is a result of a complex interaction between the availability of inner and outer resources that in turn guide their decision to stay or to leave the marriage.

This study shows that the women exposed to violence felt that they received *appropriate social support from family or friends* when they asked for it but *lacked support from neighbors*. The importance of this support has been emphasized by others (14, 21, 40). Even if it often only temporarily slows the abuse, social support is viewed as important for improving the coping capacity of women survivors of partner abuse (40). Having a trusted colleague available to discuss one's problems with has also been shown to be beneficial (41). Other studies have found that battered women tend to have low levels of perceived social support (22, 40). This might be because of the common norm of viewing domestic violence as a private issue in which outsiders should not intervene. In our study setting, traditional values, shared by most Javanese, state that once a woman marries a man, she belongs to him, and the parents no longer have power over her. Our previous findings (5) have shown that the majority of women in this study setting (94%) agreed to the statement ‘Family problems should not be discussed with people outside the family.’ On the other hand, more than half of the women (59%) agreed that in the case of violence, ‘Others outside the family should intervene.’

The women in this study lacked proper *institutional support* from the Police Department, legal institutions, or other related government bodies. Only one of the women who had reported their husband to the police was followed up seriously. However, the women who decided to leave their abusive husbands felt that they received tangible support from the government bodies that their husbands were affiliated to or from human rights organizations (nongovernment organizations). Receiving positive responses from organizational bodies gave these women confidence and a feeling of being able to change their situation. This means that support from formal institutions may significantly contribute to encouraging women who have decided to end an abusive marriage. This is in line with the framework proposed by Liang et al. (14) about the leaving process among survivors of intimate partner violence, and findings from the study of Koepsell et al. (15), where women who left the relationship were significantly more likely to have successfully accessed services from domestic violence agencies or other public or private assistance, compared with those that had not left the relationship.

Internally, women fought against the internalized norm that a woman's role is about being a good wife and a good mother (42). Thus, in a society that places the burden of family harmony on the woman, a failed marriage must be her fault. This suggests that a commitment to the relationship may be a significant factor in the decision to tolerate abuse (43). In this study setting, it is common that brides are advised by their parents to carefully hide conflict and to protect the husband's honor from people outside the family. Accordingly, in our study, we found that the powerful self of these women, what Patzel (19) calls *self-efficacy*, was counteracted by rural isolation and cultural values that emphasized family unity (43). The women in our study were thus faced with an internal conflict that hampered their ability to optimally utilize their powerful self. Therefore, this inner conflict is an important internal factor that must be taken into account in the scheme of women's empowerment efforts, in terms of enhancing their coping skills.

Overall, the coping responses described by Moos (26) and Tobin et al. (27) resemble the coping strategies used by the women in this study. They used different coping strategies that changed over time, repeatedly oscillating between avoidance or disengagement to approaching or engaging. This fits with the findings of Waldrop and Resick (23), who claim that within an abusive relationship, a woman may prefer to use a certain coping strategy, but then she must adjust that strategy to fit to a particular situation. This is because women who experience domestic violence most likely face a lack of available resources as well as social support, options for escape, and control (44). The use of approach or engagement coping responses to counter the abuse led some of the women to be in a strong position to take action to leave the abusive husband, but, later on, that strategy also led them to face retaliation from their husband. Goodman et al. (44) have shown that active coping actions (approach and engagement responses) have the potential to positively affect the elimination of violence in a woman's life but at the same time have negative consequences. This is similar to what we found in our study. When women actively sought institutional support from the police, it stopped their husband's physical abuse but also led to other psychosocial problems such as a lack of financial support for the household. The dynamics of the abusive relationship and the overall availability of internal and external resources formed an elastic band strategy among the women survivors of domestic violence. This coping strategy will lead to further decisions about whether to stay or to leave the marriage.

### Validity

Using a phenomenological approach means that the researcher builds on people's experiences of a certain phenomenon to become more experienced themselves (39). The need for validating the findings by discussing the transcripts with the informants themselves is strongly emphasized (45, 46) and is similar to what Lincoln and Guba (47) call member checking. In this study, it was only possible to perform member checking with two of the informants. However, the prolonged engagement by the first author in the field of domestic violence (being a psychologist) as well as her frequent field visits to the study site increases the validity of the study. During the analysis, regular peer debriefing sessions were held within the research group to broaden the perspective and discuss the interpretation of the data.

### Limitations of the study

The study limitations refer mainly to the sampling of informants. All the interviewed women had accessed institutional services and they all had more than 9 years of education. This means that we do not know if the described coping mechanisms apply also to less educated women who have not sought support. These groups may be more prone to accept their situation and remain in an abusive relationship.

## Conclusion and policy implications

In this setting, domestic violence is still seen as a private and internal issue in which people outside the family are not supposed to intervene. Therefore women survivors of violence face a lack of institutional support and tend to have traditional beliefs that hamper their potential to stop the abuse. The women in our study had a fairly high educational level and had their own jobs; yet, they had difficulties in mobilizing the support needed to end the abuse. This could partly be explained by internalized gender norms about their responsibility for maintaining family harmony.

Women in this rural area face many disadvantages in life, including experiencing domestic violence, which is partly accepted by patriarchal gender norms. We encourage the government of Purworejo to:

- Conduct early prevention efforts through various public educational strategies to challenge the traditional norms, gender equity, and the unfair burden of gender-based violence in the community, among men and women, boys and girls.
- Establish a responsive law enforcement system so that all women survivors’ reports are followed up appropriately along with the Indonesia Domestic Violence Act (Law number 23/2004).
- Provide consistent resources such as funds for the daily management of the service unit and capacity building for the staff. This effort will guarantee the existence of the service unit in providing any service for women survivors of violence in the district.
- Improve their outreach service programs for women in remote areas by providing trained lay counselors at the community level. These lay counselors will be acting as the first care taker for further referral and ‘the first aid’ provider for women survivors before they undergo further referral to the unit.

Finally, the Government of Purworejo must take into account the specific needs of its inhabitants and understand that maintaining family harmony at any cost may threaten the lives of women and children in their area.

## References

[1] Population Reports (1999). Ending violence against women. Population Information Program, Center for Communication Programs.

[2] Ellsberg MC (2000). Candies in hell: research and action on domestic violence against women in Nicaragua.

[3] Heise LL, Raikes A, Watts CH, Zwi AB (1994). Violence against women: a neglected public health issue in less developed countries. Soc Sci Med.

[4] World Health Organization (2005). Multi country study on women's health and domestic violence against women: initial result on prevalence, health outcomes and women's response.

[5] Hakimi M, Hayati EN, Marlinawati VU, Winkvist A, Ellsberg MC (2001). Silence for the sake of harmony: domestic violence and health in Central Java.

[6] Campbell J (2002). Health consequences of intimate partner violence. Lancet.

[7] Ceballo R, Ramirez C, Castillo M, Cabalerro GA, Lozof B (2004). Domestic violence and women's mental health in Chile. Psychol Women Q.

[8] Xu X, Zhu F, O'Campo P, Koenig MA, Mock V, Campbell J (2005). Prevalence of and risk factors for intimate partner violence in China. Am J Public Health.

[9] Abramsky T, Watts CH, Garcia-Moreno C, Devries K, Kiss L, Ellsberg M (2011). What factors are associated with recent intimate partner violence? Findings from the WHO multi-country study on women's health and domestic violence. BMC Public Health.

[10] PROWID (1999). Domestic violence in India: summary report of three studies.

[11] Gelles RJ (1976). Abused wife: why do they stay. J Marriage Fam.

[12] Barnett OW (2000). Why battered women do not leave, part I: external inhibiting factors within society. Trauma Violence Abuse.

[13] Barnett OW (2001). Why battered women do not leave, part 2: external inhibiting factors – social support and internal inhibiting factors. Trauma Violence Abuse.

[14] Liang B, Goodman L, Tummala-Nara P, Weintraub S (2005). Theoretical framework for understanding processes among survivors of intimate partner violence. Am J Community Psychol.

[15] Koepsell JK, Kernic MA, Holt FL (2006). Factors that influence battered women to leave their abusive relationship. Violence Vict.

[16] Kanazawa S (2008). Battered women have more sons: possible evolutionary reasons why some battered women stay. J Evol Psychol.

[17] Strube MJ, Barbour LS (1983). The decision to leave an abusive relationship: economic dependence and psychological commitment. J Marriage Fam.

[18] Eisikovits Z, Buchbinder E, Mor M (1998). “What it was won't be anymore”: reaching the turning point in coping with intimate partner violence. Affilia.

[19] Patzel B (2001). Women's use of resources in leaving abusive relationship: a naturalistic inquiry. Issues Ment Health Nurs.

[20] Davies RE (2002). The strongest women: exploration of the inner resources of abused women. Qual Health Res.

[21] Davies RE (2002). Leave-taking experiences in the lives of abused women. Clin Nurs Res.

[22] Mitchell RE, Hodson CA (1983). Coping with domestic violence: social support and psychological health among battered women. Am J Community Psychol.

[23] Waldrop AE, Resick PA (2004). Coping among adult victims of domestic violence. J Fam Violence.

[24] Herbert TB, Silver RC, Ellard JH (1991). Coping with abusive relationship: I. How and why do women stay?. J Marriage Fam.

[25] Lazarus RS, Folkman S (1984). Stress, appraisal and coping.

[26] Moos RH (1995). Development and applications of life stressors, social resources and coping response. Eur J Psychol Assess.

[27] Tobin DL, Halroyd KA, Reynolds RV, Wigal JK (1989). The hierarchical factor structure of coping strategy inventory. Cognit Ther Res.

[28] Sitepu HS, Bianpoen C, Oey Gardiner M (2000). Queen of the household: an empty title. Indonesian women: the journey continues.

[29] Hasyim N, Kurniawan AP, Hayati EN (2008). Report on the monitoring of the implementation of Indonesia domestic violence act in six provinces.

[30] Djohan E (1999). Women's live in Javanese family (ideology and reality).

[31] Sciortino R, Smith I (1997). Kemenangan harmoni: pengingkaran kekerasan domestik di Jawa. Jurnal Perempuan.

[32] Hayati EN, Hogberg U, Hakimi M, Ellsberg M, Emmelin M (2011). Behind the silence of harmony: risk factor for physical and sexual violence among women in rural Indonesia. BMC Womens Health.

[33] Logan TK, Walker R, Cole J, Ratliff S, Leukefel C (2003). Qualitative differences among urban and rural violence victimization experiences and consequences: a pilot study. J Fam Violence.

[34] Grosman SF, Hinkley S, Kawalski A, Margrave C (2005). Rural versus urban victim of violence: the interplay of race and region. J Fam Violence.

[35] Indonesia Statistic Central Bureau (2012). Penduduk kabupaten purworejo hasil sensus penduduk tahun 2010. BPS Purworejo.

[36] Van Manen M (1997). Researching lived experience.

[37] Creswell JW (1998). Qualitative inquiry and research design: choosing among five traditions.

[38] Cohen MZ, Omery A, Morse JM (1994). Schools of phenomenology: implications for research. Critical issues in qualitative research methods.

[39] Van Manen M Phenomenological online: resource for phenomenological inquiry. http://www.phenomenologyonline.com.

[40] Barnett OW, Martinez ET, Keyson M (1996). The relationship between violence, social support and self-blame in battered women. J Interpers Violence.

[41] Cohen S, Hoberman HM (1983). Positive events and social supports as buffers of life change stress. J Appl Soc Psychol.

[42] Strauss M, Gelles RJ, Steinmetz SK (1980). Violence in the American family: behind closed doors.

[43] Hassouneh-Phillips D (2001). American Muslim women's experiences of leaving abusive relationship. Health Care Women Int.

[44] Goodman LA, Smyth KF, Borges AM, Singer R (2009). When crisis collide: how intimate partner violence and poverty intersect to shape women's mental health and coping?. Trauma Violence Abuse.

[45] Caelli K (2001). Engaging with phenomenology: is it more of a challenge than it needs to be?. Qual Health Res.

[46] Barkway P (2001). Michael Crotty and nursing phenomenology: criticism or critique?. Nurs Inquiry.

[47] Lincoln YS, Guba EG (1985). Naturalistic inquiry.

# Sub article: ‘Expanding your mind’: the process of constructing gender-equitable masculinities in young Nicaraguan men participating in reproductive health or gender training programs

**DOI:** 10.3402/gha.v5i0.17262

**Published:** 2012-08-02

**Authors:** Virgilio Mariano Salazar Torres, Isabel Goicolea, Kerstin Edin, Ann Öhman

**Affiliations:** 1 Center for Demography and Health Research, Nicaraguan National Autonomous University, León, Nicaragua; 2 Umeå Center for Global Health Research, Umeå University, Umeå, Sweden; 3 Umeå Center for Gender Studies, Umeå University, Umeå, Sweden

**Keywords:** gender, grounded theory, masculinity, Nicaragua, young men, change

## Abstract

**Background:**

Traditional forms of masculinity strongly influence men's and women's wellbeing.

**Objective:**

This study has two aims: (i) to explore notions of various forms of masculinities in young Nicaraguan men participating in programs addressing sexual health, reproductive health, and/or gender equality and (ii) to find out how these young men perceive their involvement in actions aimed at reducing violence against women (VAW).

**Design:**

A qualitative grounded theory study. Data were collected through six focus groups and two in-depth interviews with altogether 62 young men.

**Results:**

Our analysis showed that the informants experienced a process of change, labeled ‘Expanding your mind’, in which we identified four interrelated subcategories: The apprentice, The responsible/respectful man, The proactive peer educator, and ‘The feminist man’. The process showed how an increased awareness of gender inequities facilitated the emergence of values (respect and responsibility) and behavior (thoughtful action) that contributed to increase the informant's critical thinking and agency at individual, social, and political levels. The process was influenced by individual and external factors.

**Conclusions:**

Multiple progressive masculinities can emerge from programs challenging patriarchy in this Latin American setting. The masculinities identified in this study show a range of attitudes and behaviors; however, all lean toward more equitable gender relations. The results suggest that learning about sexual and reproductive health does not directly imply developing more gender-equitable attitudes and behaviors or a greater willingness to prevent VAW. It is paramount that interventions to challenge machismo in this setting continue and are expanded to reach more young men.

This article focuses on emerging forms of masculinities that will hopefully improve health for both men and women. Gender can be understood as a set of social relations that organize our social practice and interactions between men and women in a given culture (1, 2). However, these interactions are often inequitable, defined by power imbalances between and within genders (1). In many societies, these inequalities are representations of a patriarchal system of beliefs giving men authority over women (3). Nevertheless, not all men have the same amount of power in a given society. Connell (2) proposes that there are different types of masculinities, which are related to each other in diverse ways (relations of hegemony, subordination, complicity, and marginalization). Among these, hegemonic masculinity emerges as a key concept. It is defined as ‘the configuration of gender practice which embodies the currently accepted answer to the problem of the legitimacy of patriarchy, which guarantees the dominant position of men and the subordination of women’ (2). In other words, it represents the ideal of what it is to be a man in a given society. However, it is not a static entity: it changes over time and is continuously challenged by men and women who oppose it (4).

Although the concept of hegemonic masculinity has been used extensively in many research fields, it has also been criticized. Demetriou (5) and Hirose and Kei-ho Pih (6) have pointed out that the construction of hegemonic masculinities can also be influenced by features of subordinated and marginalized masculinities reinforcing its domination. Howson has criticized Connell's theory for lacking an emphasis on how ambivalent and protest femininities influence gender politics (7). In addition, even if it has been argued that Western constructions of masculinities have a strong influence on their local and regional counterparts in other parts of the world (8), regional and local hegemonic forms of masculinity are also shaped by other factors, such as local traditions and migration (4, 9–11). One recent criticism of approaching masculinity as a plural entity is that such an approach focuses too much attention on the differing rather than the common practices that men share (12). Other authors have questioned the clarity of Connell's theory, pointing to its ambiguous and sometimes blurred definition (13–15). Nevertheless, we agree with Connell in her acknowledgement of these criticisms and reformulation of the hegemonic masculinity concept, stressing the relevance of change, power relations, and multiplicity in the study of masculinities (4).

## Masculinities in Latin America

In Latin America, machismo has historically been viewed as a set of hegemonic masculinities that legitimizes patriarchy in this setting (16, 17). Rather than a single set of defined behaviors, several authors (17–20) have proposed that machismo can be expressed differently in different men, with their behavior oscillating within a continuum of positive and negative characteristics. In a quantitative study of Latino men living in the United States, five different types of machismo were identified: contemporary masculinity, machismo, traditional machismo, compassionate machismo, and contemporary machismo (18). These types differed in key characteristics such as whether or not they were authoritarian, the degree of demands regarding women's obedience, different levels of competitiveness, and, most important, different degrees of flexibility regarding traditional gender relations.

In a review of studies exploring masculinities in Latin America, Gutmann (21) also highlights the multiplicity of masculinities in this region. This diversity has been documented in Mexico by Ramirez Rodriguez (22), who found that young men's attitudes toward gender relations can range from conservative to ambiguous to flexible. Gutmann (21) proposes that Latin American masculinities have been in a process of change, suggesting that these transitions have been influenced by global economic changes that have led to increasing modernization and urbanization, new job markets for women, and a growing feminist activism in the region. These changing patterns of masculinity may also have been influenced by ongoing interventions in a number of Latin American countries, such as Program H in Brazil, which have been actively promoting more gender-equitable forms of masculinities (23–26).

## Why is it important to challenge hegemonic masculinities?

Young people's health is often influenced by gender inequalities – one example is access to contraception – and these are reinforced by unequal gender relations present in hegemonic masculinities. Thus, challenging hegemony and hence the gender order might improve the health of young people. The World Health Organization defines gender equality as the ‘the absence of discrimination – on the basis of a person's sex – in providing opportunities, in allocating resources and benefits or in access to services’ (27). Achieving gender equity – ‘fairness and justice in the distribution of benefits and responsibilities between women and men’ (27) – is a critical step in the process of attaining gender equality. It has been argued that men are the main guarantors of the patriarchal gender order (28); thus, several researchers have pointed out that interventions aiming to achieve gender equity must involve men in order to be more successful (24, 28, 29). Around the world, different cultural constructions of masculinities have been associated with men's unhealthy behaviors, such as aggression and risk taking, leading to a higher risk of morbidity and mortality over men's life course (30, 31). Young men often feel pressured by society (32, 33) to engage in these behaviors to prove their masculinity to themselves and their peers (30, 34–36). Women's health is affected by men's negative behaviors. One significant example of this is men's violence against women (VAW), a common expression of male power and control in patriarchal societies (37). VAW is a widespread public health problem affecting women throughout their life span (38). Recent population-based data suggest that one form of VAW – intimate partner violence (IPV) – is a transcultural phenomenon, including physical, sexual, and psychological violence and affecting women and families worldwide, especially those in low-income countries (39). Exposure to IPV has consistently been associated with women's poor mental, physical, and reproductive health (40, 41).

## Rationale for this study

In Nicaragua, studies have found evidence of changing gender norms in both women (42) and men (43, 44). In a qualitative study of adult men, Sternberg et al. (43) reported that men had different discourses about masculinity, ranging from a traditional patriarchal stance to a feminist discourse. Welsh (44) proposed that men's attitudes toward more equal gender relations might be linked to their participation in activities aimed at challenging patriarchy in this setting. Quantitative and qualitative studies describing the changes that adult and young men experienced while engaging with institutions promoting changes in masculinity patterns have been conducted in Latin America and around the world (21, 26, 43–46). However, to the best of our knowledge, no studies have explored in full how these changes happened, what influenced the flow of these pathways of change, and what forms of masculinities emerged from this process. The purpose of the present paper is therefore twofold: (i) to explore notions of various forms of masculinities among young Nicaraguan men participating in training programs addressing sexual health, reproductive health, and/or gender equality and (ii) to find out how these young men perceive their participation and involvement in actions aimed at reducing VAW.

## Methods

### Setting

The study was performed in Managua and León municipalities, Nicaragua. Nicaragua is a low-income country with high unemployment. As in other low-income countries, young people face many challenges, with poverty and lack of educational opportunities the most prominent. National data show that only 39% of those attending high school complete their education (47). In addition, both the public and the private school system lack a scientific and progressive sex education program.

The hegemonic pattern of masculinity in Nicaragua is *machismo*. Machismo is understood as the set of attitudes and behaviors that dictate how men interact with women and with other men. According to Welsh et al. (48) and Lancaster (49), machismo is based on the notion that men are superior to women and that men are tough, violent, domineering, and womanizers. Conversely, *marianismo* is a form of emphasized femininity that reinforces machismo. It promotes the ideal that ‘real women’ are docile and compliant to men's patriarchal privileges (16). Several studies conducted in Nicaragua have shown that machismo strongly influences young men's behaviors, increasing health risks both to themselves and their partners (44, 50). For example, quantitative studies have shown that young Nicaraguan men, despite growing knowledge of how HIV can be transmitted, use condoms inconsistently and mainly with occasional partners (51, 52), leaving their stable partners unprotected against the possible risk of sexually transmitted diseases and HIV. Traditional machismo behavior includes promiscuity and has been associated with their female partner's higher risk of developing high grade cervical lesions (53).

The Nicaraguan government has an ambiguous position on gender and sexual and reproductive rights. Although it has increased the availability of resources to diminish maternal mortality, access to therapeutic abortions was criminalized for the first time in the last 100 years. In addition, despite the fact that Nicaragua is a signatory of The Convention on the Elimination of all Forms of Discrimination against Women, changes in the country's legislation and special units at police stations to handle VAW, it continues to be a serious public health issue. According to the most recent national survey, 21% of women reported being exposed to emotional, physical, or sexual abuse by their partners in the past year (54). In addition, a recent study found that 15% of ever-pregnant Nicaraguan women had experienced physical abuse in the past year and 24% reported a continuous pattern of abuse (42).

Population-based studies have been conducted, among women but not among men, assessing their attitudes to gender relations and VAW (42, 54). A recent 4-year panel study showed that there has been a significant change in women's attitudes toward VAW and gender relations. Nicaraguan women have become less tolerant of partner abuse and less supportive of traditional gender relations (42). In addition, a qualitative study among men found that feminist and patriarchal discourses about masculinity coexist in Nicaraguan society (43).

Thus, machismo behaviors and traditional norms have not gone unchallenged in Nicaragua. Although the government has not implemented any systematic counter-machismo programs, over the last 20 years, the strong civil society of Nicaragua and its non-governmental organizations (NGOs), such as Cantera (24, 44), CISAS (45), and Puntos de Encuentro (55) among others, have implemented grass-roots and population-based interventions aiming to challenge and change patriarchal behaviors and values among young people. Many of these interventions have been implemented from a feminist perspective (44). One recent example is a community-based intervention directed at young people and conducted by the NGO Puntos de Encuentro. This intervention included training workshops, educational materials, and mass media activities (a radio show and a soap opera). The activities aimed to challenge norms about HIV, masculinity, and VAW. The soap opera was broadcasted on national TV (55).

### Data collection and participants

We gathered the data through six focus group discussions (FGDs) and two in-depth interviews. The sampling was purposive in accordance with the flow of the emerging concepts. We contacted the participants through NGOs working with young men in two of the main Nicaraguan cities (Managua and León). The NGOs were in general addressing broad topics within a gender, VAW, reproductive, and sexual health framework. Their activities were heterogeneous and varied from group education, community outreach, and mobilization to a combination of these methods. The activities seemed to depend on the NGOs’ own agendas (24).

Three focus groups were conducted in Managua and three in León. The participants in each group belonged to the same NGO and thus knew one another. The facilitator encouraged them to discuss with each other rather than directly answering the questions. The focus groups had on average 10 participants and met in a private room to avoid outside disturbance and ensure confidentiality. As part of the emergent design, during the analysis and interpretation of the data, we also performed two in-depth interviews. In the course of the analysis of the FGDs, we started to identify the different masculinities emerging from the data and how they related to each other. The in-depth interviews allowed us to confirm and deepen our understanding of the differences and similarities between the masculinities identified from the FGDs, as well as the ways these masculinities were constructed.

In total, we gathered data from 62 young men who were participating voluntarily in NGO training programs. Their involvement with the NGO programs occurred as follows: some were recruited directly by the NGO's facilitators in the neighborhoods where the interventions were taking place, while others had been invited by friends who were already members. The young men expressed various motives for joining an NGO. Most wanted to learn more about sexual and reproductive health, some wanted to spend time with their friends, and others were curious about the NGO's activities. The participants’ age ranged from 17 to 24 years and their educational level from incomplete high school to some university education. Most of the participants were studying and some were working in the informal sector.

The participants’ length of involvement with an NGO varied from 1 to 4 years. During this period, their exposure to the NGO's activities was highly heterogeneous, even within groups. The young men were exposed to different educational sessions, each lasting from 1 to 3 months. During this time, they also participated in other NGOs’ activities, such as rallies, theater presentations, and so on. We consider this an advantage rather than a disadvantage, as the heterogeneous sample allowed us to identify the similarities and differences between informants’ experiences.

In the FGDs, very few participants spontaneously identified themselves as homosexual or bisexual. The FGDs and the interviews lasted between 60 and 150 min and were conducted in Spanish. An audio device was used to record the data, which were subsequently transcribed verbatim.

For both the focus groups and the in-depth interviews, we used a semistructured discussion guide with open-ended questions to explore the participants’ perceptions of their training experience, masculinity, gender relations, and VAW (Table 1). The discussion guide was developed by the researchers as a part of a larger project exploring masculinities in Latin America and was adapted to the Nicaraguan context. To explore informants’ attitudes toward gender relations, we used hypothetical questions (56) such as: *Someone once told me that a good wife must obey her husband even if she doesn't agree with him. What do you think?* From this statement, we then asked participants to reflect on and discuss issues of gender and violence. These questions were modified from those used by the WHO multicountry study on women's health and domestic VAW in the section on attitudes toward gender roles (57).

**Table 1:** Semistructured interview guide

Topic	Questions
Experience as activist	1. Can you tell me why you became involved in this training program? What do you think of it?
Construction of masculinity	2. Can you tell me what gender is? And sexuality? Homosexuality? Violence against women?
	3. Do you consider yourself a man or a boy? In what way? What does it mean to be a man?
	4. How do you become a man? Are there any other ways?
	5. Are there any differences between you and young men who have not received this training? In what way? How do you feel about that?
	6. What makes you different from other young men?
Attitudes toward gender relations and violence against women	7. Someone once told me, ‘A good wife must obey her husband even if she doesn't agree with him’. What do you think about that?
	8. Somebody said to me, ‘If a husband mistreats his wife, people from outside the family have to intervene’. What do you think about that?
	9. Somebody said to me, ‘A woman has the right to choose her friends, even if her partner disagrees’. What do you think about that?
Vignettes	10. Johana is 20 years old. She has been Ruben's girlfriend for the past 3 years. Ruben has been acting very jealously lately, yelling and insulting her every time she says hello to a friend. Last time, he hit her when they got home.
	What do you think about that? Why?
	What would you do if you were Ruben's friend? Or if you were Johana's friend?
	What could she do in that situation?
	11. You go to a party with your best friend. Afterward, you see that your friend is talking to a girl who is very, very drunk. It looks like he wants her to go with him somewhere, but it seems that she doesn't want to go.
	What do you think about this situation? What would you do?
	What would you do if she was your friend?

In addition, to explore informants’ specific attitudes toward VAW, we used short oral vignettes (58) describing situations in which women were experiencing IPV and sexual abuse. For example, we used the following vignette to explore the participants’ attitudes toward IPV:

Johana is 20 years old. She has been Ruben's girlfriend for the last three years. Ruben has been acting very jealously lately, yelling and insulting her every time she says hello to a friend. The last time, he hit her when they got home. What do you think about this? Why?

Follow-up, probing, and interpretative questions were employed to further explore topics that emerged from the data (56). The emergent design of our study also allowed us to include new topics that arose from the previous discussions in the subsequent FGDs and interviews.

### Analysis

We analyzed the rich data gathered from the focus groups and interviews using the grounded theory method of constant comparison (59). We used this method because we understand reality as socially constructed (60); thus, we aimed to build theory emerging from the data rather than from a preconceived hypothesis. The data collection and analysis were conducted in parallel, so-called abduction. The analytical process started with a sentence-by-sentence open coding that helped us to identify concepts and actions that described the informant's experience (61). The open coding was conducted using OpenCode 3.4, a free software program developed by Umeå University (62). We then performed selective coding. This means that open codes that had something in common were grouped together, forming categories, thus representing a higher level of abstraction. During this process, a core category emerged, and its properties and dimensions were identified. Finally, we created a model grounded in the data that represented how the categories related to each other, a step called theoretical coding (63).

### Ethical considerations

The research protocol was approved by the Faculty of Medicine Ethical Committee of the National Nicaraguan Autonomous University, León. Participation in the research project was voluntary. We obtained oral informed consent before starting to collect the data and before recording the FGDs and interviews. All participants were informed about the objectives of the study and that they could withdraw from participation at anytime.

## Results

In the following section, we present an overview of the main findings. We then describe the core category in depth, the subcategories/ideal types and their properties and dimensions, and the factors shaping the core category. Constant comparison of the data allowed us to identify a core category representing the process of change in gender relations and masculinity among organized young Nicaraguan men and the individual and external factors facilitating or constraining this process (Fig. 1). This core category, labeled ‘Expanding your mind’, contains four subcategories representing the changing masculinities that emerged through the process (The apprentice, The responsible/respectful man, The proactive peer educator, and ‘The feminist man’). Following Max Weber, the subcategories are constructed as ideal types (64), which aim to capture the principal characteristics of the identified emergent masculinities. They should be understood as joint abstractions of the ideas and attitudes presented by the young men, and do not represent any particular man, rather summarize notions from several men in the different FGDs and interviews. The model also conceptualizes how the subcategories relate to each other.

**Fig. 1:** ‘Expanding your mind’: the process of constructing masculinities in young Nicaraguan men participating in training programs and the individual and social factors facilitating or constraining this process.

The grounded theory analysis allowed us to identify seven properties of the process with their respective dimensions. These properties are the degrees of awareness, respectfulness, and responsibility, and the development of a critical stance, political stance, thoughtful actions, and agency. Table 2 shows the properties listed according to the subcategory/ideal type where they were first identified. However, it is important to highlight that properties and dimensions cut across more than one subcategory. This is clearly evident in those subcategories emerging at the end of the process of change.

**Table 2:** Properties and dimensions of the core category ‘Expanding your mind’^a^

Subcategories/ideal types	Properties	Dimensions
The apprentice	Awareness	unawareness–consciousness
The responsible/respectful man	Respectfulness	Intolerant–tolerant
	Responsibility	Irresponsible–trustworthy
	Thoughtful actions	Impulsive–reflective
The proactive peer educator	Agency	Passive–active
‘The feminist man’	Critical stance	Incipient–developed
	Political stance	Conformist–activism

a Properties and dimensions are listed according to the subcategory where they were first identified. However, it is important to highlight that some properties and dimensions cut across more than one subcategory.

### ‘Expanding your mind’

#### The apprentice: increasing individual awareness

This subcategory arose from the young men's perceptions of their positions in their initial engagement with groups offering training in sexual and reproductive health and/or gender. During this period, awareness emerged as an important property of the process. Its dimension ranged from being unaware to becoming more conscious about reproductive health risks and gender inequalities. Awareness was described as ‘opening your eyes’. This process implied not only an increase in knowledge but also an increased awareness on the part of the young man of the consequences of his own actions, an expanded assessment of the risks of being sexually active (such as unintended pregnancies and sexually transmitted diseases), and a growing consciousness of the negative consequences of machismo and VAW for men and women.

The data analysis revealed that this subcategory was defined by two concomitant actions: learning and reflection. For The apprentice, learning meant that his continued exposure to the training allowed him to gradually acquire scientific knowledge on several issues previously considered taboo, such as sexuality, methods of contraception, sexually transmitted diseases, and HIV. Depending on the content of the training program, he might also begin to be exposed to concepts such as gender equity and/or VAW prevention. Exposure to this new information and the debates triggered by it in his interactions with other young men in the group seemed to facilitate a cognitive change, enabling The apprentice to begin to reflect upon his own experiences, behavior, and attitudes toward others, as well as the social context in which he interacted.

This training has helped to open my eyes to many things: to HIV, to what it can do to you, how you can get it … I've also learned about gender equity, about sexuality, how to take care of your sexuality … So it has helped to open my eyes. (FGD 6)

Nevertheless, for The apprentice, the process of learning and reflecting on his life experiences was perceived as difficult: an internal struggle to confront internalized machismo and homophobia. Challenging the burden of traditional gender relations seemed initially more complex than the lack of reproductive health knowledge. Gender equality was perceived as difficult to grasp because it represented a contrary set of values to those the young man had learned from his environment.

Getting to grips with gender equality was a little difficult for me … for me it was difficult to realize that men in our society are machistas and then … that men and women have the same rights and the same opportunities … it was very difficult because I had learnt something different at home … that women made the food and the men sat around … it was like … in my house they say one thing and here something else. (FGD 5)

VAW was often normalized. Blaming women for the violence they experienced from their partners was common and often understood as they ‘consented’ to it. The apprentice often felt that ending IPV was mainly the woman's responsibility.

#### The responsible/respectful man: thinking before acting

The responsible/respectful man emerged as a consequence of the reflecting process initiated by The apprentice. Young men's perceptions of their own cognitive and behavioral transformation represented the first challenge to machismo and constitute the foundations for this subcategory. The properties identified in this subcategory (responsibility, thoughtful action, and respectfulness) represent the identified behavioral and cognitive transformations. The dimensions outline how The responsible/respectful man perceived himself as following a journey from irresponsibility to greater trustworthiness, from impulsiveness to thoughtfulness about his own behavior, and from intolerance to greater acceptance of others’ ideas and behavior. These properties might represent the informants’ first steps in shifting gender relations toward a more empathic and considerate stance regarding the needs and rights of others around them.

The responsible/respectful man perceived this period as a time of transformation, with the concept of growing into manhood strongly emerging from the data. This growing process was strongly associated with ‘walking the talk’, meaning putting into practice the knowledge the participant had previously acquired. A key cognitive change was that the participant began to assume responsibility for his own actions, especially regarding his sexuality – for example, a more responsible sexual behavior that challenged indiscriminate sexual contacts as a sign of manhood and that included pregnancy prevention and condom use during sexual intercourse.

To me, being a man means being faithful to your wife. I don't believe that having a lot of women means you are a man, I think it means you're a machista. To me, being a man means having only one woman, supporting your kids, giving them love and support, helping them anyway you can. (FGD 2)

In addition, the concept of thoughtful action emerged as a significant finding from the data. The responsible/respectful man recognized that he was dissimilar to his peers, i.e. that he behaved differently from ‘regular guys’. He also clearly recognized how his decision-making pathway was different from other young men not engaged in gender or reproductive health training – because he did not act on impulse. Thoughtful action for him meant ‘thinking before acting’, taking into account the consequences of his acts before choosing how to behave.

I think that what makes us different to other men is that they haven't had the information we have … we are conscious of what we're doing. We are conscious of the harm we can inflict; basically you have to be aware of your own actions and give your best to all humanity, because we are human, too, and you can hurt yourself as well … You have to think twice before doing something. (FGD 1)

The constant comparison of the data enabled us to identify other significant cognitive changes in this phase. Growing into a man meant ‘opening your mind’, a process of becoming more respectful and tolerant of other people's ideas, of women, and of sexual options other than heterosexuality. However, this transition did not seem to be complete. Although The responsible/respectful man claimed to be more open, homophobia, machismo, and heteronormativity persisted to some degree. In addition, The responsible/respectful man often expressed an ambiguous stance regarding traditional gender relations and VAW. Even though he expressed attitudes rejecting IPV and sexual abuse, partner abuse was still perceived as a private issue, and men's sexual harassment of women was somehow tolerated if it was unclear whether the situation implied the woman's consent or not. The responsible/respectful man was in two minds about how to react to IPV and sexual abuse: he mostly felt that his involvement would serve no purpose and not end the abuse and thus felt uncomfortable getting involved.

#### The proactive peer educator: empowering others

This subcategory/ideal type represents the transition from an individual, focused on his own cognitive and behavioral change, to a young man more focused on empowering others by promoting cognitive and behavioral change in his peers. The proactive peer educator's focus on action allows us to identify agency as an emergent property of this subcategory, with its dimension moving from passivity to action.

The proactive peer educator represents a young man who influences others by sharing his knowledge on reproductive/sexual health and gender. He aims to help other young people avoid the negative consequences of unsafe sexual behavior, such as unintended pregnancies, sexually transmitted disease, and HIV. However, sharing knowledge was not the only action defining The proactive peer educator. Passing on new values to his peers was also mentioned as an important part of the process of helping others to reflect on their own behavior and on how that behavior had been influenced by machismo. Key values that The proactive peer educator strived to promote were respect for other people's views, respect for women, and gender equity. The process of empowering others seemed also to boost The proactive peer educator's self-esteem, generating feelings of pride:

This training has helped me to learn many things about sexuality, about HIV, about gender equity, the meaning of sexuality … This experience has helped me remove the blinkers we were wearing, and it has helped us to remove other people's blinkers … Because the objective is not to keep the things you've learned to yourself but to disseminate the information to others. (FGD 4)

In addition, The proactive peer educator expressed a more positive attitude toward getting involved in the process of ending IPV and sexual abuse. However, ambivalence toward intervention persisted. The concept of conditional intervention emerged, influencing his actions to end abuse. His choices regarding actions to end violence appeared to depend on the severity of the abuse, the closeness to the victim, and concerns about his own safety. Providing emotional support, informing women about their rights, advising them to seek help, and promoting attitudinal change in the abusive man were the most common types of interventions described. In cases where the relationship with the victim was closer (e.g. friends and family members), The proactive peer educator seemed to be more willing to advise the woman to go to the police, call the police himself, or even to intervene physically to halt the violence.

Well, if she (a victim of IPV) was my friend, I'd advise her to leave, but if she was my sister … boy, if a guy hit my sister he'd be in serious trouble, because I'd hit him back … that's for sure! (Interview N.1)

#### ‘The feminist man’: advocating social change

The prominent feature of this subcategory is that ‘The feminist man’ strives to promote change not only at the individual level but also at societal level. This meant developing an analytical and critical stance to his social context, a key property of this phase, whose dimension signified a journey from an incipient to a developed critique of the gender order in Nicaragua. In addition, it implied assuming a political stance, another property, whose dimension represented a shifting from conformist to vigorously opposing the gender order. This subcategory was named ‘The feminist man’ because some young men spontaneously identified themselves as such.

Critical analysis of the social context allowed ‘The feminist man’ to begin to identify the inequities of gender relations (e.g. women's economic dependency) and to become more empathic toward women and lesbians, gays, bisexuals, and transgenders (LGTB). These reflections on the gender order influenced a cognitive change toward challenging and rejecting heteronormativity, traditional gender norms on homosexuality, and traditional gender relations:

Being *machista* is not cool. We are feminists because it is the opposite of being *machistas* … We recognize that men and women must have equal status in society, and that neither gender should be superior to the other … We believe that both sexes are equal and that machismo is a conduct learned in this patriarchal society. (FGD 3)

In addition, ‘The feminist man’ strongly rejected men's controlling behavior, unsafe sexual behavior, and hypersexuality as signs of masculinity. This meant that he started to experience a change in his own gender relations, performing actions traditionally deemed feminine, such as cooking and laundry. ‘The feminist man’ also experienced an important emotional turning point: he felt comfortable with his own sexuality and comfortable showing emotions and affection to others.

Declaring a political stance against traditional gender relations and VAW emerges as another key characteristic of ‘The feminist man.’ This young man perceives himself as different because he recognizes women's, gay, lesbian, and transsexual rights and autonomy. Thus, he assumes a political stance toward promoting and defending those values and rights. Regarding VAW, he felt frustrated about what he perceived as some women's submissive behavior to men and their silence and passivity in the face of IPV. These perceived attitudes and behaviors were among the most contested and criticized gender relations between men and women.

A key difference between The proactive peer educator and ‘The feminist man’ was that the latter rejected VAW in all circumstances and was more likely to get involved in actions to prevent or end the abuse regardless of whether he was acquainted with the victim or not. He clearly recognized VAW as a public rather than a private issue. This does not mean that The Proactive peer educator and ‘Feminist man’ subcategories/ideal types are opposites: they are defined not only by the focus of their actions but also by the way they think about gender relations and their level of political involvement in promoting more gender-equitable rights. The proactive peer educator might become a ‘Feminist man’ if critical thinking and reflection about the societal gender order are an important part of his process of change. Conversely, ‘The feminist man’ can also conduct peer education in sexual and reproductive health if required. The arrows in the model show the variability in subjects’ positions.

### Factors facilitating or constraining the process

The rich data show that external and individual factors interacted with the core category, ‘Expanding your mind,’ influencing its course. Peer and family pressure to conform to traditional norms and values emerged from the data as strong external factors hindering the process. Fear of rejection and discrimination, generated by the strongly critical environment the young man faced, hindered his cognitive and behavioral transition. However, as he moved along the process, he began to develop coping skills to deal with the stigma and discrimination that was generated by confronting his peers and his community with his new beliefs and behaviors.

I think I'm different to my friends … They say I act too defensively when they talk about women. When I hear them talking about mistreating women, or when they make nasty sexual comments about their girlfriends, I usually tell them … Hey, don't talk like that; remember that a woman gave birth to you and now you are insulting them (women) … sometimes they get mad and call me a faggot, gay, homosexual … And I tell them, why am I a faggot? Just because I defend women, just because I am conscious that you don't have to treat women like that? (FGD 4)

We identified that belonging to a group that shared the same gender-equitable values and behaviors was an important external factor facilitating the transition. This allowed the participant to receive group support and reinforcement of his behavioral and cognitive change, which proved to be a significant and necessary factor to sustain his new stance toward gender relations.

When I joined the group, people had started to say that I was gay just because I had started to think differently … In the group we always talk about how to overcome those criticisms. (FGD 2)

## Discussion

The main findings of this study show that our informants experienced a process of change labeled ‘Expanding your mind.’ This represents a gradual transition from values and behaviors rooted in traditional gender norms and masculinity to forms of masculinities that challenge norms that justify VAW and that promote healthy sexual and reproductive behaviors and gender-equitable attitudes. The process also shows how an increased awareness of gender inequities facilitated the emergence of values (respect and responsibility) and behavior (thoughtful action) that contributed to increase the informant's critical thinking and agency at an individual, social, and political level. The flow and direction of the process was influenced by individual and external factors facilitating or constraining it.

### ‘Expanding your mind’: the process of change

To our informants, going through the process implied a series of personal transformations. However, this process should not be understood as linear: the different subcategories/ideal types are not static and the young men moved between them as they journeyed through their process of change. Many researchers, around the world (2, 4, 8, 10, 13) and in Latin America (17–19, 21, 22), have pointed out the many types of masculinity and its relational characteristic in the general population. Connell has stated that gender relations are dynamic entities that can evolve by the pressure of men and women challenging them (4). Our results show that this transition is possible and that multiple progressive intertwined masculinities can emerge from the process of change in young men engaged in gender and reproductive health programs.

The process that emerged from the data showed a positive transformation regarding norms and values relating to gender relations. This transition has been identified by Connell as an important sign of changing masculinities (65). In addition, we concur with Howson that protest femininities are important factors shaping masculinities around the world (7). The process of change among the participants was clearly promoted and facilitated by the rising protest femininity movements in Nicaragua (44) related to the growing feminist agenda in Latin America (21).

In the last 20 years, with the aim to challenge traditional patriarchal norms, several NGOs around the world have conducted activities with adult and young men (23, 24, 26, 46, 55, 66). Many of these NGOs have documented changes in norms, attitudes, and behaviors in their target populations (26, 46, 55, 66). However, the majority of these reports did not examine the process of change their target populations experienced in-depth. We were able to identify only one published study describing the pathways that men followed in their involvement in activities against VAW, and this was from the United States (67). To the best of our knowledge, ours is one of the first qualitative studies to explore the process of change in young men participating in activities aimed at challenging patriarchy in Latin America.

The properties and dimensions of the core category process cut across the ideal types and represent the behavioral and cognitive changes identified. Thus, in the following discussion, rather than discussing the ideal types we address the process of change by discussing the properties and dimensions that emerged from the grounded theory analysis.

### Awareness, responsibility, and thoughtful action

Hegemonic masculinities often affect young men's health by encouraging risk-taking and unsafe sexual behavior (32, 33), and studies confirm that Nicaraguan young men are not an exception (50–52). Our results show that young men participating in reproductive health and/or gender equality programs experienced an increased awareness of how machismo(s) shaped their gender relations and how these unequal gender relations affected both women's and their own health. This was more evident during the initial part of the change process, where participants developed emergent masculinities that started to exhibit positive attitudes and behaviors toward a more responsible sexual and reproductive conduct. These positive changes mirror the findings of studies conducted in other low-income countries (46, 66, 68) as well as in Nicaragua (44), assessing the effectiveness of gender and reproductive health programs in changing young men's attitudes and behaviors toward sexuality and reproduction.

Being a responsible man has been part of the different expressions of hegemonic masculinities in Latin America. It has mainly been associated with being the breadwinner for one's family and policing family members’ behavior (17, 18, 22, 45, 49). We argue that the concept of responsibility that emerges from the process that our informants experienced is different from the dominative principle expressed by the hegemonic patterns of masculinities (7). The concept expressed by our informants focuses more on increasing their accountability for their own behaviors and less on becoming their family's breadwinner. Increasing young men's accountability for their own behaviors has been identified as a key attitudinal change in the process of achieving more gender-equitable and non-violent masculinities (29). Developing new values such as responsibility facilitated the emergence of the property of thoughtful action, constructed in opposition to impulsivity, a behavior that our participants strongly associated with traditional machismo(s). This property is very similar to what Weber, cited in Hughes et al., defines as ‘value rational action’ (69), in which people's actions are influenced by their own values rather than the benefits they can obtain from them.

### Respect and ambiguity

As the young men moved along the process, they seem to have modified their gender relations, expressing more gender-equitable attitudes and behaviors that challenged traditional power relations between men and women, homophobia, and VAW. Respect emerged as a key property behind these changes, emphasizing tolerance toward other people's views and sexual practices. This also implied that participants gradually developed a more empathic stance toward the needs and rights of women and subordinated masculinities (1, 2). The concept of respect emphasized by our informants is quite different from the meaning constructed by hegemonic masculinities and traditional machismo(s) in Latin America, which associate respect with authoritarianism and obedience to the male head of the household (7, 17, 18).

Although respect is a key property of the process of change, it is important to emphasize that this attitudinal and cognitive transition developed gradually, and where some forms of emergent masculinities – The apprentice, The responsible/respectful man, and The proactive peer educator – showed an ambiguous stance toward subordinated masculinities and VAW. This is in line with the findings of Pulerwitz et al. (26), showing that inequitable and equitable attitudes can coexist in young men receiving gender training and that, as illustrated in one Mexican study, some patriarchal attitudes are more easily changed than others (22).

This ambiguity can clearly be recognized in the participants’ perceptions of their own involvement in actions aimed at preventing or ending VAW. Latané and Darley (70) and, more recently, Casey and Ohler (71), using a bystander model, identified that intervening to prevent or stop VAW is a complex process that goes from identifying the violence to assuming personal responsibility to implementing the selected actions. In addition, they identified that men's choices of strategy to end or prevent the VAW are influenced by contextual and individual factors. Bystander model by Casey and Ohler (71) clearly fits our informants’ experiences. Their disapproval of VAW and willingness to prevent or deter it increased as they moved along the process of change. However, their choice of action or inaction was conditioned by their perception of the severity of the abuse, concerns for their own safety, and their closeness to the victim, which is in line with the factors reported by Casey and Ohler (71).

Our informants’ reactions to VAW can also be explained within the manhood acts theoretical framework, which focuses on the actions that men do to differentiate themselves from women (12). Schrock and Schwalbe suggest that manhood acts can vary according to situation. One such situation is when men refuse to act as the hegemonic masculinity ideal dictates (12), and this seems to be the case depicted in our informants’ process of change. It is important to stress that even if the informant's choice of strategies to end or prevent VAW was mainly based on non-confrontation, the masculinities that emerged at the end of the process (The proactive peer educator and ‘The feminist man’) were more inclined to action rather than passively standing by.

### Agency, empowerment, critical thinking, and political stance

Involving men in actions aiming to improve gender equality in health has been described as a key strategy to achieve successful results (28, 46). Broido has proposed that men, as members of the dominant social group, can become important allies in the process of ending the unequal gender order (72). Our results suggest that the progressive masculinities identified at the end of our informants’ process of change, The proactive peer educator and ‘The feminist man,’ are crucial allies in the struggle to change the current gender order.

The proactive peer educator and ‘The feminist man’ represent empowered forms of masculinity in the sense that they increased their self-esteem, expanded their ability to take action, and developed agency to achieve their desired results (73, 74). In addition, both masculinities aimed to fight gender inequality by openly criticizing other young men's oppressive manhood acts (12). However, they also show important differences. The proactive peer educator masculinity does not seem to have the same level of critical stance toward the gender order and political commitment to challenge patriarchy as ‘The feminist man.’ Another key difference is that ‘The feminist man’ clearly opposes marianismo (16), the emphasized femininity (7) in Latin America that promotes a female ideal as docile and compliant to men as well as the privileges that the gender order bestows to complicit masculinities (2, 7). We found that our emergent concept, ‘The feminist man,’ is similar to what Pulerwitz and Barker (68) have defined as the ‘Gender equitable man’. This is a man who strives to have equitable gender relations based on respect, non-violence, and tolerance for sexual options other than heterosexuality.

A possible reason for the differences between The proactive peer educator and ‘The feminist man’ could be the diverse focus of the educational activities they have been exposed to. There might be significant variation in how gender and masculinities are approached within these activities (46, 75). In our study, the NGOs’ activities were highly heterogeneous; thus, it was not possible to clearly identify how gender issues were approached and how these influenced the identified masculinities. Further studies are needed to assess whether the focus of the various NGOs’ activities influenced the masculinities that emerged from the young men's processes of change.

### Individual and social factors facilitating or constraining the process of change

As reported by other researchers, social pressure to adhere to the traditional patriarchal norms and internalized machismo are the most important factors influencing young men's behaviors toward sexuality and gender (32–34) and constraining the process. However, belonging to a group that shared the same gender-equitable values was a key factor reinforcing our informants’ process of change. It provided them with a safe place to reflect upon gender and to build a network of peers who supported and encouraged their new subject position on traditional gender values and behaviors. This is in line with the findings of several researchers pointing to the key role of group membership in facilitating and maintaining individual cognitive and behavioral change (22, 25, 26, 28, 29, 67).

### Limitations and strengths

A qualitative study aims to explore and understand informants’ experiences in depth. According to Marshall (76), the optimum sample size for a qualitative study is ‘one that adequately answers the research question’. We found that the data collected from our 62 informants (divided into six focus groups and two in-depth interviews) provided sufficient information to answer our research question. This means that we collected data until no new information relating to our research question emerged, thus reaching saturation. Although our sample size might be small for a quantitative study, it is sufficient for a qualitative study in which our aim is to illuminate the transformation process experienced by the informants. With our results, our aim is for theoretical rather than statistical generalization (63, 77).

In grounded theory, models are constructed using an abductive method – that is, generating theory that is rooted in the data and then continuously testing that theory with new informants (61). By applying this method, we found that the informants recognized that they had experienced a process of change. One possible criticism of our model is that it appears to describe a linear process of change. To our informants, going through the process implied a series of personal transformations. We argue that this process is not the same for all young men; we cannot prove exactly where in the suggested process they were when each individual started. We conclude that they have a number of common starting points but can develop different progressive masculinities depending on the influence of individual and external factors.

A limitation of this study is that we cannot explore whether the cognitive and behavioral changes reported by our informants will persist once they have ceased their interaction with the NGOs. They might revert to their preintervention attitudes and behaviors (24, 29, 66). However, a number of authors have argued that young men who have been involved in activities where they were engaged in reflecting critically about the gender order and masculinity and those who have a political stance are more likely to maintain their cognitive and behavioral change (25, 29, 78).

Social desirability bias is a risk when conducting any kind of research, especially when trying to portray change. To reduce this bias, we used several strategies during data collection, for instance, vignettes to encourage discussion of sensitive issues. This allowed us to collect the group participants’ different attitudes toward VAW and gender topics. Further, we used follow-up and probing questions to gain a deeper understanding of the issues raised during the group discussions or interviews and to encourage discussion among the focus group participants. During the whole process, we acted in a non-judgmental manner, encouraging informants to speak freely; we also emphasized that there were no right or wrong statements.

We aimed to increase the credibility of our findings by constructing a team of researchers with different theoretical backgrounds – such as public health, medical sociology, and gender studies – to analyze the data (77). Information was gathered using different data collection methods, a technique known as triangulation. Further, we conducted a ‘peer-debriefing’ session, a technique that consists of discussing our findings with other colleagues not involved in the analysis of the data (77).

## Conclusions

Our results show that multiple progressive masculinities can emerge from activities challenging traditional masculinity in this Latin American setting. Developing gender-equitable attitudes and behaviors is experienced as a process involving not only acquisition of information but also developing respect for other people's views and sexual choices, assuming responsibility for one's own sexual behavior, and learning and applying new skills such as agency, critical thinking, and a political stance.

The emergent masculinities identified in this study show a range of attitudes and behaviors; however, all lean toward more equitable gender relations. It is important to highlight that changes in gender relations were identified not only between genders but also within a gender. The masculinities that developed at the end of the process showed greater tolerance and respect toward men who have sex with other men, also termed subordinated masculinities in the construction of hegemonic masculinities. In addition, the emerging masculinities identified aimed to build relationships of mentorship and leadership with other men rather than ones of subordination, complicity, or marginalization.

Clearly, the masculinities that emerged during the informants’ processes of change can be health promoting, facilitating the empowerment of other young men and women. However, the results suggest that learning about sexual and reproductive health does not directly imply developing more gender-equitable attitudes and behaviors or a greater willingness to prevent VAW. It is paramount that institutions working with young men consider their strategic as well as their practical interest during the planning stage of activities targeting this population. Activities that aim to challenge machismo in this setting must continue and be expanded to reach more young men. Including discussion of gender equity, masculinity, and VAW in the curricula of the public education system might be an important way to reach more young men. As stated above, further studies are needed to deepen our knowledge of how the masculinities that emerged from the informants’ processes of change are influenced by the focus of the various NGOs’ activities.

## References

[1] Connell RW (2009). Gender.

[2] Conell RW (2005). Masculinities.

[3] Walby S (1989). Theorising patriarchy. Sociology.

[4] Connell RW, Messerschmidt JW (2005). Hegemonic masculinity: rethinking the concept. Gend Soc.

[5] Demetriou D (2001). Connell's concept of hegemonic masculinity: a critique. Theory Soc.

[6] Hirose A, Kei-ho Pih K (2010). Men who strike and men who submit: hegemonic and marginalized masculinities in mixed martial arts. Men Masc.

[7] Howson R (2006). Challenging hegemonic masculinity.

[8] Kimmel M, Pease B, Pringle K (2001). Global masculinities: restoration and resistance. A man's world? Changing men's practices in a globalized world.

[9] Dernés S (2002). Globalization and the reconstitution of local gender arrangements. Men Masc.

[10] Beasley C (2008). Rethinking hegemonic masculinity in a globalizing world. Men Masc.

[11] Pringle K, Hearn J, Pease B, Ruspini E, Ruspini E, Hearn J, Pease B, Pringle K (2011). Introduction. Men and masculinities around the world: transforming men's practices.

[12] Schrock D, Schwalbe M (2009). Men, masculinity, and manhood acts. Annu Rev Sociol.

[13] Whitehead SM (2002). Men and masculinities. Key themes and new directions.

[14] Hearn J (2004). From hegemonic masculinity to the hegemony of men. Feminist Theory.

[15] Martin PY (1998). Review: why can't a man be more like a woman? Reflections on Connell's masculinities. Gend Soc.

[16] Stevens EP (1973). Machismo and marianismo. Society.

[17] Stobbe L (2005). Doing machismo: legitimating speech acts as a selection discourse. Gend Work Organ.

[18] Torres JB, Solberg VSH, Carlstrom AH (2002). The myth of sameness among Latino men and their machismo. Am J Orthopsychiatry.

[19] Mirandé A (1997). Hombres y machos: masculinity and Latino culture.

[20] Arciniega GM, Anderson TC, Tovar-Blank ZG, Tracey TJG (2008). Toward a fuller conception of machismo: development of a traditional Machismo and Caballerismo Scale. J Couns Psychol.

[21] Gutmann M (2003). Changing men and masculinities in Latin America.

[22] Ramirez Rodriguez JC, Ruspini E, Hearn J, Pease B, Pringle K (2011). Young Mexican men divided: a possibility for transforming masculinity. Men and masculinities around the world: transforming men's practices.

[23] Viveros Goya M, Ruspini E, Hearn J, Pease B, Pringle K (2011). Masculinities and social intervention in Colombia. Men and masculinities around the world: transforming men's practices.

[24] White V, Greene M, Murphy E (2003). The Synergy Project. Men and reproductive health programs: influencing gender norms. http://www.usaid.gov/our_work/global_health/pop/.../gendernorms.doc.

[25] Barker G, Nascimento M, Segundo M, Pulerwitz J (2004). How do we know if men have changed? Promoting and measuring attitude change with young men. Lessons from Program H in Latin America. http://www.un.org/womenwatch/daw/egm/men-boys2003/OP2-Barker.pdf2003.

[26] Pulerwitz J, Michaelis A, Verma R, Weiss E (2010). Addressing gender dynamics and engaging men in HIV programs: lessons learned from horizons research. Public Health Rep.

[27] World Health Organization (2011). WHO gender mainstreaming strategy. Integrating gender analysis and actions into the work of WHO. http://www.who.int/gender/mainstreaming/strategy/en/.

[28] Women's Commission for Refugee Women and Children (2005). Masculinities: male roles and male involvement in the promotion of gender equality. A resource packet. http://www.unicef.org/emerg/files/male_roles.pdf2005.

[29] Flood M (2005). Harmful traditional and cultural practices related to violence against women and successful strategies to eliminate such practices – working with men. United Nations Economic and Social Commission for Asia and the Pacific (UNESCAP) Expert Group Meeting – strategies for implementing the recommendations from the Secretary-General's Study on Violence against Women with Particular Emphasis on the Role of National Machineries.

[30] Evans J, Frank B, Oliffe JL, Gregory D (2011). Health, illness, men and masculinities (HIMM): a theoretical framework for understanding men and their health. J Mens health.

[31] Courtenay W (2000). Behavioral factors associated with disease, injury, and death among men: evidence and implications for prevention. J Mens Stud.

[32] Marston C, King E (2006). Factors that shape young people's sexual behaviour: a systematic review. Lancet.

[33] Kostrzewa K (2008). The sexual and reproductive health of young people in Latin America: evidence from WHO case studies. Salud Publica Mex.

[34] World Health Organization (2000). What about the boys? A literature review on the health and development of adolescent boys. http://whqlibdoc.who.int/hq/2000/WHO_FCH_CAH_00.7.pdf.

[35] Courtenay WH (2000). Constructions of masculinity and their influence on men's well-being: a theory of gender and health. Soc Sci Med.

[36] Courtenay WH (2000). Engendering health: a social constructionist examination of men's health beliefs and behaviors. Psychol Men Masc.

[37] Jewkes R (2002). Intimate partner violence: causes and prevention. Lancet.

[38] Watts C, Zimmerman C (2002). Violence against women: global scope and magnitude. Lancet.

[39] Garcia-Moreno C, Jansen HA, Ellsberg M, Heise L, Watts CH (2006). Prevalence of intimate partner violence: findings from the WHO multi-country study on women's health and domestic violence. Lancet.

[40] Campbell JC (2002). Health consequences of intimate partner violence. Lancet.

[41] Ellsberg M, Jansen HAFM, Heise L, Watts CH, Garcia-Moreno C (2008). Intimate partner violence and women's physical and mental health in the WHO multi-country study on women's health and domestic violence: an observational study. Lancet.

[42] Salazar M, Valladares E, Ohman A, Hogberg U (2009). Ending intimate partner violence after pregnancy: findings from a community-based longitudinal study in Nicaragua. BMC Public Health.

[43] Sternberg P, White A, Hubley JH (2008). Damned if they do, damned if they don't: tensions in Nicaraguan masculinities as barriers to sexual and reproductive health promotion. Men Masc.

[44] Welsh P (2010). Community development: a gendered activism? The masculinities question. Community Dev J.

[45] Sternberg P (2000). Challenging machismo: promoting sexual and reproductive health with Nicaraguan men. Gend Dev.

[46] Barker G, Ricardo C, Nascimento M (2007). Engaging men and boys in changing gender-based inequity in health: evidence from programme interventions. http://www.who.int/gender/documents/Engaging_men_boys.pdf2007.

[47] INIDE (2006). VIII population survey and IV household survey. Educational characteristics of the population. Managua, Nicaragua. http://www.inide.gob.ni/censos2005/VolPoblacion/Volumen%20Poblacion%201-4/Vol.II%20Poblacion-Caracteristicas%20Educ.pdf.

[48] Welsh P, Stankovitch M, Bradbury A (2001). Men aren't from Mars. Unlearning machismo in Nicaragua.

[49] Lancaster R (1992). Life is hard. Machismo, danger and the intimacy of power in Nicaragua.

[50] Berglund S (2008). Competing everyday discourses: the construction of heterosexual risk-taking behaviour among adolescents in Nicaragua: towards a strategy for sexual and reproductive health empowerment.

[51] Kalk A, Kroeger A, Meyer R, Cuan M, Dickson R (2001). Influences on condom use among young men in Managua, Nicaragua. Cult Health Sex.

[52] Manji A, Peña R, Dubrow R (2007). Sex, condoms, gender roles, and HIV transmission knowledge among adolescents in León, Nicaragua: implications for HIV prevention. AIDS Care.

[53] Claeys P, Gonzalez C, Gonzalez M, Van Renterghem L, Temmerman M (2002). Prevalence and risk factors of sexually transmitted infections and cervical neoplasia in women's health clinics in Nicaragua. Sex Transm Infect.

[54] INIDE (2008). Nicaraguan Demographic and Health Survey 2006/2007. Final Report Managua.

[55] Solórzano I, Bank A, Peña R, Espinoza H, Ellsberg M, Pulerwitz J (2008). Catalyzing individual and social change around gender, sexuality, and HIV: impact evaluation of Puntos de Encuentro's communication strategy in Nicaragua. Horizons Final Report. http://www.popcouncil.org/pdfs/horizons/Nicaragua_StigmaReduction.pdf.

[56] Kvale S (1996). Interviews: an introduction to qualitative research interviewing.

[57] García-Moreno C, Jansen HA, Ellsberg M, Heise L, Watts CH (2005). WHO multi-country study on women's health and domestic violence against women: initial results on prevalence, health outcomes and women's responses. http://www.who.int/gender/violence/who_multicountry_study/en/.

[58] Soydan H, Hantrais L, Mangen S (1996). Using the vignette method in cross-cultural comparisons. Cross-national research methods in the social sciences.

[59] Glaser B, Strauss A (1967). The discovery of grounded theory.

[60] Berger PL, Luckmann T (1967). The social construction of reality: a treatise in the sociology of knowledge.

[61] Glaser B (1978). Theoretical sensitivity.

[62] UMDAC (2001). Epidemiology Department of Public Health and Clinical Medicine Umeå University. OpenCode 3.4.

[63] Dahlgren L, Emmelin M, Winkvist A (2004). Qualitative methodology for international public health.

[64] Weber M, Sieback P (1972). Wirtschaft und Gesellschaft. Die Wirtschaft und die gesellschaftlichen Ordnungen und Mächte.

[65] Conell RW (2000). The men and the boys.

[66] Verma RK, Pulerwitz J, Mahendra V, Khandekar S, Barker G, Fulpagare P (2006). Challenging and changing gender attitudes among young men in Mumbai, India. Reprod Health Matters.

[67] Casey E, Smith T (2010). ‘How Can I Not?’: men's pathways to involvement in anti-violence against women work. Violence Against Women.

[68] Pulerwitz J, Barker G (2008). Measuring attitudes toward gender norms among young men in Brazil. Men Masc.

[69] Hughes JA, Martin PJ, Sharrock WW (1995). Understanding classical sociology. Marx, Weber, Durkheim.

[70] Latané B, Darley J (1969). Bystander ‘apathy’. Am Sci.

[71] Casey EA, Ohler K (2011). Being a positive bystander: male antiviolence allies’ experiences of ‘stepping up. J Interpers Violence.

[72] Broido EM (2000). The development of social justice allies during college: a phenomenological investigation. J Coll Stud Dev.

[73] Alsop R, Heinsohn N (2005). Measuring empowerment in practice: structuring analysis and framing indicators. http://siteresources.worldbank.org/INTEMPOWERMENT/Resources/41307_wps3510.pdf.

[74] Mosedale S (2005). Assessing women's empowerment: towards a conceptual framework. J Int Dev.

[75] Gupta G, Whelan D, Allendor A (2003). Integrating gender into HIV/AIDS programmes. A review paper. http://www.who.int/gender/hiv_aids/en/Integrating.pdf.

[76] Marshall MN (1996). Sampling for qualitative research. Fam Pract.

[77] Lincoln Y, Guba E (1985). Naturalistic inquiry.

[78] McAdam D (1989). The biographical consequences of activism. Am Sociol Rev.

# Sub article: Condemning violence without rejecting sexism? Exploring how young men understand intimate partner violence in Ecuador

**DOI:** 10.3402/gha.v5i0.18049

**Published:** 2012-06-19

**Authors:** Isabel Goicolea, Ann Öhman, Mariano Salazar Torres, Ione Morrás, Kerstin Edin

**Affiliations:** 1 Umeå Center for Global Health Research, Umeå University, Umeå, Sweden; 2 Umeå Center for Gender Studies, Umeå University, Umeå, Sweden; 3 Center for Demography and Health Research, Nicaraguan National Autonomous University, León, Nicaragua; 4 Medicus Mundi Gipuzkoa, San Sebastian, Spain; 5 Red de Prevención y Atención de la Violencia de Francisco de Orellana, Coca, Ecuador

**Keywords:** intimate partner violence, qualitative content analysis, gender, masculinities, gender equality, machismo, Ecuador

## Abstract

**Background:**

This study aims to explore young men’s understanding of intimate partner violence (IPV) in Ecuador, examining similarities and differences between how ordinary and activist young men conceptualize IPV against women.

**Methods:**

We conducted individual interviews and focus group discussions (FGDs) with 35 young men – five FGDs and five interviews with ordinary young men, and 11 interviews with activists – and analysed the data generated using qualitative content analysis.

**Results:**

Among the ordinary young men the theme ‘too much gender equality leads to IPV’ emerged, while among the activists the theme ‘gender inequality is the root of IPV’. Although both groups in our study rejected IPV, their positions differed, and we claim that this is relevant. While activists considered IPV as rooted in gender inequality, ordinary young men understood it as a response to the conflicts generated by increasing gender equality and women’s attempts to gain autonomy.

Intimate partner violence (IPV), defined by the World Health Organization as ‘behavior within an intimate relationship that causes physical, sexual or psychological harm, including acts of physical aggression, sexual coercion, psychological abuse and controlling behavior’ (1), is the most prevalent form of violence against women, with devastating effects on their health and well-being (1–3). Although we acknowledge that IPV can occur against men and within same-sex intimate relationships, here we focus on men’s intimate partner violence against women, and the acronym used – IPV – refers to that. Despite the fact that IPV remains commonplace, progress towards its eradication has been notoriously difficult; however, IPV is today less accepted and has become the subject of public policies, laws and interventions in many countries (1, 4).

From a feminist perspective, IPV is one extreme consequence of gender-power structures that force women into a subordinate position (5). Consequently, dismantling sexism is the key to eradicating IPV. However, the connections between sexism and patriarchal dominance and intimate partner violence are not as straightforward as it might appear to be. On the one hand research shows that men who commit IPV have more sexist attitudes, and IPV is more prevalent in settings with higher gender inequality (6, 7). On the other hand, progressive changes towards gender equality may also trigger IPV as a reaction against increased women’s autonomy (7–9). Ambivalent sexism theory states that the pervasiveness of sexism is based on its ambivalent construction. According to this theory, sexism has two faces: hostile sexism, constituted by all the extreme features that may arise, less acceptance and sanction; and benevolent sexism, constituted by features that sustain gender inequality and women subordination but that are categorized as prosocial and may even be honored (9–12). In the Latin American context Torres has applied this theory to explore the complexities of machismo, and other authors have also discussed its ambivalent features (13, 14). Machismo can be considered a regional form of sexism that is hegemonic in Latin America – and elsewhere (15–19). Marianismo, constructed as machismo’s complement, stresses the construction of women as submissive, chaste and self-sacrificing – a concept similar to Connell’s emphasized femininity (20–22). Similar to what ambivalent sexism theory states, several Latin American authors (13, 14, 16) have proposed that machismo can be considered as including both positive and negative characteristics. Torres, for example, identifies five different types of machismo, which differed in issues such as authoritarianism, control over women and openness to change (13).

While for many years the fight against IPV focused on women’s empowerment and targeting men only as potential perpetrators (5, 23, 24), more recently the need to involve men has been highlighted (25, 26). Evidence shows that young men are the most likely to engage in IPV (26), that men’s attitudes to and perceptions of gender relations and IPV are strongly linked with exercising violence against their partners (27), and that programmes that promote gender equality among young men show promising results (6, 27–31). Despite such evidence, research on IPV has paid only limited attention to exploring how young men understand IPV – especially in low-income settings (26, 27) – and even less to whether these understandings may change among young men who participate in programmes promoting gender equality.

In Ecuador, where this study was conducted, IPV remains prevalent and severe: the last DHS (Demographic Health Survey) conducted in 2004 showed that 46% of Ecuadorian women had experienced violence during their lifetime, with 95% of cases occurring at home (32). This is despite that the country is making notable progress putting IPV on the public agenda (33). The Law Against Violence Against Women and the Family (Law 103) was passed in 1995, one year after the first Women’s Police Stations were set up. Law 103, together with initiatives in the health and education sectors, represented a considerable advance, not least because it contributed to visibilizing an issue that was previously portrayed as private (34, 35). Twelve years later, in 2007, a presidential decree was passed declaring the ‘eradication of IPV as state policy’. A commission was also established at the highest level, launching the ‘National Plan for the Eradication of Gender-Based Violence’ (36). Unlike Law 103, the decree and plan explicitly state that IPV is rooted in gender inequality and machismo. The decree and plan also address the state’s responsibility to support shelters and centres to provide adequate care for victims, and include amongst the five main strategies one aimed at transforming sociocultural patterns, such as machismo, that generate IPV. Since 2009, the Women’s Police Stations have been transformed into specialized courts on violence against women and the family, and integrated into the judicial system. Currently the penal code is under revision, with a proposal to classify violence against women as a crime rather than a minor offence (37). Despite these notable advances at policy level, published research on IPV in Ecuador is scarce, and formative unpublished research focuses on experiences of women surviving IPV and their perceptions of available services (35, 38–40).

This study aims to explore how young men in Ecuador understand men’s intimate partner violence against women (IPV), and whether young men participating in programmes promoting sexual and reproductive rights and gender equality conceptualize IPV differently from young men who do not. Gaining insight on how young men reason regarding IPV, and how this reasoning might be challenged by activism, may provide relevant input to ongoing policies and programmes aiming to transform the sociocultural patterns that sustain IPV.

## Methodology

### Study area

This study was conducted in the province of Orellana, located in the Amazon Basin of Ecuador. Orellana is a large but sparsely populated province, with an ethnically diverse and young population. The majority live in rural communities, with the main sources of income being subsistence farming, work in the oil extraction sector or in the civil service (41).

A qualitative study conducted in the region in 2000 showed that physical IPV was perceived as a main problem by women, closely linked to men’s alcohol abuse and psychological violence. Unwanted pregnancies and single motherhood were also mentioned as problematic (42). The results of this study were used to develop an intervention aiming to improve young people’s knowledge of sexual and reproductive health and their access to services, with a human rights and gender approach. The intervention was funded by UNFPA, and implemented through the local organization FUSA in collaboration with other public and private organizations and grass roots groups. It began in 2001, with some activities still ongoing. With young people, the intervention worked through the implementation of sex education in schools and through workshops with youth groups and organizations. In these workshops several issues – including sexuality, gender as socially constructed, violence, sexual diversity and reproductive rights – were discussed in an open and informal way each week throughout the year. In addition to the activities directed towards young people, the intervention implemented activities with health and educational providers and grass roots organizations, and supported the implementation of an integral centre for women’s health that included health, legal and social services for women experiencing or survivors of IPV and sexual violence (43).

Despite the many implemented activities and the advances made in terms of local policies and investment in the prevention and management of IPV, more recent research has shown that gendered structures still strongly constrain young women’s agency and sexual and reproductive freedom, and place them at increased risk of control, abuse and violence (44). However, some signs of change and resistance are also emerging, i.e. some providers criticize the moralist approach to girls’ sexuality, and young girls are aiming for financial autonomy as a way of gaining power and independence (15, 44). Our previous research exploring how activist young men construct masculinities and sexual relations shows that change towards more ‘gender-equal’ masculinities might be taking place (unpublished observation Goicolea & Öhman (45)).

### Participants and data collection

For this study, we conducted individual interviews and focus group discussions (FGDs) with two different groups of participants. The first resulted in data set 1, consisting of five individual interviews and five FGDs with 19 ordinary young men who had not participated in groups or organizations receiving training/education on gender issues. Ages ranged from 17 to 25 (mean age 21). The second resulted in data set 2, consisting of 11 individual interviews with activist young men who had participated in groups or organizations receiving training/education on gender issues and were also engaged in training/educating other young people. Ages ranged from 20 to 25 (mean age 21.5). Each group was considered a separate data set and analysed accordingly. All but one young man in each data set was single and none stated a particular sexual orientation except for one in the second data set who identified himself as gay. Educational levels and job status were similar in both groups.

The first author (IG) conducted all the individual interviews and moderated the focus group discussions. IG lived and worked in the area for more than 10 years, which facilitated access to participants. Data collection took place from December 2009 to March 2010 within a larger research project on young men’s masculinities, gender relations and health.

Both the individual interviews and FGD guides followed an open format, and several aspects were explored, such as sexuality, reproduction, fatherhood, masculinities, marriage, contraceptive use, gender relations and violence in general. Across the interviews, participants used diverse terms to refer to men’s violence against women in intimate relationships, including mistreatment, wife abuse, domestic violence, intrafamily violence and partner violence. Direct translation of these terms will be maintained in the quotations, but elsewhere we will use the term IPV when referring to men’s violence against women in intimate relationships.

The interviews were conducted in Spanish, which was the mother tongue of the interviewer and all of the respondents. Transcriptions in Spanish were entered into Open Code 3.4 for managing the analysing process (46). Two data sets were created, one for each group.

### Data analysis

For this study, all the original transcriptions in Spanish were analysed using qualitative content analysis, focusing on aspects related to IPV (47). After reading the interview transcriptions several times, meaning units that referred to IPV were identified. From the meaning units – short summarized versions of the sentences – codes were developed. For each data set, codes were grouped together to build categories. Categories reflected the manifest content, i.e. what the interview transcripts overtly expressed about IPV. Finally, from each data set one theme emerged that cut across the categories identified within each data set and reflected the latent content. All the authors were involved in the data analysis, and categories, themes and comparisons between the two data sets were negotiated and refined through discussion between them.

### Ethical considerations

The study was approved by the Bioethics Committee of the Universidad Central de Ecuador. Informed consent was obtained from all the participants. Names were erased to ensure confidentiality. During the FGDs, the moderator stressed the importance of respecting others’ opinions and maintaining the privacy of what was said within the group. Participants were encouraged to talk about their perceptions and opinions, and not necessarily about their personal experiences. However, during the interviews and group discussions some participants openly described personal experiences.

## Results

The two data sets were analyzed separately, and consequently themes and categories emerged specifically for each data set. The two emerging themes referred to how both groups established connections between gender equality/inequality and IPV. On the one hand, within the data set of the ordinary young men one theme was identified: too much gender equality leads to IPV. This theme represented how ordinary young men generally rejected IPV but justified it as men’s response to increased women’s power and autonomy. On the other hand, within the data set of the activist young men, a different theme was identified: gender inequality is the root of IPV. This theme represented how activist young men categorically rejected violence and linked the existence of IPV with gender inequality and machismo.

The themes were cut across categories that referred to young men’s level of consciousness regarding IPV, how they positioned themselves in relation to IPV, and their views and actions concerning the fight against IPV. Within data set 1 – ordinary young men – the theme ‘too much gender equality leads to IPV’ cut across three categories: acknowledging the existence of IPV, fluctuating positions on IPV and ambivalent positions on actions against IPV.

Within data set 2 – activists – the theme ‘gender inequality is the root of IPV’ cut across four categories: understanding IPV pervasiveness, connecting IPV with machismo, acknowledging (limited) social changes, and becoming personally involved and rejecting violence. Table 1 presents the themes and categories and the main differences between the two data sets.

**Table 1:** Themes and categories emerging from each data set and the main similarities and differences between them

Comparisons between the two groups	Data set 1 general young men	Data set 2 activist young men
*Connections between gender equality/inequality and IPV* While general young men in the main rejected IPV but justified it as men’s response to increased gender equality, activists categorically rejected violence and linked the existence of IPV with gender inequality.	Theme: Too much gender equality leads to IPV	Theme: Gender inequality is the root of IPV
*Young men’s level of consciousness regarding IPV* Both groups recognized the existence and harmful effects of IPV on women. However, the activists showed a deeper understanding of the complexities of IPV and the difficulties that women suffering from IPV face when they seek help or want to end an abusive relationship.	Category: Acknowledging the existence of IPV	Category: Understanding the pervasiveness of IPV
*Young men’s position regarding IPV* General young men in the main rejected IPV, but they also considered it inherent in men and understandable under certain circumstances; they did not consider controlling behaviour as IPV. Activist young men firmly rejected IPV. They considered controlling behaviour as a form of IPV and understood machismo as generating IPV.	Category: Fluctuating positions on IPV	Category: Connecting IPV with machismo
*Young men’s views and actions concerning the fight against IPV* Although general young men agreed with institutional measures against IPV, they feared that these could give too much power to women. Activist young men were knowledgeable about the institutional responses against IPV and the social changes towards lower tolerance of IPV (pushed for by the women’s movement); however, they criticized their poor implementation. Activist men felt a personal conflict between maintaining a pacifist position and avoiding responding with violence against IPV aggressors.	Category: Ambivalent positions on actions against IPV	Categories: Acknowledging (limited) social changes Becoming personally involved and rejecting violence

### Ordinary young men – too much gender equality leads to IPV

#### Acknowledging the existence of IPV

Ordinary young men did not deny the existence of IPV in Orellana: they acknowledged that IPV was very common; they did not minimize its harmful effects, and they commented on personal experience of witnessing IPV or hearing about cases of IPV from other people. They stated the strong role of the family in reproducing IPV: boys and young men learn to exercise IPV because they witness their fathers or other male relatives exercising violence against their intimate female partners. IPV was described as an escalating process, where insults led to arguments, and ended up in physical violence. In this process jealousy was considered a strong trigger of IPV.

These things happen when he starts reproaching her, then he insults her, then it leads to fighting, and then comes the slap. That happens because there is an argument, a fight, because she cheated on him, or he saw her kissing another boy … (Interview 1)

Participants identified IPV as occurring within stable (adult) couple relationships, even if some references were made to violence between young people in boyfriend–girlfriend relationships. IPV was associated with ignorance, with some participants remarking that IPV was more common among poor uneducated people, and that it was less tolerated nowadays than in the past:

Mistreatment is related to poverty. Since they are little boys from poor families, they are raised wrongly … education makes you understand that you shouldn’t do that, that there are ways to solve it. And it also depends on the person’s education. In the family, with the parents, how they educate their children. I mean, if the father was a *machista* and beat his wife, then the son will learn to do those bad things, too. (FGD 1)

#### Fluctuating positions on IPV

Across the interviews there were remarks against violence in general, and a call to solve conflicts through dialogue, never resorting to violence of any kind. Men who committed physical violence against women were labelled ‘*machistas*’ and criticized. Violence was not considered a solution and was never justified, even in cases of flagrant infidelity. Separation and divorce were considered much better options than violence; separation was also felt to be best for the children. IPV was perceived as negatively affecting all members of the family equally, rather than solely women:

It is better to divorce than letting the beatings continue […] It seems that if children see how the couple mistreat each other, they suffer more, then it is better to separate, and the children will decide who they’ll follow, either the father or the mother. (Interview 2)

There were also remarks against IPV not based on an overall rejection of violence, but on the view that women were a vulnerable group who needed men’s protection. It was acceptable for a man to fight another man – since they were at the same level – but not to fight people who were viewed as weaker, such as women or children. That was a gentleman’s position, implying that real men would never hit a woman, but would always be willing to fight another man to protect her.

I like to defend women […] If a man is beating his wife, and he is not my friend, I will beat him, I will give him some of the same to make him understand that a man should never hit a woman, and that he should fight a man if he wants to fight. (Interview 3)

Finally, even if IPV was generally rejected, it was also constructed as understandable, inherent in men’s impulsive nature. Even if they criticized it, participants declared reasons that could justify IPV, such as infidelity. Participants also referred to how women could tease and rile men until they were beaten; in that sense they felt that physical IPV could be a way of ‘calming down’ women.

I was there and they were having an argument […] and he told her that he was going to beat her, that he was going to slap her … and she teased him ‘Go on, beat me, beat me. You’re not man enough to beat me’, she told him. Then he raised his hand and ‘Bam’, he slapped her in the face, and the woman just shut up and calmed down. There are women like that who like to goad men. (FGD 3)

Controlling behaviour was not considered IPV and the connections between the two were not established by the participants. Even if there were expressions criticizing men who considered themselves the owner of their female partners, men’s controlling behaviour was strongly taken for granted. They distinguished between being abusive, prepotent and violent, being what they called ‘*machista*’, which was generally considered bad, and being controlling and holding the reins, which was accepted and promoted.

I am not a *machista*, because it’s one thing to be a *machista* but a very different one to be authoritarian! I’m authoritarian, because I have authority over my partner. (FGD 1)

To minimize the risk of IPV, women should be accountable to men, behave in a respectable way, avoid raising suspicion and be able to ‘manage’ men’s impulsive behaviours:

The woman is acting wrong. If there’s a problem, they have to try and solve it together. They are both wrong, the man because he beats her, but the woman, too: she is even worse, because instead of reasoning … She should try to foresee what might happen and behave accordingly. (FGD 1)

Ambivalence and contradictions were present not just between participants but also in the accounts of individual participants, fluctuating from justifying IPV, to rejecting violence out of hand, to adopting the gentleman’s position, as seen in the quote below from one participant in a FGD:

Violence against women is the worst thing possible …, it’s the worst thing a man can do …, I would hang those motherfuckers … I mean, a woman can’t defend herself. It’s deplorable, any mistreatment of a woman by a man … Although sometimes I think there are some women who … I don’t know …, they go beyond what a man can tolerate. There are women that keep on and on, and then you feel you need to grab hold of her and punch her. (FGD 3)

#### Ambivalent positions on actions against IPV

Participants’ positions regarding actions against IPV were also ambivalent and contradictory. On getting personally involved in cases of IPV, participants expressed how they felt it deeply and reacted with rage. They described how they could get in between the couple to stop the fight, or even beat the aggressor. However, they also stated that they were reluctant to get involved in an issue that would likely bring them trouble. As one participant pointed out, ‘He [the aggressor] can ask you: “What’s it got to do with you? Are you her lover or something?”’ (FGD 4)

Between these two positions, there were also vague allusions to trying to reason with the aggressor, to talk with him, appeal to him and question him regarding his reasons for engaging in IPV.

Participant A: [when faced with a case of a man beating his girlfriend in the street] I would go to help, I would ask him ‘What’s going on?’ and shout at him if he was going to beat her.

Participant B: I would talk to him, try to make him see sense, talk with him, have a dialogue. (FGD 4)

Participants were aware of the state’s legal responses to punish IPV, and preferred to resort to those means rather than getting physically involved when faced with cases of IPV. References to the Women’s Police Station were constantly made, and here again ambivalence and contradiction emerged. On the one hand, participants acknowledged the need for a legal response to protect women exposed to IPV. They recognized IPV as a problem where police and law enforcement structures should intervene, and they acknowledged that the Women’s Police Station had been beneficial for women. The legal measures that a woman exposed to IPV could make use of were considered to give power to women. On the other hand, participants expressed caution regarding the law enforcement measures against IPV. They claimed that women were taking advantage of these measures, or even ‘abusing’ them to oppress men. The Women’s Police Station was considered to be biased in favour of women, and was portrayed more as a way of exacting revenge than justice.

[The Women’s Police Station] is very good, it’s a way of enforcing women’s rights, backing women … Even if I have to say that nowadays women resort too much to that, and sometimes they abuse it.[…] there are cases when the man is calm, but he just touches his wife and she runs for a restraining order and he ends up in jail. That’s bad, because there are women who abuse that authority. (Interview 1)

The existence of these legal measures also led participants to consider that women now had no excuses for not reacting against IPV. Some comments blaming women who did not ‘take action’ against violent partners were made.

### Young activist men – gender inequality is the root of IPV

#### Understanding the pervasiveness of IPV

The participants described IPV against women as widespread, and references to personal experiences in their own families were common. Their understanding of IPV went beyond physical abuse to include psychological, sexual and economic violence:

I knew a case from a friend. She was thrown out of the house by her husband, and they were married, and after seven years he threw her out. And she didn’t have any rights to the house or anything, because she was ‘not working’ [quotation marks made by participant] during the seven years …, but I mean, she had contributed as well during those years … There is a lot of violence, and discrimination. Violence doesn’t just come in the form of beating, but also in psychological and economic mistreatment. Women are economically tied because their husbands never share a cent, and they are reminded that they are ‘not working’ [quotation marks made by participant]. (Interview 1)

Even if IPV was mainly portrayed as occurring between formal cohabiting partners, they also referred to violence within boyfriend–girlfriend relationships, especially sexual violence.

Participants did not express any blame towards women who stayed in violent relationships. On the contrary, the barriers and difficulties that those women could face were recognized. The judicial system, police and other institutions were criticized for their lack of sensitivity in dealing with these issues, and were considered a reason for the persistence of IPV:

[Regarding IPV] the legal and other authorities are not well prepared to carry out that role, that’s the way I see it. You go there to report a case of intrafamily violence, and they tell you that it is the woman who should come, she has to report it …, I mean the husband can be about to kill her and they want her to come and inform them … We still don’t have authorities who are really sensitive to the issue. (Interview 2)

Criticisms referred not only to the poor implementation of protection measures and the unsympathetic attitudes of those dealing with IPV in public institutions, but also to the way the system itself was structured. Participants stated that the judicial system was structured in a way that favoured men economically in cases of separation or divorce, and which lessened men’s financial (and other) responsibilities towards their children if they did not cohabit with the mother.

#### Connecting IPV with machismo

The participants connected IPV with women’s subordination to men and machismo. They strongly criticized and rejected machismo, referring to it as ‘Man’s immaturity that makes him feel above everybody else …, believing he is superior to everybody else, superior to women, and consequently marginalizes and discriminates against women’ (Interview 4). Participants distanced themselves from ‘*machista*’ men, criticizing them as resorting to violence as a way of being dominant, of ‘showing who is in charge’ and always ‘getting away with it’. Participants portrayed ‘*machista*’ men as narrow-minded, stubborn and unreasonable in their attitudes and actions. Machismo was considered a form of violence in itself, as well as the root of IPV and other forms of gender-based and even social violence. Connections between machismo, women’s subordination, controlling behaviour, and IPV were made across the interviews:

I think it’s wrong if a man beats his wife. That’s because he is too *machista*, he thinks that he is the one that rules, that he is the owner of the family, the boss, the one in charge …, that’s the root of violence, because it means, like, women are useless […] Those are the men that abuse women, who say that men are the ones that rule, because they are the ones who are earning …, that women are not able to work, that women shouldn’t have a job but rather stay at home and do this and do that …, and he can come home drunk and turn up the music loud and so on … (Interview 3)

#### Acknowledging (limited) social changes

Participants acknowledged that progress had been made on IPV in the country. They were familiar with policies, programmes, campaigns and institutions dealing with IPV and felt that they had helped to reduce the number of cases and severity of IPV. They considered that this change has taken place mainly as a result of pressure by women, who had become less tolerant of IPV. Supported by emerging progressive policies and spaces to exercise their right to denounce, women were becoming more aware of their right to live free from violence and were increasingly demanding this right:

In the past women may have said, ‘If he beats me, if he kills me, he’s still my husband’ … and they put up with it. But now women are much more aware …, and they don’t put up with violent behaviour, they leave. (Interview 1)

Participants felt that men in general had been forced to change and reduce their exercise of IPV. The activist young men considered that this change had occurred because men in Ecuador generally were afraid of the consequences, namely being reported to the police followed by prison. There was also an increased social rejection of men who were violent towards their partners:

Change has happened. Compared to old times, how our fathers behaved …, young men are not like that. Because there have been a lot of campaigns and this has increased the fear among young men. I think that awareness raising among young men is working, regarding not engaging in violent behaviour … But I think that the majority of young men react more because of fear, fear of going to prison, fear of being reported to the police. I don’t think it’s because they truly assume the change, that they have become more responsible and aware of women’s rights. (Interview 5)

#### Becoming personally involved and rejecting violence

Participants recounted several occasions when they had witnessed and got involved in stopping IPV. Their knowledge was not theoretical but based on actual experience, and one that raised strong emotions. They described IPV as something that enraged them, or something that made them feel impotent, and definitely something that moved them to act. Acting could mean becoming personally involved themselves, calling the police or other law enforcement agencies, or advising women they knew about services and procedures available.

I always get involved, because it enrages me. And even if you advise the woman to report it …. I mean, I have advised my relatives like that, ‘Go, report him, it shouldn’t be like this’ …, but sometimes it’s much more complex, because they may answer that he is supporting her [financially], or that she loves him …, and I always argue that that can’t be called love, nobody who loves you hits you. I can’t understand why that happens. And it really makes me angry. The other day I earned myself a punch in the street, because there was a man hitting a young woman and I faced up to him and said: ‘Why are you hitting her?’ And he punched me and told me it was none of my business. (Interview 1)

Participants’ rejection of IPV was based on a general rejection of violence as a means to resolve conflicts. Participants took a pacifist stance, repeatedly stating that violence was never justified, that nothing is solved by fighting, that nobody has the right to beat others and that it does not mean that you are not a man if you do not retaliate:

Boys learn that a man’s attitude is that if somebody shouts at him, if somebody strikes him, then he should respond like a man, hitting back … I mean, as if beating someone up was a ‘man’s attitude’. But I think that I’m still a man even if I decide not to retaliate. I mean, what’s the point? If somebody is going to hit me, what is the point of squaring up to him? I will solve nothing by getting into a fight. (Interview 5)

The participants rejected not only IPV, but also violence against other men, or punishment of children as a way of ‘teaching them a lesson’. In this sense, they described a process of ‘learning to control their own violent behaviour’, and how participation in groups and organizations had changed their approach to problems, frustrations and disappointments. This meant taking an analytical stance and not resorting to violence:

I have changed a lot from participating in these workshops. Especially regarding the issue of violence, especially on that. I mean, before I was so violent, very violent, half a word and I would react badly. But since I started this, because we talked about the types of violence, machismo and all that, I have changed quite a lot, a hundred percent. (Interview 6)

However, maintaining a pacifist position was difficult when confronted with cases of IPV – when they were in two minds about whether to fight the aggressor, or stay calm and appeal to him, or report it to the authorities, knowing that the authorities might not be sympathetic and helpful in such cases.

## Discussion

Young men in this study – both ordinary young men and activists – recognized the existence and harmful effects of IPV, rejected it and distanced themselves from men who committed IPV. This contrasts with previous research by Segura (35) conducted with indigenous communities in the neighbouring province of Sucumbíos. In Segura’s study, adult men naturalized IPV and trivialized its pervasiveness and harmful effects. This divergence could be due to the fact that our participants were younger, better educated and living in a semi-urban area where access to information and services – including those dealing with IPV – was easier. It may also be a sign that existing policies and regulations – especially Law 103 and the Women’s Police Stations – have influenced how people conceptualize IPV. In fact, several studies have shown that changes towards less tolerance of IPV among men are also taking place in other Latin America settings (48–50).

Although both groups in our study rejected IPV, their positions differed, and we hypothesize that this is significant. Ordinary young men had an ambivalent view of IPV, justifying it under certain circumstances, while activist men were categorical in their rejection of it. Gender relations were strongly present in the accounts of both ordinary young men and activists. However, the way they were constructed differed radically: activists considered gender inequality to be the cause of IPV and aimed to challenge sexism, while ordinary young men justified IPV when gender inequality and men’s dominance were threatened. For ordinary young men, IPV could be a way of placating women’s attempts to gain power. In that sense, IPV was viewed as a last resort when men could not maintain their supremacy by other means. IPV was rejected because real men should not need to use it in order to maintain their hegemony, and good women should know how to behave in order to avoid threatening the status quo. This is in line with Connell’s gender-power theory, which portrays violence as not only part of the system of patriarchal domination, but also as a measure of its deficiency (21, 51). Other authors have also pointed out that IPV can be triggered as a reaction against increased female autonomy (7–9).

The accounts of ordinary young men in our study support ambivalent sexism theory (10–12): these young men support benevolent sexism, but may resort to hostile sexism and violence if the former is threatened. Their rejection of IPV is conditional on the maintenance and naturalization of men’s power over women. McCarry, exploring young people’s understandings of IPV in Scotland, also found a similar pattern: young men rejected IPV but at the same time justified it because they considered that men were naturally violent and socially entitled to it (26).

Parallelisms with how Connell and other authors describe the complex process through which hegemonic masculinity adapts and incorporates features of other masculinities – without challenging the sexist structure that is produced and reproduced through the hegemony schema – in order to sustain its ascendancy can be noticed (21, 22). In that sense, the ordinary young men’s rejection of IPV could be more a sign of the ability of hegemonic masculinity to adapt to social changes, maintaining its preeminent and normative position. The ascendency of benevolent sexism over hostile sexism does not contribute to the eradication of IPV; instead it constructs gender equality as a threat to stability and a justification for the exercise of IPV.

Research shows that men who show greater support of gender equality are less likely to engage in IPV (6, 7, 27). Several studies have pointed out that interventions aimed at primary prevention of IPV by young men are much more effective than interventions attempting to change the behaviours of batterer men (30, 52, 53). An increasing number of academic and formative research studies show that among certain groups of men, real change towards increased gender equality and non-violent intimate relationships could be possible (6, 25, 27–29, 31). The activist men in our study firmly rejected machismo, supported gender equality, and considered that IPV was never justified. For them, IPV was rooted in machismo, and machismo was understood as men’s dominance over women. We argue that this is a more substantial change in the direction of eradicating IPV. Even if we were not ascertaining actual behaviour, we can assume that activist young men were less likely to engage in IPV compared to ordinary young men. Challenging machismo and taking a stance in favour of gender equality and against IPV was not a matter of chance, but the effect of engagement in programmes or groups that encouraged men to critically reflect on traditional masculinities and unequal gender relations. Similar to the process in Orellana, interventions underway in different countries show promising results (6, 25, 28–31). We do not claim that the intervention was the ‘sole’ and direct cause of this change – activist young men may have been different from ordinary young men before the intervention – indeed, that difference may have led to their participation. What we claim is that there is a difference in the way the two groups understand IPV, and that raising gender-consciousness seems to be connected with a deeper understanding and with being aware of the connections between gender inequality and IPV.

Diminished social tolerance and increased rejection of IPV may not be sufficient in themselves. Rejecting IPV without challenging the gender-power relations that support it is possible. In fact, proponents of gender symmetry on domestic violence criticize the feminist argument that IPV is gendered and disproportionately affects women (54). Such an approach to IPV might be less contentious to implement. However, we agree with other authors that it would also be less effective in eradicating IPV (51).

Degendering IPV means addressing all cases of IPV as if they are ‘common couple violence’ and negating the existence of cases of ‘intimate terrorism’. Intimate terrorism – defined by Johnson as ‘a terroristic control of wives by their husbands that involves the systematic use of not only violence but economic subordination, threats, isolation and other control tactics’ – is rooted on patriarchy (55) (p. 284). Intimate terrorism has more devastating and long-lasting effects on direct victims (the majority of them women) – and children and adolescents who witness the violence – than common couple violence (55, 56). At least in this setting, IPV is constructed as strongly gendered and as a means for maintaining patriarchy and sexism in times of significant changes in gender relations. Ignoring that fact that challenging gender relations is at the root of challenging IPV does not seem useful. In fact, the latest national plan and 2007 presidential decree constitute strong efforts to engender IPV in that they refer explicitly to ‘gender violence against women’ and to ‘machista violence’. They focus not only on punitive measures but aim to challenge social norms that sustain women’s discrimination and violence within intimate relationships (36, 57).

An additional finding from this study is that IPV was still generally understood as physical abuse happening to adult women in cohabiting relationships. Even if some allegations of women’s violence against men were made, the gender asymmetry of IPV was acknowledged, contrary to findings from other settings that claim that young people are more critical of the feminist position that IPV disproportionally affects women (26). Violence among young people in informal relationships, date rape and other forms of IPV that may be more frequent among young people were cited but not strongly perceived as part of IPV. This may reflect how policies, programmes and campaigns against IPV have failed to consider the specificities of IPV among young people and target this audience (26, 27).

### Methodological considerations

This study was based in a specific setting – the Amazon of Ecuador – with a significant proportion of the population living in poverty, and where subsistence agriculture coexists with major foreign industries with minimum local investment. It is also a setting where national and local policies and programmes against IPV have flourished and women’s access to education and the workforce has increased, but where machismo and *marianismo* remain strongly influential in the way gender relations are constructed and IPV is far from being eradicated. We claim that many settings in Latin America and (arguably) in other low income contexts share these characteristics, and thus our results may be transferable to them.

Triangulation of researchers – bringing different perspectives by having different backgrounds and degrees of familiarity with the setting – and prolonged engagement (two authors lived in the area for several years) enhanced the study’s credibility (58). In addition, we carried out peer debriefing by discussing preliminary results in workshops held with young people, providers and stakeholders both in Orellana and Quito.

As we have described before, this research was part of a larger study in which exploring IPV was not the main focus; thus, we may have failed to inquire in greater depth on relevant issues. A further limitation may arise from the use of the term IPV, which may not have encompassed the diversity of concepts and wordings that the participants used to refer to, what we interpreted as, men’s violence against women within an intimate relationship.

However, the central role of IPV in the construction of masculinities and gender relations, and the qualitative differences in how activist and ordinary young men constructed IPV, emerged from the data, and this was what motivated this study. We argue that following an emergent design adds to the study’s dependability, which contributes to research trustworthiness (58).

During the interviews and focus group discussions, the interviewer (IG) tried to create a conducive environment and the participants said that they enjoyed the discussions. However, the fact that the interviewer was a woman, and was identified with activist work on sexual and reproductive rights by some of the participants, may have led to more socially desirable responses. Further, the fact that we used mainly FGDs with the ordinary young men and individual interviews with the activists may also have influenced the results. However, we did this because we assumed that the activists might be more familiar with interviews and more at ease with the questions and topics, while the ordinary young men might feel less threatened in a group situation.

Within FGDs, the group dynamic can influence the answers of some members, and participants with a leadership position might have more influence than others. However, during all the FGDs that were conducted, young men participated actively and different opinions and contradictions emerged, as shown in the results. The fact that the number of participants was larger in the first data set than in the second is an additional limitation of the study. However, if we consider each FGD as a unit of analysis, then the difference in sample size is minimal. It is also important to notice that activist young men are still a small group within the general young men’s population and consequently it does not exist an ample population of activist young men from whom to choose a large number of potential participants.

## Conclusions

The young men in this study generally recognized the existence and harmful effects of men’s intimate partner violence against women. They rejected it and criticized male aggressors. Despite these commonalities, the differences between the two groups of men were remarkable. IPV rejection was categorical among the activists but milder among ordinary young men, allowing the latter to justify IPV under certain circumstances. The way the groups understood IPV was also very different. While activist men considered IPV to be rooted in gender inequality, ordinary young men considered IPV as a way of solving conflicts generated by women’s attempts to gain greater independence and power.

This study shows on the one hand, that policies and programmes against IPV might reduce social tolerance of IPV. However, if they fail to engender IPV they may not succeed in addressing the unequal gender structures that sustain it. On the other hand, programmes and interventions generating gender-consciousness among young men might lead to more profound changes by challenging gender inequalities and sexism as the roots of IPV. Scaling up such interventions may have a stronger impact.

An additional recommendation for strengthening programmes aimed at preventing IPV might be to increase awareness of all forms of violence – not only physical abuse. Finally, these programmes might benefit from highlighting that IPV can also happen among young people and in casual relationships, and to target this audience accordingly.

## References

[1] World Health Organization (2010). Preventing intimate partner and sexual violence against women: taking action and generating evidence. http://www.who.int/violence_injury_prevention/publications/violence/9789241564007_eng.pdf.

[2] Ellsberg M, Jansen HA, Heise L, Watts CH, Garcia-Moreno C (2008). Intimate partner violence and women’s physical and mental health in the WHO multi-country study on women’s health and domestic violence: an observational study. Lancet.

[3] Krantz G, Garcia-Moreno C (2005). Violence against women. J Epidemiol Commun Health.

[4] Fuller N, Public policies against IPV (2010). Where are we twenty years later? [Políticas pública contra la violencia conyugal, Dónde estamos veinte años después?] Sexualidad. Salud y Sociedad: Revista Latinoamericana.

[5] McPhail BA, Busch NB, Kulkarni S, Rice G (2007). An integrative feminist model: the evolving feminist perspective on intimate partner violence. Violence Against Women.

[6] Pulerwitz J, Barker G (2008). Measuring attitudes toward gender norms among young men in Brazil – development and psychometric evaluation of the GEM Scale. Men Masc.

[7] Abramsky T, Watts CH, Garcia-Moreno C, Devries K, Kiss L, Ellsberg M (2011). What factors are associated with recent intimate partner violence? Findings from the WHO multi-country study on women’s health and domestic violence. BMC Public Health.

[8] Jewkes R (2002). Intimate partner violence: causes and prevention. Lancet.

[9] Allen CT, Swan SC, Raghavan C (2009). Gender symmetry, sexism, and intimate partner violence. J Interpers Violence.

[10] Glick P, Fiske ST (1996). The ambivalent sexism inventory: differentiating hostile and benevolent sexism. Journal of Personality and Social Psychology.

[11] Glick P, Fiske ST (2001). Ambivalent sexism. Adv Exp Soc Psychol.

[12] Glick P, Fiske ST, Mladinic A, Saiz JL, Abrams D, Masser B (2000). Beyond prejudice as simple antipathy: hostile and benevolent sexism across cultures. J Pers Soc Psychol.

[13] Torres JB, Solberg VS, Carlstrom AH (2002). The myth of sameness among Latino men and their machismo. Am J Orthopsychiat.

[14] Viveros Vigoya M (2001). Contemporary Latin American perspectives on masculinity. Men Masc.

[15] Goicolea I, Wulff M, Ohman A (2010). Gender structures constraining girl’s agency – exploring pregnancy and motherhood among adolescent girls in Ecuador’s Amazon basin. Sexualidad, Salud y Sociedad: Revista Latinoamericana.

[16] Gutmann MC (1996). The meanings of macho.

[17] Gutmann MC (2003). Changing men and masculinities in Latin America.

[18] Lancaster R (1992). Machismo, danger and the intimacy of power in Nicaragua. Life is hard.

[19] Troya MP, Andrade X, Herrera G (2001). I am not machista but … Masculinities among middle class profesionals in Quito [No soy machista pero … Masculinidades en profesionales de clase media en la ciudad de Quito]. Masculinities in Ecuador [Masculinidades en Ecuador].

[20] Connell RW (1987). Gender and power.

[21] Connell RW (1995). Masculinities.

[22] Connell RW, Messerschmidt JW (2005). Hegemonic masculinity. Rethinking the concept. Gender Soc.

[23] Yllo K, Gelles RJ, Loseke DR (1993). Through a feminist lens: gender, power, and violence. Current controversies on family violence.

[24] Stark E (2007). Coercive control: how men entrap women in personal life.

[25] United Nations (2008). The role of men and boys in the achievement of gender equality [El papel de los hombres y los niños en el logro de la igualdad entre los géneros]. http://www.un.org/womenwatch/daw/public/w2000/08-52641_Women2000_SP_FIN.pdf.

[26] McCarry MJ (2009). Justifications and contradictions: understanding young people’s views of domestic abuse. Men Masc.

[27] Gomez AM, Speizer IS, Moracco KE (2011). Linkages between gender equity and intimate partner violence among urban Brazilian youth. J Adolesc Health.

[28] Casey E, Smith T (2010). ‘How Can I Not?’: men’s pathways to involvement in anti-violence against women work. Violence Against Women.

[29] Casey EA, Ohler K (2012). Being a positive bystander: male antiviolence allies’ experiences of ‘stepping up’. J Interpers Violence.

[30] OLeary KD, Smith Slep AM (2011). Prevention of partner violence by focusing on behaviors of both young males and females. Prev Sci.

[31] Ricardo C, Barker G, Pulerwitz J, Rocha U, Ingham R, Aggleton P (2006). Gender, sexual behaviour and vulnerability among young people. Promoting young people’s sexual health: international perspectives.

[32] CEPAR. ENDEMAIN (2005). Demographic and maternal health national survey, 2004 [ENDEMAIN, 2004: Encuesta Nacional de Demografía y Salud Materno Infantil, 2004].

[33] Jubb N, Camacho G, D’Angelo A, Yáñez G, Hernández K, Macassi I (2008). Regional mapping of women’s police stations in Latin America [Mapeo regional de las comisarías de la mujer en América Latina].

[34] Congreso Nacional del Ecuador (1995). Law 103 against violence against women and family [Ley 103 contra la violencia contra la mujer y la familia].

[35] Segura Villalba M (2006). Resolution or silence? Violence against kichwa women in Sucumbios [¿Resolución o silencio? La violencia contra las mujeres kichwas de Sucumbios].

[36] Presidencia de la República del Ecuador (2007). Decree 620 for the eradication of gender based violence against children, adolescents and women [Decreto 620 Erradicación de la violencia de género hacia la niñez, adolescencia y mujeres].

[37] Camacho G, Hernández K, Redrobán V (2009). Women’s police stations; a path towards justice? A case study from Cuenca county [Las comisarías de la mujer ¿un camino hacia la justicia? Estudio de caso en el cantón Cuenca].

[38] Salgado J (2009). Violence against indigenous women: between ‘justices’ and lack of protection [Violencia contra las mujeres indígenas: entre las ‘justicias’ y la desprotección].

[39] Vaca C (2010). The implications of denouncing: the administration of justice and subjectivity [Las implicaciones de la denuncia: administración de justicia y subjetividad. Investigación realizada en las comisarías de la mujer del distrito metropolitano de Quito 2007–2010].

[40] Balbuera P (2004). Justice has no woman’s face. Women’s obstacles for access to justice [La justicia no tiene rostro de mujer. Obstáculos para el acceso a la justicia de las mujeres].

[41] Honorable Consejo Provincial de Orellana (2007). Demographic, health and environmental information system in the province of Orellana. Base line 2006 [Sistema de información en demografía, salud y ambiente en la provincia de Orellana. Línea de base 2006].

[42] Goicolea I (2001). Exploring women’s needs in an Amazon region of Ecuador. Reprod Health Matters.

[43] UNFPA Ecuador (2004). Bodies, drums and footprints. Compilation of sexual and reproductive health projects in Ecuador 2000–2003 [Cuerpos, Tambores y Huellas. Sistematizacion de Proyectos de Salud Sexual y Reproductiva 2000–2003 en Ecuador].

[44] Goicolea I, Wulff M, Sebastian MS, Ohman A (2010). Adolescent pregnancies and girls’ sexual and reproductive rights in the Amazon basin of Ecuador: an analysis of providers’ and policy makers’ discourses. BMC Int Health Hum Rights.

[45] Goicolea I, Ohman A Macho, gentleman or something else? Young Ecuadorian’s men’s attempts to dismatle machismo.

[46] Open Code 3.4 Software UMDAC and Division of Epidemiology and Public Health Sciences, Department of Public Health and Clinical Medicine, Umeå University.

[47] Graneheim UH, Lundman B (2004). Qualitative content analysis in nursing research: concepts, procedures and measures to achieve trustworthiness. Nurse Educ Today.

[48] Salazar M (2011). Intimate partner violence in Nicaragua – studies on ending abuse, child growth, and contraception.

[49] Sternberg P (2008). Damned if they do, damned if they don’t: tensions in Nicaraguan masculinities as barriers to sexual and reproductive health promotion. Men Masc.

[50] Welsh P (2010). Community development: a gendered activism? The masculinities question. Community Dev J.

[51] Kimmel MS (2002). ‘Gender symmetry’ in domestic violence – a substantive and methodological research review. Violence Against Women.

[52] Foshee VA, Bauman KE, Greene WF, Koch GG, Linder GF, MacDougall JE (2000). The safe dates program: one-year follow-up results. Am J Public Health.

[53] Wolfe DA, Crooks C, Jaffe P, Chiodo D, Hughes R, Ellis W (2009). A school-based program to prevent adolescent dating violence a cluster randomized trial. Arch Pediatr Adolesc Med.

[54] Archer J (2000). Sex differences in aggression between heterosexual partners: a meta-analytic review. Psychol Bull.

[55] Johnson MP (1995). Patriarchal terrorism and common couple violence – two forms of violence against women. J Marriage Fam.

[56] Johnson MP (2006). Conflict and control – gender symmetry and asymmetry in domestic violence. Violence Against Women.

[57] Con Vivir (2009). Plan for the eradication of gender based violence against children, adolescents and women [Plan para la erradicación de la violencia de género hacia la niñez, adolescencia y mujeres].

[58] Lincoln Y, Guba E (1985). Naturalistic inquiry.
